# Cooperative Catalysis for the Highly Diastereo‐ and Enantioselective [4+3]‐Cycloannulation of *ortho*‐Quinone Methides and Carbonyl Ylides

**DOI:** 10.1002/anie.201913603

**Published:** 2020-01-23

**Authors:** Arun Suneja, Henning Jakob Loui, Christoph Schneider

**Affiliations:** ^1^ Institut für Organische Chemie Universität Leipzig Johannisallee 29 04103 Leipzig Germany

**Keywords:** asymmetric synthesis, carbonyl ylides, cooperative catalysis, cycloannulation, *ortho*-quinone methides

## Abstract

We describe herein a highly diastereo‐ and enantioselective [4+3]‐cycloannulation of *ortho*‐quinone methides and carbonyl ylides to furnish functionalized oxa‐bridged dibenzooxacines with excellent yields and stereoselectivity in a single synthetic step. The combination of rhodium and chiral phosphoric acid catalysis working in concert to generate both transient intermediates in situ provides direct access to complex bicyclic products with two quaternary and one tertiary stereogenic centers. The products may be further functionalized into valuable and enantiomerically highly enriched building blocks.

## Graphical abstract

**It takes two**: A combination of Rh_2_(OAc)_4_ and a chiral phosphoric acid catalyzes the in situ generation of transient carbonyl ylides and chiral hydrogen‐bonded *ortho*‐quinone methides through cooperative catalytic cycles. These transient species then undergo a highly diastereo‐ and enantioselective cycloannulation to afford a broad range of functionalized oxa‐bridged dibenzooxacines featuring two quaternary and one tertiary stereogenic centers.

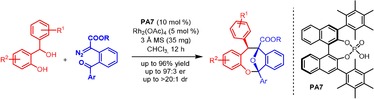

Oxa‐bridged heterocyclic skeletons are ubiquitously present in numerous natural products and bioactive molecules.^1^ Therefore, the development of new, efficient, and stereoselective synthetic methods towards their rapid construction is highly desirable. Extensive efforts have previously been directed at carbo‐bridged bicyclic frameworks through Lewis‐acid‐catalyzed intramolecular Diels–Alder (IMDA) reactions, molecular rearrangements involving a ring‐opening/closure tandem process, free‐radical reactions, and transition‐metal‐catalyzed annulation reactions.^2^ However, there still remains a high demand for the synthesis of oxa‐bridged heterocycles.^3^

The combination of a transition‐metal catalyst and a Lewis acid or organocatalyst to activate two different substrates for a given reaction has attracted significant interest among synthetic organic chemists recently since it potentially enables highly efficient and/or unprecedented complex chemical transformations in a one‐pot operation.^4^ The success of this strategy relies upon the simultaneous activation of two reacting partners by two different catalysts that operate in concert in two distinct catalytic cycles.4c A prominent early example is the work of Hu, Gong, and co‐workers on cooperative Rh‐/chiral phosphoric acid catalyzed multicomponent reactions of α‐diazoesters, alcohols, and imines, which were converted into α‐hydroxy β‐amino esters with excellent enantio‐ and diastereocontrol.^5^ In another example, Terada et al. developed an elegant carbonyl ylide formation/reduction sequence towards isochromanones under cooperative Rh‐/chiral phosphoric acid catalysis.^6^

Carbonyl ylides generated from carbonyl compounds and a rhodium carbene complex are classically considered as highly reactive transient species and are widely employed in 1,3‐dipolar cycloaddition reactions with a wide variety of 2π‐systems.^7^, ^8^ However, their reactivity with 4π‐systems is still underexplored due to the challenges associated with entropy factors and strain aspects in the formation of seven‐membered rings.^9^, ^10^

*Ortho*‐quinone methides (*o*‐QMs) feature a particularly reactive 4π‐system and have increasingly been exploited as versatile synthetic intermediates for the construction of complex heterocycles.^11^ In recent years, we and others have meticulously developed Brønsted acid catalyzed reactions of *o*‐QMs with a wide range of typically 2π‐nucleophiles, leading to a broad range of benzannulated oxygen heterocycles with good to excellent stereocontrol.^12^, ^13^

We now report the first cooperative, catalytic, enantioselective [4+3]‐cycloannulation of *o*‐QMs and carbonyl ylides to afford complex and enantiomerically highly enriched oxabicyclic dibenzooxacines. We envisioned that a chiral phosphoric acid would easily form hydrogen‐bonded *o*‐QM **A** starting from *ortho*‐hydroxy benzylalcohol **1** in one catalytic cycle, while in a second and separate catalytic cycle, carbonyl ylide **B** would be generated through Rh‐catalyzed decomposition of a α‐diazoester **2** tethered to an aryl ketone (Scheme 1). The decisive question here was whether both transient intermediates **A** and **B** formed in only very low amounts would have sufficient stability and lifetime to undergo the desired [4+3]‐cycloannulation and provide the product **3** with good stereocontrol in the chiral environment provided by the phosphoric acid catalyst.

**Scheme 1:**
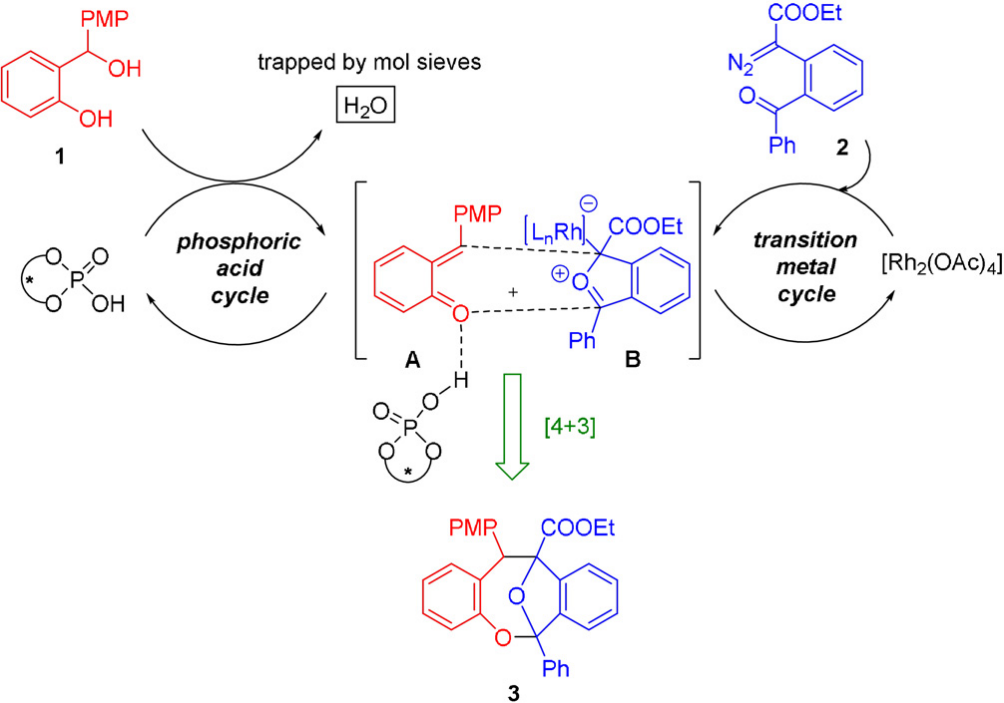
Design plan for the reaction between *o*‐QMs and carbonyl ylides through cooperative Rh/phosphoric acid catalysis.

Enantioselective [4+3]‐cycloadditions of *o*‐QMs were first described independently by the groups of Scheidt and Ye under chiral NHC catalysis to produce benzoxopinones.14a, 14b Very recently, the first example of a phosphoric acid catalyzed enantioselective reaction of *o*‐QMs with 2‐indolylmethanols as 1,3‐dipoles toward indolylbenzoxepins was established by Shi et al.14c An interesting study by the Lautens group described a purely Brønsted acid catalyzed diastereoselective synthesis of oxa‐bridged oxazocines through cycloaddition with isomünchnones.14e

To test our hypothesis, we initiated our investigations with the model reaction between benzhydryl alcohol **1 a** and α‐diazoester **2 a** in the presence of 5 mol % of Rh_2_(OAc)_4_ and chiral phosphoric acid **PA1** (10 mol %) in CHCl_3_ at room temperature. We were delighted to obtain the desired product **3 a** in 77 % yield after 12 h with moderate diastereo‐ and enantioselectivity (Table 1, entry 1). Importantly, diazoester **2 a** had to be added slowly for a period of 1 h using a syringe pump to avoid side reactions of the transient carbonyl ylide.

**Table 1:** Catalyst screening and optimization studies.^[a]^ 
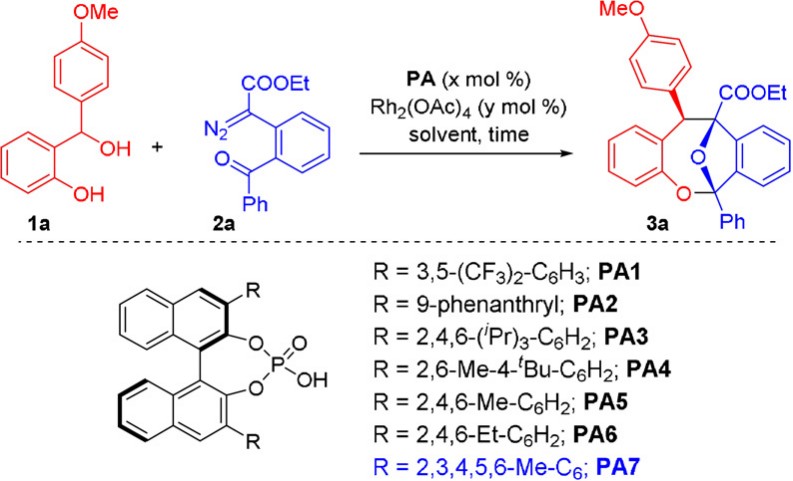

Entry	**PA**	Solvent	Time [h]	**3 a** (%)^[b,c]^	er^[d]^	dr^[e]^
1	**1**	CHCl_3_	12	77	66:34	16:1
2	**2**	CHCl_3_	12	71	82:18	20:1
3	**3**	CHCl_3_	12	75	69:31	10:1
4	**4**	CHCl_3_	12	79	83:17	20:1
5	**5**	CHCl_3_	12	80	84:16	20:1
6	**6**	CHCl_3_	12	73	88:12	20:1
7	**7**	CHCl_3_	12	79	92:8	20:1
8	**7**	CH_2_Cl_2_	12	83	83:17	20:1
9	**7**	1,2‐DCE	12	75	83:17	15:1
10	**7**	PhMe	48	58	85:15	8:1
11	**7**	CPME	48	trace	ND	ND
12^[f]^	**7**	CHCl_3_	12	96	96:4	20:1

[a] Reactions were carried out with 0.10 mmol of **1 a**, 0.11 mmol of **2 a** and Rh_2_(OAc)_4_ (5 mol %) in the presence of catalyst **PA** (10 mol %) in CHCl_3_ (3 mL). [b] Yield of isolated product of both diastereomers after chromatographic purification. [c] Decomposition accounts for remainder of mass balance. [d] Enantiomeric ratios (er) were determined by chiral HPLC. [e] Diastereomeric ratios (dr) were determined from ^1^H NMR of crude reaction mixture. [f] With 3 Å MS (35 mg).

Extensive screening of suitable chiral phosphoric acid catalysts^15^ revealed that Rh_2_(OAc)_4_ (5 mol %) and 10 mol % of (*R*)‐**PA7** provided the best combination, which afforded **3 a** in 79 % yield with 20:1‐diastereoselectivity and with 92:8 e.r. (entry 7). A short study of reaction conditions revealed CHCl_3_ to be the solvent of choice and that both chemical yield and enantioselectivity were further improved by using 3 Å molecular sieves (MS) as dehydrating agent. When using these conditions, **3 a** was eventually obtained in 96 % yield with 20:1 diastereoselectivity and with 96:4 e.r. (entry 12). Interestingly, lowering the catalyst loading of Rh_2_(OAc)_4_ and (*R*)‐**PA7** did not decrease the enantiomeric ratio, but led to a decrease in the diastereoselectivity of the product (see the Supporting Information for more details).

With optimized conditions in hand, we set out to examine the substrate scope of the reaction. Initially, a series of α‐diazoesters **2 a**–**k** was tested with benzhydryl alcohol **1 a** as a model *ortho*‐quinone methide precursor. Pleasingly, the reaction worked well with all substrates and afforded products **3 a**–**k** in good to excellent yields and with excellent enantioselectivity of up to 97:3 e.r. (Scheme 2). The diastereoselectivity appeared to be dependent on the electronic character of the aryl substituent, with electron‐rich aryl groups generally giving rise to almost perfect selectivity. In particular, the thiophene‐substituted diazoester **2 k** gave rise to product **3 k** in 92 % yield as a single diastereomer and with 95:5 e.r. On the other hand, substrates **2 g**–**j**, which carry electron‐poor substituents (such as halogen and CF_3_ groups) furnished products **3 g**–**j** with diminished diastereoselectivity, albeit in excellent yields and up to 96:4 e.r. *Ortho*‐substituted aryl groups had a detrimental effect on both the diastereo‐ and enantioselectivity, as shown for **3 e** (6:1 d.r., 89:11 e.r.), probably for steric reasons. Most importantly, this cycloannulation is not limited to aryl‐ and heteroaryl‐substituted diazoketones but could be extended to an alkyl‐substituted substrate as well, since dibenzooxacine **3 l** was obtained in high yield and stereoselectivity similar to the other products.

**Scheme 2:**
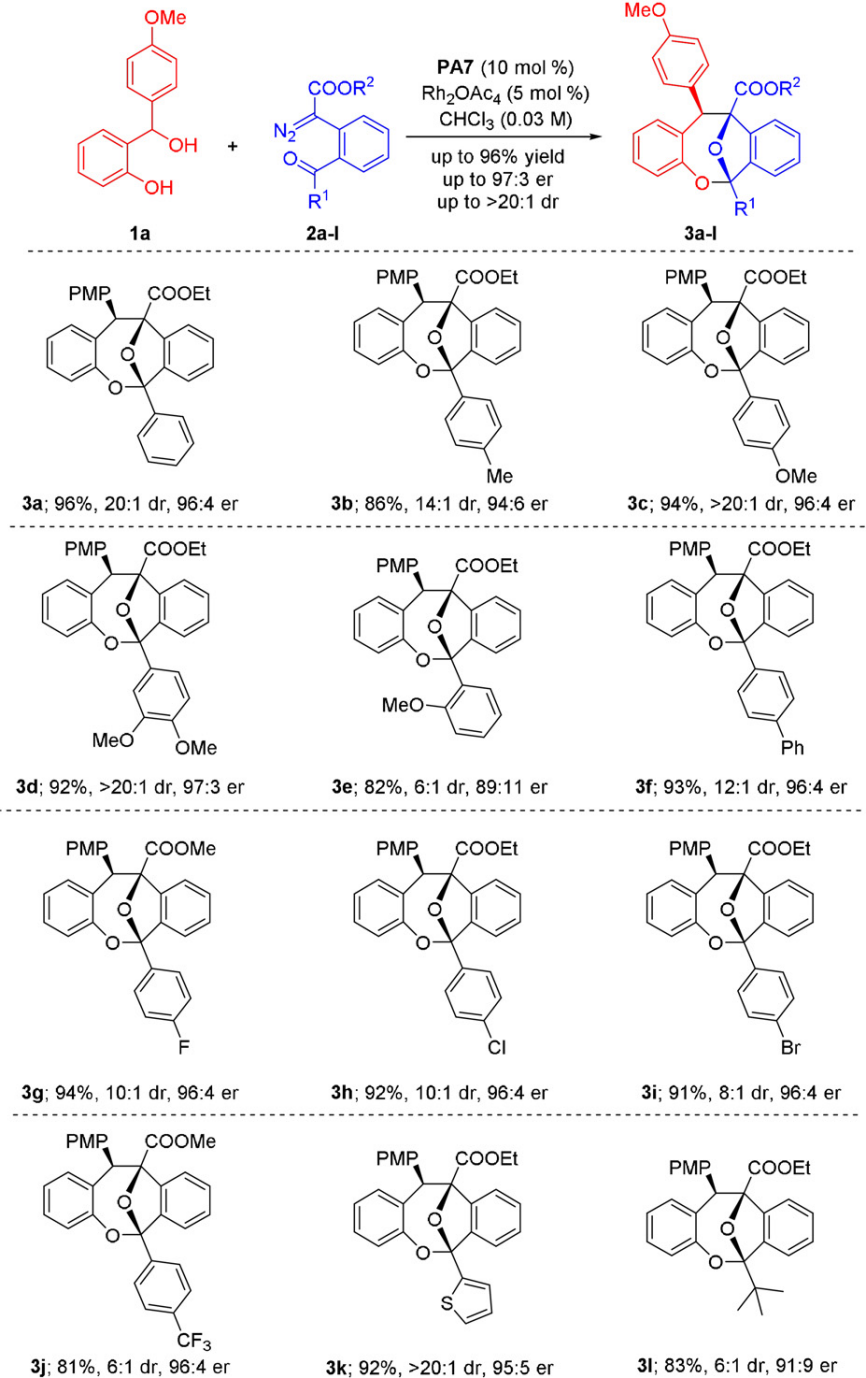
Substrate scope for reactions of *ortho*‐hydroxy benzhydryl alcohol **1 a** with various α‐diazoesters (**2 a**–**l**). Reactions were carried out with 0.1 mmol of **1 a**, 0.11 mmol of **2**, 3 Å MS (35 mg) and Rh_2_(OAc)_4_ (5 mol %) in the presence of catalyst **PA7** (10 mol %) in CHCl_3_ (3 mL).

We then turned our attention to reactions of α‐diazoester **2 a** with various substituted *o*‐hydroxy benzhydryl alcohols **1** as *ortho*‐quinone methide precursors (Scheme 3). Gratifyingly, a broad variety of substrates with both electron‐donating and electron‐withdrawing substituents in the *o*‐QM component were readily converted into products **4 a**–**n** at slightly elevated temperature. Yields ranged from moderate to excellent and the diastereo‐ and enantioselectivity were generally very high. Here again, a dependence of reaction outcome on the electronic character of the substrates was observed. Whereas electron‐rich benzhydryl alcohols furnished products with very good yields (e.g., **4 a**–**4 f**), electron‐poor substrates afforded products with only moderate chemical yield, albeit excellent enantioselectivity (e.g., **4 g** and **4 h**).

**Scheme 3:**
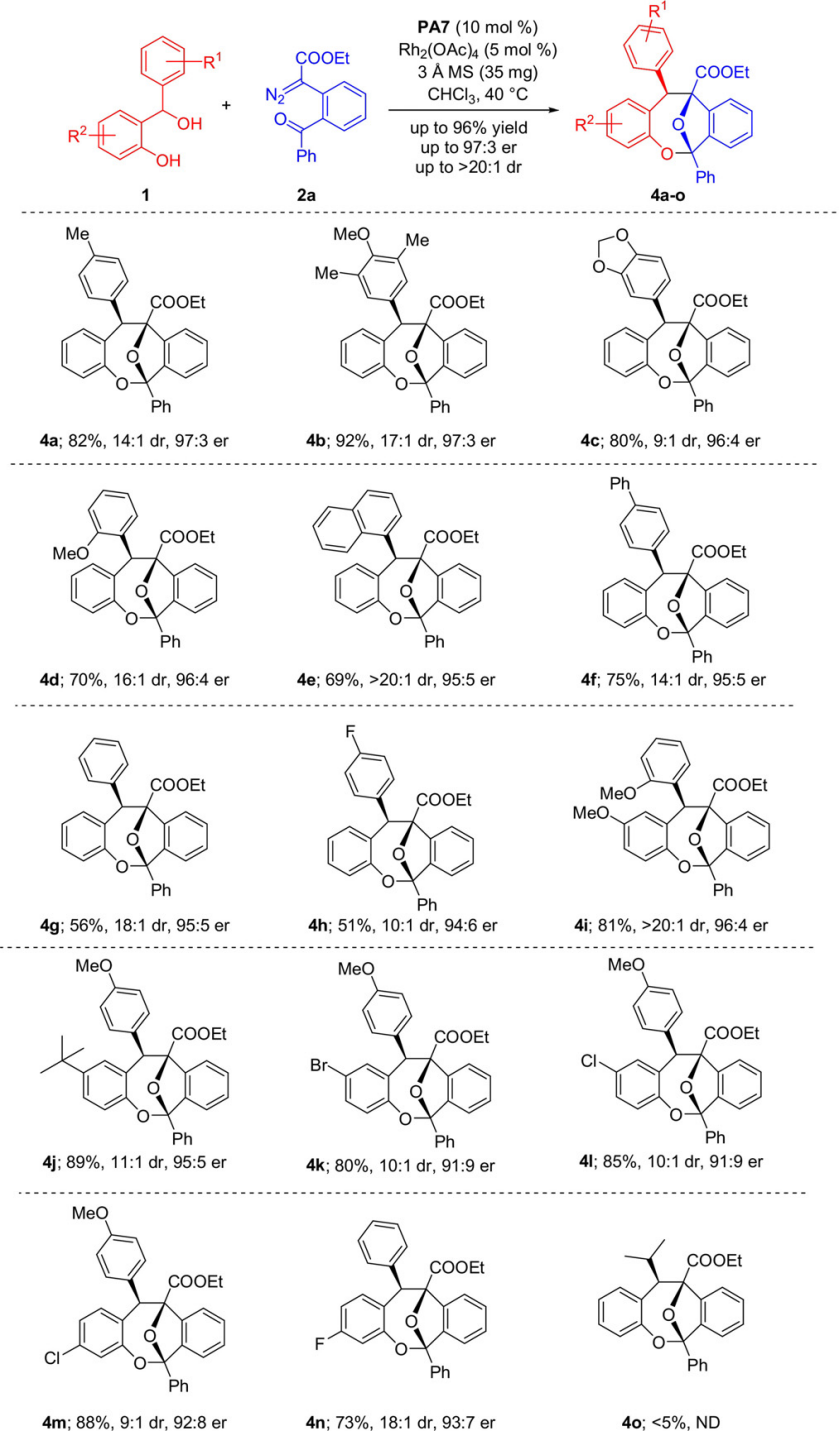
Expansion of substrate scope for the reaction of *ortho*‐hydroxy benzhydryl alcohols **1** with α‐diazoester **2 a**. Reactions were carried out with 0.1 mmol of **1**, 0.11 mmol of **2 a**, 3 Å MS (35 mg) and Rh_2_(OAc)_4_ (5 mol %) in the presence of catalyst **PA7** (10 mol %) in CHCl_3_ (3 mL).

Structural variation of the quinone moiety was more readily tolerated irrespective of the electronic character, and products **4 j**–**4 n** with alkyl and various halogen substituents were obtained with synthetically useful yields and very good diastereo‐ and enantioselectivity (Scheme 3). Unfortunately, the *i*Pr‐substituted benzhydryl alcohol **1 o** failed to deliver product **4 o** because the transient *o*‐QM generated in situ from **1 o** was apparently too unstable to successfully engage the transient carbonyl ylide in the cycloannulation event. The X‐ray structure analysis of the major diastereomer of product **3 k** confirmed both the relative and absolute configuration, which was assigned to all other products accordingly (Figure 1).^16^, ^17^

**Figure 1:**
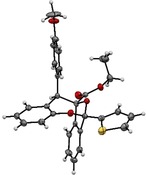
X‐ray crystal structure of product **3 k**.^16^

To gain more insight into the mechanism of this cycloannulation process, some control experiments were conducted. Under the standard conditions, O‐methyl‐protected benzhydryl alcohol **1 p** failed to react with **2 a**, thereby underlining the importance of the *o*‐QM structure for this reaction [Scheme 4, Eq. (1)]. Furthermore, neither in the presence of the phosphoric acid alone with Rh_2_(OAc)_4_ absent (case A) nor in the presence of Rh_2_(OAc)_4_ alone with the phosphoric acid absent (case B) was a successful reaction observed [Eq. (2)]. We therefore conclude that both catalysts actively participate in this reaction by generating both the *o*‐QM and the carbonyl ylide as transient intermediates. These control experiments strongly support our initial reaction design of a cooperative catalytic activation of both nucleophile and electrophile in a one‐pot process.

**Scheme 4:**
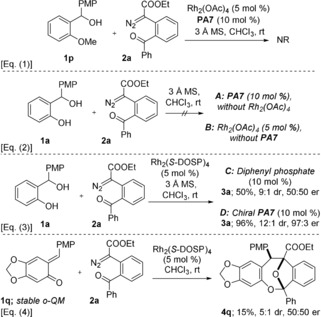
Control experiments.

In order to further shine light on the origin of the enantioselectivity, we conducted reactions of **1 a** and **2 a** in the presence of a chiral rhodium catalyst and both an achiral and a chiral phosphoric acid [Scheme 4, Eq. (3)]. Whereas the enantioselectivity of the latter reaction was virtually unchanged in comparison to the reaction with Rh_2_(OAc)_4_ reported above, no enantioselectivity was observed with diphenyl phosphate as a Brønsted acid catalyst. Moreover, reaction of the stable *ortho*‐quinone methide **1 q** and **2 a** in the presence of the chiral rhodium catalyst alone delivered dibenzooxacine **4 q** in low yield and as a racemic mixture, indicating once again the critical role of the chiral phosphoric acid for the enantioselectivity of the process [Eq. (4)].

Finally, we attempted some structural modifications of the products and identified the acetal moiety of **3 a** as a good starting point for further synthetic elaborations. Under BF_3_ activation, the acetal was readily cleaved to the corresponding oxonium ion, which was trapped with allyltributylstannane to furnish isobenzofuran **5** with good yield and complete diastereocontrol. Phenol **5** was then lactonized with *p*‐TsOH to produce the highly congested spirocyclic dihydrocoumarin **6**, again with good yield (Scheme 5). Product **6** was obtained in 72 % yield over two steps as a single diastereomer and with 98:2 e.r. On the other hand, the oxa‐bridged products **3** are sufficiently stable as to easily tolerate further post‐modifications such as a Suzuki–Miyaura cross coupling reaction, which proceeded in very good yield in the case of **3 i**.

**Scheme 5:**
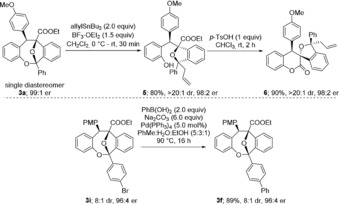
Synthetic elaborations of oxa‐bridged dibenzooxacines 3.

In conclusion, we have developed a novel and highly stereoselective [4+3]‐cycloannulation of transient carbonyl ylides with in situ generated *o*‐QMs through cooperative Rh/phosphoric acid catalysis. The reaction enables the catalytic enantio‐ and diastereoselective synthesis of oxa‐bridged heterocycles featuring two quaternary and one tertiary stereogenic centers in a one‐pot operation. The benzannulated O‐heterocycles were obtained in typically high yields (up to 96 %) and excellent stereoselectivity (up to >20:1 d.r. and up to 97:3 e.r.). Moreover, the products may be successfully manipulated to access valuable synthetic building blocks. The striking feature of this process is the separate catalytic activation of nucleophile and electrophile, with a chiral phosphoric acid enabling the formation of a transient hydrogen‐bonded *ortho*‐quinone methide and Rh_2_(OAc)_4_ delivering the transient carbonyl ylide in a one‐pot operation. Further extensions of this process are currently underway in our laboratory.

## Conflict of interest

The authors declare no conflict of interest.

## Supporting information

As a service to our authors and readers, this journal provides supporting information supplied by the authors. Such materials are peer reviewed and may be re‐organized for online delivery, but are not copy‐edited or typeset. Technical support issues arising from supporting information (other than missing files) should be addressed to the authors.

Supplementary

## References

[1a] Y. Li , M. Dai , Angew. Chem. Int. Ed. 2017, 56, 11624; 10.1002/anie.201706845 PMC5682107 28708291

[1b] R. C. Jadulco , C. D. Pond , R. M. Van Wagoner , M. Koch , O. G. Gideon , T. K. Matainaho , P. Piskaut , L. R. Barrows , J. Nat. Prod. 2014, 77, 183. 24392742 10.1021/np400847t PMC3931125

[2a] W. Zhao , Chem. Rev. 2010, 110, 1706; 19831366 10.1021/cr9002402

[2b] Z. Yin , Y. He , P. Chiu , Chem. Soc. Rev. 2018, 47, 8881. 30394457 10.1039/c8cs00532j

[3a] J. Zhang , Z. Liao , L. Chen , S. Zhu , Chem. Eur. J. 2019, 25, 9405; 30883965 10.1002/chem.201900807

[3b] J. Zhang , Z. Liao , L. Chen , H. Jiang , S. Zhu , Chem. Commun. 2019, 55, 7382. 10.1039/c9cc03715b 31173008

[4] Selected reviews:

[4a] M. H. Wang , K. A. Scheidt , Angew. Chem. Int. Ed. 2016, 55, 14912; 10.1002/anie.201605319 27763702

[4b] S. Afewerki , A. Córdova , Chem. Rev. 2016, 116, 13512; 27723291 10.1021/acs.chemrev.6b00226

[4c] Y. Deng , S. Kumar , H. Wang , Chem. Commun. 2014, 50, 4272; 10.1039/c4cc00072b 24637566

[4d] H. Pellissier , Tetrahedron 2013, 69, 7171;

[4e] X. Guo , W. Hu , Acc. Chem. Res. 2013, 46, 2427; 24246000 10.1021/ar300340k

[4f] Z. Du , Z. Shao , Chem. Soc. Rev. 2013, 42, 1337; 23154522 10.1039/c2cs35258c

[4g] A. E. Allen , D. W. C. MacMillan , Chem. Sci. 2012, 3, 633; 10.1039/C2SC00907B PMC3327486 22518271

[4h] R. C. Wende , P. R. Schreiner , Green Chem. 2012, 14, 1821.

[5a] W. H. Hu , X. F. Xu , J. Zhou , W. J. Liu , H. X. Huang , J. Hu , L. P. Yang , L. Z. Gong , J. Am. Chem. Soc. 2008, 130, 7782. 18512907 10.1021/ja801755z

[6] M. Terada , Y. Toda , Angew. Chem. Int. Ed. 2012, 51, 2093; 10.1002/anie.201107805 22262558

[7a] M. Petzold , P. J. Jones , D. B. Werz , Angew. Chem. Int. Ed. 2019, 58, 6225; 10.1002/anie.201814409 30758111

[7b] Q. Q. Cheng , M. Lankelma , D. Wherritt , H. Arman , M. P. Doyle , J. Am. Chem. Soc. 2017, 139, 9839; 28696679 10.1021/jacs.7b05840

[7c] A. G. Smith , H. M. L. Davies , J. Am. Chem. Soc. 2012, 134, 18241; 23098215 10.1021/ja3092399 PMC3549400

[7d] X. Hong , S. France , A. Padwa , Tetrahedron 2007, 63, 5962; 17710185 10.1016/j.tet.2007.01.064 PMC1948834

[7e] M. P. Doyle , M. A. McKervey , T. Ye , Modern Catalytic Methods for Organic Synthesis with Diazo Compounds, Wiley, New York, 1998.

[8a] S. A. Bonderoff , A. Padwa , J. Org. Chem. 2017, 82, 642; 27976898 10.1021/acs.joc.6b02663

[8b] H. Li , S. A. Bonderoff , B. Cheng , A. Padwa , J. Org. Chem. 2014, 79, 392; 24328106 10.1021/jo4026622

[8c] M. P. Doyle , R. J. Pieters , J. Taunton , H. Q. Pho , A. Padwa , D. L. Hertzog , L. Precedo , J. Org. Chem. 1991, 56, 820.

[9] Selected reviews on [4+3]-cycloadditions:

[9a] A. G. Lohse , R. P. Hsung , Chem. Eur. J. 2011, 17, 3812; 21384451 10.1002/chem.201100260 PMC3098035

[9b] M. Harmata , Chem. Commun. 2010, 46, 8904. 10.1039/c0cc03621h 21063614

[10] Selected recent examples of [4+3]-cycloadditions:

[10a] H. Xu , J.-L. Hu , L. Wang , S. Liao , Y. Tang , J. Am. Chem. Soc. 2015, 137, 8006; 26068395 10.1021/jacs.5b04429

[10b] H. Shang , Y. Wang , Y. Tian , J. Feng , Y. Tang , Angew. Chem. Int. Ed. 2014, 53, 5662; 10.1002/anie.201400426 24729335

[10c] B. D. Schwartz , J. R. Denton , Y. Lian , H. M. L. Davies , C. M. Williams , J. Am. Chem. Soc. 2009, 131, 8329; 19445507 10.1021/ja9019484 PMC2717789

[10d] R. P. Reddy , H. M. L. Davies , J. Am. Chem. Soc. 2007, 129, 10312. 17685525 10.1021/ja072936e

[11] Representative reviews on *o*-OMs:

[11a] C. D. T. Nielsen , H. Abas , A. C. Spivey , Synthesis 2018, 50, 4008;

[11b] B. Yang , S. Gao , Chem. Soc. Rev. 2018, 47, 7926; 29993045 10.1039/c8cs00274f

[11c] A. A. Jaworski , K. A. Scheidt , J. Org. Chem. 2016, 81, 10145; 27513764 10.1021/acs.joc.6b01367

[11d] L. Caruana , M. Fochi , L. Bernardi , Molecules 2015, 20, 11733; 26121398 10.3390/molecules200711733 PMC6331896

[11e] Z. Wang , J. Sun , Synthesis 2015, 47, 3629;

[11f] W.-J. Bai , J. G. David , Z.-G. Feng , M. G. Weaver , K.-L. Wu , T. R. R. Pettus , Acc. Chem. Res. 2014, 47, 3655; 25469551 10.1021/ar500330x PMC4270411

[11g] N. J. Willis , C. D. Bray , Chem. Eur. J. 2012, 18, 9160; 22707392 10.1002/chem.201200619

[11h] T. P. Pathak , M. S. Sigman , J. Org. Chem. 2011, 76, 9210. 21999240 10.1021/jo201789k PMC3215806

[12] From our group:

[12a] R. Ukis , C. Schneider , J. Org. Chem. 2019, 84, 7175; 31117571 10.1021/acs.joc.9b00860

[12b] A. Suneja , C. Schneider , Org. Lett. 2018, 20, 7576; 30407018 10.1021/acs.orglett.8b03311

[12c] F. Göricke , C. Schneider , Angew. Chem. Int. Ed. 2018, 57, 14736; 10.1002/anie.201809692 30278112

[12d] M. Spanka , C. Schneider , Org. Lett. 2018, 20, 4769; 30074397 10.1021/acs.orglett.8b01865

[12e] K. Gebauer , F. Reuß , M. Spanka , C. Schneider , Org. Lett. 2017, 19, 4588; 28809505 10.1021/acs.orglett.7b02185

[12f] S. K. Alamsetti , M. Spanka , C. Schneider , Angew. Chem. Int. Ed. 2016, 55, 2392; 10.1002/anie.201509247 26762542

[12g] S. Saha , S. K. Alamsetti , C. Schneider , Chem. Commun. 2015, 51, 1461; 10.1039/c4cc08559k 25493449

[12h] S. Saha , C. Schneider , Org. Lett. 2015, 17, 648; 25611975 10.1021/ol503662g

[12i] S. Saha , C. Schneider , Chem. Eur. J. 2015, 21, 2348; 25488376 10.1002/chem.201406044

[12j] O. El-Sepelgy , S. Haseloff , S. K. Alamsetti , C. Schneider , Angew. Chem. Int. Ed. 2014, 53, 7923; 10.1002/anie.201403573 24938645

[13] From other groups:

[13a] F. Jiang , K.-W. Chen , P. Wu , Y.-C. Zhang , Y. Jiao , F. Shi , Angew. Chem. Int. Ed. 2019, 58, 15104; 10.1002/anie.201908279 31441203

[13b] R. P. Pandit , S. T. Kim , D. H. Ryu , Angew. Chem. Int. Ed. 2019, 58, 13427; 10.1002/anie.201906954 31309680

[13c] C. D. Gheewala , J. S. Hirschi , W.-H. Lee , D. W. Paley , M. J. Vetticatt , T. H. Lambert , J. Am. Chem. Soc. 2018, 140, 3523; 29485273 10.1021/jacs.8b00260 PMC5859540

[13d] H. J. Jeong , D. Y. Kim , Org. Lett. 2018, 20, 2944; 29715043 10.1021/acs.orglett.8b00993

[13e] Y. Xie , B. List , Angew. Chem. Int. Ed. 2017, 56, 4936; 10.1002/anie.201612149 28211227

[13f] J.-L. Wu , J.-Y. Wang , P. Wu , G.-J. Mei , F. Shi , Org. Chem. Front. 2017, 4, 2465;

[13g] Z. Wang , J. Sun , Org. Lett. 2017, 19, 2334; 28445070 10.1021/acs.orglett.7b00867

[13h] C. Gharui , S. Singh , S. C. Pan , Org. Biomol. Chem. 2017, 15, 7272; 28858366 10.1039/c7ob01766a

[13i] M.-M. Xu , H.-Q. Wang , Y. Wan , G. He , J. Yan , S. Zhang , S.-L. Wang , F. Shi , Org. Chem. Front. 2017, 4, 358;

[13j] X.-Y. Yu , J.-R. Chen , Q. Wei , H.-G. Cheng , Z.-C. Liu , W.-J. Xiao , Chem. Eur. J. 2016, 22, 6774; 26990670 10.1002/chem.201601227

[13k] Y.-C. Zhang , Q.-N. Zhu , X. Yang , L.-J. Zhou , F. Shi , J. Org. Chem. 2016, 81, 1681; 26800116 10.1021/acs.joc.6b00078

[13l] Z. Lai , J. Sun , Synlett 2016, 27, 555;

[13m] J.-J. Zhao , Y.-C. Zhang , M.-M. Xu , M. Tang , F. Shi , J. Org. Chem. 2015, 80, 10016; 26387550 10.1021/acs.joc.5b01613

[13n] G. C. Tsui , L. Liu , B. List , Angew. Chem. Int. Ed. 2015, 54, 7703; 10.1002/anie.201500219 26015083

[13o] H. Hu , Y. Liu , J. Guo , L. Lin , Y. Xu , X. Liu , X. Feng , Chem. Commun. 2015, 51, 3835; 10.1039/c4cc10343b 25649623

[13p] W. Zhao , Z. Wang , B. Chu , J. Sun , Angew. Chem. Int. Ed. 2015, 54, 1910; 10.1002/anie.201405252 25088146

[13q] J.-J. Zhao , S.-B. Sun , S.-H. He , Q. Wu , F. Shi , Angew. Chem. Int. Ed. 2015, 54, 5460; 10.1002/anie.201500215 25693691

[13r] Z. Wang , F. Ai , Z. Wang , W. Zhao , G. Zhu , Z. Lin , J. Sun , J. Am. Chem. Soc. 2015, 137, 383; 25482291 10.1021/ja510980d

[13s] C.-C. Hsiao , S. Raja , H.-H. Liao , I. Atodiresei , M. Rueping , Angew. Chem. Int. Ed. 2015, 54, 5762; 10.1002/anie.201409850 25784558

[13t] Z. Lai , Z. Wang , J. Sun , Org. Lett. 2015, 17, 6058; 26637015 10.1021/acs.orglett.5b03072

[13u] C.-C. Hsiao , H.-H. Liao , M. Rueping , Angew. Chem. Int. Ed. 2014, 53, 13258; 10.1002/anie.201406587 25287936

[13v] M. Rueping , U. Uria , M. Y. Lin , I. Atodiresei , J. Am. Chem. Soc. 2011, 133, 3732; 21355548 10.1021/ja110213t

[13w] D. Wilcke , E. Herdtweck , T. Bach , Synlett 2011, 1235.

[14] Recent enantioselective [4+3]-cycloannulations of *o*-QMs: with chiral NHC catalysis:

[14a] J. Izquierdo , A. Orue , K. A. Scheidt , J. Am. Chem. Soc. 2013, 135, 10634; 23829462 10.1021/ja405833m PMC3746556

[14b] H. Lv , W.-Q. Jia , L.-H. Sun , S. Ye , Angew. Chem. Int. Ed. 2013, 52, 8607; 10.1002/anie.201303903 23818406

[14c] M. Sun , C. Ma , S.-J. Zhou , S.-F. Lou , J. Xiao , Y. Jiao , F. Shi , Angew. Chem. Int. Ed. 2019, 58, 8703; 10.1002/anie.201901955 30977568

[14d] J.-Y. Du , Y.-H. Ma , F.-X. Meng , R.-R. Zhang , R.-N. Wang , H.-L. Shi , Q. Wang , Y.-X. Fan , H.-L. Huang , J.-C. Cui , C.-L. Ma , Org. Lett. 2019, 21, 465; 30618260 10.1021/acs.orglett.8b03709

[14e] H. Lam , Z. Qureshi , M. Wegmann , M. Lautens , Angew. Chem. Int. Ed. 2018, 57, 16185; 10.1002/anie.201810760 30338629

[14f] a related process not involving *o*-QMs that appeared during the revision of this manuscript see: C. R. Xu , K. X. Wang , D. W. Li , L. L. Lin , X. M. Feng , Angew. Chem. Int. Ed. 2019, 58, 18438;

[14g] G.-J. Mei , Z.-Q. Zhu , J.-J. Zhao , C.-Y. Bian , J. Chen , R.-W. Chen , F. Shi , Chem. Commun. 2017, 53, 2768; 10.1039/c6cc09775h 28217784

[14h] J. Xu , S. Yuan , J. Peng , M. Miao , Z. Chen , H. Ren , Org. Biomol. Chem. 2017, 15, 7513. 28869260 10.1039/c7ob01783a

[15] Selected reviews:

[15a] F. E. Heldt , D. Grau , S. B. Tsogoeva , Molecules 2015, 20, 16103; 26404222 10.3390/molecules200916103 PMC6332096

[15b] D. Parmar , E. Sugiono , S. Raja , M. Rueping , Chem. Rev. 2014, 114, 9047; 25203602 10.1021/cr5001496

[15c] A. A. Desai , W. D. Wulff , Synthesis 2010, 3670;

[15d] D. Kampen , C. M. Reisinger , B. List , Top. Curr. Chem. 2010, 291, 395; 21494945 10.1007/978-3-642-02815-1_1

[15e] A. Zamfir , S. Schenker , M. Freund , S. B. Tsogoeva , Org. Biomol. Chem. 2010, 8, 5262; 20820680 10.1039/c0ob00209g

[15f] M. Terada , Synthesis 2010, 12, 1929;

[15g] T. Akiyama , Chem. Rev. 2007, 107, 5744. 17983247 10.1021/cr068374j

[16] CCDC https://www.ccdc.cam.ac.uk/services/structures?id=doi:10.1002/anie.201913603 (**3 k**) contains the supplementary crystallographic data for this paper. These data are provided free of charge by http://www.ccdc.cam.ac.uk/.

[17] We assume a similar transition-state assembly in this reaction as in previous examples of phosphoric acid catalyzed reactions of *ortho*-quinone methides (see Ref. [12j] for example) based upon the identical absolute configuration of the products.

