# Supplementary material for: Cooperative Catalysis for the Highly Diastereo‐ and Enantioselective [4+3]‐Cycloannulation of ortho‐Quinone Methides and Carbonyl Ylides
Source: Angew Chem Int Ed Engl. 2020 Jan 23;59(14):5536–40. doi: 10.1002/anie.201913603 (PMC7155103; doi:10.1002/anie.201913603)
Supplement: Supplementary file 1 — Supplementary [file ANIE-59-5536-s001.pdf]

Angewandte Chemie
Eine Zeitschrift der Gesellschaft Deutscher Chemiker

Supporting Information

Cooperative Catalysis for the Highly Diastereo- and Enantioselective [4+3]-Cycloannulation of *ortho*-Quinone Methides and Carbonyl Ylides

*Arun Suneja, Henning Jakob Loui, and Christoph Schneider**

anie_201913603_sm_miscellaneous_information.pdf

Table of Contents

Materials and methods	S2
General procedure for the synthesis of compounds **2a-l**	S3 – S4
Spectral analysis of compounds **2a-l**	S4 – S11
Optimization Studies	S12
General procedure for [4+3]-cycloannulation reaction	S13
Spectral analysis of compounds **3a-l**	S13 – S20
Spectral analysis of compounds **4a-q**	S20 – S28
Procedure for the synthesis of compound **5**	S28
Spectral analysis of compound **5**	S29
Procedure for the synthesis of compound **6**	S29
Spectral analysis of compound **6**	S29
Procedure for the synthesis of compound **3f**	S30
X-ray crystal structure of compound **3k**	S30
References	S30
Spectral graphics (^{1}H, and ^{13}C) NMR	S31 – S82
HPLC traces	S83 – S111
Crystallographic Data	S112 – S122

Materials and Methods

Unless otherwise stated, all reactions were performed in oven-dried glassware fitted with rubber septa under an inert atmosphere and were stirred with Teflon-coated magnetic stirring bars. Liquid reagents and solvents were transferred *via* syringe using standard Schlenk techniques. Tetrahydrofuran (THF), toluene and diethyl ether (Et_2O) were distilled over sodium/benzophenone ketyl. Dichloromethane (CH_2Cl_2), and $CHCl_3$ were distilled over calcium hydride. All other solvents and reagents were used as received unless otherwise noted. Reaction temperatures above 23 °C refer to oil bath temperature. Thin layer chromatography was performed using silica gel 60 F-254 pre-coated plates (0.25 mm) and visualized by UV irradiation and Vanillin stain solution. Silica gel of particle size 230-400 mesh was used for flash chromatography. ^{1}H, and ^{13}C NMR spectra were recorded using 300, and 400 MHz spectrometers. Chemical shifts (δ) are reported in ppm relative to the residual solvent ($CDCl_3$) signal (δ = 7.28 ppm for ^{1}H NMR and δ = 77.0 ppm for ^{13}C NMR). Data for ^{1}H NMR spectra are reported as follows: chemical shift (multiplicity, coupling constants, and number of hydrogen). Abbreviations are as follows: s (singlet), d (doublet), t (triplet), m (multiplet), br (broad). IR spectra were recorded on a FT-IR system and reported in frequency of absorption (cm^{-1}). High-Resolution Mass Spectrometry (HRMS) data was recorded on TOF-Q-II mass spectrometer. Optical rotations were measured on a commercial automatic polarimeter. Enantiomeric ratio was determined by chiral HPLC analysis with Daicel Chiralpak IA, and IE columns.

ortho-Hydroxy benzhydryl alcohols **1a-r** were prepared according to the literature known procedure.[1]

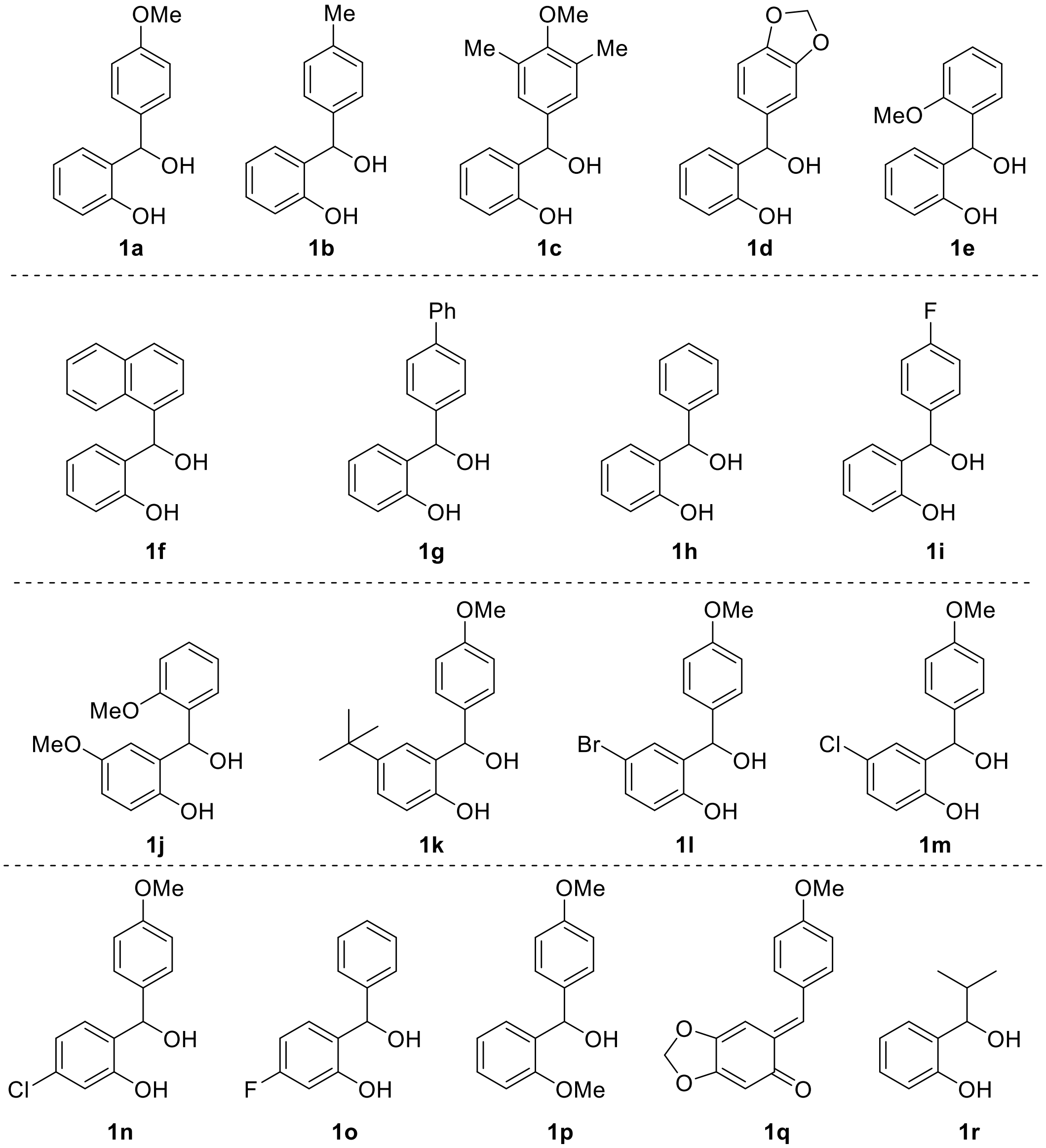

Figure 1. Structures of differently substituted *o*-hydroxy benzhydryl alcohols **1a-r**.

General procedure for the synthesis of *α*-diazoesters 2a-l:[2]

Step 1:

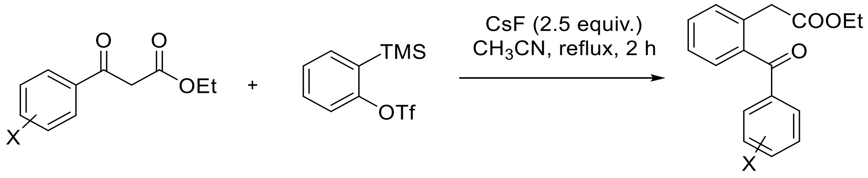

2a'-l'

To a solution of benzoylacetates (1.0 equiv.) and 2-(trimethylsilyl)phenyl triflate (1.3 equiv.) in CH_3CN (4 mL/mmol) under Ar was added CsF (2.5 equiv.). The reaction mixture was refluxed for 2 h. After completion of the reaction (monitored by TLC), the reaction mixture was cooled to RT and quenched by a aqueous solution of saturated NaCl (15 mL). The aqueous layer was extracted twice with EtOAc (15 mL each times). The combined organic layers were dried over Na_2SO_4 and conc. *in vacuo* to obtain crude product. The residue was purified over silica gel column chromatography using EtOAc/hexanes as eluent.

Step 2:

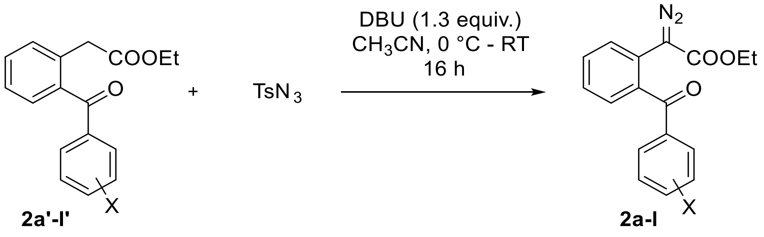

To a solution of acyl-alkyl derivative **2a'-l'** (1.0 equiv.) and TsN_3 (1.2 equiv.) in CH_3CN (3 mL/mmol) under Ar at 0 °C was slowly added DBU (1.3 equiv.). The reaction mixture was then stirred for 16 h at RT. Upon completion of the reaction (monitored by TLC), the mixture was concentrated under reduced pressure to remove excess of CH_3CN. The residue was then purified by flash chromatography (using EtOAc/hexanes as eluent) to afford the desired diazoesters **2a-l** in high yields.

Spectral Analysis:

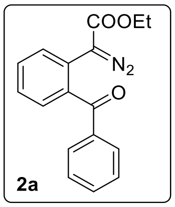

Ethyl 2-(2-benzoylphenyl)-2-diazoacetate (2a): Orange liquid, 1.05 g, 90% yield; **^{1}H NMR** (400 MHz, $CDCl_3$) δ 7.81 – 7.83 (m, 2H), 7.57 – 7.62 (m, 2H), 7.40 – 7.55 (m, 5H), 4.12 (q, *J* = 7.1 Hz, 2H), 1.15 (t, *J* = 7.1 Hz, 3H); **^{13}C NMR** (100 MHz, $CDCl_3$) δ 196.5, 165.2, 137.6, 137.1, 133.1, 131.1, 130.2, 129.9, 129.6, 128.3, 127.5, 125.1, 61.2, 14.3; **IR** (film) υ_{max} 3062, 2981, 2935, 2088, 1698, 1666, 1595, 1448, 1370, 1294, 1253, 1176, 1032, 926, 763 cm^{-1}; **HRMS** (ESI) m/z 317.0911 $[M + Na]^+$; calculated for $[C_{17}H_{14}N_2O_3 + Na]^+$: 317.0897.

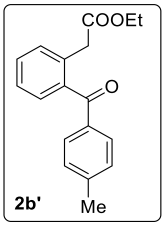

Ethyl 2-(2-(4-methylbenzoyl)phenyl)acetate (2b'): Light yellow liquid; 721 mg, 62% yield; **^{1}H NMR** (400 MHz, $CDCl_3$) δ 7.75 (d, *J* = 8.2 Hz, 2H), 7.49 (td, *J* = 7.4, 1.7 Hz, 1H), 7.32 – 7.41 (m, 3H), 7.26 – 7.28 (m, 2H), 4.04 (q, *J* = 7.1 Hz, 2H), 3.88 (s, 2H), 2.45 (s, 3H), 1.14 (t, *J* = 7.1 Hz, 3H); **^{13}C NMR** (100 MHz, $CDCl_3$) δ 197.6, 171.2, 143.7, 138.6, 135.1, 133.7, 131.6, 130.5, 130.5, 129.6, 128.9, 126.4, 60.7, 38.8, 21.6, 14.0; **IR** (film) υ_{max} 3062, 2981, 2905, 1735, 1659, 1605, 1573, 1446, 1407, 1368, 1271, 1214, 1154, 1030, 919, 780 cm^{-1}; **HRMS** (ESI) m/z 305.1152 [M + Na]$^+$; calculated for $[C_{18}H_{18}O_3 + Na]^+$: 305.1148.

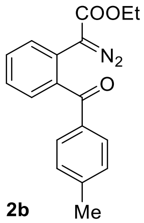

2b

Ethyl 2-diazo-2-(2-(4-methylbenzoyl)phenyl)acetate (2b): Orange liquid; 538 mg, 90% yield; **^{1}H NMR** (400 MHz, $CDCl_3$) δ 7.73 (d, *J* = 8.2 Hz, 2H), 7.50 – 7.57 (m, 3H), 7.41 (td, *J* = 7.4, 1.7 Hz, 1H), 7.25 – 7.28 (m, 2H), 4.12 (q, *J* = 7.0 Hz, 2H), 2.44 (s, 3H), 1.16 (t, *J* = 7.1 Hz, 3H); **^{13}C NMR** (100 MHz, $CDCl_3$) δ 196.2, 165.2, 144.0, 137.8, 134.4, 130.8, 130.1, 130.0, 129.6, 129.0, 127.5, 124.9, 61.1, 21.7, 14.3; **IR** (film) υ_{max} 3061, 2981, 2929, 2088, 1696, 1661, 1605, 1484, 1370, 1295, 1252, 1175, 1032, 928, 760 cm^{-1}; **HRMS** (ESI) m/z 331.1059 [M + Na]$^+$; calculated for $[C_{18}H_{16}N_2O_3 + Na]^+$: 331.1053.

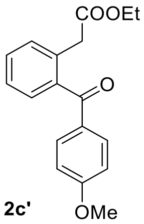

2c'

Ethyl 2-(2-(4-methoxybenzoyl)phenyl)acetate (2c'): Light yellow liquid; 864 mg, 57% yield; **^{1}H NMR** (400 MHz, $CDCl_3$) δ 7.84 (d, *J* = 8.9 Hz, 2H), 7.47 (td, *J* = 7.4, 1.7 Hz, 1H), 7.32 – 7.40 (m, 3H), 6.95 (d, *J* = 8.9 Hz, 2H), 4.03 (q, *J* = 7.1 Hz, 2H), 3.90 (s, 3H), 3.86 (s, 2H), 1.13 (t, *J* = 7.1 Hz, 3H); **^{13}C NMR** (100 MHz, $CDCl_3$) δ 196.6, 171.2, 163.5, 138.9, 133.5, 132.7, 131.5, 130.5, 130.3, 129.3, 126.4, 113.5, 60.7, 55.5, 38.7, 14.0; **IR** (film) υ_{max} 3065, 2980, 2904, 2840, 1733, 1654, 1599, 1574, 1509, 1419, 1368, 1290, 1258, 1176, 1151, 1028, 920, 748 cm^{-1}; **HRMS** (ESI) m/z 321.1107 [M + Na]$^+$; calculated for $[C_{18}H_{18}O_4 + Na]^+$: 321.1097.

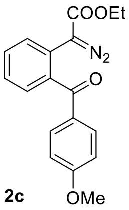

2c

Ethyl 2-diazo-2-(2-(4-methoxybenzoyl)phenyl)acetate (2c): Orange liquid; 557 mg, 90% yield; **^{1}H NMR** (400 MHz, $CDCl_3$) δ 7.82 (d, *J* = 8.8 Hz, 2H), 7.49 – 7.59 (m, 3H), 7.38 – 7.43 (m, 1H), 6.95 (d, *J* = 8.9 Hz, 2H), 4.13 (q, *J* = 7.1 Hz, 2H), 3.89 (s, 3H), 1.17 (t, *J* = 7.1 Hz, 3H); **^{13}C NMR** (100 MHz, $CDCl_3$) δ 195.2, 165.3, 163.6, 138.0, 132.3, 130.7, 129.8, 129.7, 127.5, 124.7, 113.6, 61.1, 55.5, 14.3; **IR** (film) υ_{max} 3063, 2980, 2840, 2088, 1698, 1655, 1599, 1509,

1462, 1370, 1294, 1258, 1152, 1030, 929, 761 cm^{-1}; **HRMS** (ESI) m/z 347.1017 [M + Na]$^+$; calculated for [$C_{18}H_{16}N_2O_4$ + Na]$^+$: 347.1002.

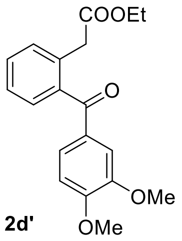

2d'

Ethyl 2-(2-(3,4-dimethoxybenzoyl)phenyl)acetate (2d'): Light yellow liquid, 770 mg, 74% yield; **^{1}H NMR** (400 MHz, $CDCl_3$) δ 7.54 (t, *J* = 1.7 Hz, 1H), 7.47 (t, *J* = 7.3 Hz, 1H), 7.31 – 7.40 (m, 4H), 6.87 (d, *J* = 8.3 Hz, 1H), 4.03 (qd, *J* = 7.2, 1.6 Hz, 2H), 3.95 (s, 3H), 3.94 (s, 3H), 3.84 (s, 2H), 1.13 (t, *J* = 7.1 Hz, 3H); **^{13}C NMR** (100 MHz, $CDCl_3$) δ 196.6, 171.2, 153.3, 148.9, 138.8, 133.5, 131.5, 130.6, 130.3, 129.3, 126.3, 126.1, 111.6, 109.7, 60.7, 56.0, 56.0, 38.7, 14.0; **IR** (film) υ_{max} 3077, 2981, 2935, 2905, 2838, 1732, 1650, 1593, 1512, 1463, 1416, 1339, 1272, 1230, 1133, 1097, 1024, 985, 761 cm^{-1}; **HRMS** (ESI) m/z 351.1212 [M + Na]$^+$; calculated for [$C_{19}H_{20}O_5$ + Na]$^+$: 351.1203.

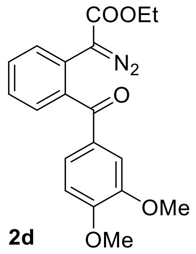

2d

Ethyl 2-diazo-2-(2-(3,4-dimethoxybenzoyl)phenyl)acetate (2d): Yellow solid, 660 mg, 84% yield; **^{1}H NMR** (400 MHz, $CDCl_3$) δ 7.49 – 7.59 (m, 4H), 7.38 – 7.43 (m, 1H), 7.34 (d, *J* = 8.4 Hz, 1H), 6.87 (d, *J* = 8.8 Hz, 1H), 4.13 (q, *J* = 7.0 Hz, 2H), 3.95 (s, 3H), 3.93 (s, 3H), 1.17 (t, *J* = 7.1 Hz, 3H); **^{13}C NMR** (100 MHz, $CDCl_3$) δ 195.3, 165.3, 153.5, 148.9, 137.9, 130.7, 129.8, 129.8, 127.5, 125.5, 124.7, 111.4, 109.8, 61.1, 56.0, 55.9, 14.3; **IR** (film) υ_{max} 3073, 2982, 2907, 2837, 2088, 1698, 1649, 1580, 1508, 1466, 1417, 1339, 1297, 1232, 1172, 1109, 1021, 886, 763 cm^{-1}; **HRMS** (ESI) m/z 377.1113 [M + Na]$^+$; calculated for [$C_{19}H_{18}N_2O_5$ + Na]$^+$: 377.1108.

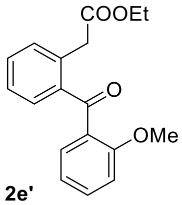

2e'

Ethyl 2-(2-(2-methoxybenzoyl)phenyl)acetate (2e'): Yellow liquid, 958 mg, 70% yield; **^{1}H NMR** (300 MHz, $CDCl_3$) δ 7.41 – 7.49 (m, 3H), 7.38 (dd, *J* = 7.8, 1.5 Hz, 1H), 7.29 – 7.33 (m, 1H), 7.23 – 7.28 (m, 1H), 6.94 – 7.02 (m, 2H), 4.13 (q, *J* = 7.1 Hz, 2H), 3.97 (s, 2H), 3.70 (s, 3H), 1.23 (t, *J* = 7.1 Hz, 3H); **^{13}C NMR** (100 MHz, $CDCl_3$) δ 197.7, 171.4, 158.0, 138.6, 134.4, 132.5, 131.9, 131.3, 131.2, 130.8, 129.1, 126.6, 120.2, 111.6, 60.6, 55.6, 39.6, 14.1; **IR** (film) υ_{max} 3070, 2981, 2904, 2837, 1731, 1666, 1597, 1577, 1486, 1435, 1368, 1303, 1243, 1212, 1160, 1024, 921, 756 cm^{-1}; **HRMS** (ESI) m/z 321.1106 [M + Na]$^+$; calculated for [$C_{18}H_{18}O_4$ + Na]$^+$: 321.1097.

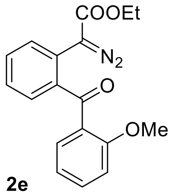

2e

Ethyl 2-diazo-2-(2-(2-methoxybenzoyl)phenyl)acetate (2e): Yellow solid, 888 mg, 87% yield; **^{1}H NMR** (400 MHz, $CDCl_3$) δ 7.46 – 7.59 (m, 5H), 7.34 – 7.39 (m, 1H), 7.04 (t, *J* = 7.5 Hz, 1H), 6.94 (d, *J* = 8.4 Hz, 1H), 4.17 (q, *J* = 7.1 Hz, 2H), 3.63 (s, 3H), 1.22 (t, *J* = 7.1 Hz, 3H); **^{13}C NMR** (100 MHz, $CDCl_3$) δ 196.0, 165.4, 158.2, 139.1, 133.3, 131.1, 130.9, 130.2, 130.0, 128.2, 127.7, 124.7, 120.5, 111.3, 61.9, 61.0, 55.5, 14.4; **IR** (film) $υ_{max}$ 2986, 2910, 2841, 2089, 1691, 1644, 1596, 1483, 1434, 1371, 1302, 1237, 1176, 1097, 1017, 931, 761 cm^{-1}; **HRMS** (ESI) m/z 347.1008 $[M + Na]^+$; calculated for $[C_{18}H_{16}N_2O_4 + Na]^+$: 347.1002.

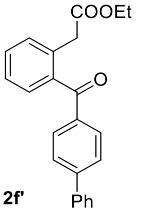

2f'

Ethyl 2-(2-([1,1'-biphenyl]-4-carbonyl)phenyl)acetate (2f'): Off-white solid, 716 mg, 65% yield; **^{1}H NMR** (400 MHz, $CDCl_3$) δ 7.88 – 7.91 (m, 2H), 7.66 – 7.69 (m, 2H), 7.62 – 7.65 (m, 2H), 7.43 – 7.51 (m, 4H), 7.33 – 7.42 (m, 3H), 4.04 (q, *J* = 7.1 Hz, 2H), 3.91 (s, 3H), 1.13 (t, *J* = 7.1 Hz, 3H); **^{13}C NMR** (100 MHz, $CDCl_3$) δ 197.5, 171.2, 145.6, 139.9, 138.4, 136.4, 133.9, 131.7, 130.9, 130.7, 129.8, 128.9, 128.2, 127.2, 126.9, 126.4, 60.8, 38.8, 14.0; **IR** (film) $υ_{max}$ 3069, 2984, 2907, 2872, 1729, 1652, 1599, 1482, 1447, 1406, 1338, 1275, 1213, 1173, 1030, 920, 739 cm^{-1}; **HRMS** (ESI) m/z 367.1310 $[M + Na]^+$; calculated for $[C_{23}H_{20}O_3 + Na]^+$: 367.1305.

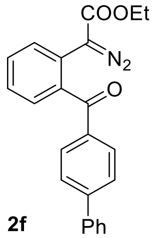

2f

Ethyl 2-(2-([1,1'-biphenyl]-4-carbonyl)phenyl)-2-diazoacetate (2f): Orange viscous liquid, 615 mg, 83% yield; **^{1}H NMR** (400 MHz, $CDCl_3$) δ 7.89 (d, *J* = 8.2 Hz, 2H), 7.68 (d, *J* = 8.0 Hz, 2H), 7.63 (d, *J* = 7.1 Hz, 2H), 7.51 – 7.59 (m, 3H), 7.45 – 7.49 (m, 2H), 7.38 – 7.43 (m, 2H), 4.11 (q, *J* = 7.1 Hz, 2H), 1.14 (t, *J* = 7.1 Hz, 3H); **^{13}C NMR** (100 MHz, $CDCl_3$) δ 196.1, 165.2, 145.7, 139.8, 137.6, 135.7, 131.0, 130.5, 130.1, 129.5, 128.9, 128.2, 127.5, 127.2, 126.9, 125.1, 61.1, 14.3; **IR** (film) $υ_{max}$ 3060, 2981, 2906, 2089, 1698, 1663, 1601, 1484, 1446, 1370, 1296, 1254, 1156, 1032, 929, 755 cm^{-1}; **HRMS** (ESI) m/z 393.1216 $[M + Na]^+$; calculated for $[C_{23}H_{18}N_2O_3 + Na]^+$: 393.1210.

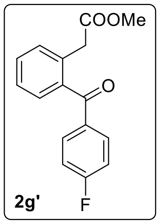

Methyl 2-(2-(4-fluorobenzoyl)phenyl)acetate (2g'): Pale yellow liquid, 800 mg, 64% yield; **^{1}H NMR** (400 MHz, $CDCl_3$) δ 7.82 – 7.87 (m, 2H), 7.45 – 7.49 (m, 1H), 7.31 – 7.38 (m, 3H), 7.09 – 7.15 (m, 2H), 3.89 (s, 2H), 3.55 (s, 3H); **^{13}C NMR** (100 MHz, $CDCl_3$) δ 196.4, 171.5, 165.6 (d, *J* = 253.4 Hz), 138.1, 134.0 (d, *J* = 2.8 Hz), 133.8, 133.0 (d, *J* = 9.2 Hz), 131.8, 130.9, 129.6, 126.5, 115.4 (d, *J* = 21.7 Hz), 51.8, 38.5; **IR** (film) υ_{max} 3071, 3022, 2952, 2844, 1736, 1663, 1597, 1504, 1435, 1408, 1343, 1271, 1226, 1150, 1012, 919, 853, 756 cm^{-1}; **HRMS** (ESI) m/z 295.0751 $[M + Na]^+$; calculated for $[C_{16}H_{13}FO_3 + Na]^+$: 295.0741.

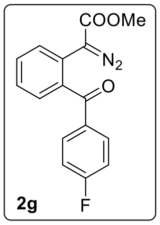

Methyl 2-diazo-2-(2-(4-fluorobenzoyl)phenyl)acetate (2g): Orange liquid; 769 mg, 92% yield; **^{1}H NMR** (400 MHz, $CDCl_3$) δ 7.86 (dd, *J* = 8.5, 5.4 Hz, 2H), 7.59 (t, *J* = 8.0 Hz, 1H), 7.48 – 7.52 (m, 2H), 7.42 (t, *J* = 7.4 Hz, 1H), 7.15 (t, *J* = 8.5 Hz, 2H), 3.64 (s, 3H); **^{13}C NMR** (100 MHz, $CDCl_3$) δ 195.0, 165.8 (d, *J* = 253.7 Hz), 165.5, 137.3, 133.4 (d, *J* = 2.7 Hz), 132.6 (d, *J* = 9.3 Hz), 131.1, 129.9, 129.5, 127.6, 124.9, 115.5 (d, J = 21.8 Hz), 52.0; **IR** (film) υ_{max} 3068, 2953, 2847, 2094, 1703, 1667, 1597, 1505, 1485, 1435, 1351, 1256, 1151, 1029, 931, 761 cm^{-1}; **HRMS** (ESI) m/z 321.0658 $[M + Na]^+$; calculated for $[C_{16}H_{11}FN_2O_3 + Na]^+$: 321.0646.

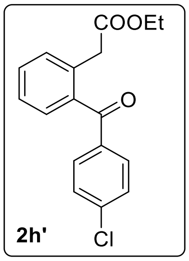

Ethyl 2-(2-(4-chlorobenzoyl)phenyl)acetate (2h'): Pale yellow liquid, 680 mg, 55% yield; **^{1}H NMR** (400 MHz, $CDCl_3$) δ 7.79 (d, *J* = 8.5 Hz, 2H), 7.44 – 7.52 (m, 3H), 7.34 – 7.39 (m, 3H), 4.05 (q, *J* = 7.1 Hz, 2H), 3.91 (s, 2H), 1.15 (t, *J* = 7.1 Hz, 3H); **^{13}C NMR** (100 MHz, $CDCl_3$) δ 196.7, 171.1, 139.3, 137.9, 136.1, 134.0, 131.8, 131.7, 131.0, 129.7, 128.5, 126.5, 60.8, 38.7, 14.0; **IR** (film) υ_{max} 3065, 2981, 2904, 1733, 1663, 1597, 1587, 1483, 1400, 1336, 1267, 1215, 1175, 1090, 1029, 919, 847, 735 cm^{-1}; **HRMS** (ESI) m/z 325.0606 $[M + Na]^+$; calculated for $[C_{17}H_{15}ClO_3 + Na]^+$: 325.0602.

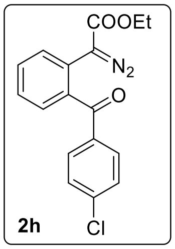

Ethyl 2-(2-(4-chlorobenzoyl)phenyl)-2-diazoacetate (2h): Orange liquid; 440 mg, 82% yield; **^{1}H NMR** (400 MHz, $CDCl_3$) δ 7.78 (d, *J* = 8.6 Hz, 2H), 7.59 (td, *J* = 7.6, 1.5 Hz, 1H), 7.48 – 7.52 (m, 2H), 7.39 – 7.46 (m, 3H), 4.12 (q, *J* = 7.1 Hz, 2H), 1.17 (t, *J* = 7.1 Hz, 3H); **^{13}C NMR** (100 MHz, $CDCl_3$) δ 195.2, 165.1, 139.5, 137.1, 135.4, 131.3, 131.2, 130.0, 129.4, 128.6, 127.5, 125.1, 61.2, 14.3; **IR** (film) υ_{max} 3064, 2982, 2906, 2088, 1698, 1661, 1587, 1483, 1444, 1399, 1300, 1253, 1176, 1090, 1032, 927, 763 cm^{-1}; **HRMS** (ESI) m/z 351.0527 $[M + Na]^+$; calculated for $[C_{17}H_{13}ClN_2O_3 + Na]^+$: 351.0507.

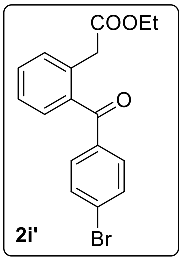

Ethyl 2-(2-(4-bromobenzoyl)phenyl)acetate (2i'): Light yellow liquid, 860 mg, 60% yield; **^{1}H NMR** (300 MHz, $CDCl_3$) δ 7.66 – 7.70 (m, 2H), 7.57 – 7.61 (m, 2H), 7.44 – 7.50 (m, 1H), 7.30 – 7.37 (m, 3H), 4.02 (q, *J* = 7.1 Hz, 2H), 3.89 (s, 2H), 1.13 (t, *J* = 7.1 Hz, 3H); **^{13}C NMR** (75 MHz, $CDCl_3$) δ 196.9, 171.1, 137.8, 136.5, 134.0, 131.8, 131.8, 131.5, 131.0, 129.7, 128.0, 126.5, 60.8, 38.7, 14.0; **IR** (film) υ_{max} 3063, 2981, 2903, 1733, 1664, 1585, 1480, 1395, 1368, 1267, 1214, 1175, 1068, 1011, 918, 845, 739 cm^{-1}; **HRMS** (ESI) m/z 369.0100 $[M + Na]^+$; calculated for $[C_{17}H_{15}{}^{79}BrO_3 + Na]^+$: 369.0097.

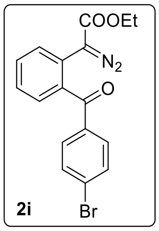

Ethyl 2-(2-(4-bromobenzoyl)phenyl)-2-diazoacetate (2i): Orange solid, 706 mg, 82% yield; **^{1}H NMR** (400 MHz, $CDCl_3$) δ 7.70 (d, *J* = 8.5 Hz, 2H), 7.57 – 7.62 (m, 3H), 7.48 – 7.52 (m, 2H), 7.42 (td, *J* = 7.5, 1.3 Hz, 1H), 4.13 (q, *J* = 7.1 Hz, 2H), 1.18 (t, *J* = 7.1 Hz, 3H); **^{13}C NMR** (100 MHz, $CDCl_3$) δ 195.4, 165.1, 137.0, 135.9, 131.6, 131.4, 131.3, 130.0, 129.4, 128.2, 127.5, 125.2, 61.2, 14.3; **IR** (film) υ_{max} 3063, 2986, 2905, 2087, 1695, 1668, 1585, 1481, 1396, 1339, 1298, 1241, 1167, 1099, 1036, 951, 762 cm^{-1}; **HRMS** (ESI) m/z 395.0011 $[M + Na]^+$; calculated for $[C_{17}H_{13}{}^{79}BrN_2O_3 + Na]^+$: 395.0002.

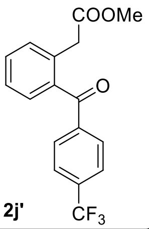

2j'

Methyl 2-(2-(4-(trifluoromethyl)benzoyl)phenyl)acetate (2j'): Pale yellow liquid, 800 mg, 47% yield; **^{1}H NMR** (400 MHz, $CDCl_3$) δ 7.95 (d, *J* = 8.0 Hz, 2H), 7.76 (d, *J* = 8.2 Hz, 2H), 7.51 – 7.55 (m, 1H), 7.35 – 7.41 (m, 3H), 3.98 (s, 2H), 3.60 (s, 3H); **^{13}C NMR** (100 MHz, $CDCl_3$) δ 196.9, 171.6, 140.8, 137.4, 134.3, 134.1 (q, *J* = 32.5 Hz) 132.0, 131.4, 130.5, 130.2, 126.7, 125.3 (q, *J* = 3.8 Hz), 123.6 (q, *J* = 271 Hz), 51.9, 38.6; **IR** (film) $υ_{max}$ 3067, 2954, 2845, 1739, 1670, 1576, 1509, 1436, 1408, 1325, 1269, 1169, 1065, 920, 859, 757 cm^{-1}; **HRMS** (ESI) m/z 345.0727 $[M + Na]^+$; calculated for $[C_{17}H_{13}F_3O_3 + Na]^+$: 345.0709.

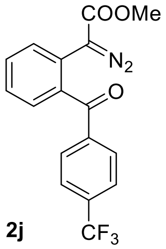

2j

Methyl 2-diazo-2-(2-(4-(trifluoromethyl)benzoyl)phenyl)acetate (2j): Yellow liquid, 520 mg, 65% yield; **^{1}H NMR** (400 MHz, $CDCl_3$) δ 7.95 (d, *J* = 8.0 Hz, 2H), 7.75 (d, *J* = 8.2 Hz, 2H), 7.62 (td, *J* = 7.6, 1.6 Hz, 1H), 7.50 (td, *J* = 7.7, 1.0 Hz, 2H), 7.43 (td, *J* = 7.5, 1.2 Hz, 1H), 3.64 (s, 3H); **^{13}C NMR** (100 MHz, $CDCl_3$) δ 195.3, 165.5, 140.1, 136.7, 134.2 (q, *J* = 32.4 Hz), 131.6, 130.2, 130.1, 129.2, 127.5, 125.3 (q, *J* = 3.7 Hz), 123.6 (q, *J* = 271 Hz), 61.9, 52.1; **IR** (film) $υ_{max}$ 3069, 2965, 2088, 1693, 1671, 1593, 1486, 1438, 1356, 1293, 1252, 1064, 1030, 931, 861, 758 cm^{-1}; **HRMS** (ESI) m/z 371.0607 $[M + Na]^+$; calculated for $[C_{17}H_{11}F_3N_2O_3 + Na]^+$: 371.0614.

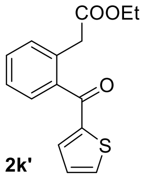

2k'

Ethyl 2-(2-(thiophene-2-carbonyl)phenyl)acetate (2k'): Yellow liquid, 980 mg, 66% yield; **^{1}H NMR** (400 MHz, $CDCl_3$) δ 7.74 (dd, *J* = 4.9, 1.2 Hz, 1H), 7.61 (dd, *J* = 7.9, 1.5 Hz, 1H), 7.48 – 7.53 (m, 2H), 7.37 – 7.40 (m, 2H), 7.15 (dd, *J* = 4.9, 3.8 Hz, 1H), 4.05 (q, *J* = 7.1 Hz, 2H), 3.90 (s, 2H), 1.13 (t, *J* = 7.1 Hz, 3H); **^{13}C NMR** (100 MHz, $CDCl_3$) δ 189.6, 171.1, 144.6, 138.2, 135.5, 134.7, 133.5, 131.7, 130.8, 129.2, 128.0, 126.5, 60.8, 38.7, 13.9; **IR** (film) $υ_{max}$ 3100, 2981, 2903, 1733, 1638, 1599, 1574, 1514, 1411, 1294, 1213, 1161, 1029, 849, 761 cm^{-1}; **HRMS** (ESI) m/z 297.0565 $[M + Na]^+$; calculated for $[C_{15}H_{14}O_3S + Na]^+$: 297.0556.

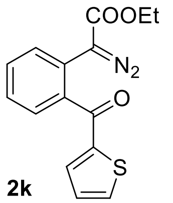

2k

Ethyl 2-diazo-2-(2-(thiophene-2-carbonyl)phenyl)acetate (2k): Yellow Solid; 798 mg, 83% yield; **^{1}H NMR** (400 MHz, $CDCl_3$) δ 7.70 – 7.72 (m, 1H), 7.60 – 7.62 (m, 1H), 7.51 – 7.57 (m, 2H), 7.48 – 7.50 (m, 1H), 7.37 – 7.41 (m, 1H), 7.10 – 7.13 (m, 1H), 4.13 (q, *J* = 7.1 Hz, 2H), 1.15 (t, *J* = 7.1 Hz, 3H); **^{13}C NMR** (100 MHz, $CDCl_3$) δ 188.4, 165.3, 143.9, 137.3, 135.0, 134.7, 131.0, 129.9, 129.6, 128.0, 127.4, 124.7, 61.2, 14.2; **IR** (film) $υ_{max}$ 3080, 2985, 2901, 2088, 1696, 1666, 1566, 1516, 1445, 1413, 1358, 1304, 1262, 1174, 1064, 1024, 847, 754 cm^{-1}; **HRMS** (ESI) m/z 323.0481 [M + Na]$^+$; calculated for $[C_{15}H_{12}N_2O_3S + Na]^+$: 323.0461.

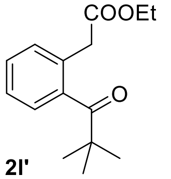

2l'

Ethyl 2-(2-pivaloylphenyl)acetate (2l'):[2b] Yellow liquid, 171 mg, 69% yield; **^{1}H NMR** (400 MHz, $CDCl_3$) δ 7.33 – 7.40 (m, 3H), 7.27 – 7.31 (m, 1H), 4.17 (q, *J* = 7.1 Hz, 2H), 3.64 (s, 2H), 1.32 (s, 9H), 1.27 (t, *J* = 7.1 Hz, 3H).

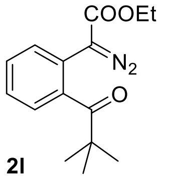

2l

Ethyl 2-diazo-2-(2-pivaloylphenyl)acetate (2l): Yellow liquid; 78 mg, 41% yield; **^{1}H NMR** (400 MHz, $CDCl_3$) δ 7.33 – 7.39 (m, 2H), 7.22 – 7.28 (m, 2H), 4.19 (q, *J* = 7.1 Hz, 2H), 1.22 (t, *J* = 7.1 Hz, 3H), 1.20 (s, 9H); **^{13}C NMR** (100 MHz, $CDCl_3$) δ 212.9, 165.8, 141.0, 131.6, 129.3, 127.7, 126.0, 122.3, 61.2, 44.9, 27.6, 14.5; **IR** (film) $υ_{max}$ 2974, 2934, 2871, 2092, 1740, 1698, 1479, 1461, 1393, 1368, 1290, 1248, 1175, 1054, 964, 759 cm^{-1}; **HRMS** (ESI) m/z 297.1200 [M + Na]$^+$; calculated for $[C_{15}H_{18}N_2O_3 + Na]^+$: 297.1210.

Table 1: **Catalyst screening and optimization studies.[a]**

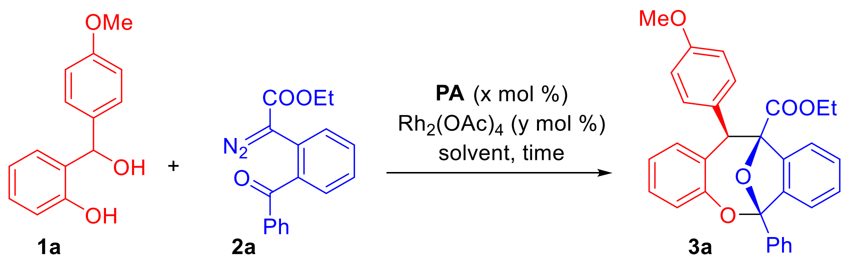

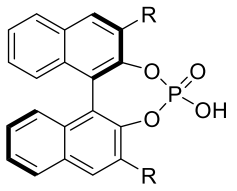

R = 3,5-$(CF_3)_2$-C_6H_3; **PA1**
R = 9-phenanthryl; **PA2**
R = 2,4,6-(^{i}Pr)$_3$-C_6H_2; **PA3**
R = 2,6-Me-4-^{t}Bu-C_6H_2; **PA4**
R = 2,4,6-Me-C_6H_2; **PA5**
R = 2,4,6-Et-C_6H_2; **PA6**
R = 2,3,4,5,6-Me-C_6; **PA7**

entry	**PA**	solvent	time [h]	**3a** (%)[b,c]	er[d]	dr[e]
1	**1**	$CHCl_3$	12	77	66:34	16:1
2	**2**	$CHCl_3$	12	71	82:18	20:1
3	**3**	$CHCl_3$	12	75	69:31	10:1
4	**4**	$CHCl_3$	12	79	83:17	20:1
5	**5**	$CHCl_3$	12	80	84:16	20:1
6	**6**	$CHCl_3$	12	73	88:12	20:1
7	**7**	$CHCl_3$	12	79	92:8	20:1
8	**7**	CH_2Cl_2	12	83	83:17	20:1
9	**7**	1,2-DCE	12	75	83:17	15:1
10	**7**	PhMe	48	58	85:15	8:1
11	**7**	CPME	48	trace	ND	ND
12[f]	**7**	$CHCl_3$	12	96	96:4	20:1
13[f,g]	**7**	$CHCl_3$	12	94	96:4	7:1
14[f,h]	**7**	$CHCl_3$	12	94	96:4	9:1
15[f,i]	**7**	$CHCl_3$	12	96	96:4	10:1
16[f,j]	**7**	$CHCl_3$	30	63	95:5	10:1

[a]Reactions were carried out with 0.10 mmol of **1a**, 0.11 mmol of **2a** and $Rh_2(OAc)_4$ (5 mol %) in the presence of catalyst **PA** (10 mol %) in $CHCl_3$ (3 mL). [b]Isolated yield of both diastereomers after chromatographic purification. [c]Decomposition accounts for remainder of mass balance. [d]Enantiomeric ratios (e.r.) were determined by chiral HPLC. [e]Diastereomeric ratios (d.r.) were determined from ^1H NMR of crude reaction mixture. [f] With 3 Å MS (35 mg). [g]With Rh/PA (5.0/5.0 mol %). [h]With Rh/PA (2.0/5.0 mol %). [i]With Rh/PA (2.0/10 mol %). [j]Reaction was conducted at 0 °C.

General procedure for catalytic diastereo- and enantioselective [4+3]-cycloannulation reaction between *in situ* generated *o*-QMs of compound 1 and *α*-diazoesters 2:

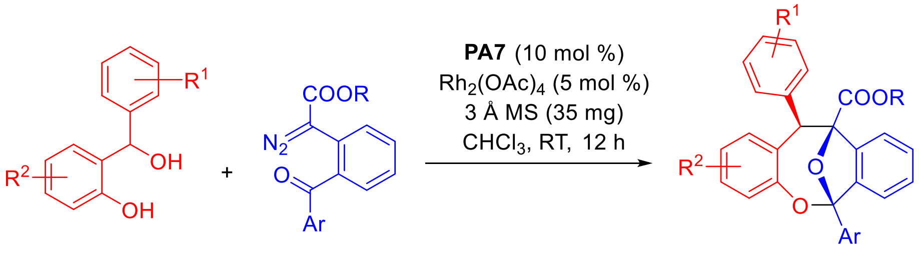

1 **2** **3 or 4**

A solution of **1** (0.1 mmol), (*R*)-**PA7** (6.4 mg, 0.01 mmol), 3 Å MS (35 mg) and $Rh_2(OAc)_4$ (2.2 mg, 0.005 mmol) were dissolved in $CHCl_3$ (2 mL). A solution of diazo compound **2** (0.11 mmol) in $CHCl_3$ (1 mL) was then added to the reaction mixture for a period of 1 h by using syringe pump and stirred at the indicated temperature in a closed vessel for 12 h. The product was purified over silica gel by flash column chromatography using EtOAc/hexanes mixtures as eluent.

Analytical data of all new compounds (data provided for major diastereomer):

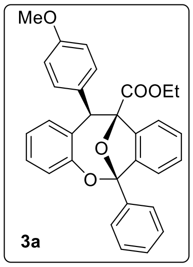

Ethyl (6*R*,11*R*,12*R*)-12-(4-methoxyphenyl)-6-phenyl-6*H*-6,11-epoxydibenzo[*b*,*f*]oxocine-11(12*H*)-carboxylate (3a): A solution of **1a** (23 mg, 0.1 mmol), (*R*)-**PA7** (6.4 mg, 0.01 mmol), 3 Å MS (35 mg) and $Rh_2(OAc)_4$ (2.2 mg, 0.005 mmol) were dissolved in $CHCl_3$ (2 mL). A solution of diazo compound **2a** (33 mg, 0.11 mmol) in $CHCl_3$ (1 mL) was then added slowly for a period of 1 h by using syringe pump to the reaction mixture and stirred at rt in a closed vessel for 12 h. The product was purified over silica gel by column chromatography (5-7% EtOAc in hexanes). The compound was obtained as a white solid (20:1 dr, 96% of isolated yield for both diastereomers, 46 mg); $\boldsymbol{R_f}$ = 0.25 (15% EtOAc-Hexane); **MP**: 242-244 °C; **^{1}H NMR** (400 MHz, $CDCl_3$) *δ* 7.94 – 7.97 (m, 2H), 7.60 (d, *J* = 7.5 Hz, 1H), 7.43 – 7.52 (m, 3H), 7.40 (d, *J* = 8.7 Hz, 2H), 7.24 – 7.28 (m, 1H), 7.19 (td, *J* = 7.4, 1.1 Hz, 1H), 7.00 – 7.06 (m, 2H), 6.95 – 6.97 (m, 2H), 6.75 – 6.80 (m, 3H), 4.96 (s, 1H), 4.29 (q, *J* = 7.1 Hz, 2H), 3.74 (s, 3H), 1.29 (t, *J* = 7.1 Hz, 3H); **^{13}C NMR** (100 MHz, $CDCl_3$) δ 169.5, 158.6, 152.9, 142.1, 139.5, 139.3, 132.3, 131.8, 130.6, 129.9, 129.5, 128.8, 128.2, 127.8, 126.8, 123.2, 122.7, 122.2, 120.4, 113.7, 110.2, 90.0, 62.9, 61.8, 55.1, 14.2; **IR** (film) $υ_{max}$ 3065, 2929, 2836, 1757, 1719, 1608, 1510, 1302, 1249, 1212, 1098, 983, 762 cm^{-1}; **HRMS** (ESI) m/z 501.1662 $[M + Na]^+$; calculated for $[C_{31}H_{26}O_5 + Na]^+$: 501.1672; Enantiomeric ratio was determined *via* HPLC analysis using a Chiralpak IE column; solvent: hexane/2-propanol = 90/10; flow rate: 1.0 mL/min; detection at 230 nm; t_R major = 6.74 min, t_R minor = 7.77 min; $[α]_D^{25}$ = –247.1 (*c* = 0.34, $CHCl_3$ for 96:4 er).

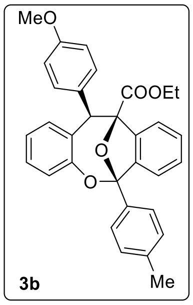

Ethyl (6*R*,11*R*,12R)-12-(4-methoxyphenyl)-6-(*p*-tolyl)-6*H*-6,11-epoxydibenzo[*b*,*f*]oxocine-11(12*H*)-carboxylate (3b): A solution of **1a** (23 mg, 0.1 mmol), (*R*)-**PA7** (6.4 mg, 0.01 mmol), 3 Å MS (35 mg) and $Rh_2(OAc)_4$ (2.2 mg, 0.005 mmol) were dissolved in $CHCl_3$ (2 mL). A solution of diazo compound **2b** (34 mg, 0.11 mmol) in $CHCl_3$ (1 mL) was then added slowly for a period of 1 h by using syringe pump to the reaction mixture and stirred at rt in a closed vessel for 12 h. The product was purified over silica gel by column chromatography (5-7% EtOAc in hexanes). The compound was obtained as a white solid (14:1 dr, 86% of isolated yield for both diastereomers, 42 mg); R_f = 0.25 (15% EtOAc-Hexane); **MP**: 210-212 °C; **^{1}H NMR** (400 MHz, $CDCl_3$) δ 7.82 (d, J = 8.2 Hz, 2H), 7.56 (d, J = 7.5 Hz, 1H), 7.38 (d, J = 8.8 Hz, 2H), 7.25 – 7.28 (m, 2H), 7.23 (td, J = 7.5, 1.2 Hz, 1H), 7.15 (td, J = 7.4, 1.1 Hz, 1H), 6.98 – 7.02 (m, 2H), 6.91 – 6.95 (m, 2H), 6.70 – 6.77 (m, 3H), 4.92 (s, 1H), 4.25 (q, J = 7.0 Hz, 2H), 3.71 (s, 3H), 2.41 (s, 3H), 1.25 (t, J = 7.1 Hz, 3H); **^{13}C NMR** (100 MHz, $CDCl_3$) δ 169.6, 158.6, 152.9, 142.1, 139.6, 138.5, 136.5, 132.2, 131.9, 130.6, 129.9, 129.4, 129.3, 128.9, 127.7, 126.7, 123.1, 122.7, 122.2, 120.4, 113.7, 110.2, 89.9, 62.8, 61.8, 55.1, 21.2, 14.1; IR (film) υ_{max} 2930, 2835, 1758, 1725, 1609, 1510, 1483, 1460, 1443, 1301, 1247, 1210, 1180, 1058, 1010, 961, 761 cm^{-1}; **HRMS** (ESI) m/z 515.1831 [M + Na]$^+$; calculated for $[C_{32}H_{28}O_5 + Na]^+$: 515.1829; Enantiomeric ratio was determined *via* HPLC analysis using a Chiralpak IA column; solvent: hexane/2-propanol = 90/10; flow rate: 1.0 mL/min; detection at 230 nm; t_R minor = 7.41 min, t_R major = 14.0 min; $[\alpha]_D^{25}$ = –202.6 (c = 0.38, $CHCl_3$ for 94:6 er).

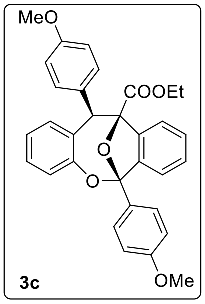

Ethyl (6*R*,11*R*,12*R*)-6,12-bis(4-methoxyphenyl)-6*H*-6,11-epoxydibenzo[*b*,*f*]oxocine-11(12*H*)-carboxylate (3c): A solution of **1a** (23 mg, 0.1 mmol), (*R*)-**PA7** (6.4 mg, 0.01 mmol), 3 Å MS (35 mg) and $Rh_2(OAc)_4$ (2.2 mg, 0.005 mmol) were dissolved in $CHCl_3$ (2 mL). A solution of diazo compound **2c** (36 mg, 0.11 mmol) in $CHCl_3$ (1 mL) was then added slowly for a period of 1 h by using syringe pump to the reaction mixture and stirred at rt in a closed vessel for 12 h. The product was purified over silica gel by column chromatography (8-12% EtOAc in hexanes). The compound was obtained as a white solid (>20:1 dr, 94% of isolated yield for both diastereomers, 48 mg); R_f = 0.25 (20% EtOAc-Hexane); **MP**: 198-200 °C; **^{1}H NMR** (300 MHz, $CDCl_3$) δ 7.86 (d, J = 8.9 Hz, 2H), 7.56 (d, J = 7.7 Hz, 1H), 7.38 (d, J = 8.7 Hz, 2H), 7.20 – 7.26 (m, 1H), 7.16 (td, J = 7.4, 1.2 Hz, 1H), 6.91 – 7.02 (m, 6H), 6.71 – 6.78 (m, 3H), 4.93 (s, 1H), 4.26 (q, J = 7.1 Hz, 2H), 3.85 (s, 3H), 3.72 (s, 3H), 1.26 (t, J = 7.1 Hz, 3H); **^{13}C NMR** (100 MHz, $CDCl_3$) δ 169.6, 160.0, 158.6, 153.0, 142.1, 139.7, 132.3, 131.9, 131.8, 130.6, 129.9, 129.4, 129.4, 128.1, 127.8, 123.1, 122.7, 122.2,

120.4, 113.7, 113.6, 110.2, 89.9, 62.8, 61.8, 55.3, 55.1, 14.2; **IR** (film) υmax 3073, 2933, 2835, 1755, 1725, 1612, 1511, 1460, 1443, 1302, 1249, 1211, 1174, 1058, 1004, 836, 761 cm^{-1}; **HRMS** (ESI) m/z 531.1765 $[M + Na]^+$; calculated for $[C_{32}H_{28}O_6 + Na]^+$: 531.1778; Enantiomeric ratio was determined *via* HPLC analysis using a Chiralpak IA column; solvent: hexane/2-propanol = 90/10; flow rate: 1.0 mL/min; detection at 240 nm; t_R minor = 11.71 min, t_R major = 18.41 min; $[\alpha]_D^{25}$ = –238.1 (*c* = 0.21, $CHCl_3$ for 96:4 er).

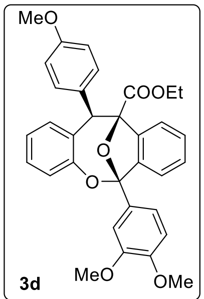

3d

Ethyl (6*R*,11*R*,12*R*)-6-(3,4-dimethoxyphenyl)-12-(4-methoxyphenyl)-6*H*-6,11-epoxydibenzo[*b*,*f*]oxocine-11(12*H*)-carboxylate (3d): A solution of **1a** (23 mg, 0.1 mmol), (*R*)-**PA7** (6.4 mg, 0.01 mmol), 3 Å MS (35 mg) and $Rh_2(OAc)_4$ (2.2 mg, 0.005 mmol) were dissolved in $CHCl_3$ (2 mL). A solution of diazo compound **2d** (39 mg, 0.11 mmol) in $CHCl_3$ (1 mL) was then added slowly for a period of 1 h by using syringe pump to the reaction mixture and stirred at rt in a closed vessel for 12 h. The product was purified over silica gel by column chromatography (10-18% EtOAc in hexanes). The compound was obtained as a pale yellow solid (>20:1 dr, 92% of isolated yield for both diastereomers, 49 mg); ***R$_f$*** = 0.2 (20% EtOAc-Hexane); **MP**: 190-192 °C; **^{1}H NMR** (400 MHz, $CDCl_3$) *δ* 7.52 – 7.56 (m, 2H), 7.42 (d, *J* = 2.1 Hz, 1H), 7.38 (d, *J* = 8.7 Hz, 2H), 7.22 – 7.26 (m, 2H), 7.17 (t, *J* = 7.4 Hz, 1H), 6.92 – 7.00 (m, 5H), 6.72 – 6.77 (m, 2H), 4.94 (s, 1H), 4.24 (qd, *J* = 7.2, 2.7 Hz, 2H), 3.93 (s, 3H), 3.92 (s, 3H), 3.72 (s, 3H), 1.25 (t, *J* = 7.1 Hz, 3H); **^{13}C NMR** (100 MHz, $CDCl_3$) δ 169.6, 158.6, 152.9, 149.4, 148.7, 142.1, 139.6, 132.3, 132.1, 131.8, 130.7, 129.9, 129.5, 129.4, 127.8, 123.2, 122.6, 122.2, 120.4, 119.3, 113.7, 110.7, 110.2, 110.2, 89.9, 62.7, 61.8, 56.0, 55.8, 55.1, 14.2; **IR** (film) υmax 2933, 2835, 1755, 1727, 1609, 1511, 1462, 1302, 1248, 1208, 1139, 1027, 861, 759 cm^{-1}; **HRMS** (ESI) m/z 561.1881 $[M+Na]^+$; calculated for $[C_{33}H_{30}O_7 + Na]^+$: 561.1884; Enantiomeric ratio was determined *via* HPLC analysis using a Chiralpak IA column; solvent: hexane/2-propanol = 90/10; flow rate: 1.0 mL/min; detection at 230 nm; t_R minor = 12.84 min, t_R major = 16.52 min; $[\alpha]_D^{25}$ = –192.0 (*c* = 0.5, $CHCl_3$ for 97:3 er).

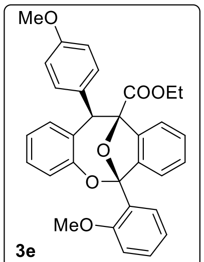

3e

Ethyl (6*S*,11*R*,12*R*)-6-(2-methoxyphenyl)-12-(4-methoxyphenyl)-6*H*-6,11-epoxydibenzo[*b*,*f*]oxocine-11(12*H*)-carboxylate (3e): A solution of **1a** (23 mg, 0.1 mmol), (*R*)-**PA7** (6.4 mg, 0.01 mmol), 3 Å MS (35 mg) and $Rh_2(OAc)_4$ (2.2 mg, 0.005 mmol) were dissolved in $CHCl_3$ (2 mL). A solution of diazo compound **2e** (36 mg, 0.11 mmol) in $CHCl_3$ (1 mL) was then added slowly for a period of 1 h by using syringe pump to the reaction mixture and stirred at rt in a closed vessel for 12 h. The product was purified over silica gel by column chromatography (8-12% EtOAc in hexanes).

The compound was obtained as a white solid (6:1 dr, 82% of isolated yield for both diastereomers, 42 mg); ***R_f*** = 0.25 (20% EtOAc-Hexane); **MP**: 84-86 °C; **^{1}H NMR** (400 MHz, $CDCl_3$) *δ* 8.27 (dd, *J* = 7.8, 1.1 Hz, 1H), 7.61 (d, *J* = 7.5 Hz, 1H), 7.39 – 7.45 (m, 3H), 7.21 – 7.27 (m, 2H), 7.16 (td, *J* = 7.4, 1.1 Hz, 1H), 7.11 (td, *J* = 7.5, 1.1 Hz, 1H), 6.99 – 7.03 (m, 3H), 6.95 (d, *J* = 7.9 Hz, 1H), 6.74 – 6.79 (m, 3H), 4.91 (s, 1H), 4.21 – 4.30 (m, 2H), 3.74 (s, 3H), 3.73 (s, 3H), 1.26 (t, *J* = 7.1 Hz, 3H); ^{13}C NMR (100 MHz, $CDCl_3$) δ 169.8, 158.6, 157.4, 153.0, 142.2, 139.7, 132.1, 132.0, 130.6, 130.5, 130.4, 129.1, 129.1, 128.7, 127.7, 127.1, 123.0, 122.5, 122.3, 120.3, 120.2, 113.7, 112.3, 109.3, 89.7, 63.3, 61.6, 55.5, 55.1, 14.2; **IR** (film) $υ_{max}$ 2933, 2834, 1758, 1724, 1603, 1509, 1461, 1439, 1370, 1302, 1247, 1211, 1179, 1029, 960, 759 cm^{-1}; **HRMS** (ESI) m/z 531.1769 $[M + Na]^+$; calculated for $[C_{32}H_{28}O_6 + Na]^+$: 531.1778; Enantiomeric ratio was determined *via* HPLC analysis using a Chiralpak IE column; solvent: hexane/2-propanol = 90/10; flow rate: 1.0 mL/min; detection at 230 nm; t_R major = 7.96 min, t_R minor = 10.09 min; $[α]_D^{25}$ = –176.0 (*c* = 0.25, $CHCl_3$ for 89:11 er).

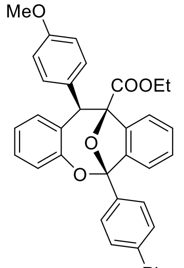

3f

Ethyl (6*R*,11*R*,12*R*)-6-([1,1'-biphenyl]-4-yl)-12-(4-methoxyphenyl)-6*H*-6,11-epoxydibenzo[*b*,*f*]oxocine-11(12*H*)-carboxylate (3f): A solution of **1a** (23 mg, 0.1 mmol), (*R*)-**PA7** (6.4 mg, 0.01 mmol), 3 Å MS (35 mg) and $Rh_2(OAc)_4$ (2.2 mg, 0.005 mmol) were dissolved in $CHCl_3$ (2 mL). A solution of diazo compound **2f** (42 mg, 0.11 mmol) in $CHCl_3$ (1 mL) was then added slowly for a period of 1 h by using syringe pump to the reaction mixture and stirred at rt in a closed vessel for 12 h. The product was purified over silica gel by column chromatography (5-7% EtOAc in hexanes). The compound was obtained as a white solid (12:1 dr, 93% of isolated yield for both diastereomers, 52 mg); ***R_f*** = 0.25 (15% EtOAc-Hexane); **MP**: 96-98 °C; **^{1}H NMR** (300 MHz, $CDCl_3$) *δ* 7.98 – 8.02 (m, 2H), 7.67 – 7.71 (m, 2H), 7.61 – 7.66 (m, 2H), 7.57 – 7.60 (m, 1H), 7.43 – 7.49 (m, 2H), 7.34 – 7.42 (m, 3H), 7.23 – 7.28 (m, 1H), 7.19 (td, *J* = 7.4, 1.2 Hz, 1H), 6.98 – 7.07 (m, 3H), 6.93 – 6.97 (m, 1H), 6.73 – 6.79 (m, 3H), 4.95 (s, 1H), 4.27 (qd, *J* = 7.1, 0.9 Hz, 2H), 3.72 (s, 3H), 1.27 (t, *J* = 7.1 Hz, 3H); **^{13}C NMR** (100 MHz, $CDCl_3$) δ 169.5, 158.6, 152.9, 142.2, 141.7, 140.9, 139.4, 138.3, 132.3, 131.8, 130.6, 129.9, 129.5, 128.7, 127.8, 127.4, 127.2, 127.2, 127.0, 123.2, 122.7, 122.2, 120.5, 113.7, 110.1, 90.0, 62.9, 61.8, 55.1, 14.2; **IR** (film) $υ_{max}$ 2930, 2835, 1757, 1725, 1610, 1509, 1483, 1443, 1301, 1247, 1211, 1179, 1097, 1007, 764 cm^{-1}; **HRMS** (ESI) m/z 577.1996 $[M + Na]^+$; calculated for $[C_{37}H_{30}O_5 + Na]^+$: 577.1985; Enantiomeric ratio was determined *via* HPLC analysis using a Chiralpak IA column; solvent: hexane/2-propanol = 90/10; flow rate: 1.0 mL/min; detection at 230 nm; t_R minor = 11.31 min, t_R major = 19.05 min; $[α]_D^{25}$ = –171.4 (*c* = 0.21, $CHCl_3$ for 96:4 er).

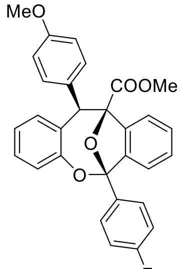

3g

Methyl (6*R*,11*R*,12*R*)-6-(4-fluorophenyl)-12-(4-methoxyphenyl)-6*H*-6,11-epoxydibenzo[*b*,*f*]oxocine-11(12*H*)-carboxylate (3g): A solution of **1a** (23 mg, 0.1 mmol), (*R*)-**PA7** (6.4 mg, 0.01 mmol), 3 Å MS (35 mg) and $Rh_2(OAc)_4$ (2.2 mg, 0.005 mmol) were dissolved in $CHCl_3$ (2 mL). A solution of diazo compound **2g** (33 mg, 0.11 mmol) in $CHCl_3$ (1 mL) was then added slowly for a period of 1 h by using syringe pump to the reaction mixture and stirred at rt in a closed vessel for 12 h. The product was purified over silica gel by column chromatography (5-7% EtOAc in hexanes). The compound was obtained as a white solid (10:1 dr, 94% of isolated yield for both diastereomers, 45 mg); R_f = 0.25 (15% EtOAc-Hexane); **MP**: 94-96 °C; **^{1}H NMR** (400 MHz, $CDCl_3$) δ 7.88 – 7.92 (m, 2H), 7.57 (d, *J* = 7.5 Hz, 1H), 7.32 – 7.35 (m, 2H), 7.23 – 7.27(m, 1H), 7.11 – 7.19 (m, 3H), 6.98 – 7.01 (m, 2H), 6.91 – 6.94 (m, 2H), 6.73 – 6.77 (m, 3H), 4.94 (s, 1H), 3.76 (s, 3H), 3.71 (s, 3H); **^{13}C NMR** (100 MHz, $CDCl_3$) δ 170.0, 163.1 (d, *J* = 245.9 Hz), 158.7, 152.8, 141.9, 139.2, 135.2 (d, *J* = 2.9 Hz), 132.3, 131.5, 130.4, 129.7, 129.6, 129.6, 128.9 (d, *J* = 8.3 Hz), 127.9, 123.3, 122.6, 122.1, 120.5, 115.1 (d, *J* = 21.4 Hz), 113.8, 109.9, 90.2, 62.8, 55.0, 52.7; **IR** (film) υ_{max} 2953, 2835, 1761, 1732, 1609, 1510, 1441, 1303, 1248, 1211, 1123, 1059, 1011, 842, 761 cm^{-1}; **HRMS** (ESI) m/z 505.1416 $[M + Na]^+$; calculated for $[C_{30}H_{23}FO_5 + Na]^+$: 505.1422; Enantiomeric ratio was determined *via* HPLC analysis using a Chiralpak IA column; solvent: hexane/2-propanol = 95/5; flow rate: 1.0 mL/min; detection at 230 nm; t_R minor = 9.59 min, t_R major = 12.41 min; $[\alpha]_D^{25}$ = –189.5 (*c* = 0.38, $CHCl_3$ for 96:4 er).

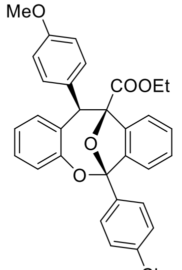

3h

Ethyl (6*R*,11*R*,12*R*)-6-(4-chlorophenyl)-12-(4-methoxyphenyl)-6*H*-6,11-epoxydibenzo[*b*,*f*]oxocine-11(12*H*)-carboxylate (3h): A solution of **1a** (23 mg, 0.1 mmol), (*R*)-**PA7** (6.4 mg, 0.01 mmol), 3 Å MS (35 mg) and $Rh_2(OAc)_4$ (2.2 mg, 0.005 mmol) were dissolved in $CHCl_3$ (2 mL). A solution of diazo compound **2h** (36 mg, 0.11 mmol) in $CHCl_3$ (1 mL) was then added slowly for a period of 1 h by using syringe pump to the reaction mixture and stirred at rt in a closed vessel for 12 h. The product was purified over silica gel by column chromatography (5-7% EtOAc in hexanes). The compound was obtained as a white solid (10:1 dr, 92% of isolated yield for both diastereomers, 47 mg); R_f = 0.3 (15% EtOAc-Hexane); **MP**: 84-86 °C; **^{1}H NMR** (300 MHz, $CDCl_3$) δ 7.85 – 7.89 (m, 2H), 7.58 (d, *J* = 7.3 Hz, 1H), 7.42 – 7.47 (m, 2H), 7.33 – 7.40 (m, 2H), 7.22 – 7.27 (m, 1H), 7.17 (t, *J* = 7.3 Hz, 1H), 6.99 – 7.07 (m, 2H), 6.92 (t, *J* = 8.3 Hz, 2H), 6.71 – 6.78 (m, 3H), 4.94 (s, 1H), 4.22 – 4.30 (m, 2H), 3.72 (s, 3H), 1.27 (t, *J* = 6.9 Hz, 3H); **^{13}C NMR** (100 MHz, $CDCl_3$) δ 169.3, 158.7, 152.7, 142.1, 139.1, 137.9, 134.8, 132.3, 131.6, 130.6, 129.8, 129.7, 129.6, 128.4, 128.4,

127.9, 123.3, 122.5, 122.1, 120.5, 113.7, 109.7, 90.1, 62.8, 61.9, 55.1, 14.2; **IR** (film) $υ_{max}$ 3072, 2932, 2835, 1757, 1727, 1609, 1509, 1483, 1443, 1303, 1248, 1211, 1179, 1091, 1008, 835, 762 cm^{-1}; **HRMS** (ESI) m/z 535.1284 $[M + Na]^+$; calculated for $[C_{31}H_{25}ClO_5 + Na]^+$: 535.1283; Enantiomeric ratio was determined *via* HPLC analysis using a Chiralpak IA column; solvent: hexane/2-propanol = 95/5; flow rate: 1.0 mL/min; detection at 230 nm; t_R minor = 9.64 min, t_R major = 15.24 min; $[α]_D^{25}$ = –200.0 (*c* = 0.37, $CHCl_3$ for 96:4 er).

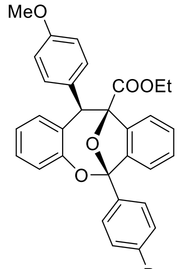

3i

Ethyl (6*R*,11*R*,12*R*)-6-(4-bromophenyl)-12-(4-methoxyphenyl)-6*H*-6,11-epoxydibenzo[*b*,*f*]oxocine-11(12*H*)-carboxylate (3i): A solution of **1a** (23 mg, 0.1 mmol), (*R*)-**PA7** (6.4 mg, 0.01 mmol), 3 Å MS (35 mg) and $Rh_2(OAc)_4$ (2.2 mg, 0.005 mmol) were dissolved in $CHCl_3$ (2 mL). A solution of diazo compound **2i** (41 mg, 0.11 mmol) in $CHCl_3$ (1 mL) was then added slowly for a period of 1 h by using syringe pump to the reaction mixture and stirred at rt in a closed vessel for 12 h. The product was purified over silica gel by column chromatography (5-7% EtOAc in hexanes). The compound was obtained as a white solid (8:1 dr, 91% of isolated yield for both diastereomers, 51 mg); R_f = 0.3 (15% EtOAc-Hexane); **MP**: 180-182 °C; **^{1}H NMR** (400 MHz, $CDCl_3$) *δ* 7.81 (d, *J* = 8.5 Hz, 2H), 7.56 – 7.60 (m, 3H), 7.36 (d, *J* = 8.7 Hz, 2H), 7.22 – 7.26 (m, 1H), 7.17 (td, *J* = 7.5, 1.1 Hz, 1H), 6.99 – 7.00 (m, 2H), 6.89 – 6.94 (m, 2H), 6.71 – 6.78 (m, 3H), 4.93 (s, 1H), 4.21 – 4.29 (m, 2H), 3.72 (s, 3H), 1.26 (t, *J* = 7.1 Hz, 3H); **^{13}C NMR** (100 MHz, $CDCl_3$) δ 169.3, 158.7, 152.7, 142.1, 139.1, 138.4, 132.3, 131.6, 131.4, 130.6, 129.8, 129.7, 129.6, 128.7, 127.9, 123.4, 123.1, 122.5, 122.1, 120.5, 113.7, 109.8, 90.1, 62.8, 61.9, 55.1, 14.2; **IR** (film) $υ_{max}$ 2928, 2835, 1758, 1725, 1605, 1508, 1484, 1447, 1370, 1298, 1241, 1213, 1009, 967, 760 cm^{-1}; **HRMS** (ESI) m/z 579.0766 $[M + Na]^+$; calculated for $[C_{31}H_{25}{}^{79}BrO_5 + Na]^+$: 579.0778; Enantiomeric ratio was determined *via* HPLC analysis using a Chiralpak IA column; solvent: hexane/2-propanol = 95/5; flow rate: 1.0 mL/min; detection at 230 nm; t_R minor = 10.08 min, t_R major = 17.1 min; $[α]_D^{25}$ = –200.0 (*c* = 0.34, $CHCl_3$ for 96:4 er).

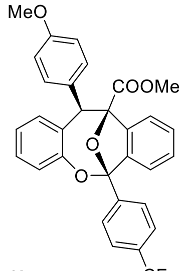

3j

Methyl (6*R*,11*R*,12*R*)-12-(4-methoxyphenyl)-6-(4-(trifluoromethyl)phenyl)-6*H*-6,11-epoxydibenzo[*b*,*f*]oxocine-11(12*H*)-carboxylate (3j): A solution of **1a** (23 mg, 0.1 mmol), (*R*)-**PA7** (6.4 mg, 0.01 mmol), 3 Å MS (35 mg) and $Rh_2(OAc)_4$ (2.2 mg, 0.005 mmol) were dissolved in $CHCl_3$ (2 mL). A solution of diazo compound **2j** (39 mg, 0.11 mmol) in $CHCl_3$ (1 mL) was then added slowly for a period of 1 h by using syringe pump to the reaction mixture and stirred at

rt in a closed vessel for 12 h. The product was purified over silica gel by column chromatography (5-7% EtOAc in hexanes). The compound was obtained as a white solid (6:1 dr, 81% of isolated yield for both diastereomers, 43 mg); ***R*** R_f = 0.25 (15% EtOAc-Hexane); **MP**: 108-110 °C; **^{1}H NMR** (400 MHz, $CDCl_3$) *δ* 8.06 (d, *J* = 8.2 Hz, 2H), 7.73 (d, *J* = 8.3 Hz, 2H), 7.60 (d, *J* = 7.6 Hz, 1H), 7.33 (d, *J* = 8.8 Hz, 2H), 7.24 – 7.28 (m, 1H), 7.19 (t, *J* = 7.5 Hz, 1H), 7.02 (d, *J* = 4.2 Hz, 2H), 6.95 (d, *J* = 7.9 Hz, 1H), 6.91 (d, *J* = 7.5 Hz, 1H), 6.74 – 6.79 (m, 3H), 4.95 (s, 1H), 3.77 (s, 3H), 3.72 (s, 3H); **^{13}C NMR** (101 MHz, $CDCl_3$) δ 169.9, 158.7, 152.6, 142.9, 142.0, 138.7, 132.3, 131.4, 130.4, 129.8, 129.7, 129.6, 128.0, 127.4, 127.3, 125.3 (q, *J* = 3.7 Hz), 123.5, 122.5, 122.1, 120.6, 113.8, 109.6, 90.5, 62.8, 55.0, 52.8; **IR** (film) $υ_{max}$ 2952, 2841, 1764, 1725, 1608, 1509, 1485, 1444, 1411, 1325, 1249, 1212, 1168, 1130, 1068, 1013, 946, 860, 763 cm^{-1}; **HRMS** (ESI) m/z 555.1385 $[M + Na]^+$; calculated for $[C_{31}H_{23}F_3O_5 + Na]^+$: 555.1390; Enantiomeric ratio was determined *via* HPLC analysis using a Chiralpak IA column; solvent: hexane/2-propanol = 90/10; flow rate: 1.0 mL/min; detection at 230 nm; t_R minor = 6.13 min, t_R major = 8.61 min; $[α]_D^{25}$ = –173.3 (*c* = 0.3, $CHCl_3$ for 96:4 er).

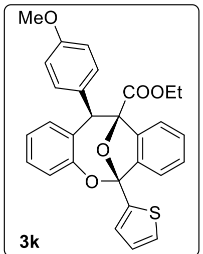

3k

Ethyl (6*S*,11*R*,12*R*)-12-(4-methoxyphenyl)-6-(thiophen-2-yl)-6*H*-6,11-epoxydibenzo[*b*,*f*]oxocine-11(12*H*)-carboxylate (3k): A solution of **1a** (23 mg, 0.1 mmol), (*R*)-**PA7** (6.4 mg, 0.01 mmol), 3 Å MS (35 mg) and $Rh_2(OAc)_4$ (2.2 mg, 0.005 mmol) were dissolved in $CHCl_3$ (2 mL). A solution of diazo compound **2k** (33 mg, 0.11 mmol) in $CHCl_3$ (1 mL) was then added slowly for a period of 1 h by using syringe pump to the reaction mixture and stirred at rt in a closed vessel for 12 h. The product was purified over silica gel by column chromatography (5-7% EtOAc in hexanes). The compound was obtained as a yellow solid (>20:1 dr, 92% of isolated yield for both diastereomers, 44 mg); R_f = 0.3 (15% EtOAc-Hexane); **MP**: 198-200 °C; **^{1}H NMR** (400 MHz, $CDCl_3$) *δ* 7.58 (d, *J* = 7.6 Hz, 1H), 7.37 – 7.43 (m, 4H), 7.24 – 7.28 (m, 1H), 7.21 (t, *J* = 7.4 Hz, 1H), 7.08 – 7.12 (m, 2H), 6.96 – 7.01 (m, 2H), 6.92 (d, *J* = 7.8 Hz, 1H), 6.72 – 6.79 (m, 3H), 4.91 (s, 1H), 4.23 (q, *J* = 7.1 Hz, 2H), 3.72 (s, 3H), 1.24 (t, *J* = 7.1 Hz, 3H); **^{13}C NMR** (100 MHz, $CDCl_3$) δ 169.3, 158.7, 152.6, 142.3, 142.0, 139.0, 132.3, 131.6, 130.6, 129.8, 129.6, 127.9, 127.0, 126.6, 126.4, 123.4, 122.7, 122.3, 120.5, 113.8, 109.0, 90.0, 62.9, 61.9, 55.1, 14.1; **IR** (film) $υ_{max}$ 2932, 2835, 1760, 1720, 1608, 1510, 1483, 1441, 1301, 1249, 1178, 1096, 981, 759 cm^{-1}; **HRMS** (ESI) m/z 507.1237 $[M + Na]^+$; calculated for $[C_{29}H_{24}O_5S + Na]^+$: 507.1237; Enantiomeric ratio was determined *via* HPLC analysis using a Chiralpak IA column; solvent: hexane/2-propanol = 90/10; flow rate: 1.0 mL/min; detection at 230 nm; t_R minor = 8.84 min, t_R major = 9.86 min; $[α]_D^{25}$ = –187.5 (*c* = 0.32, $CHCl_3$ for 95:5 er).

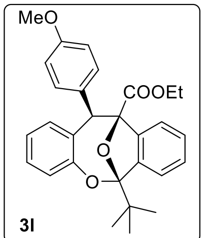

3l

Ethyl (6*R*,11*R*,12*R*)-6-(*tert*-butyl)-12-(4-methoxyphenyl)-6*H*-6,11-epoxydibenzo[*b*,*f*]oxocine-11(12*H*)-carboxylate (3l): A solution of **1a** (23 mg, 0.1 mmol), (*R*)-**PA7** (6.4 mg, 0.01 mmol), 3 Å MS (35 mg) and $Rh_2(OAc)_4$ (2.2 mg, 0.005 mmol) were dissolved in $CHCl_3$ (2 mL). A solution of diazo compound **2l** (30 mg, 0.11 mmol) in $CHCl_3$ (1 mL) was then added slowly for a period of 1 h by using syringe pump to the reaction mixture and stirred at rt in a closed vessel for 12 h. The product was purified over silica gel by column chromatography (3-6% EtOAc in hexanes). The compound was obtained as a colorless oil (6:1 dr, 83% of isolated yield for both diastereomers, 38 mg); R_f = 0.52 (15% EtOAc-Hexane); **^{1}H NMR** (400 MHz, $CDCl_3$) δ 7.51 – 7.53 (m, 1H), 7.45 – 7.47 (m, 1H), 7.37 – 7.40 (m, 2H), 7.19 – 7.27 (m, 2H), 6.91 – 6.95 (m, 1H), 6.87 (dd, *J* = 7.7, 1.7 Hz, 1H), 6.78 – 6.83 (m, 3H), 6.65 – 6.70 (m, 1H), 4.79 (s, 1H), 4.12 – 4.25 (m, 2H), 3.78 (s, 3H), 1.41 (s, 9H), 1.23 (t, *J* = 7.1 Hz, 3H); **^{13}C NMR** (100 MHz, $CDCl_3$) δ 169.7, 158.5, 153.6, 143.8, 137.6, 132.4, 132.2, 130.7, 129.7, 129.2, 129.0, 127.4, 123.2, 122.4, 121.9, 120.6, 114.9, 113.4, 88.6, 62.9, 61.4, 55.1, 38.8, 25.7, 14.1; **IR** (film) υ_{max} 2963, 2909, 2835, 1758, 1726, 1609, 1571, 1509, 1482, 1393, 1364, 1292, 1248, 1180, 1130, 1035, 967, 757 cm^{-1}; **HRMS** (ESI) m/z 481.1995 $[M + Na]^+$; calculated for $[C_{29}H_{30}O_5 + Na]^+$: 481.1985; Enantiomeric ratio was determined *via* HPLC analysis using a Chiralpak IA column; solvent: hexane/2-propanol = 99/1; flow rate: 0.5 mL/min; detection at 240 nm; t_R major = 12.70 min, t_R minor = 15.90 min; $[\alpha]_D^{25}$ = –63 (*c* = 1.34, $CHCl_3$ for 91:9 er).

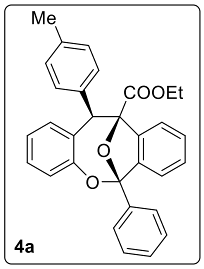

4a

Ethyl (6*R*,11*R*,12*R*)-6-phenyl-12-(*p*-tolyl)-6*H*-6,11-epoxydibenzo[*b*,*f*]oxocine-11(12*H*)-carboxylate (4a): A solution of **1b** (22 mg, 0.1 mmol), (*R*)-**PA7** (6.4 mg, 0.01 mmol), 3 Å MS (35 mg) and $Rh_2(OAc)_4$ (2.2 mg, 0.005 mmol) were dissolved in $CHCl_3$ (2 mL). A solution of diazo compound **2a** (33 mg, 0.11 mmol) in $CHCl_3$ (1 mL) was then added slowly for a period of 1 h by using syringe pump to the reaction mixture and stirred at 40 °C in a closed vessel for 12 h. The product was purified over silica gel by column chromatography (3-5% EtOAc in hexanes). The compound was obtained as a white solid (14:1 dr, 82% of isolated yield for both diastereomers, 38 mg); R_f = 0.35 (10% EtOAc-Hexane); **MP**: 184-186 °C; **^{1}H NMR** (400 MHz, $CDCl_3$) *δ* 7.92 – 7.95 (m, 2H), 7.58 (dt, *J* = 7.6, 1.0 Hz, 1H), 7.40 – 7.49 (m, 3H), 7.32 – 7.34 (m, 2H), 7.21 – 7.25 (m, 1H), 7.16 (td, *J* = 7.5, 1.1 Hz, 1H), 6.97 – 7.04 (m, 4H), 6.92 – 6.95 (m, 2H), 6.71 – 6.76 (m, 1H), 4.95 (s, 1H), 4.26 (qd, *J* = 7.1, 0.8 Hz, 2H), 2.24 (s, 3H), 1.26 (t, *J* = 7.1 Hz, 3H); **^{13}C NMR** (100 MHz, $CDCl_3$) δ 169.5, 153.0, 142.1, 139.5, 139.3, 136.7, 136.5, 132.3, 129.8, 129.4, 129.4, 129.0, 128.7, 128.2, 127.8, 126.8, 123.1, 122.7, 122.1, 120.5, 110.1, 90.0, 63.3, 61.8, 21.0, 14.1; **IR** (film) υ_{max} 3065, 2977, 2922, 1759, 1720, 1605, 1512, 1483, 1447, 1300, 1244, 1211, 1123, 1098, 1057, 961, 760 cm^{-1}; **HRMS** (ESI) m/z 485.1729 $[M + Na]^+$; calculated for $[C_{31}H_{26}O_4 + Na]^+$: 485.1723; Enantiomeric ratio was determined *via* HPLC analysis using a Chiralpak IA column; solvent: hexane/2-propanol = 95/5; flow rate: 1.0 mL/min; detection at 230 nm; t_R minor = 8.83 min, t_R major = 9.50 min; $[\alpha]_D^{25}$ = –217.14 (*c* = 0.35, $CHCl_3$ for 97:3 er).

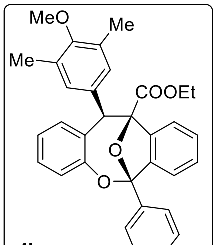

4b

Ethyl (6*R*,11*R*,12*R*)-12-(4-methoxy-3,5-dimethylphenyl)-6-phenyl-6*H*-6,11-epoxydibenzo[*b*,*f*]oxocine-11(12*H*)-carboxylate (4b): A solution of **1c** (26 mg, 0.1 mmol), (*R*)-**PA7** (6.4 mg, 0.01 mmol), 3 Å MS (35 mg) and $Rh_2(OAc)_4$ (2.2 mg, 0.005 mmol) were dissolved in $CHCl_3$ (2 mL). A solution of diazo compound **2a** (33 mg, 0.11 mmol) in $CHCl_3$ (1 mL) was then added slowly for a period of 1 h by using syringe pump to the reaction mixture and stirred at 40 °C in a closed vessel for 12 h. The product was purified over silica gel by column chromatography (5-7% EtOAc in hexanes). The compound was obtained as a white solid (17:1 dr, 92% of isolated yield for both diastereomers, 46 mg); R_f = 0.25 (15% EtOAc-Hexane); **MP**: >300 °C; **^{1}H NMR** (400 MHz, $CDCl_3$) δ 7.91 – 7.93 (m, 2H), 7.58 (dt, *J* = 7.6, 0.9 Hz, 1H), 7.39 – 7.49 (m, 3H), 7.23 (td, *J* = 7.5, 1.1 Hz, 1H), 7.15 (td, *J* = 7.4, 1.1 Hz, 1H), 6.98 – 7.04 (m, 4H), 6.90 – 6.93 (m, 2H), 6.75 (ddd, *J* = 7.7, 6.1, 2.4 Hz, 1H), 4.87 (s, 1H), 4.21 – 4.35 (m, 2H), 3.62 (s, 3H), 2.18 (s, 6H), 1.26 (t, *J* = 7.1 Hz, 3H); **^{13}C NMR** (100 MHz, $CDCl_3$) δ 169.5, 156.1, 153.0, 142.2, 139.5, 139.3, 134.6, 132.3, 130.3, 129.9, 129.8, 129.4, 128.7, 128.2, 127.8, 126.8, 123.1, 122.7, 122.2, 120.5, 110.2, 90.0, 63.2, 61.8, 59.3, 16.2, 14.2; **IR** (film) υ_{max} 2933, 2836, 1758, 1725, 1604, 1483, 1447, 1370, 1297, 1241, 1213, 1099, 1013, 939, 759 cm^{-1}; **HRMS** (ESI) m/z 529.1980 $[M + Na]^+$; calculated for $[C_{33}H_{30}O_5 + Na]^+$: 529.1985; Enantiomeric ratio was determined *via* HPLC analysis using a Chiralpak IE column; solvent: hexane/2-propanol = 95/5; flow rate: 1.0 mL/min; detection at 230 nm; t_R major = 5.97 min, t_R minor = 6.56 min; $[\alpha]_D^{25}$ = –180.0 (*c* = 0.3, $CHCl_3$ for 97:3 er).

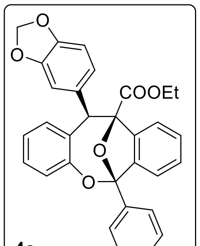

4c

Ethyl (6*R*,11*R*,12*R*)-12-(benzo[*d*][1,3]dioxol-5-yl)-6-phenyl-6*H*-6,11-epoxydibenzo[*b*,*f*]oxocine-11(12*H*)-carboxylate (4c): A solution of **1d** (25 mg, 0.1 mmol), (*R*)-**PA7** (6.4 mg, 0.01 mmol), 3 Å MS (35 mg) and $Rh_2(OAc)_4$ (2.2 mg, 0.005 mmol) were dissolved in $CHCl_3$ (2 mL). A solution of diazo compound **2a** (33 mg, 0.11 mmol) in $CHCl_3$ (1 mL) was then added slowly for a period of 1 h by using syringe pump to the reaction mixture and stirred at 40 °C in a closed vessel for 12 h. The product was purified over silica gel by column chromatography (5-7% EtOAc in hexanes). The compound was obtained as a white solid (9:1 dr, 80% of isolated yield for both diastereomers, 39 mg); R_f = 0.25 (15% EtOAc-Hexane); **MP**: 222-224 °C; **^{1}H NMR** (400 MHz, $CDCl_3$) δ 7.91 – 7.94 (m, 2H), 7.54 – 7.57 (m, 1H), 7.40 – 7.49 (m, 3H), 7.21 – 7.25 (m, 1H), 7.16 (td, *J* = 7.5, 1.1 Hz, 1H), 7.00 – 7.03 (m, 3H), 6.92 – 6.94 (m, 2H), 6.89 (dt, *J* = 8.0, 1.9 Hz, 1H), 6.74 – 6.78 (m, 1H), 6.66 (dd, *J* = 8.0, 1.5 Hz, 1H), 5.84 (s, 2H), 4.90 (s, 1H), 4.24 – 4.32 (m, 2H), 1.29 (t, *J* = 7.1 Hz, 3H); **^{13}C NMR** (100 MHz, $CDCl_3$) δ 169.4, 152.9, 147.4, 146.7, 142.0, 139.4, 139.2, 133.4, 132.2, 129.7, 129.5, 129.5, 128.8, 128.2, 127.9, 126.8, 123.2, 122.7, 122.4, 122.3, 120.4, 110.5, 110.2, 107.7, 100.7, 90.0, 63.2, 61.9, 14.2; **IR** (film) υ_{max} 3073, 2976, 2894, 1757, 1725, 1605, 1503, 1485, 1442, 1368, 1297, 1243, 1212, 1128,

1098, 1042, 993, 941, 760 cm^{-1}; **HRMS** (ESI) m/z 515.1461 $[M + Na]^+$; calculated for $[C_{31}H_{24}O_6 + Na]^+$: 515.1465; Enantiomeric ratio was determined *via* HPLC analysis using a Chiralpak IE column; solvent: hexane/2-propanol = 90/10; flow rate: 1.0 mL/min; detection at 230 nm; t_R major = 7.51 min, t_R minor = 9.63 min; $[\alpha]_D^{25}$ = –176.7 (*c* = 0.43, $CHCl_3$ for 96:4 er).

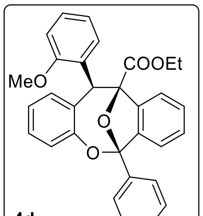

4d

Ethyl (6*R*,11*R*,12*R*)-12-(2-methoxyphenyl)-6-phenyl-6*H*-6,11-epoxydibenzo[*b*,*f*]oxocine-11(12*H*)-carboxylate (4d): A solution of **1e** (23 mg, 0.1 mmol), (*R*)-**PA7** (6.4 mg, 0.01 mmol), 3 Å MS (35 mg) and $Rh_2(OAc)_4$ (2.2 mg, 0.005 mmol) were dissolved in $CHCl_3$ (2 mL). A solution of diazo compound **2a** (33 mg, 0.11 mmol) in $CHCl_3$ (1 mL) was then added slowly for a period of 1 h by using syringe pump to the reaction mixture and stirred at 40 °C in a closed vessel for 12 h. The product was purified over silica gel by column chromatography (5-7% EtOAc in hexanes). The compound was obtained as a white solid (16:1 dr, 70% of isolated yield for both diastereomers, 33 mg); $\boldsymbol{R_f}$ = 0.25 (15% EtOAc-Hexane); **MP**: 78-80 °C; ^{1}**H NMR** (300 MHz, $CDCl_3$) *δ* 7.93 – 7.97 (m, 2H), 7.86 (dt, *J* = 7.7, 1.5 Hz, 1H), 7.71 (dt, *J* = 7.6, 1.0 Hz, 1H), 7.40 – 7.51 (m, 3H), 7.22 – 7.27 (m, 1H), 7.09 – 7.18 (m, 2H), 7.02 – 7.05 (m, 1H), 6.92 – 7.00 (m, 3H), 6.78 – 6.86 (m, 2H), 6.68 – 6.74 (m, 1H), 5.68 (s, 1H), 4.09 – 4.29 (m, 2H), 3.94 (s, 3H), 1.21 (t, *J* = 7.1 Hz, 3H); ^{13}**C NMR** (75 MHz, $CDCl_3$) δ 169.1, 155.6, 152.8, 142.6, 139.6, 139.5, 132.0, 131.8, 130.9, 129.3, 129.3, 128.7, 128.4, 128.2, 128.1, 127.6, 126.8, 123.0, 122.6, 122.1, 121.2, 121.2, 110.2, 110.0, 89.7, 61.4, 55.6, 53.5, 13.9; **IR** (film) υ_{max} 3066, 2933, 2836, 1755, 1730, 1601, 1486, 1441, 1295, 1243, 1211, 1125, 1058, 994, 759 cm^{-1}; **HRMS** (ESI) m/z 501.1659 $[M + Na]^+$; calculated for $[C_{31}H_{26}O_5 + Na]^+$: 501.1672; Enantiomeric ratio was determined *via* HPLC analysis using a Chiralpak IE column; solvent: hexane/2-propanol = 90/10; flow rate: 1.0 mL/min; detection at 230 nm; t_R major = 6.67 min, t_R minor = 9.65 min; $[\alpha]_D^{25}$ = –227.7 (*c* = 0.33, $CHCl_3$ for 96:4 er).

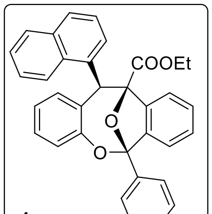

4e

Ethyl (6*R*,11*R*,12*R*)-12-(naphthalen-1-yl)-6-phenyl-6*H*-6,11-epoxydibenzo[*b*,*f*]oxocine-11(12*H*)-carboxylate (4e): A solution of **1f** (25 mg, 0.1 mmol), (*R*)-**PA7** (6.4 mg, 0.01 mmol), 3 Å MS (35 mg) and $Rh_2(OAc)_4$ (2.2 mg, 0.005 mmol) were dissolved in $CHCl_3$ (2 mL). A solution of diazo compound **2a** (33 mg, 0.11 mmol) in $CHCl_3$ (1 mL) was then added slowly for a period of 1 h by using syringe pump to the reaction mixture and stirred at 40 °C in a closed vessel for 12 h. The product was purified over silica gel by column chromatography (3-5% EtOAc in hexanes). The compound was obtained as a white solid (>20:1 dr, 69% of isolated yield for both diastereomers, 34 mg); $\boldsymbol{R_f}$ = 0.35 (10% EtOAc-Hexane); **MP**: 226-228 °C; ^{1}**H NMR** (300 MHz, $CDCl_3$) *δ* 8.60 (d, *J* = 8.7 Hz, 1H), 8.08 (dd, *J* = 7.4, 1.3 Hz, 1H), 7.96 – 8.00 (m, 2H), 7.83 (t, *J* = 7.4 Hz, 2H), 7.63 – 7.71 (m, 2H), 7.42 – 7.54 (m, 4H), 7.29 – 7.40 (m, 2H), 7.22 (td, *J* = 7.5,

1.1 Hz, 1H), 6.96 – 7.09 (m, 4H), 6.64 – 6.70 (m, 1H), 5.97 (s, 1H), 3.90 – 4.01 (m, 1H), 3.75 – 3.85 (m, 1H), 0.84 (t, *J* = 7.1 Hz, 3H); **^{13}C NMR** (100 MHz, $CDCl_3$) δ 169.2, 152.6, 142.4, 139.7, 139.4, 135.8, 133.9, 131.9, 130.9, 129.7, 129.6, 129.6, 128.9, 128.8, 128.3, 127.8, 127.6, 126.9, 126.2, 125.8, 125.0, 123.4, 123.0, 122.8, 122.4, 121.0, 110.4, 89.5, 61.7, 56.3, 13.5; **IR** (film) $υ_{max}$ 3060, 2980, 2934, 2868, 1757, 1725, 1599, 1508, 1481, 1444, 1370, 1296, 1212, 1102, 1052, 997, 858, 762 cm^{-1}; **HRMS** (ESI) m/z 521.1716 [M + Na]$^+$; calculated for $[C_{34}H_{26}O_4 + Na]^+$: 521.1723; Enantiomeric ratio was determined *via* HPLC analysis using a Chiralpak IA column; solvent: hexane/2-propanol = 95/5; flow rate: 1.0 mL/min; detection at 230 nm; t_R minor = 8.21 min, t_R major = 9.04 min; $[α]_D^{25}$ = –333.3 (*c* = 0.24, $CHCl_3$ for 95:5 er).

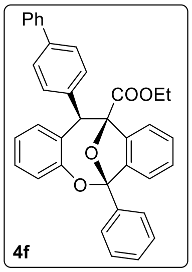

Ethyl (6*R*,11*R*,12*R*)-12-([1,1'-biphenyl]-4-yl)-6-phenyl-6*H*-6,11-epoxydibenzo[*b*,*f*]oxocine-11(12*H*)-carboxylate (4f): A solution of **1g** (28 mg, 0.1 mmol), (*R*)-**PA7** (6.4 mg, 0.01 mmol), 3 Å MS (35 mg) and $Rh_2(OAc)_4$ (2.2 mg, 0.005 mmol) were dissolved in $CHCl_3$ (2 mL). A solution of diazo compound **2a** (33 mg, 0.11 mmol) in $CHCl_3$ (1 mL) was then added slowly for a period of 1 h by using syringe pump to the reaction mixture and stirred at 40 °C in a closed vessel for 12 h. The product was purified over silica gel by column chromatography (4-6% EtOAc in hexanes). The compound was obtained as a white solid (14:1 dr, 75% of isolated yield for both diastereomers, 39 mg); ***R_f*** = 0.25 (10% EtOAc-Hexane); **MP**: 178-180 °C; **^{1}H NMR** (300 MHz, $CDCl_3$) *δ* 7.93 – 7.97 (m, 2H), 7.61 (dt, *J* = 7.6, 1.0 Hz, 1H), 7.48 – 7.56 (m, 5H), 7.35 – 7.47 (m, 6H), 7.28 – 7.32 (m, 1H), 7.23 – 7.27 (m, 1H), 7.18 (td, *J* = 7.4, 1.2 Hz, 1H), 7.02 – 7.08 (m, 2H), 6.98 (tt, *J* = 7.3, 0.9 Hz, 2H), 6.78 (ddd, *J* = 7.7, 6.3, 2.2 Hz, 1H), 5.04 (s, 1H), 4.24 – 4.31 (m, 2H), 1.26 (t, *J* = 7.1 Hz, 3H); **^{13}C NMR** (75 MHz, $CDCl_3$) δ 169.5, 153.1, 142.1, 141.0, 139.9, 139.5, 139.2, 138.6, 132.3, 130.0, 129.5, 129.5, 128.8, 128.6, 128.2, 128.0, 127.1, 127.0, 127.0, 126.8, 123.2, 122.7, 122.3, 120.5, 110.3, 89.9, 63.3, 61.9, 14.1; **IR** (film) $υ_{max}$ 3062, 2980, 1758, 1718, 1601, 1485, 1459, 1447, 1300, 1242, 1210, 1124, 1097, 1046, 985, 755 cm^{-1}; **HRMS** (ESI) m/z 547.1888 [M + Na]$^+$; calculated for $[C_{36}H_{28}O_4 + Na]^+$: 547.1880; Enantiomeric ratio was determined *via* HPLC analysis using a Chiralpak IA column; solvent: hexane/2-propanol = 98/2; flow rate: 0.25 mL/min; detection: at 250 nm): t_R minor = 52.33 min, t_R major = 62.80 min; $[α]_D^{25}$ = –222.2 (*c* = 0.27, $CHCl_3$ for 95:5 er).

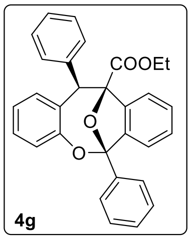

Ethyl (6*R*,11*R*,12*R*)-6,12-diphenyl-6*H*-6,11-epoxydibenzo[*b*,*f*]oxocine-11(12*H*)-carboxylate (4g): A solution of **1h** (20 mg, 0.1 mmol), (*R*)-**PA7** (6.4 mg, 0.01 mmol), 3 Å MS (35 mg) and $Rh_2(OAc)_4$ (2.2 mg, 0.005 mmol) were dissolved in $CHCl_3$ (2 mL). A solution of diazo compound **2a** (33 mg, 0.11 mmol) in $CHCl_3$ (1 mL) was then added slowly for a

period of 1 h by using syringe pump to the reaction mixture and stirred at 40 °C in a closed vessel for 12 h. The product was purified over silica gel by column chromatography (3-5% EtOAc in hexanes). The compound was obtained as a white solid (18:1 dr, 56% of isolated yield for both diastereomers, 25 mg); ***R_f*** = 0.35 (10% EtOAc-Hexane); **MP**: 153-155 °C; **^{1}H NMR** (400 MHz, $CDCl_3$) *δ* 7.96 – 7.98 (m, 2H), 7.62 (d, *J* = 7.5 Hz, 1H), 7.41 – 7.52 (m, 5H), 7.22 – 7.29 (m, 3H), 7.16 – 7.21 (m, 2H), 7.01 – 7.08 (m, 2H), 6.98 (d, *J* = 7.6 Hz, 2H), 6.73 – 6.80 (m, 1H), 5.00 (s, 1H), 4.21 – 4.33 (m, 2H), 1.26 (t, *J* = 7.1 Hz, 3H); **^{13}C NMR** (100 MHz, $CDCl_3$) δ 169.5, 153.1, 142.1, 139.6, 139.6, 139.3, 132.4, 129.6, 129.5, 129.5, 128.8, 128.3, 128.2, 127.9, 127.1, 126.8, 123.2, 122.7, 122.2, 120.5, 110.2, 89.9, 63.7, 61.8, 14.1; **IR** (film) υ_{max} 3086, 2979, 2903, 1758, 1724, 1602, 1483, 1448, 1370, 1299, 1243, 1212, 1123, 1099, 1057, 993, 764 cm^{-1}; **HRMS** (ESI) m/z 471.1555 $[M + Na]^+$; calculated for $[C_{30}H_{24}O_4 + Na]^+$: 471.1567; Enantiomeric ratio was determined *via* HPLC analysis using a Chiralpak IA column; solvent: hexane/2-propanol = 95/5; flow rate: 1.0 mL/min; detection at 230 nm; t_R major = 8.27 min, t_R minor = 9.85 min; $[\alpha]_D^{25}$ = –166.7 (*c* = 0.3, $CHCl_3$ for 95:5 er).

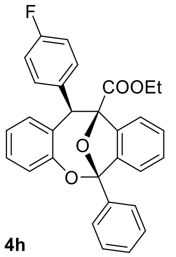

4h

Ethyl (6*R*,11*R*,12*R*)-12-(4-fluorophenyl)-6-phenyl-6*H*-6,11-epoxydibenzo[*b*,*f*]oxocine-11(12*H*)-carboxylate (4h): A solution of **1i** (22 mg, 0.1 mmol), (*R*)-**PA7** (6.4 mg, 0.01 mmol), 3 Å MS (35 mg) and $Rh_2(OAc)_4$ (2.2 mg, 0.005 mmol) were dissolved in $CHCl_3$ (2 mL). A solution of diazo compound **2a** (33 mg, 0.11 mmol) in $CHCl_3$ (1 mL) was then added slowly for a period of 1 h by using syringe pump to the reaction mixture and stirred at 40 °C in a closed vessel for 12 h. The product was purified over silica gel by column chromatography (3-5% EtOAc in hexanes). The compound was obtained as a pale yellow solid (10:1 dr, 51% of isolated yield for both diastereomers, 24 mg); ***R_f*** = 0.35 (10% EtOAc-Hexane); **MP**: 173-175 °C; **^{1}H NMR** (300 MHz, $CDCl_3$) *δ* 7.90 – 7.94 (m, 2H), 7.56 (dd, *J* = 7.4, 1.1 Hz, 1H), 7.38 – 7.51 (m, 5H), 7.20 – 7.26 (m, 1H), 7.17 (td, *J* = 7.4, 1.1 Hz, 1H), 7.00 – 7.09 (m, 2H), 6.87 – 6.99 (m, 4H), 6.73 – 6.79 (m, 1H), 4.97 (s, 1H), 4.20 – 4.30 (m, 2H), 1.25 (t, *J* = 7.1 Hz, 3H); **^{13}C NMR** (100 MHz, $CDCl_3$) δ 169.4, 162.1 (d, *J* = 243.9 Hz), 152.9, 141.9, 139.4, 139.2, 135.4 (d, *J* = 3.2 Hz), 132.2, 131.3 (d, *J* = 7.9 Hz), 129.6, 129.6, 129.5, 128.8, 128.3, 128.1, 126.7, 123.3, 122.7, 122.3, 120.4, 115.1 (d, *J* = 21.1 Hz), 110.3, 89.8, 62.7, 61.9, 14.1; **IR** (film) υ_{max} 3067, 2925, 2853, 1758, 1728, 1603, 1508, 1483, 1447, 1299, 1244, 1213, 1058, 1013, 961, 762 cm^{-1}; **HRMS** (ESI) m/z 489.1472 $[M + Na]^+$; $[C_{30}H_{23}FO_4 + Na]^+$: 489.1473; Enantiomeric ratio was determined *via* HPLC analysis using a Chiralpak IA column; solvent: hexane/2-propanol = 98/2; flow rate: 1.0 mL/min; detection at 220 nm; t_R major = 9.97 min, t_R minor = 11.10 min; $[\alpha]_D^{25}$ = –150.0 (*c* = 0.36, $CHCl_3$ for 94:6 er).

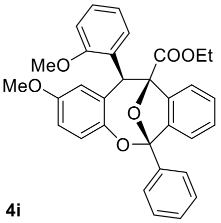

4i

Ethyl (6*R*,11*R*,12*R*)-2-methoxy-12-(2-methoxyphenyl)-6-phenyl-6H-6,11-epoxydibenzo[*b*,*f*]oxocine-11(12*H*)-carboxylate (4i): A solution of **1j** (26 mg, 0.1 mmol), (*R*)-**PA7** (6.4 mg, 0.01 mmol), 3 Å MS (35 mg) and $Rh_2(OAc)_4$ (2.2 mg, 0.005 mmol) were dissolved in $CHCl_3$ (2 mL). A solution of diazo compound **2a** (33 mg, 0.11 mmol) in $CHCl_3$ (1 mL) was then added slowly for a period of 1 h by using syringe pump to the reaction mixture and stirred at 40 °C in a closed vessel for 12 h. The product was purified over silica gel by column chromatography (8-12% EtOAc in hexanes). The compound was obtained as a white solid (>20:1 dr, 81% of isolated yield for both diastereomers, 41 mg); $\boldsymbol{R_f}$ = 0.25 (20% EtOAc-Hexane); **MP**: 96-98 °C; **^{1}H NMR** (400 MHz, $CDCl_3$) *δ* 7.92 – 7.95 (m, 2H), 7.90 (dd, *J* = 7.7, 1.7 Hz, 1H), 7.70 (dt, *J* = 7.6, 0.9 Hz, 1H), 7.39 – 7.49 (m, 3H), 7.23 – 7.27 (m, 1H), 7.11 – 7.18 (m, 2H), 6.93 (dt, *J* = 7.5, 1.0 Hz, 1H), 6.90 (d, *J* = 8.8 Hz, 1H), 6.80 – 6.85 (m, 2H), 6.57 (d, *J* = 3.1 Hz, 1H), 6.51 (dd, *J* = 8.8, 3.1 Hz, 1H), 5.60 (s, 1H), 4.11 – 4.27 (m, 2H), 3.94 (s, 3H), 3.59 (s, 3H), 1.20 (t, *J* = 7.1 Hz, 3H); **^{13}C NMR** (100 MHz, $CDCl_3$) δ 169.1, 155.6, 154.6, 146.7, 142.5, 139.8, 139.5, 131.8, 131.8, 129.3, 129.2, 128.6, 128.2, 128.2, 128.1, 126.8, 122.8, 122.6, 121.2, 121.2, 116.5, 112.8, 110.2, 110.0, 89.6, 61.4, 55.6, 55.1, 53.3, 13.9; **IR** (film) υ_{max} 2935, 2904, 2835, 1756, 1732, 1609, 1490, 1460, 1292, 1242, 1206, 1122, 1056, 1009, 962, 754 cm^{-1}; **HRMS** (ESI) m/z 531.1778 $[M + Na]^+$; calculated for $[C_{32}H_{28}O_6 + Na]^+$: 531.1778; Enantiomeric ratio was determined *via* HPLC analysis using a Chiralpak IA column; solvent: hexane/2-propanol = 90/10; flow rate: 1.0 mL/min; detection at 230 nm; t_R major = 7.36 min, t_R minor = 14.32 min; $[\alpha]_D^{25}$ = –220.5 (*c* = 0.39, $CHCl_3$ for 96:4 er).

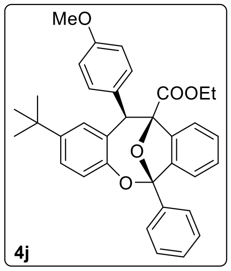

4j

Ethyl (6*R*,11*R*,12*R*)-2-(*tert*-butyl)-12-(4-methoxyphenyl)-6-phenyl-6*H*-6,11-epoxydibenzo[*b*,*f*]oxocine-11(12*H*)-carboxylate (4j): A solution of **1k** (29 mg, 0.1 mmol), (*R*)-**PA7** (6.4 mg, 0.01 mmol), 3 Å MS (35 mg) and $Rh_2(OAc)_4$ (2.2 mg, 0.005 mmol) were dissolved in $CHCl_3$ (2 mL). A solution of diazo compound **2a** (33 mg, 0.11 mmol) in $CHCl_3$ (1 mL) was then added slowly for a period of 1 h by using syringe pump to the reaction mixture and stirred at 40 °C in a closed vessel for 12 h. The product was purified over silica gel by column chromatography (4-6% EtOAc in hexanes). The compound was obtained as a white solid (11:1 dr, 89% of isolated yield for both diastereomers, 47 mg); $\boldsymbol{R_f}$ = 0.35 (15% EtOAc-Hexane); **MP**: 86-88 °C; **^{1}H NMR** (400 MHz, $CDCl_3$) *δ* 7.94 – 7.96 (m, 2H), 7.59 (d, *J* = 7.5 Hz, 1H), 7.43 – 7.50 (m, 3H), 7.39 – 7.42 (m, 2H), 7.24 (td, *J* = 7.5, 1.2 Hz, 1H), 7.17 (td, *J* = 7.4, 1.1 Hz, 1H), 7.00 – 7.03 (m, 1H), 6.94 (d, *J* = 8.7 Hz, 2H), 6.90 (d, *J* = 2.4 Hz, 1H), 6.76 – 6.80 (m, 2H), 4.92 (s, 1H), 4.28 (q, *J* = 7.1 Hz, 2H), 3.75 (s, 3H), 1.29 (t, *J* = 7.1 Hz, 3H), 1.14 (s, 9H); **^{13}C NMR** (100 MHz, $CDCl_3$) δ 169.7, 158.5, 150.6, 145.6, 142.1, 139.7, 139.5, 132.1, 130.7, 129.4, 129.2, 129.0, 128.8, 128.7, 128.2, 126.8, 124.9, 122.7, 121.5, 120.5, 113.6, 110.1, 90.2, 63.0, 61.8, 55.1, 33.9, 31.2, 14.2; **IR** (film) υ_{max} 2962, 2867, 2834, 1759, 1726, 1611, 1510, 1460, 1302, 1250, 1215, 1179, 1130, 1055, 1009, 962, 761 cm^{-1}; **HRMS** (ESI) m/z 557.2296 $[M + Na]^+$; calculated for $[C_{35}H_{34}O_5 + Na]^+$: 557.2298; Enantiomeric ratio was determined *via* HPLC analysis using a Chiralpak IA column; solvent: hexane/2-propanol = 95/5; flow rate: 1.0 mL/min; detection at 230 nm; t_R minor = 7.07 min, t_R major = 7.83 min; $[\alpha]_D^{25}$ = –166.7 (*c* = 0.42, $CHCl_3$ for 95:5 er).

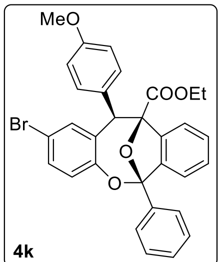

4k

Ethyl (6*R*,11*R*,12*R*)-2-bromo-12-(4-methoxyphenyl)-6-phenyl-6*H*-6,11-epoxydibenzo[*b*,*f*]oxocine-11(12*H*)-carboxylate (4k): A solution of **1l** (31 mg, 0.1 mmol), (*R*)-**PA7** (6.4 mg, 0.01 mmol), 3 Å MS (35 mg) and $Rh_2(OAc)_4$ (2.2 mg, 0.005 mmol) were dissolved in $CHCl_3$ (2 mL). A solution of diazo compound **2a** (33 mg, 0.11 mmol) in $CHCl_3$ (1 mL) was then added slowly for a period of 1 h by using syringe pump to the reaction mixture and stirred at 40 °C in a closed vessel for 12 h. The product was purified over silica gel by column chromatography (5-7% EtOAc in hexanes). The compound was obtained as a white solid (10:1 dr, 80% of isolated yield for both diastereomers, 45 mg); R_f = 0.25 (15% EtOAc-Hexane); **MP**: 186-188 °C; **^{1}H NMR** (400 MHz, $CDCl_3$) δ 7.92 – 7.94 (m, 2H), 7.61 (dt, *J* = 7.5, 0.9 Hz, 1H), 7.45 – 7.52 (m, 3H), 7.36 – 7.40 (m, 2H), 7.28 – 7.32 (m, 1H), 7.22 (td, *J* = 7.5, 1.1 Hz, 1H), 7.09 – 7.13 (m, 2H), 6.96 – 6.99 (m, 1H), 6.93 (d, *J* = 8.4 Hz, 1H), 6.78 – 6.82 (m, 2H), 4.90 (s, 1H), 4.24 – 4.31 (m, 2H), 3.76 (s, 3H), 1.28 (t, *J* = 7.1 Hz, 3H); **^{13}C NMR** (100 MHz, $CDCl_3$) δ 169.1, 158.9, 152.2, 141.8, 139.2, 138.9, 134.7, 132.0, 131.1, 130.8, 130.6, 129.8, 129.7, 128.9, 128.3, 126.7, 124.1, 122.7, 120.6, 115.5, 113.8, 110.4, 89.9, 62.5, 61.9, 55.1, 14.2; **IR** (film) υ_{max} 3065, 2932, 2835, 1757, 1726, 1611, 1510, 1473, 1303, 1247, 1213, 1179, 1123, 1056, 1012, 824, 761 cm^{-1}; **HRMS** (ESI) m/z 579.0770 $[M + Na]^+$; calculated for $[C_{31}H_{25}{}^{79}BrO_5 + Na]^+$: 579.0778; Enantiomeric ratio was determined *via* HPLC analysis using a Chiralpak IE column; solvent: hexane/2-propanol = 95/5; flow rate: 1.0 mL/min; detection at 245 nm; t_R major = 8.04 min, t_R minor = 9.59 min; $[\alpha]_D^{25}$ = –125.7 (*c* = 0.35, $CHCl_3$ for 91:9 er).

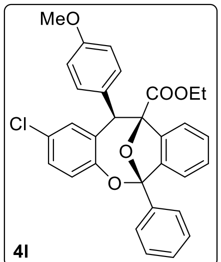

4l

Ethyl (6*R*,11*R*,12*R*)-2-chloro-12-(4-methoxyphenyl)-6-phenyl-6*H*-6,11-epoxydibenzo[*b*,*f*]oxocine-11(12*H*)-carboxylate (4l): A solution of **1m** (27 mg, 0.1 mmol), (*R*)-**PA7** (6.4 mg, 0.01 mmol), 3 Å MS (35 mg) and $Rh_2(OAc)_4$ (2.2 mg, 0.005 mmol) were dissolved in $CHCl_3$ (2 mL). A solution of diazo compound **2a** (33 mg, 0.11 mmol) in $CHCl_3$ (1 mL) was then added slowly for a period of 1 h by using syringe pump to the reaction mixture and stirred at 40 °C in a closed vessel for 12 h. The product was purified over silica gel by column chromatography (5-7% EtOAc in hexanes). The compound was obtained as a white solid (10:1 dr, 85% of isolated yield for both diastereomers, 44 mg); R_f = 0.25 (15% EtOAc-Hexane); **MP**: 84-86 °C; **^{1}H NMR** (400 MHz, $CDCl_3$) δ 7.94 (dd, *J* = 8.2, 1.6 Hz, 2H), 7.61 (dt, *J* = 7.6, 0.9 Hz, 1H), 7.43 – 7.52 (m, 3H), 7.37 – 7.41 (m, 2H), 7.29 – 7.32 (m, 1H), 7.22 (td, *J* = 7.4, 1.1 Hz, 1H), 6.95 – 6.99 (m, 4H), 6.81 (d, *J* = 8.8 Hz, 2H), 4.91 (s, 1H), 4.29 (qd, *J* = 7.1, 1.4 Hz, 2H), 3.76 (s, 3H), 1.29 (t, *J* = 7.1 Hz, 3H); **^{13}C NMR** (100 MHz, $CDCl_3$) δ 169.2, 158.9, 151.7, 141.9, 139.2, 138.9, 131.8, 131.6, 131.1, 130.6, 129.8, 129.7, 128.9, 128.3, 127.9, 127.8, 126.7, 123.7, 122.7, 120.5, 113.8, 110.4, 89.9, 62.5, 61.9, 55.1, 14.2; **IR** (film) υ_{max} 2980, 2933, 2835, 1758, 1727, 1610, 1510, 1475, 1395, 1303, 1247, 1212, 1179, 1123, 1056, 961, 762 cm^{-1}; **HRMS** (ESI) m/z 535.1285

[M + Na]$^+$; calculated for [$C_{31}H_{25}ClO_5$ + Na]$^+$: 535.1283; Enantiomeric ratio was determined *via* HPLC analysis using a Chiralpak IE column; solvent: hexane/2-propanol = 90/10; flow rate: 1.0 mL/min; detection at 230 nm; t_R major = 6.43 min, t_R minor = 7.32 min; $[\alpha]_D^{25}$ = –141.9 (*c* = 0.31, $CHCl_3$ for 91:9 er).

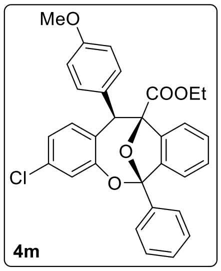

Ethyl (6*R*,11*R*,12*R*)-3-chloro-12-(4-methoxyphenyl)-6-phenyl-6*H*-6,11-epoxydibenzo[*b*,*f*]oxocine-11(12*H*)-carboxylate (4m): A solution of **1n** (27 mg, 0.1 mmol), (*R*)-**PA7** (6.4 mg, 0.01 mmol), 3 Å MS (35 mg) and $Rh_2(OAc)_4$ (2.2 mg, 0.005 mmol) were dissolved in $CHCl_3$ (2 mL). A solution of diazo compound **2a** (33 mg, 0.11 mmol) in $CHCl_3$ (1 mL) was then added slowly for a period of 1 h by using syringe pump to the reaction mixture and stirred at 40 °C in a closed vessel for 12 h. The product was purified over silica gel by column chromatography (5-7% EtOAc in hexanes). The compound was obtained as a white solid (9:1 dr, 88% of isolated yield for both diastereomers, 45 mg); $\boldsymbol{R_f}$ = 0.25 (15% EtOAc-Hexane); **MP**: 85-87 °C; **^{1}H NMR** (300 MHz, $CDCl_3$) *δ* 7.89 – 7.92 (m, 2H), 7.56 – 7.59 (m, 1H), 7.42 – 7.50 (m, 3H), 7.33 – 7.36 (m, 2H), 7.24 – 7.29 (m, 1H), 7.20 (td, *J* = 7.4, 1.3 Hz, 1H), 7.05 (dd, *J* = 2.2, 0.8 Hz, 1H), 6.95 – 6.98 (m, 1H), 6.87 (d, *J* = 8.3 Hz, 1H), 6.71 – 6.78 (m, 3H), 4.92 (s, 1H), 4.25 (q, *J* = 7.1 Hz, 2H), 3.72 (s, 3H), 1.26 (t, *J* = 7.1 Hz, 3H); **^{13}C NMR** (75 MHz, $CDCl_3$) δ 169.2, 158.8, 153.6, 141.9, 139.1, 138.9, 133.3, 132.9, 131.3, 130.5, 129.7, 129.7, 128.9, 128.6, 128.3, 126.7, 123.4, 122.8, 122.2, 120.4, 113.8, 110.5, 90.0, 62.5, 61.9, 55.1, 14.1; **IR** (film) υ_{max} 2929, 2835, 1758, 1729, 1609, 1510, 1481, 1449, 1397, 1303, 1247, 1211, 1179, 1132, 1057, 961, 762 cm^{-1}; **HRMS** (ESI) m/z 535.1280 [M + Na]$^+$; calculated for [$C_{31}H_{25}ClO_5$ + Na]$^+$: 535.1283; Enantiomeric ratio was determined *via* HPLC analysis using a Chiralpak IA column; solvent: hexane/2-propanol = 95/5; flow rate: 1.0 mL/min; detection at 230 nm; t_R minor = 15.72 min, t_R major = 21.36 min; $[\alpha]_D^{25}$ = –133.3 (*c* = 0.27, $CHCl_3$ for 92:8 er).

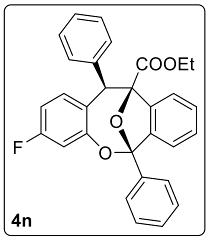

Ethyl (6*R*,11*R*,12*R*)-3-fluoro-6,12-diphenyl-6*H*-6,11-epoxydibenzo[*b*,*f*]oxocine-11(12*H*)-carboxylate (4n): A solution of **1o** (22 mg, 0.1 mmol), (*R*)-**PA7** (6.4 mg, 0.01 mmol), 3 Å MS (35 mg) and $Rh_2(OAc)_4$ (2.2 mg, 0.005 mmol) were dissolved in $CHCl_3$ (2 mL). A solution of diazo compound **2a** (33 mg, 0.11 mmol) in $CHCl_3$ (1 mL) was then added slowly for a period of 1 h by using syringe pump to the reaction mixture and stirred at 40 °C in a closed vessel for 12 h. The product was purified over silica gel by column chromatography (3-6% EtOAc in hexanes). The compound was obtained as a white solid (18:1 dr, 73% of isolated yield for both diastereomers, 34 mg); $\boldsymbol{R_f}$ = 0.35 (10% EtOAc-Hexane); **MP**: 176-178 °C; **^{1}H NMR** (300 MHz, $CDCl_3$) *δ* 7.88 – 7.92 (m, 2H), 7.60 (ddd, *J* = 7.5, 1.2, 0.7 Hz, 1H), 7.39 – 7.51 (m, 5H), 7.14 – 7.30 (m, 5H), 6.98 (ddd, *J* = 7.4, 1.2, 0.7 Hz, 1H), 6.87 – 6.92 (m, 1H), 6.75 (dd, *J* = 9.9, 2.7 Hz, 1H), 6.48

(ddd, J = 8.6, 7.5, 2.7 Hz, 1H), 4.96 (s, 1H), 4.24 (qd, J = 7.1, 2.9 Hz, 2H), 1.23 (t, J = 7.1 Hz, 3H); **^{13}C NMR** (100 MHz, $CDCl_3$) δ 169.3, 161.8 (d, J = 244.7 Hz), 154.3, 154.2, 142.0, 139.4, 139.2, 138.9, 133.5 (d, J = 9.5 Hz), 129.7, 129.5, 128.9, 128.3 (d, J = 3.3 Hz), 127.3, 126.7, 125.7 (d, J = 3.6 Hz), 122.8, 120.5, 110.6 (d, J = 9.0 Hz), 110.3, 109.2 (d, J = 23.2 Hz), 90.0, 63.1, 61.9, 14.1; **IR** (film) $υ_{max}$ 3065, 2929, 2835, 1759, 1729, 1614, 1496, 1370, 1295, 1216, 1150, 1098, 1056, 998, 858, 750 cm^{-1}; **HRMS** (ESI) m/z 489.1470 [M + Na]$^+$; calculated for [$C_{30}H_{23}FO_4$ + Na]$^+$: 489.1473; Enantiomeric ratio was determined *via* HPLC analysis using a Chiralpak IE column; solvent: hexane/2-propanol = 95/5; flow rate: 1.0 mL/min; detection at 230 nm; t_R major = 5.49 min, t_R minor = 6.09 min; $[α]_D^{25}$ = –152.7 (c = 0.55, $CHCl_3$ for 93:7 er).

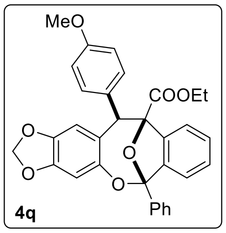

4q

Ethyl 12-(4-methoxyphenyl)-6-phenyl-6*H*-6,11-epoxy[1,3]dioxolo[4',5':4,5]benzo[1,2-*b*]benzo[*f*]oxocine-11(12*H*)-carboxylate (4q): A solution of **1q** (26 mg, 0.1 mmol), and Rh_2(*S*-DOSP)$_4$ (9.5 mg, 0.005 mmol) were dissolved in $CHCl_3$ (2 mL). A solution of diazo compound **2a** (33 mg, 0.11 mmol) in $CHCl_3$ (1 mL) was then added slowly for a period of 1 h by using syringe pump to the reaction mixture and stirred at rt in a closed vessel for 12 h. The product was purified over silica gel by column chromatography (5-7% EtOAc in hexanes). The compound was obtained as a colorless oil (5:1 dr, 15% of isolated yield for both diastereomers, 8 mg); ***R**$_f$* = 0.35 (15% EtOAc-Hexane); **^{1}H NMR** (400 MHz, $CDCl_3$) δ 7.89 – 7.92 (m, 2H), 7.58 (dt, J = 7.6, 0.9 Hz, 1H), 7.44 – 7.48 (m, 2H), 7.38 – 7.42 (m, 2H), 7.27 – 7.31 (m, 2H), 7.20 – 7.24 (m, 1H), 6.95 – 6.99 (m, 1H), 6.77 – 6.80 (m, 2H), 6.53 (s, 1H), 6.36 (s, 1H), 5.81 (d, J = 1.5 Hz, 1H), 5.78 (d, J = 1.5 Hz, 1H), 4.78 (s, 1H), 4.27 (qd, J = 7.1, 0.8 Hz, 2H), 3.75 (s, 3H), 1.27 (t, J = 7.1 Hz, 3H); **^{13}C NMR** (100 MHz, $CDCl_3$) δ 169.5, 158.7, 147.6, 146.9, 143.3, 142.2, 139.4, 139.2, 131.7, 130.6, 129.5, 129.4, 128.7, 128.2, 126.7, 122.7, 122.3, 120.3, 113.7, 110.3, 110.2, 103.1, 101.3, 90.1, 62.5, 61.8, 55.1, 14.1; **IR** (film) $υ_{max}$ 3438, 2925, 2853, 1728, 1609, 1509, 1483, 1301, 1213, 1180, 1036, 938, 759 cm^{-1}; **HRMS** (ESI) m/z 545.1575 [M + Na]$^+$; calculated for [$C_{32}H_{26}O_7$ + Na]$^+$: 545.1571; Enantiomeric ratio was determined *via* HPLC analysis using a Chiralpak IA column; solvent: hexane/2-propanol = 90/10; flow rate: 1.0 mL/min; detection at 211 nm; $t_{R(1)}$ = 12.58 min, $t_{R(2)}$ = 24.12 min; 50:50 er.

Procedure for the synthesis of compound 5:

A solution of enantioenriched compound **3a** (1.0 equiv, 0.167 mmol) in CH_2Cl_2 (5 mL) was cooled to 0°C using ice-water mixture. Allyl tributylstannane (2.0 equiv) and $BF_3{\cdot}OEt_2$ (1.5 equiv.) were sequentially added to the reaction mixture and allowed to stir under an Ar atmosphere at the same temperature for 30 min. Upon completion of the reaction (monitored by TLC), the reaction mixture was quenched by addition of aq. $NaHCO_3$ solution and extracted with CH_2Cl_2 (3 x 20 mL). The organic layers were dried over Na_2SO_4, filtered, and concentrated under reduced pressure. The residue was purified over silica gel by flash chromatography to obtain compound **5** as a white solid (80% yield).

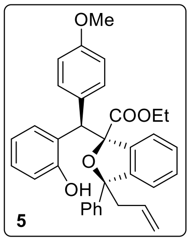

Ethyl (1*R*,3*S*)-3-allyl-1-((*R*)-(2-hydroxyphenyl)(4-methoxyphenyl)methyl)-3-phenyl-1,3-dihydroisobenzofuran-1-carboxylate (5): The compound **5** was obtained as a white solid (>20:1 dr, 80% yield, 70 mg); ***R**$_f$* = 0..25 (20% EtOAc-Hexane); **MP**: 68-70 °C; **^{1}H NMR** (400 MHz, $CDCl_3$) *δ* 7.77 (d, *J* = 7.7 Hz, 1H), 7.49 (d, *J* = 8.4 Hz, 2H), 7.42 (td, *J* = 7.5, 1.1 Hz, 1H), 7.33 (dd, *J* = 7.5, 1.2 Hz, 1H), 7.25 – 7.28 (m, 1H), 7.20 (t, *J* = 7.6 Hz, 2H), 7.08 – 7.12 (m, 3H), 6.79 – 6.83 (m, 3H), 6.73 (td, *J* = 7.6, 1.7 Hz, 1H), 6.29 (td, *J* = 7.4, 1.3 Hz, 1H), 6.11 (dd, *J* = 8.0, 1.3 Hz, 1H), 5.94 – 5.96 (m, 1H), 5.62 – 5.72 (m, 1H), 5.25 (brs, 1H), 4.99 – 5.02 (m, 1H), 4.92 – 4.98 (m, 1H), 4.02 – 4.13 (m, 2H), 3.76 (s, 3H), 3.57 – 3.63 (m, 1H), 3.03 (dd, *J* = 14.2, 9.0 Hz, 1H), 1.06 (t, *J* = 7.1 Hz, 3H); **^{13}C NMR** (100 MHz, $CDCl_3$) δ 172.4, 157.9, 154.0, 141.9, 140.6, 139.4, 133.0, 132.6, 132.3, 130.1, 128.1, 128.0, 127.9, 127.7, 127.6, 127.3, 126.1, 124.3, 123.7, 119.5, 118.8, 117.6, 113.4, 94.4, 93.5, 61.5, 55.1, 54.7, 46.5, 13.8; **IR** (film) $υ_{max}$ 3426, 1746, 1724, 1608, 1510, 1455, 1247, 1182, 1095, 1030, 915, 754 cm^{-1}; **HRMS** (ESI) m/z 543.2133 $[M + Na]^+$; calculated for $[C_{34}H_{32}O_5 + Na]^+$: 543.2142; Enantiomeric ratio was determined *via* HPLC analysis using a Chiralpak IA column; solvent: hexane/2-propanol = 90/10; flow rate: 1.0 mL/min; detection at 230 nm; t_R major = 9.41 min, t_R minor = 10.36 min; $[α]_D^{25}$ = +152.4 (*c* = 0.21, $CHCl_3$ for 98:2 er).

Procedure for the synthesis of compound 6:

The compound **5** (0.12 mmol, 1.0 equiv) was dissolved in $CHCl_3$ (2 mL) and *p*-TsOH.H_2O (1.0 equiv) was added to it. The reaction mixture was stirred at rt for 2 h. The solvent was removed *in vacuo* and residue was purified by flash column chromatography using EtOAc/Hexane as eluent to afford product **6** as a white solid (90% yield).

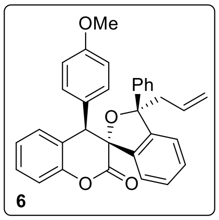

(3*R*,3'*S*,4*R*)-3'-Allyl-4-(4-methoxyphenyl)-3'-phenyl-3'*H*-spiro[chromane-3,1'-isobenzofuran]-2-one (6): The compound **6** was obtained as a white solid (90% yield, 51 mg); ***R**$_f$* = 0.3 (15% EtOAc-Hexane); **MP**: 66-68 °C; **^{1}H NMR** (400 MHz, $CDCl_3$) *δ* 7.38 – 7.43 (m, 3H), 7.23 – 7.231 (m, 6H), 7.13 – 7.17 (m, 2H), 6.97 (dt, *J* = 7.7, 1.1 Hz, 1H), 6.71 (d, *J* = 8.7 Hz, 2H), 6.63 (d, *J* = 8.8 Hz, 2H), 6.27 (d, *J* = 7.8 Hz, 1H), 5.45 – 5.55 (m, 1H), 4.93 – 4.99 (m, 2H), 4.22 (s, 1H), 3.76 (s, 3H), 2.85 – 2.95 (m, 2H); **^{13}C NMR** (100 MHz, $CDCl_3$) δ 166.5, 159.1, 151.2, 144.1, 144.0, 136.2, 132.7, 130.7, 129.3, 128.8, 128.7, 128.0, 127.4, 127.3, 127.0, 125.7, 125.4, 125.1, 124.7, 123.0, 118.6, 116.7, 113.8, 91.8, 87.9, 55.1, 53.3, 47.6; **IR** (film) $υ_{max}$ 3073, 2927, 1777, 1611, 1512, 1486, 1456, 1252, 1156, 1005, 757 cm^{-1}; **HRMS** (ESI) m/z 497.1736 $[M + Na]^+$; calculated for $[C_{32}H_{26}O_4 + Na]^+$: 497.1723; Enantiomeric ratio was determined *via* HPLC analysis using a Chiralpak IE column; solvent: hexane/2-propanol = 90/10; flow rate: 1.0 mL/min; detection at 230 nm; t_R major = 11.59 min, t_R minor = 13.47 min; $[α]_D^{25}$ = –250.0 (*c* = 0.32, $CHCl_3$ for 98:2 er).

Procedure for the synthesis of compound 3f: Suzuki-Miyaura Cross-Coupling Reaction[1e]

The bromo-substituted compound **3i** (0.1 mmol, 1.0 equiv.), phenylboronic acid (0.2 mmol, 2.0 equiv.), anhydrous Na_2CO_3 (0.6 mmol, 6.0 equiv.) and $Pd(PPh_3)_4$ (0.005 mmol, 5 mol%) were suspended in a degassed solvent mixture of toluene/H_2O/EtOH (5:3:1 v/v/v) in a round bottom flask-fitted with a reflux condensor under Ar-atmosphere. The reaction mixture was refluxed at 90 °C until complete consumption of the starting material was indicated by TLC. The crude reaction mixture was directly purified by flash chromatography to deliver the desired compound **3f** as a white solid (89% yield). The enantiomeric excess was determined by HPLC on chiral stationary phase.

Note: For spectral data; see compound **3f** on page S16.

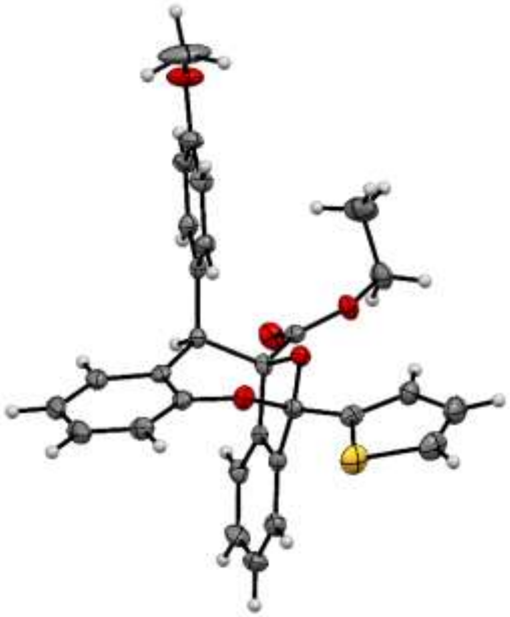

Figure 2. X-ray crystal structure of compound **(6*S*,11*R*,12*R*)-3k** (CCDC 1946746).

References:

1. (a) O. El-Sepelgy, S. Haseloff, S. K. Alamsetti, C. Schneider, *Angew. Chem. Int. Ed.* **2014**, *53*, 7923; (b) C. C. Hsiao, H. H. Liao, M. Rueping, *Angew. Chem., Int. Ed.* **2014**, *53*, 13258; (c) S. K. Alamsetti, M. Spanka, C. Schneider, *Angew. Chem. Int. Ed.* **2016**, *55*, 2392; (d) M. Spanka, C. Schneider, *Org. Lett.* **2018**, *20*, 4769; (e) F. Göricke, C. Schneider, *Angew. Chem. Int. Ed.* **2018**, *57*, 14736; (f) A. Suneja, C. Schneider, *Org. Lett.* **2018**, *20*, 7576.

2. (a) H. B. Abed, O. Bande, O. Mammoliti, G. V. Lommen, P. Herdewijn, *Tetrahedron Lett.* **2013**, *54*, 7056; (b) K. Kobayashi, T. Mannami, M. Kawakita, J. Tokimatsu, H. Konishi, *Bull. Chem. Soc. Jpn.* **1994**, *67*, 582.

Spectral Graphics

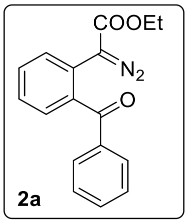

2a

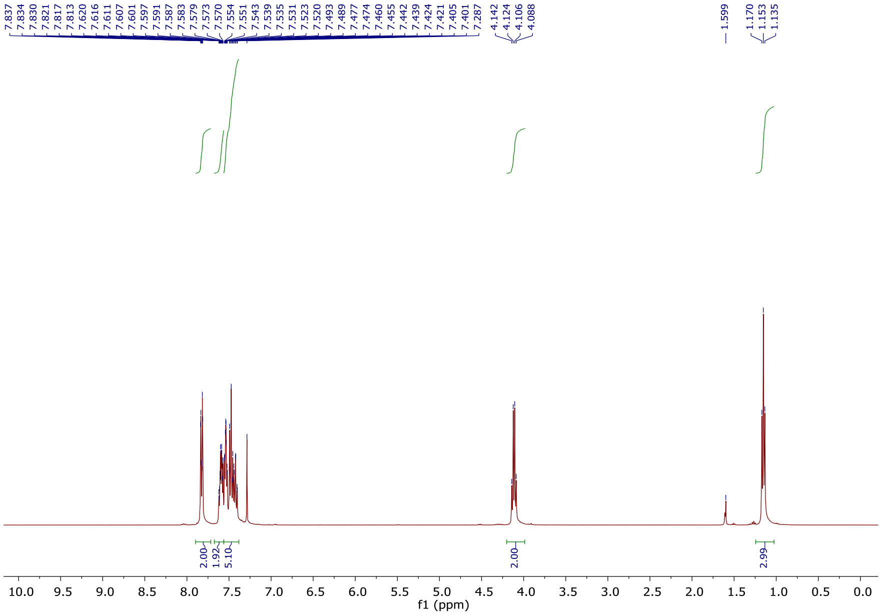

^{1}H NMR (400 MHz, $CDCl_3$) of compound **2a**

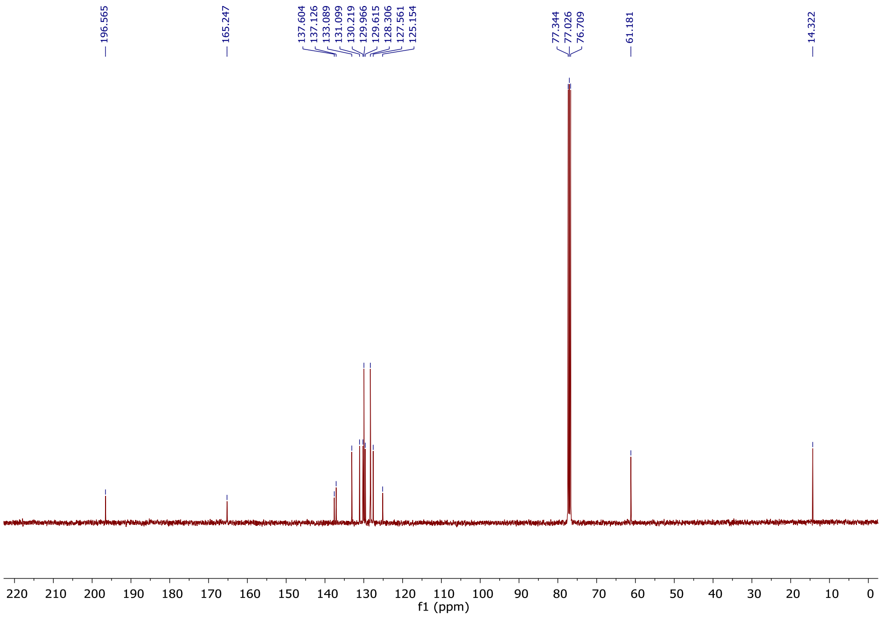

^{13}C NMR (100 MHz, $CDCl_3$) of compound **2a**

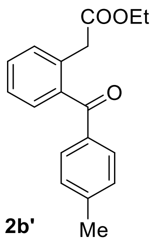

2b'

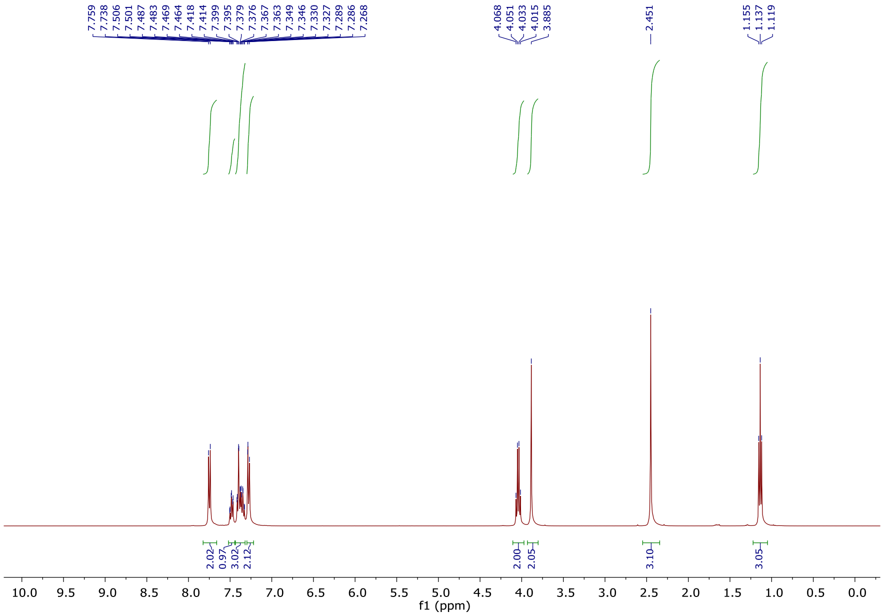

^{1}H NMR (400 MHz, $CDCl_3$) of compound **2b'**

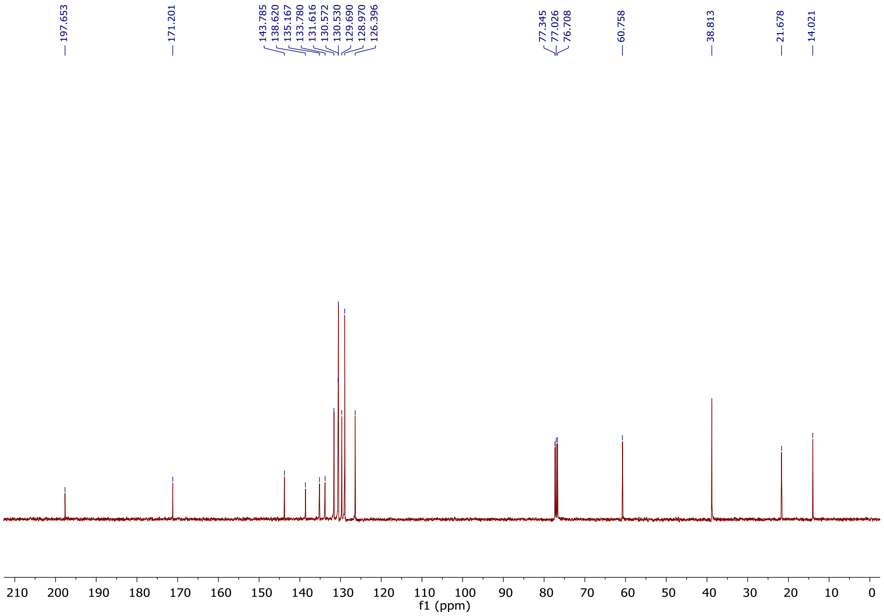

^{13}C NMR (100 MHz, $CDCl_3$) of compound **2b'**

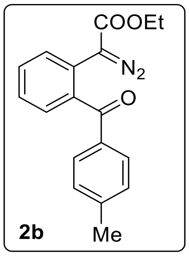

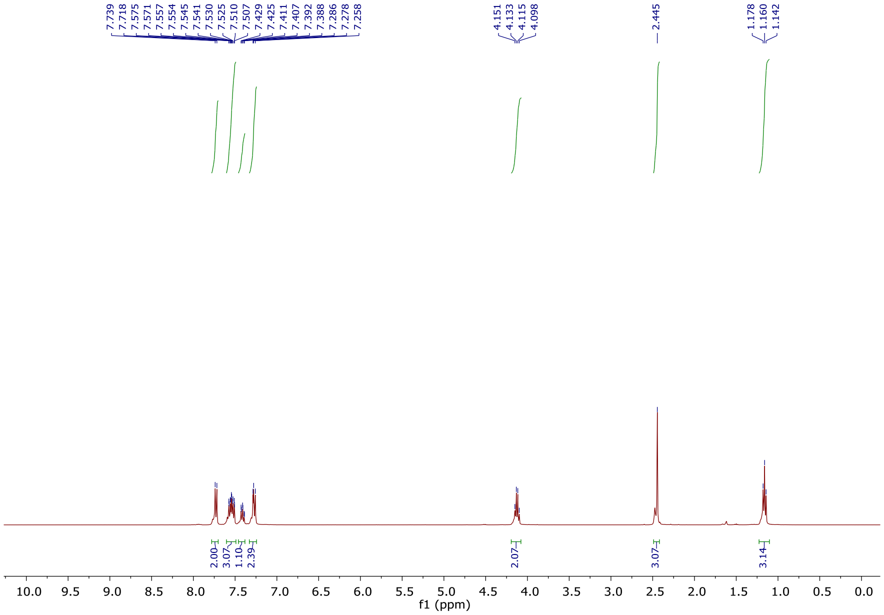

^{1}H NMR (400 MHz, $CDCl_3$) of compound **2b**

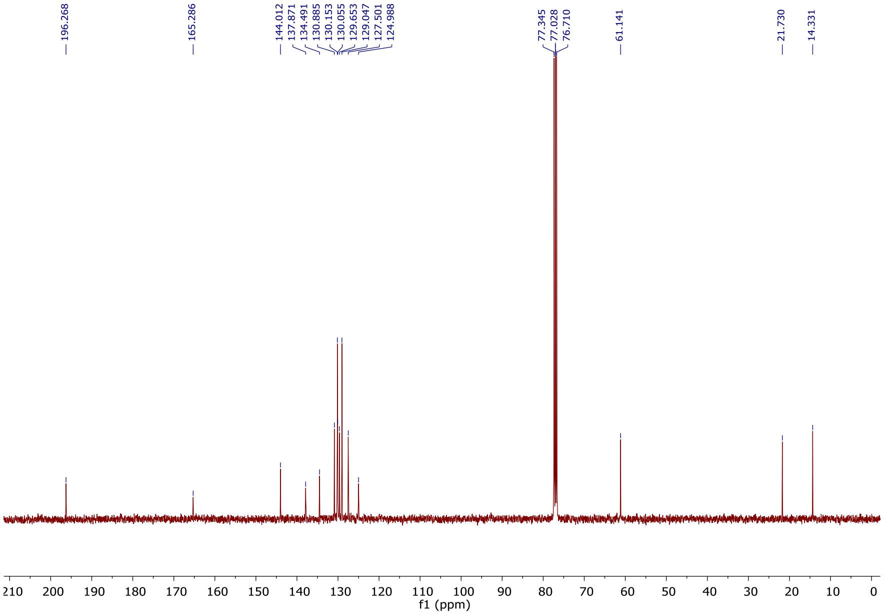

^{13}C NMR (100 MHz, $CDCl_3$) of compound **2b**

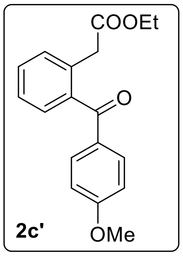

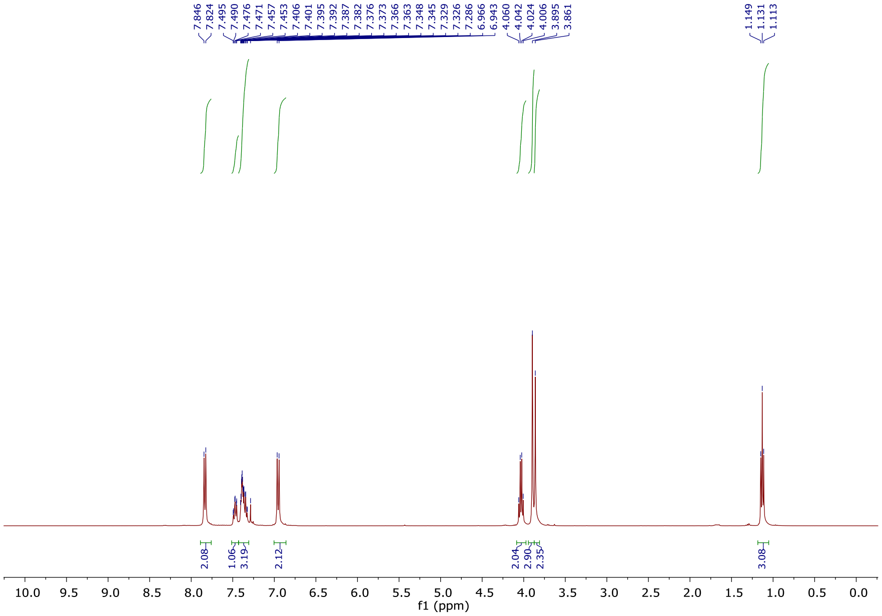

^{1}H NMR (400 MHz, $CDCl_3$) of compound **2c'**

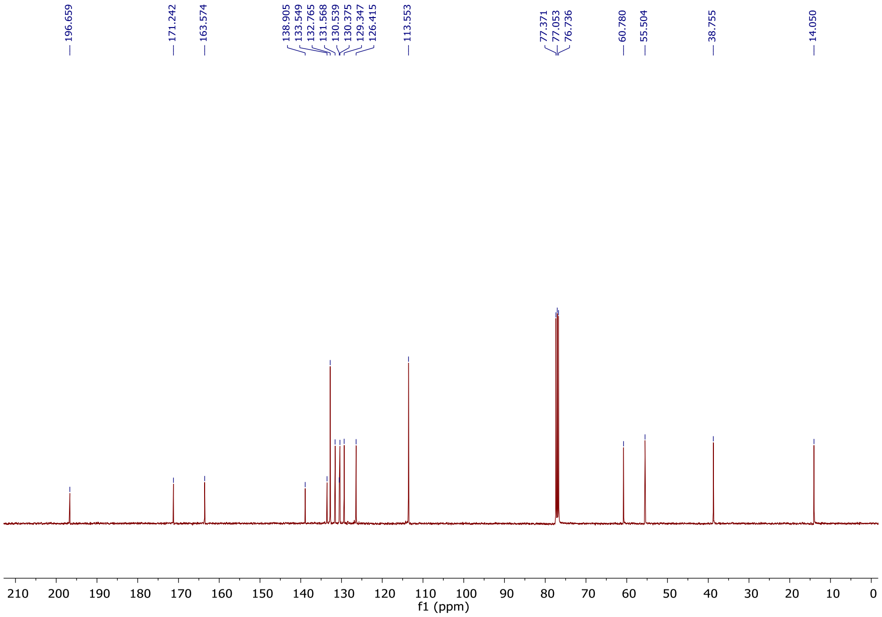

^{13}C NMR (100 MHz, $CDCl_3$) of compound **2c'**

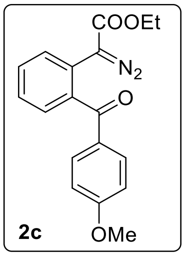

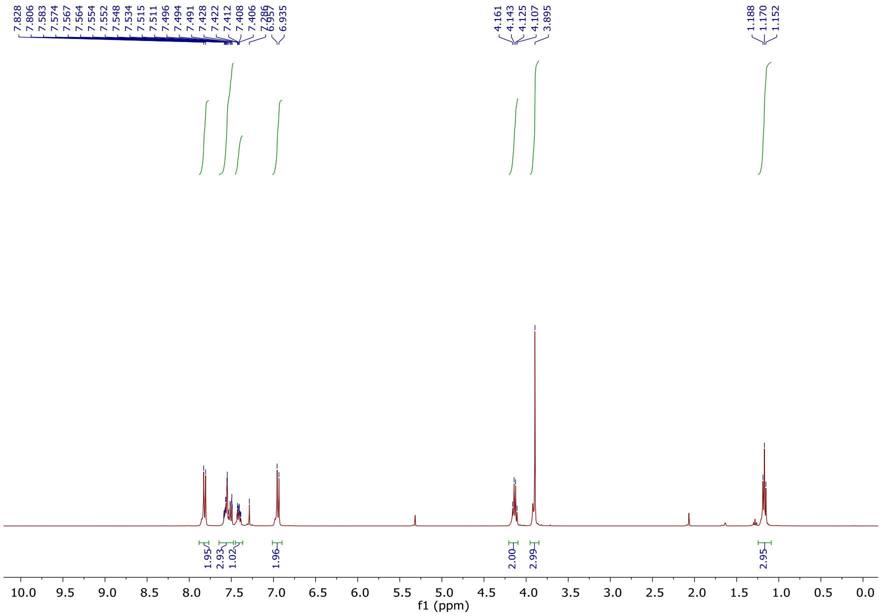

^{1}H NMR (400 MHz, $CDCl_3$) of compound **2c**

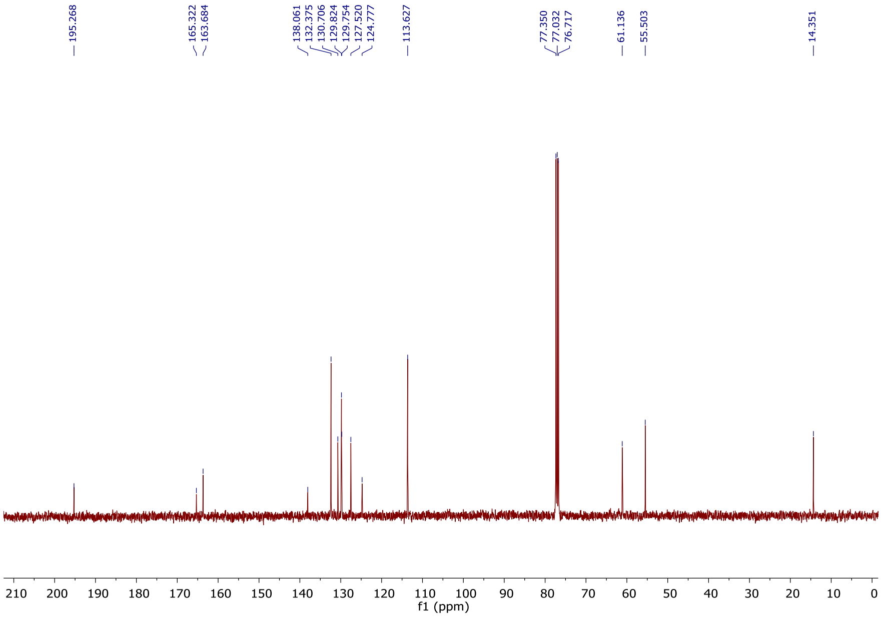

^{13}C NMR (100 MHz, $CDCl_3$) of compound **2c**

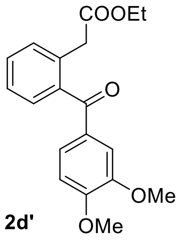

2d'

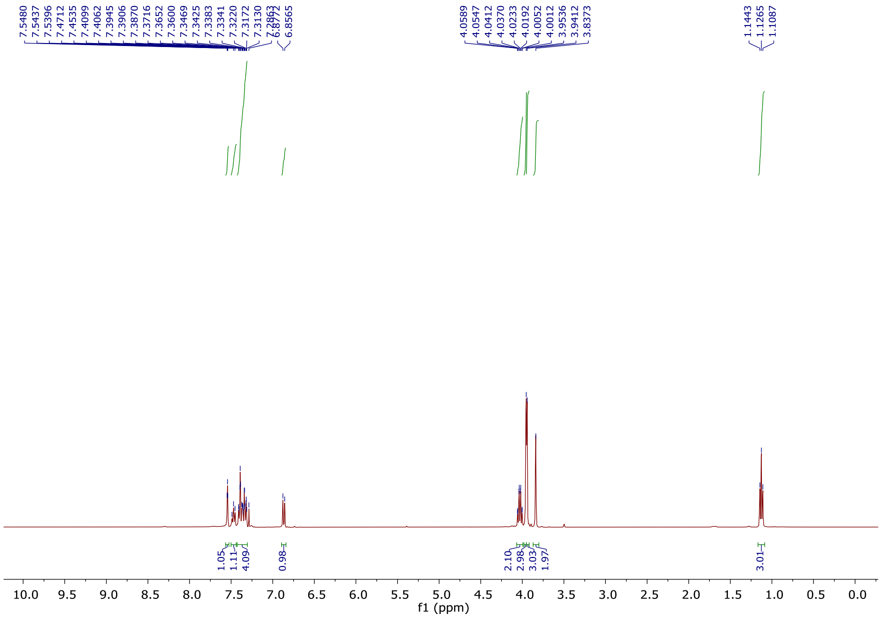

^{1}H NMR (400 MHz, $CDCl_3$) of compound **2d'**

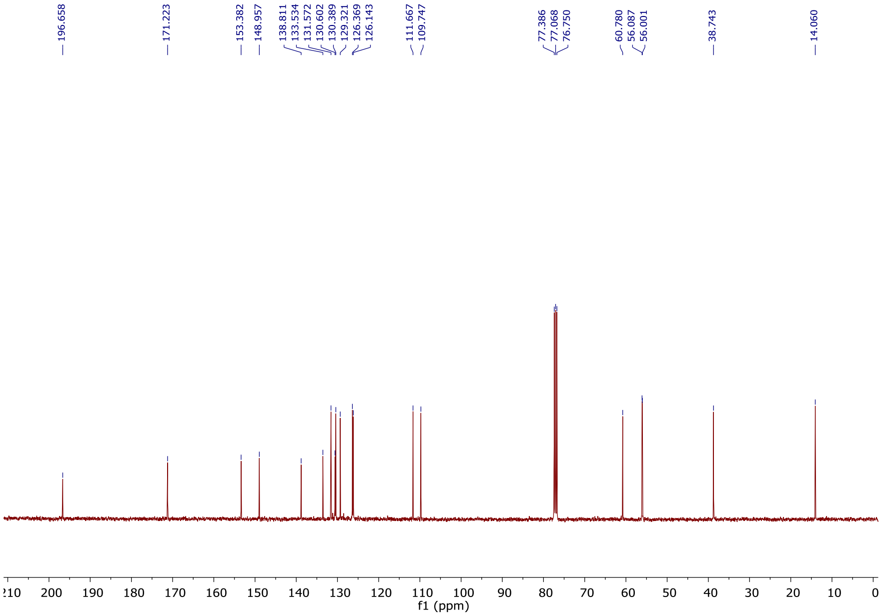

^{13}C NMR (100 MHz, $CDCl_3$) of compound **2d'**

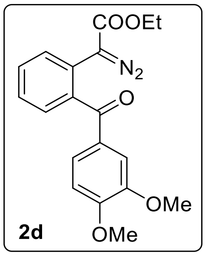

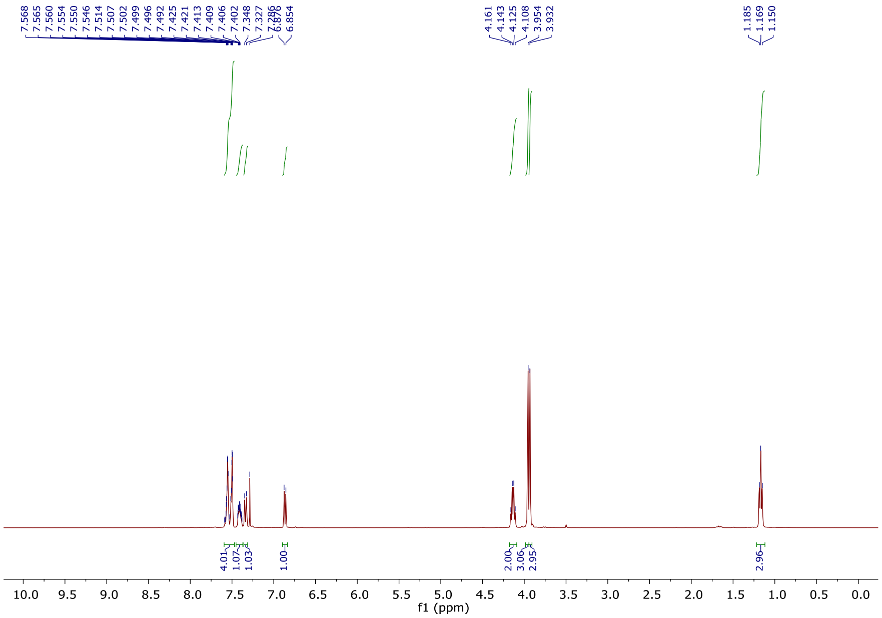

^{1}H NMR (400 MHz, $CDCl_3$) of compound **2d**

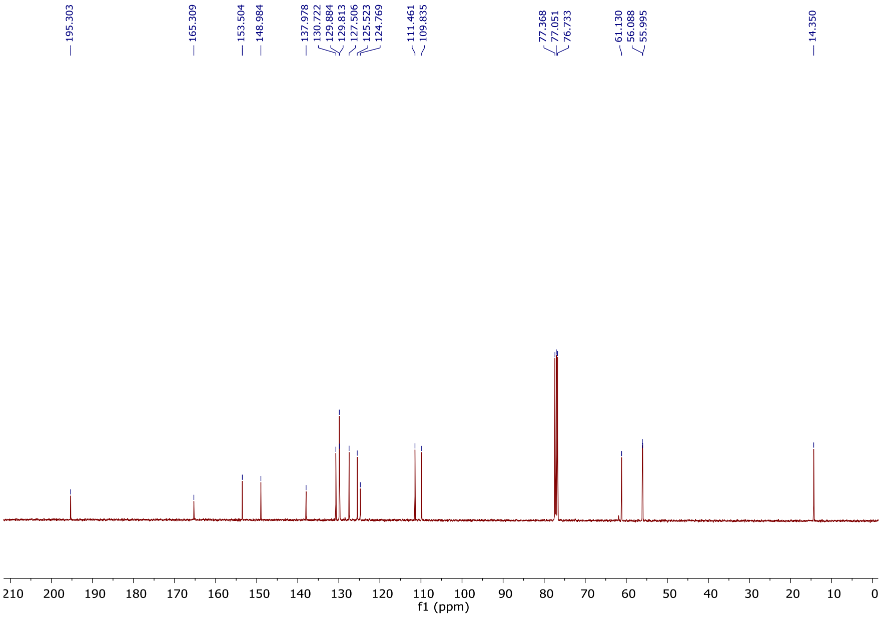

^{13}C NMR (100 MHz, $CDCl_3$) of compound **2d**

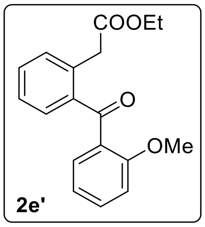

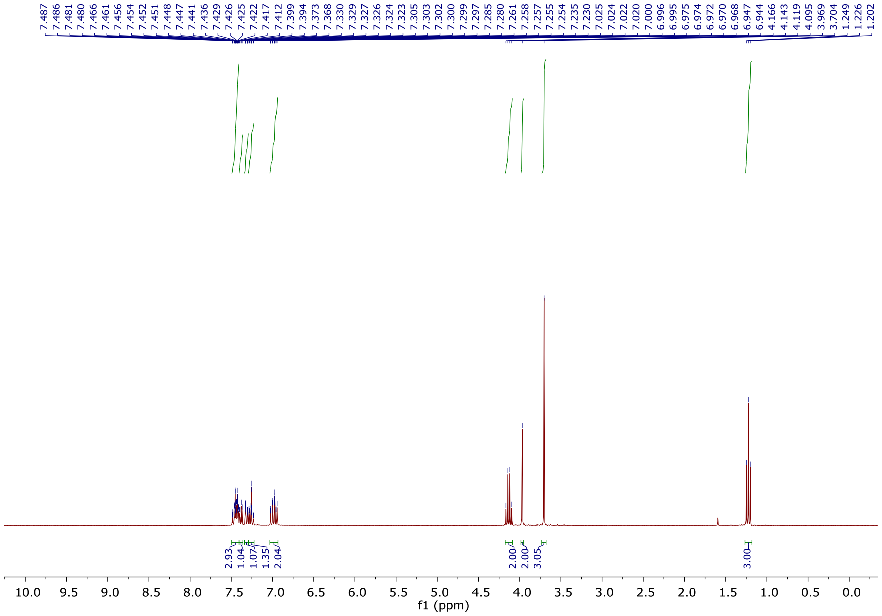

^{1}H NMR (300 MHz, $CDCl_3$) of compound **2e’**

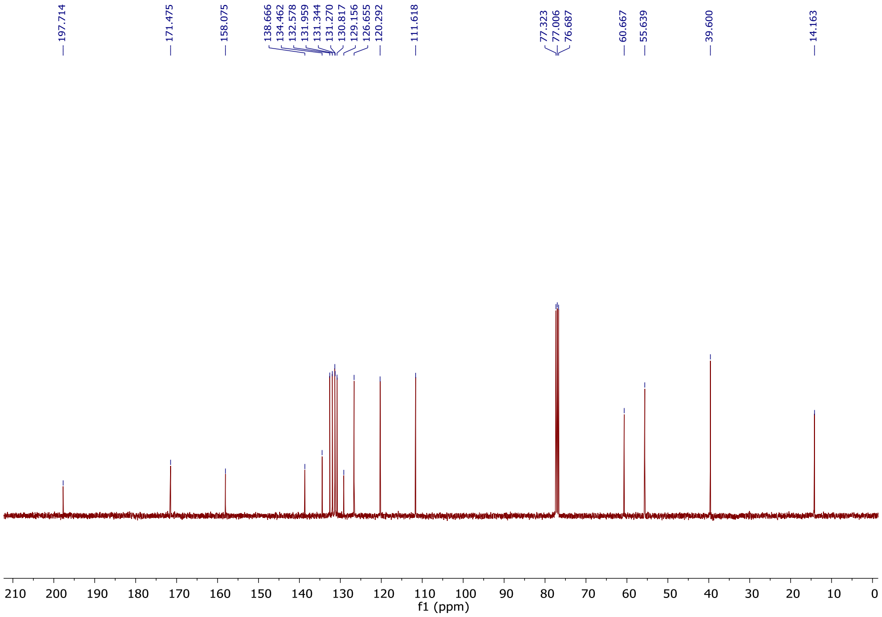

^{13}C NMR (100 MHz, $CDCl_3$) of compound **2e’**

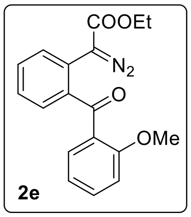

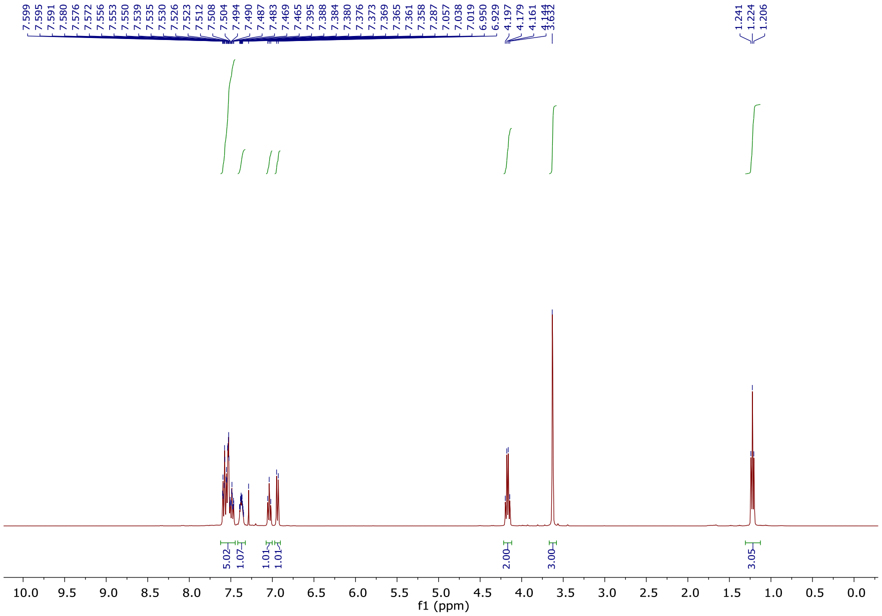

^{1}H NMR (400 MHz, $CDCl_3$) of compound **2e**

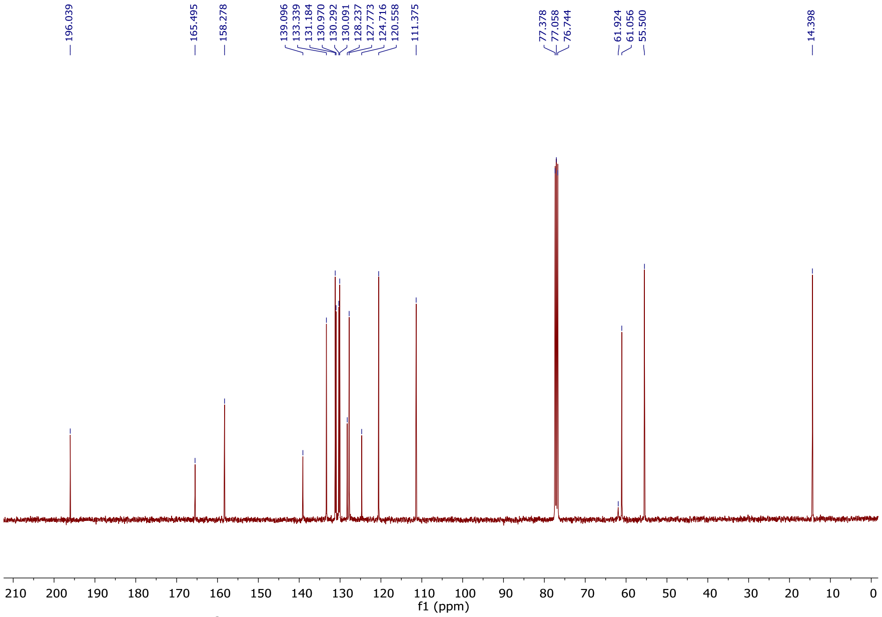

^{13}C NMR (100 MHz, $CDCl_3$) of compound **2e**

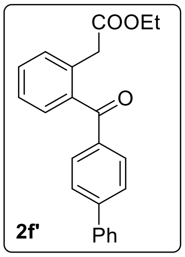

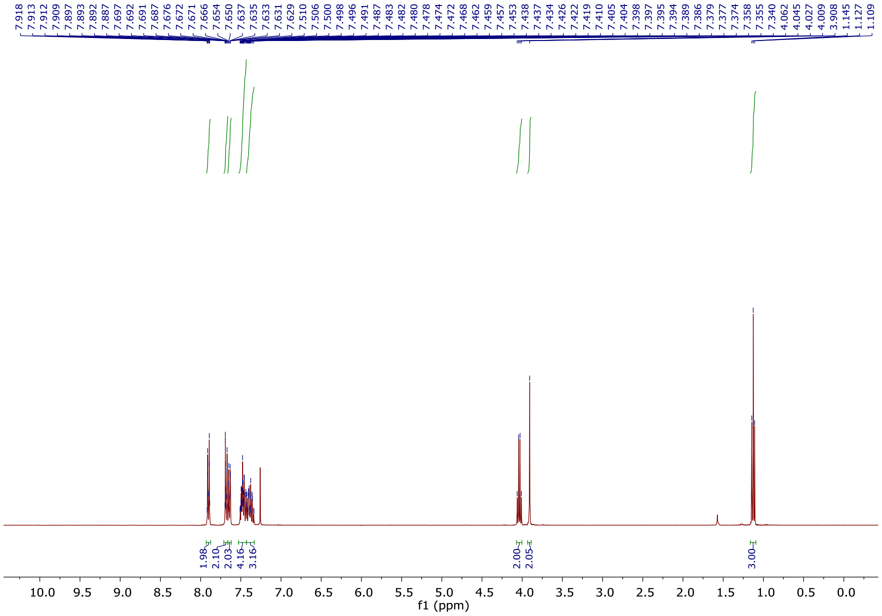

^1H NMR (400 MHz, $CDCl_3$) of compound **2f'**

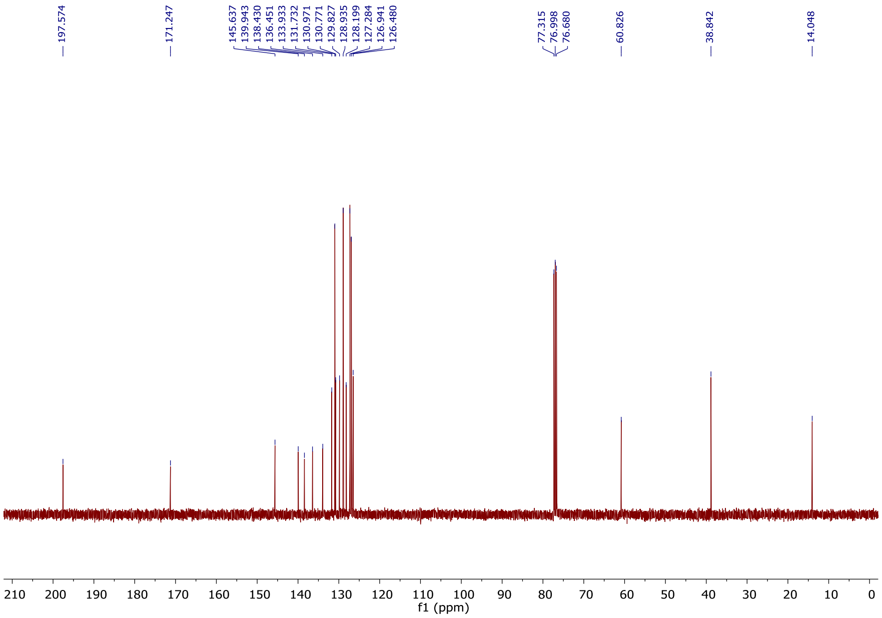

^{13}C NMR (100 MHz, $CDCl_3$) of compound **2f'**

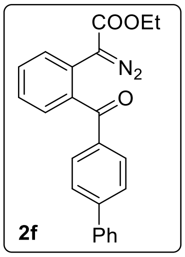

2f

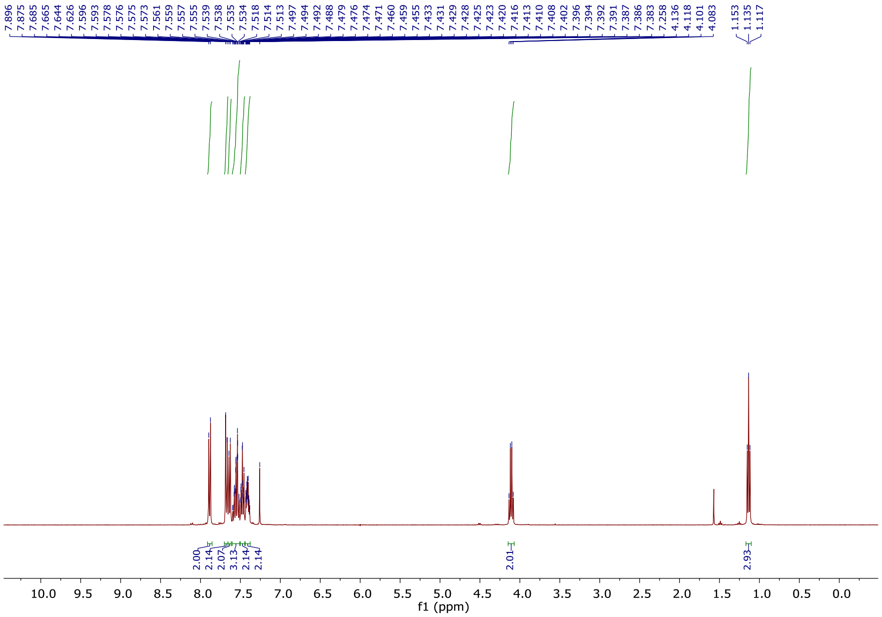

^{1}H NMR (400 MHz, CDCl$_3$) of compound **2f**

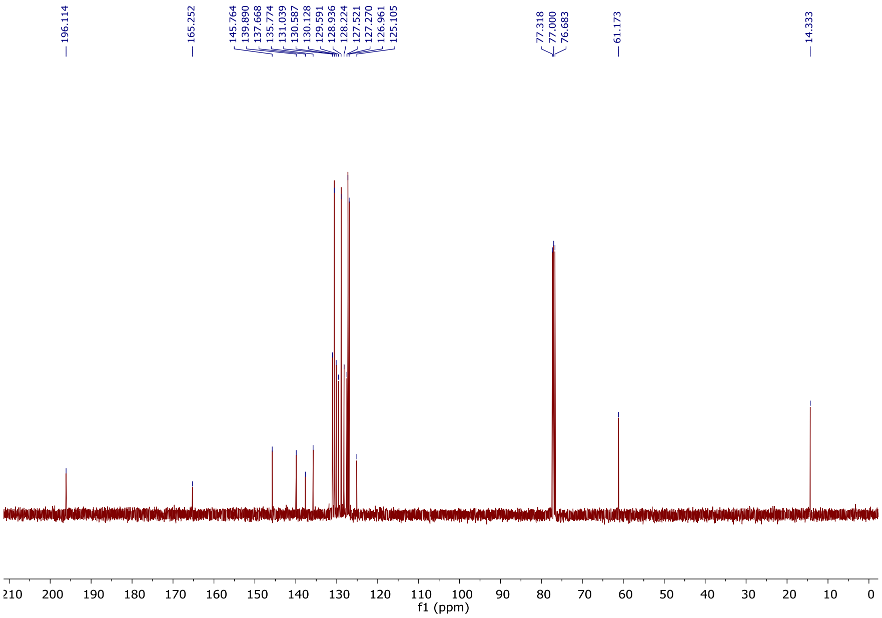

^{13}C NMR (100 MHz, CDCl$_3$) of compound **2f**

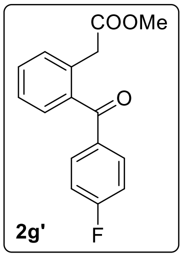

2g'

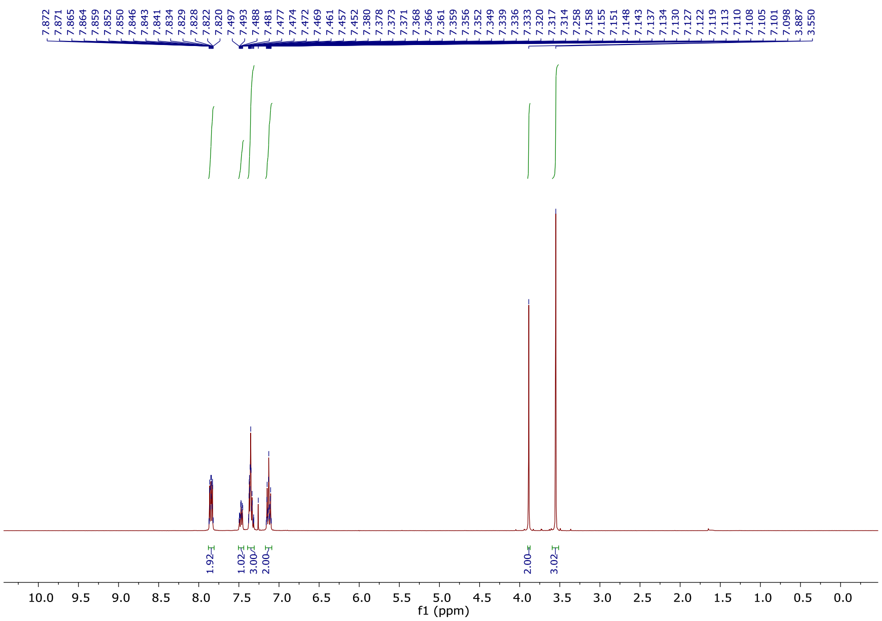

^{1}H NMR (400 MHz, $CDCl_3$) of compound **2g’**

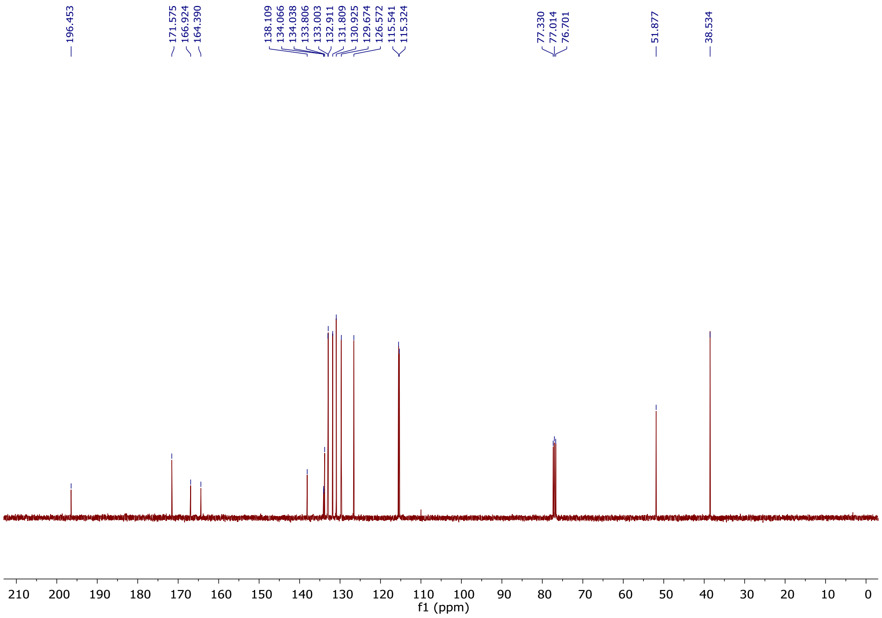

^{13}C NMR (100 MHz, $CDCl_3$) of compound **2g’**

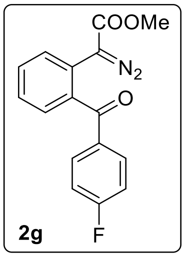

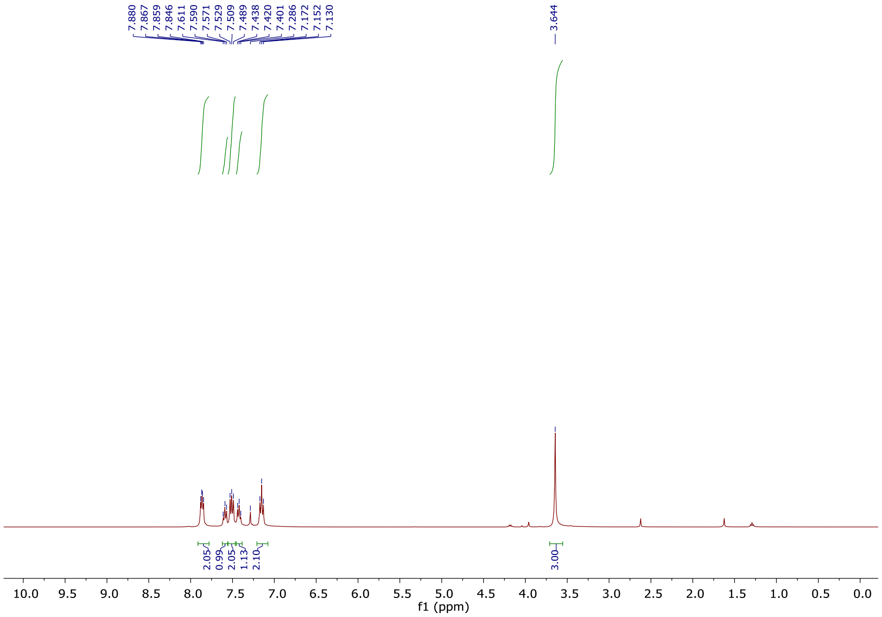

^{1}H NMR (400 MHz, CDCl$_3$) of compound **2g**

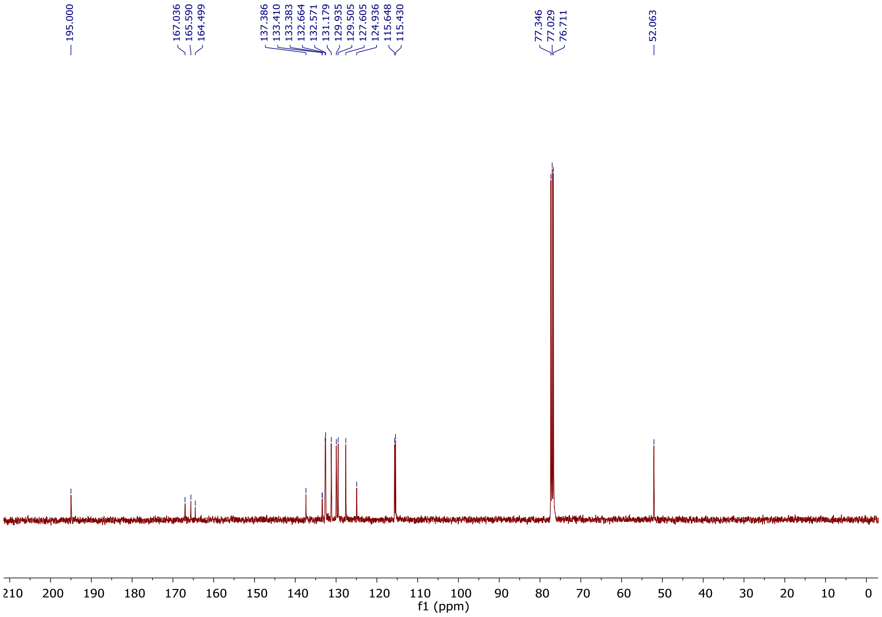

^{13}C NMR (100 MHz, CDCl$_3$) of compound **2g**

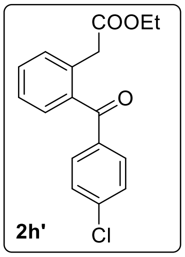

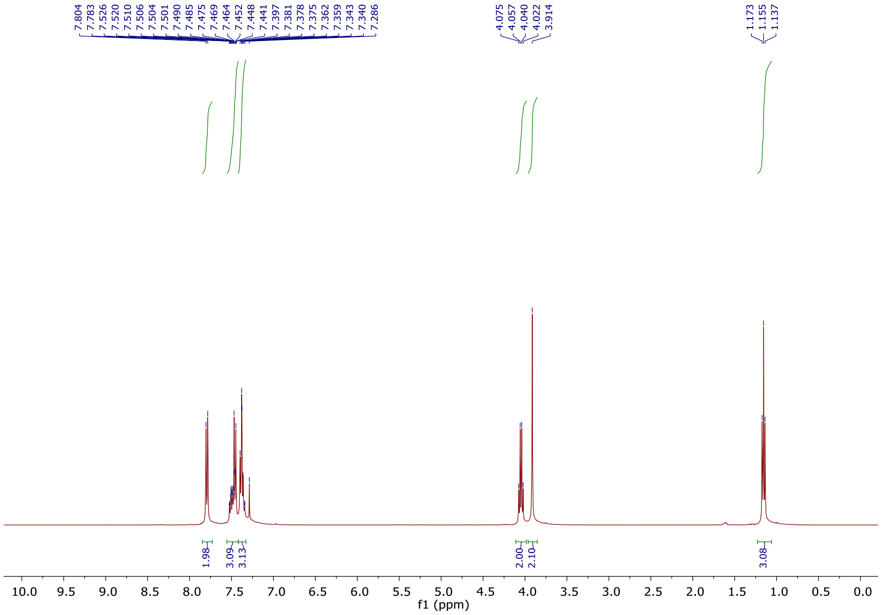

^{1}H NMR (400 MHz, $CDCl_3$) of compound **2h’**

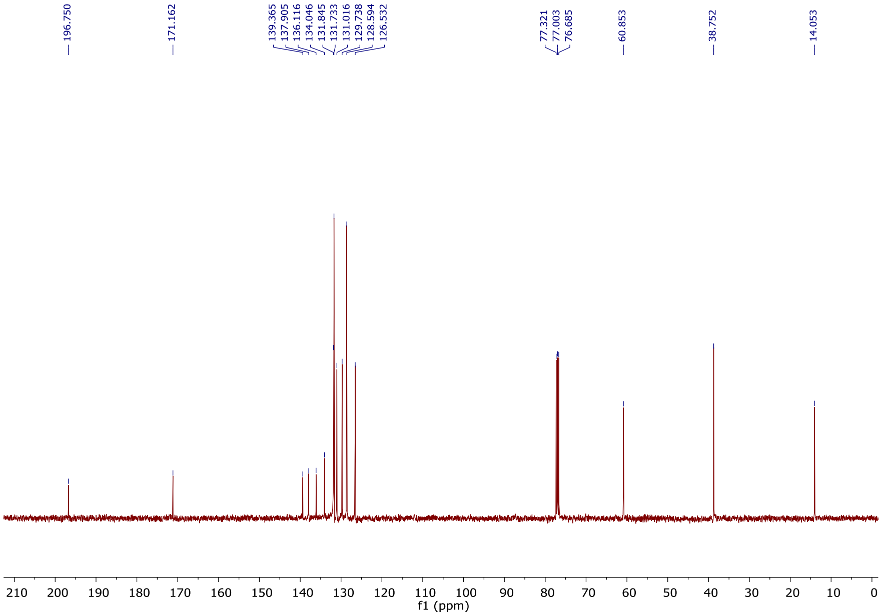

^{13}C NMR (100 MHz, $CDCl_3$) of compound **2h’**

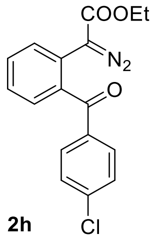

2h

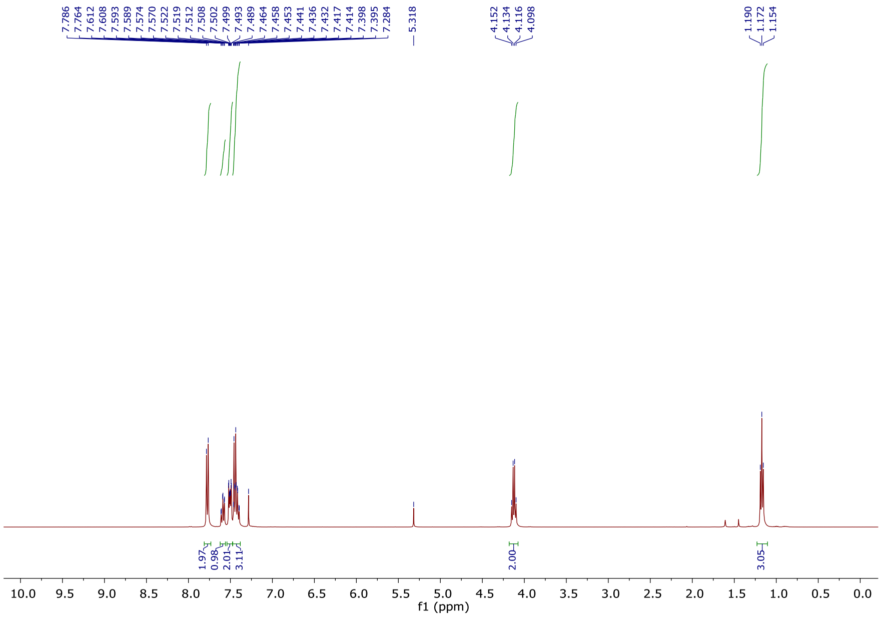

^{1}H NMR (400 MHz, $CDCl_3$) of compound **2h**

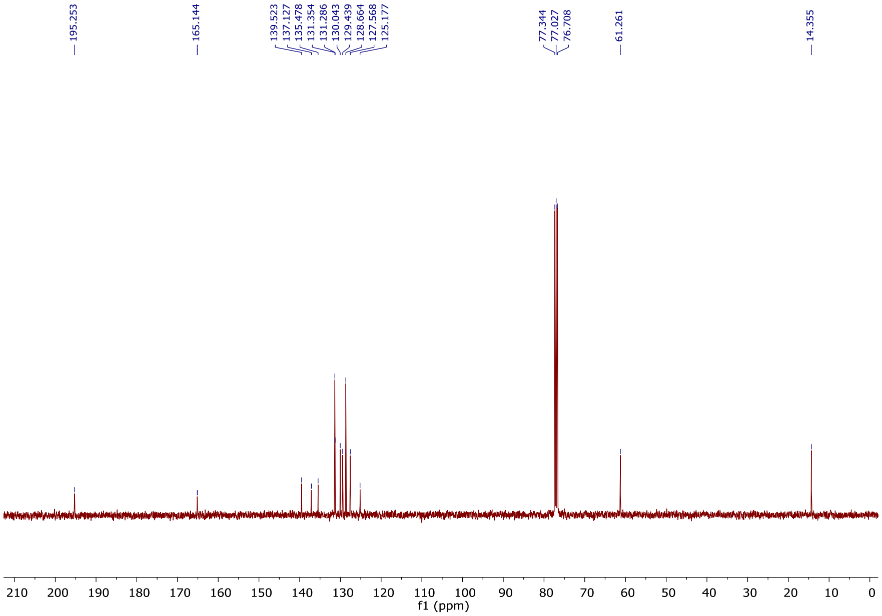

^{13}C NMR (100 MHz, $CDCl_3$) of compound **2h**

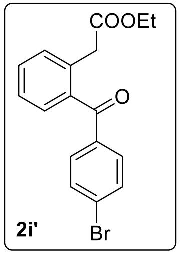

2i'

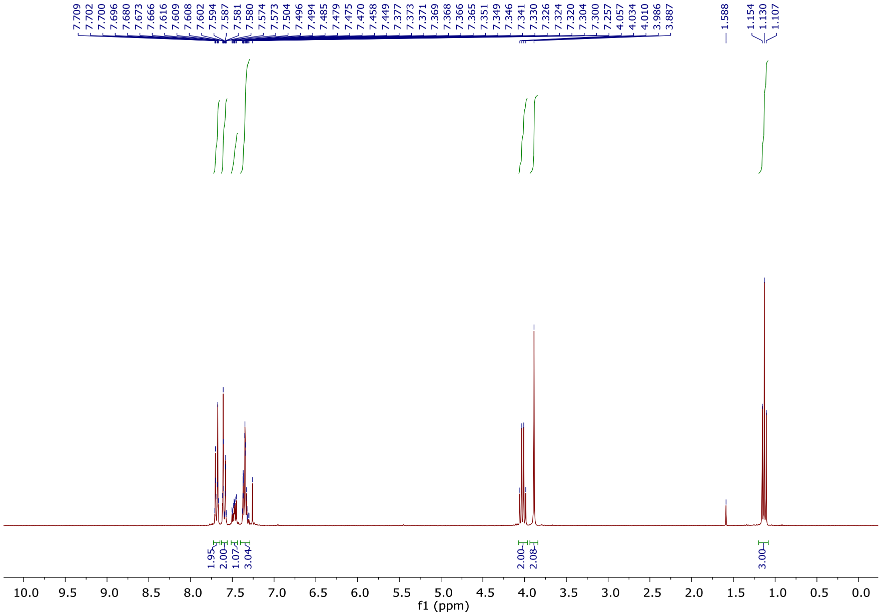

^{1}H NMR (300 MHz, $CDCl_3$) of compound **2i’**

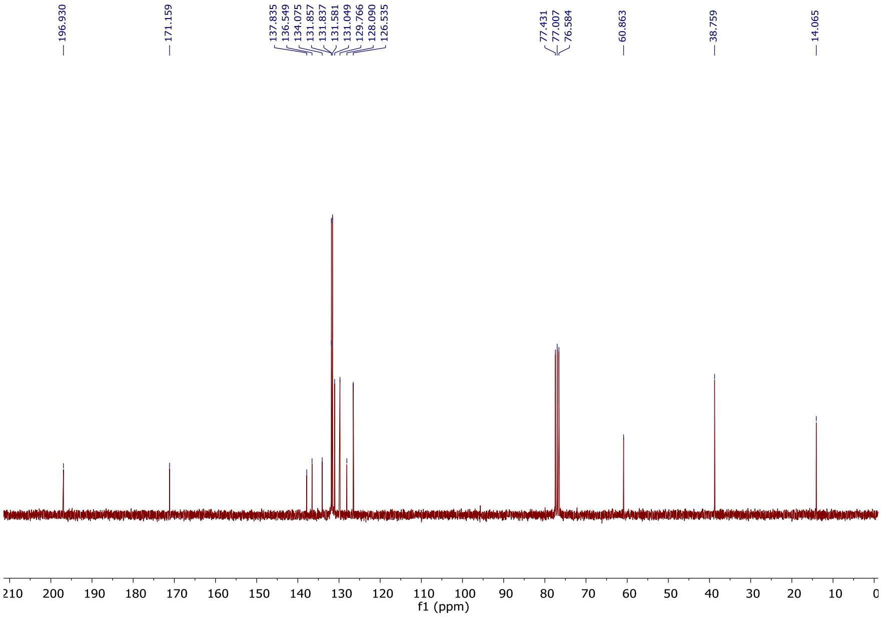

^{13}C NMR (75 MHz, $CDCl_3$) of compound **2i’**

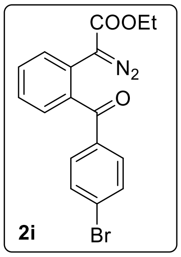

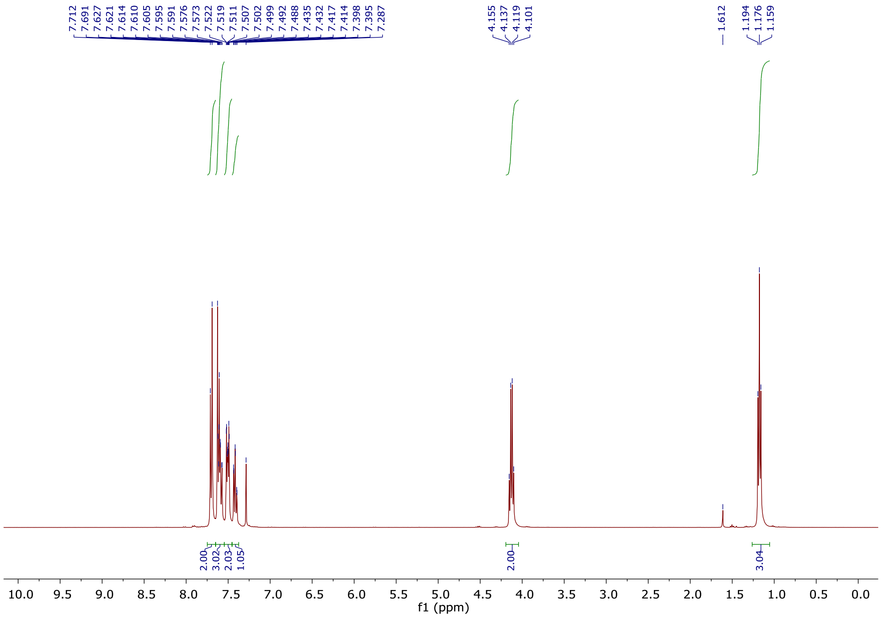

^{1}H NMR (400 MHz, $CDCl_3$) of compound **2i**

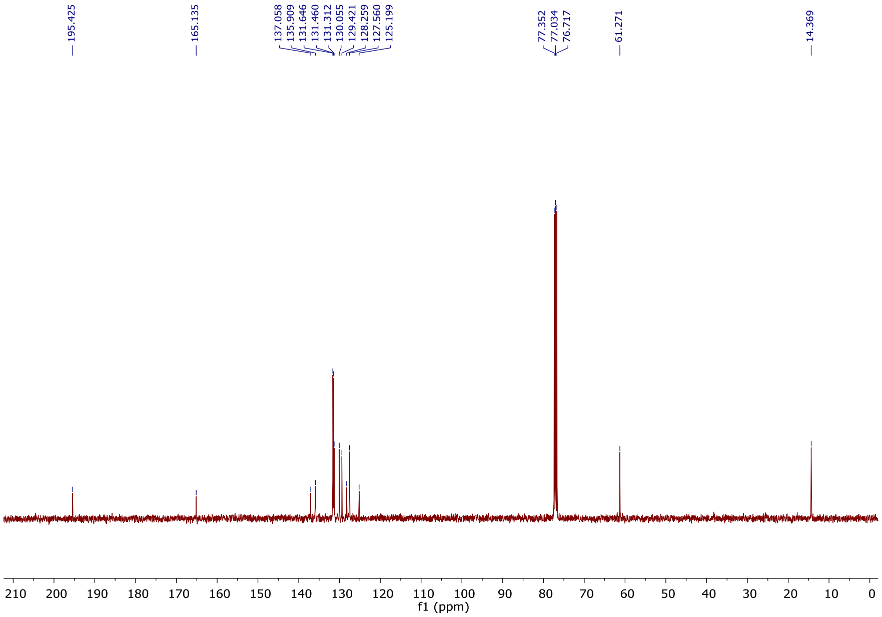

^{13}C NMR (100 MHz, $CDCl_3$) of compound **2i**

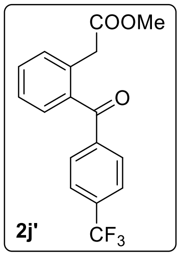

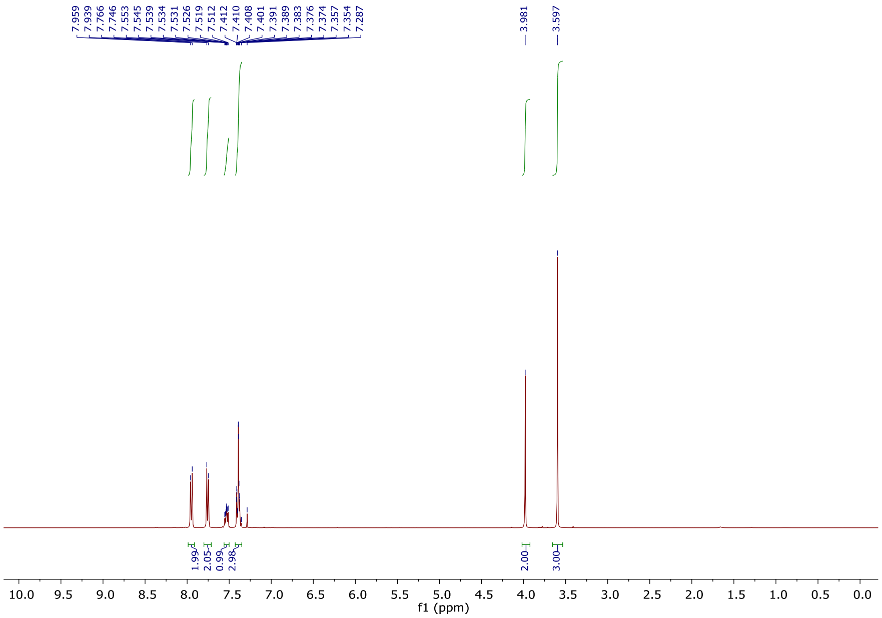

^{1}H NMR (400 MHz, CDCl$_3$) of compound **2j'**

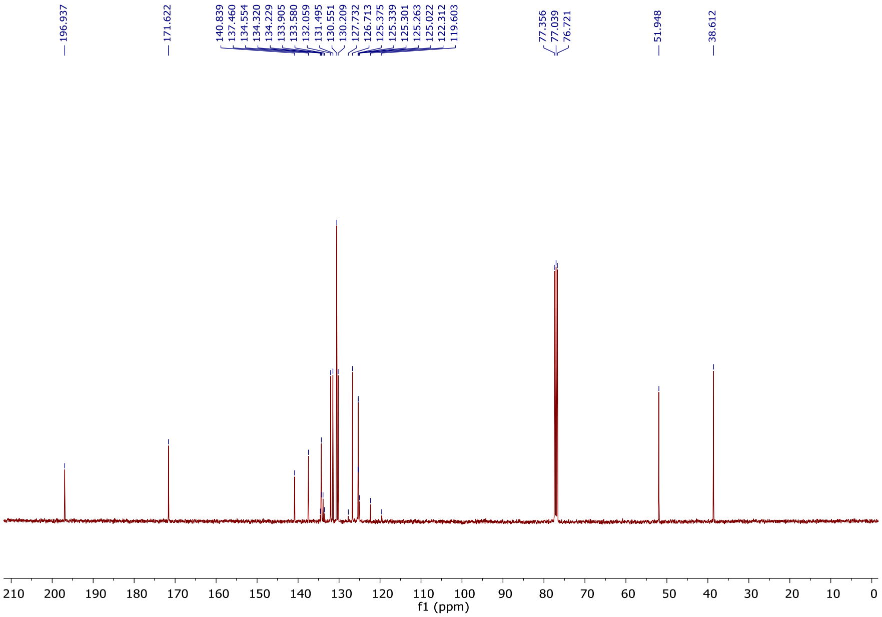

^{13}C NMR (100 MHz, CDCl$_3$) of compound **2j'**

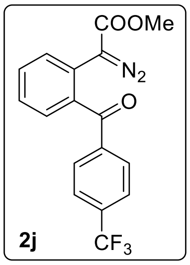

2j

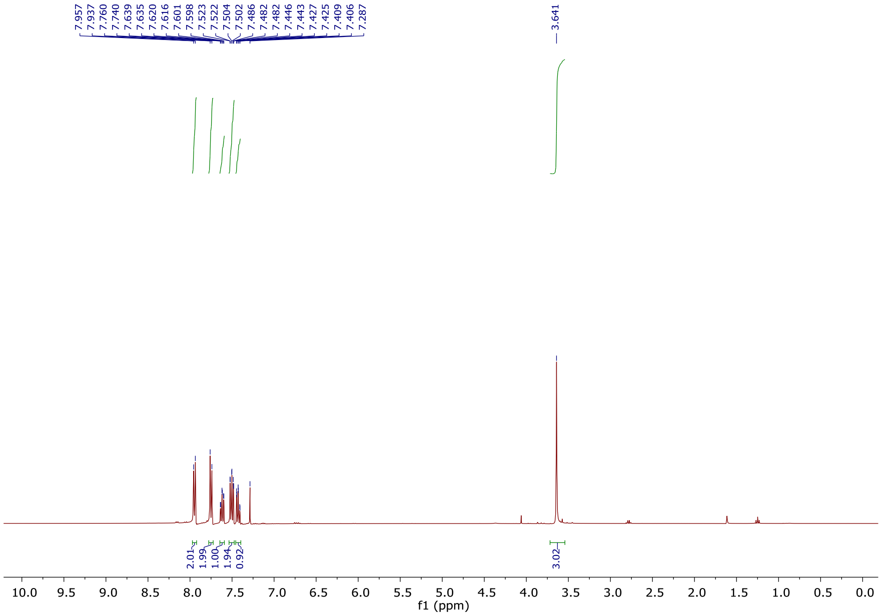

^{1}H NMR (400 MHz, $CDCl_3$) of compound **2j**

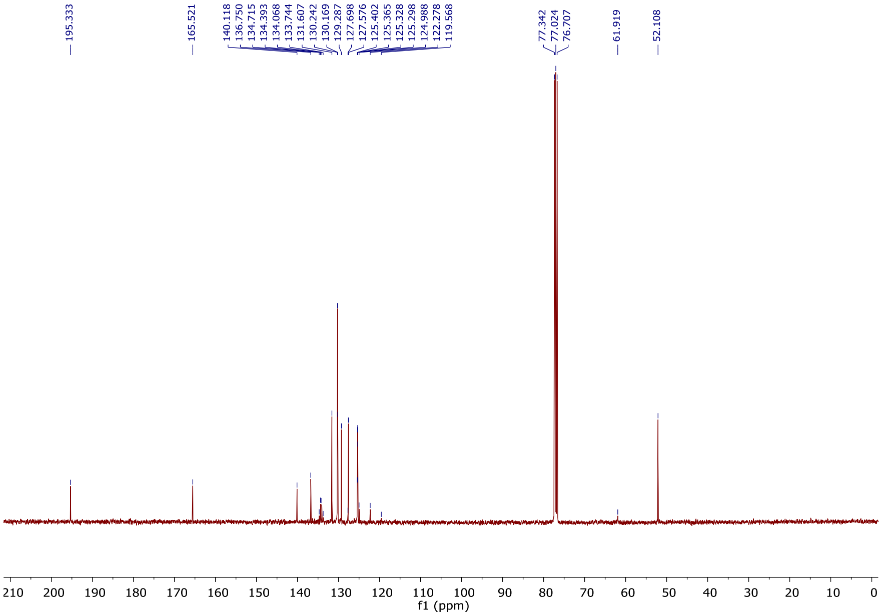

^{13}C NMR (100 MHz, $CDCl_3$) of compound **2j**

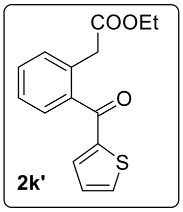

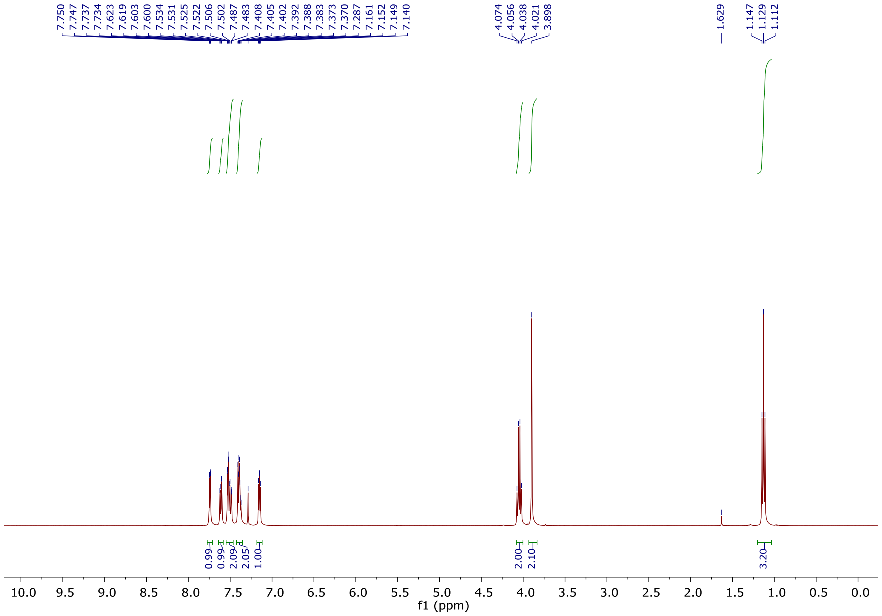

^{1}H NMR (400 MHz, $CDCl_3$) of compound **2k’**

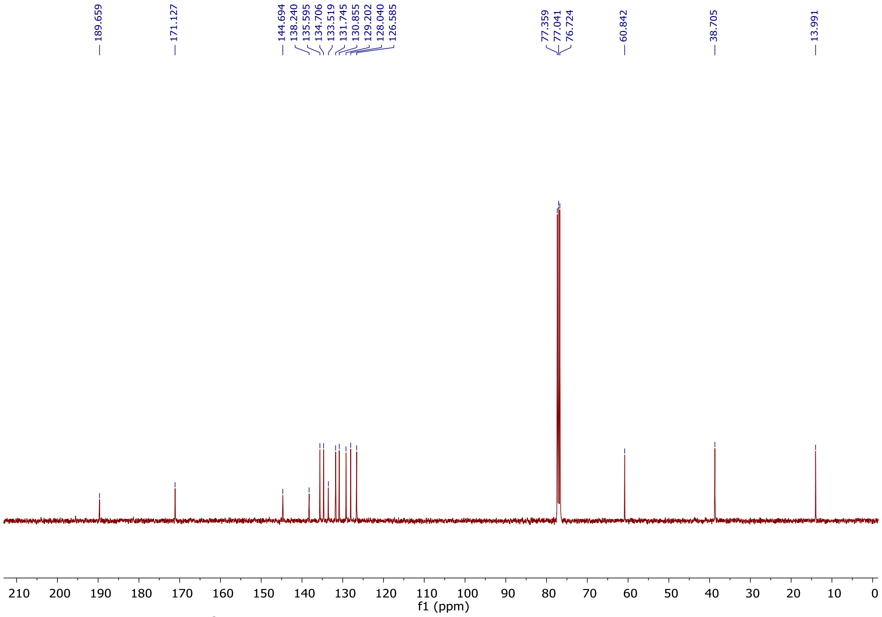

^{13}C NMR (100 MHz, $CDCl_3$) of compound **2k’**

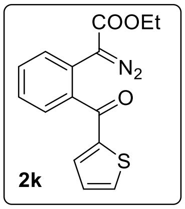

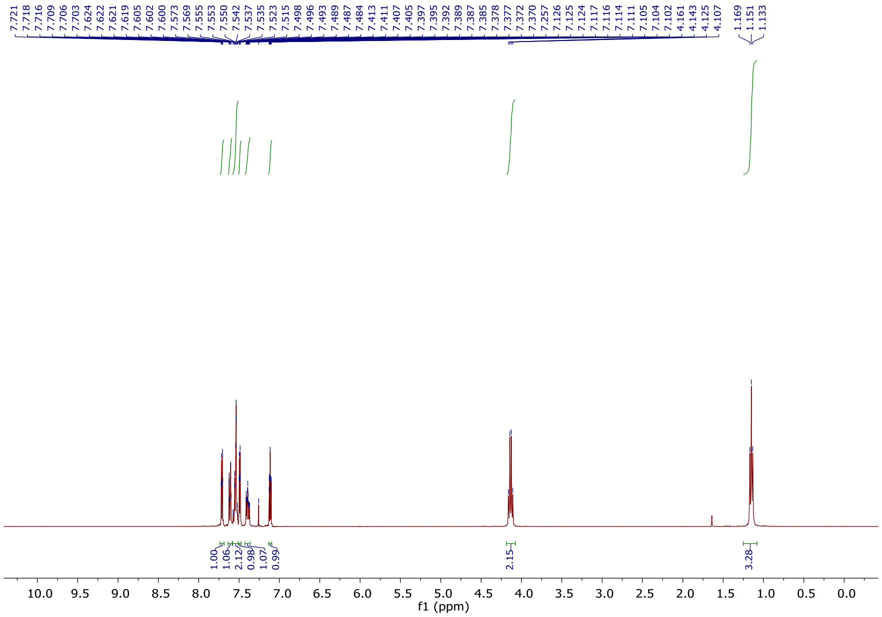

^{1}H NMR (400 MHz, $CDCl_3$) of compound **2k**

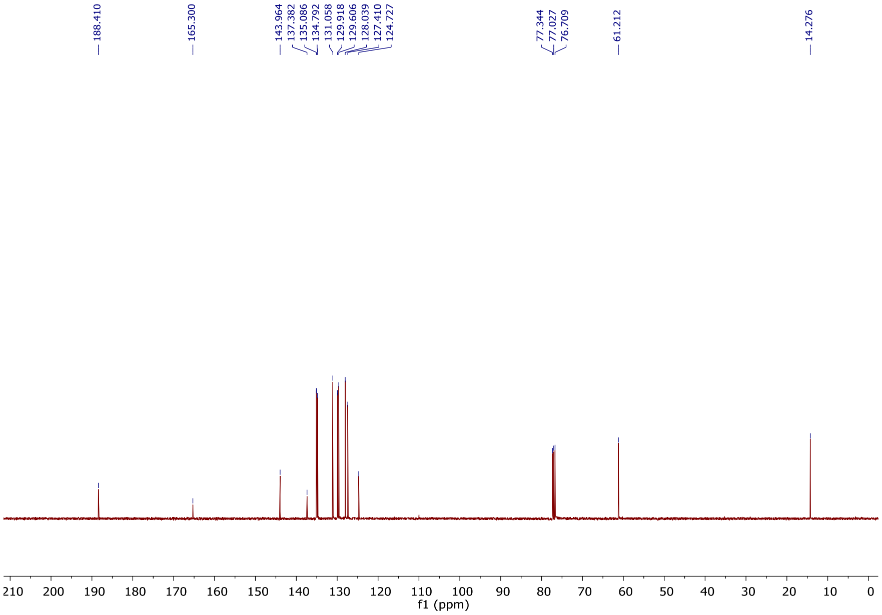

^{13}C NMR (100 MHz, $CDCl_3$) of compound **2k**

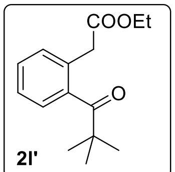

2l'

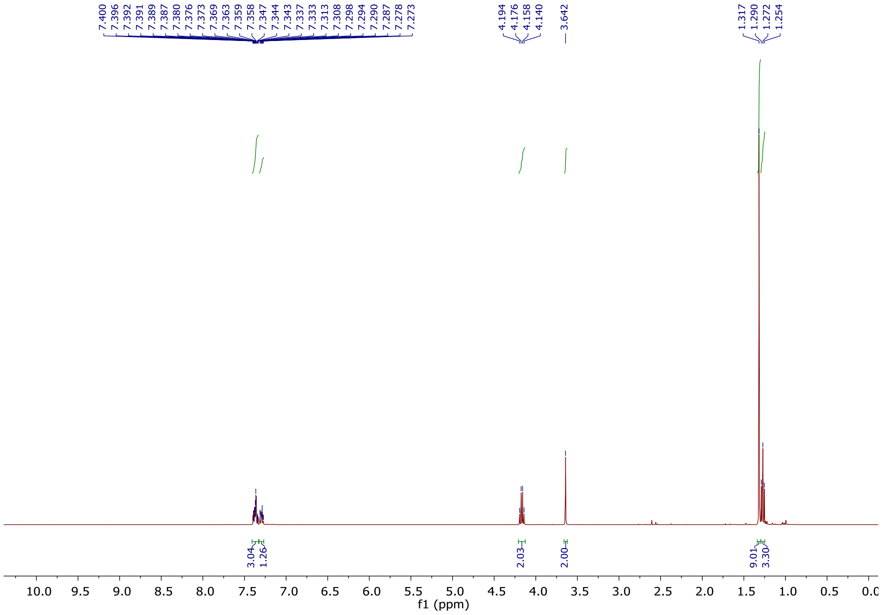

^{1}H NMR (400 MHz, $CDCl_3$) of compound **2l’**

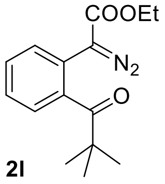

2l

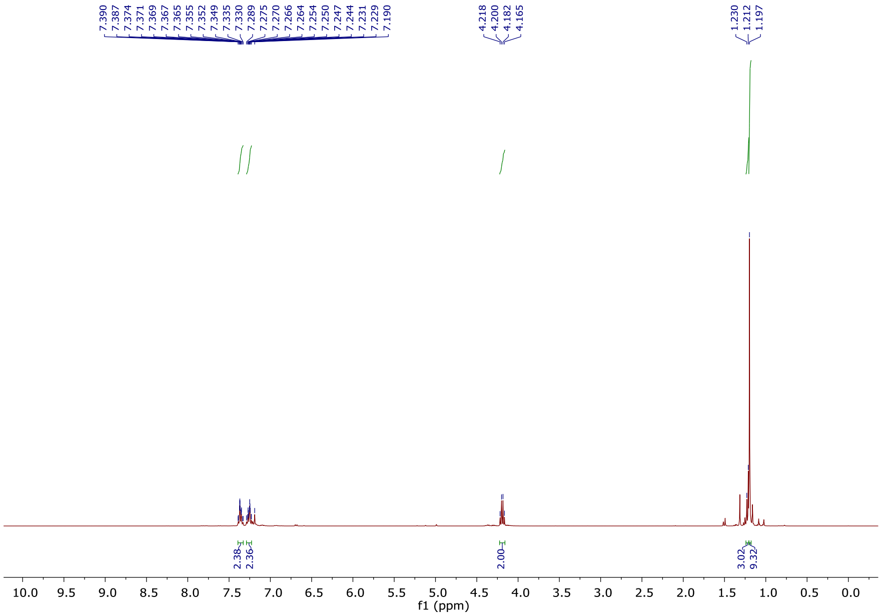

^{1}H NMR (400 MHz, $CDCl_3$) of compound **2l**

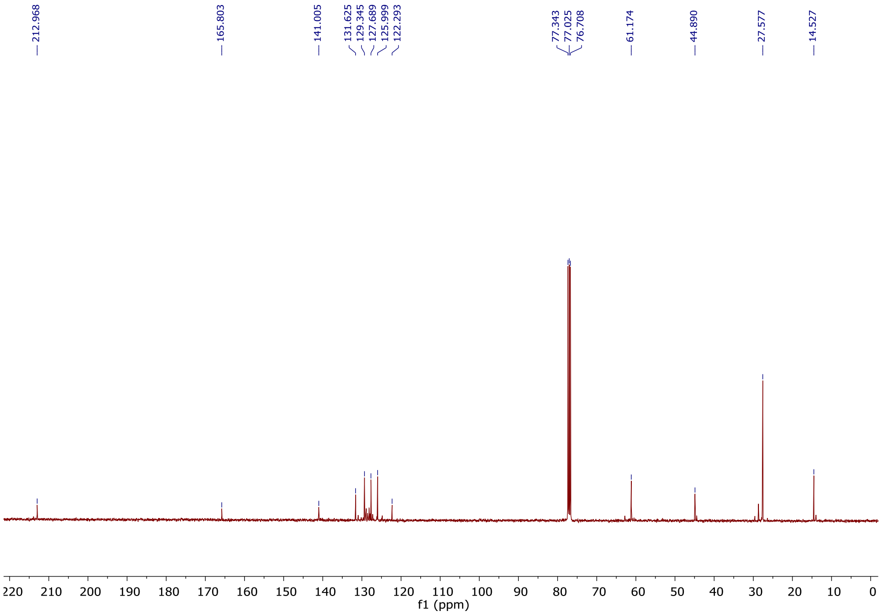

^{13}C NMR (100 MHz, $CDCl_3$) of compound **2l**

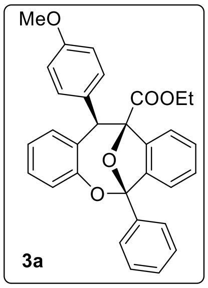

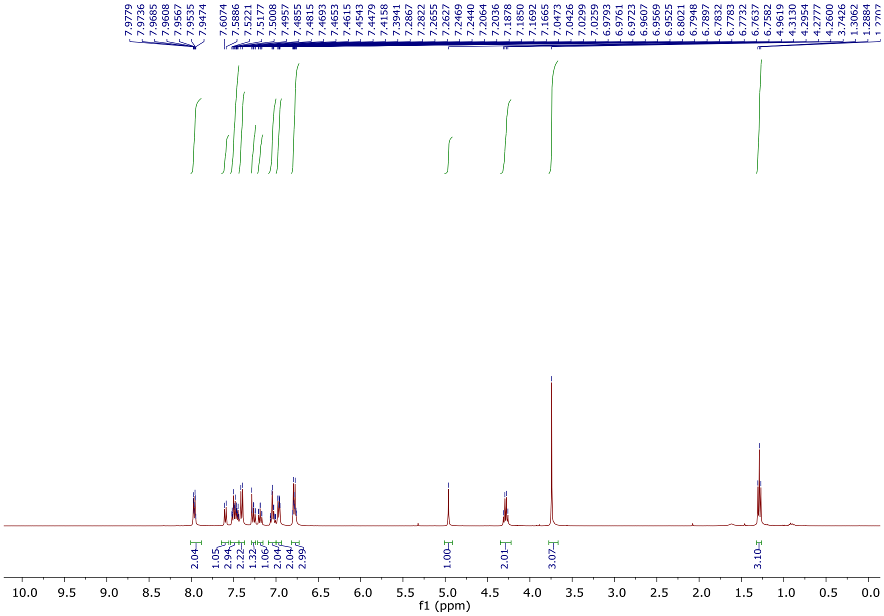

^{1}H NMR (400 MHz, $CDCl_3$) of compound **3a**

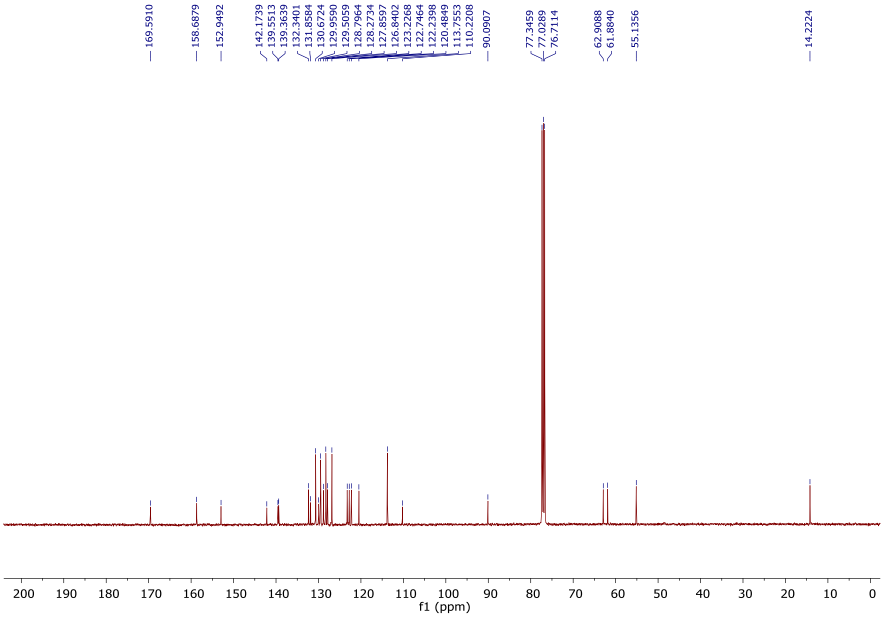

^{13}C NMR (100 MHz, $CDCl_3$) of compound **3a**

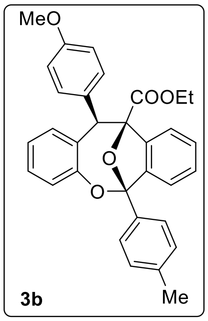

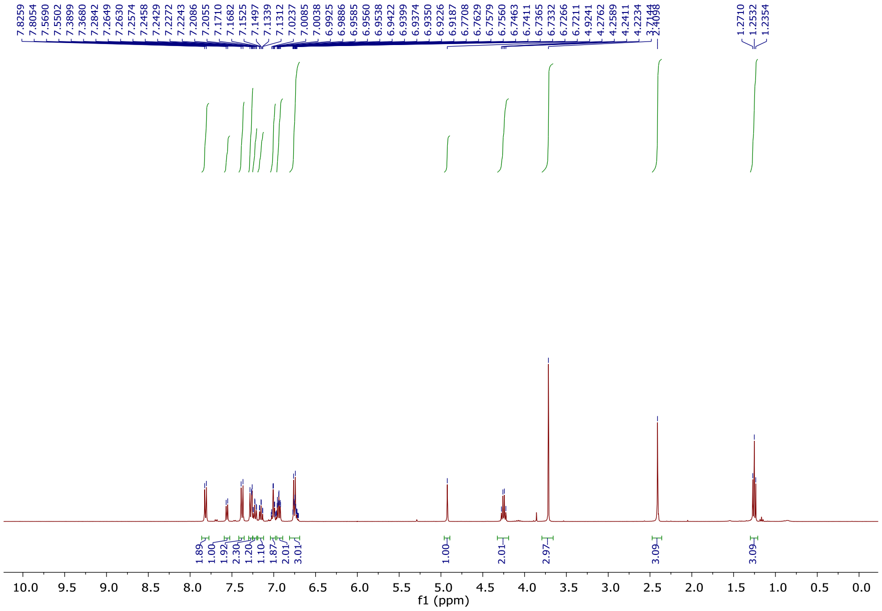

^{1}H NMR (400 MHz, $CDCl_3$) of compound **3b**

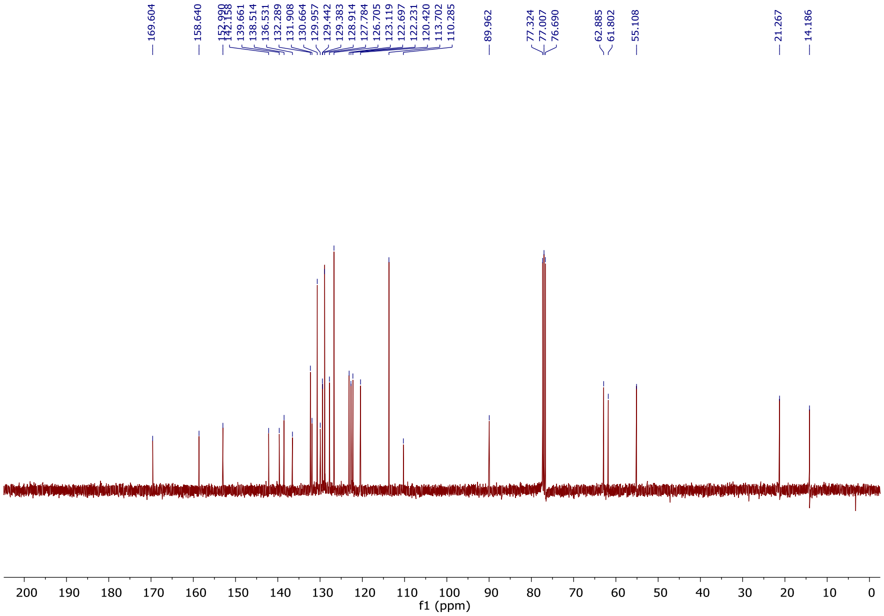

^{13}C NMR (100 MHz, $CDCl_3$) of compound **3b**

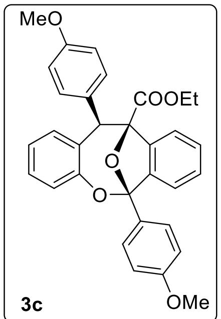

3c

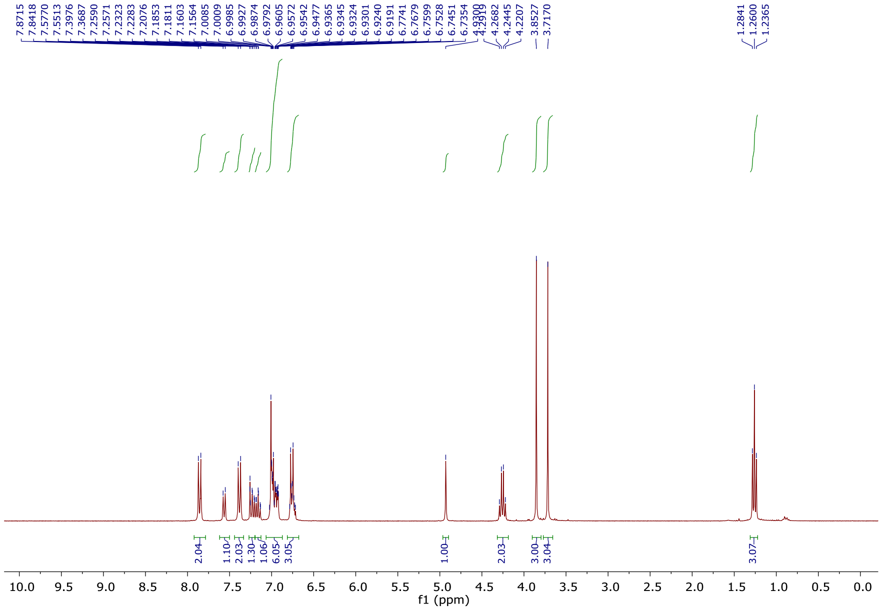

^{1}H NMR (300 MHz, $CDCl_3$) of compound **3c**

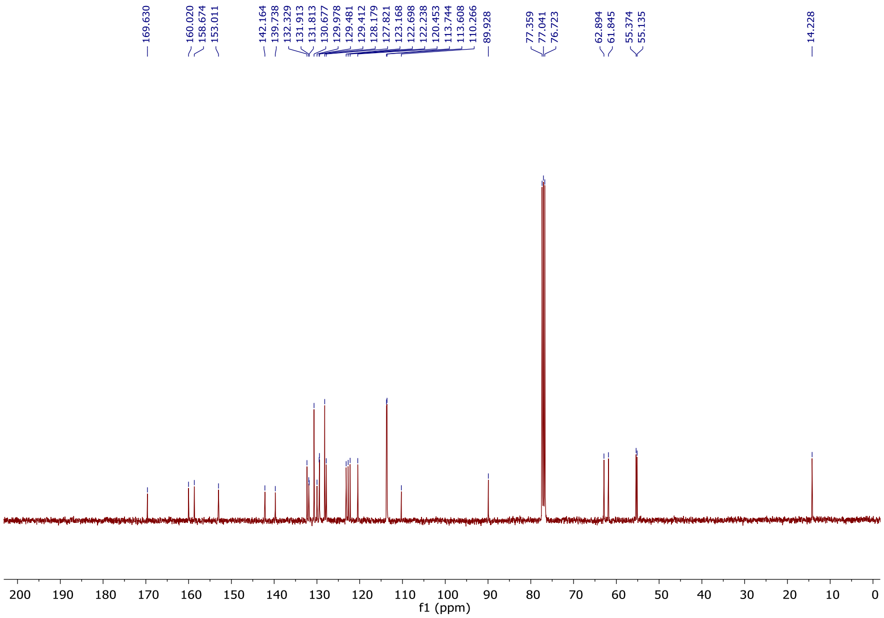

^{13}C NMR (100 MHz, $CDCl_3$) of compound **3c**

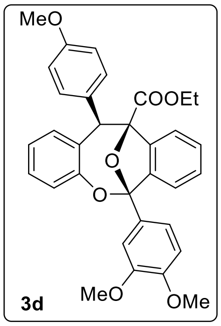

3d

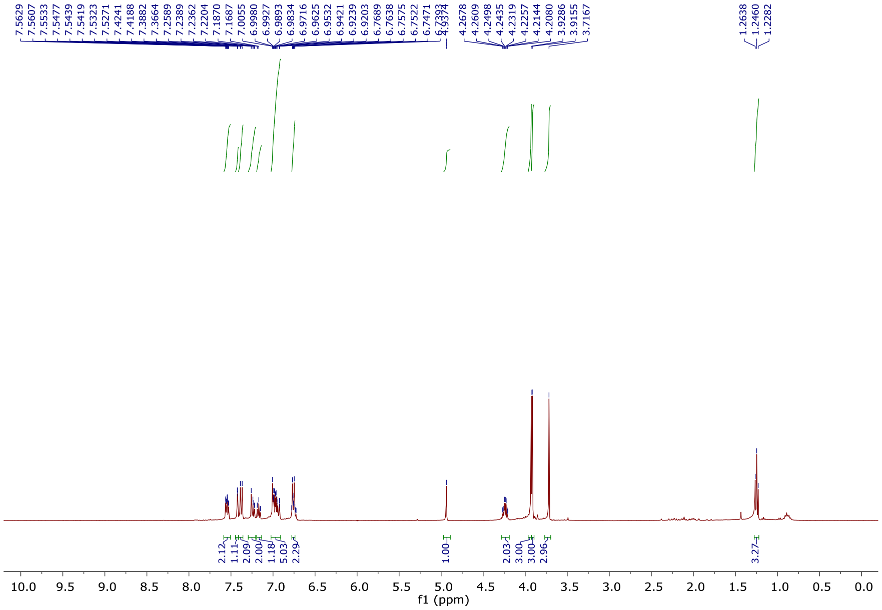

^{1}H NMR (400 MHz, $CDCl_3$) of compound **3d**

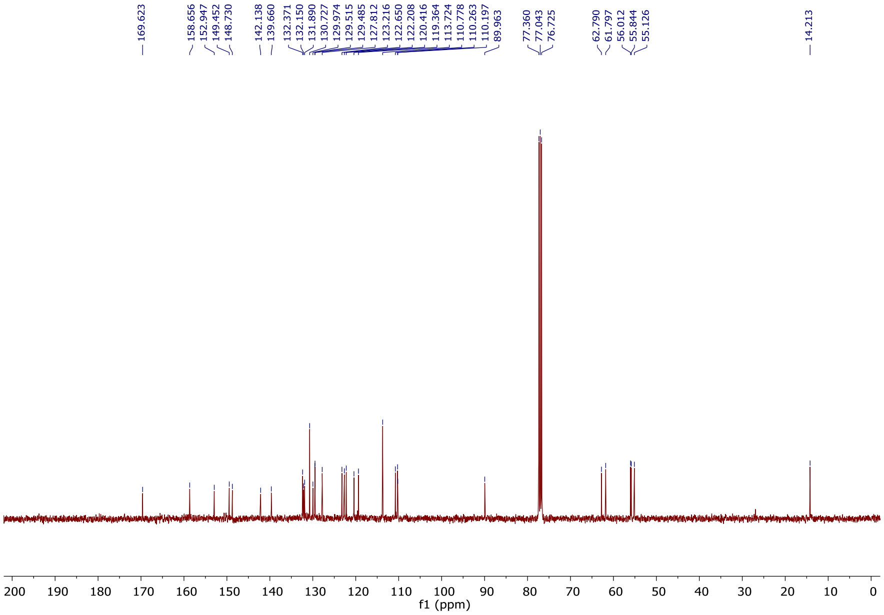

^{13}C NMR (100 MHz, $CDCl_3$) of compound **3d**

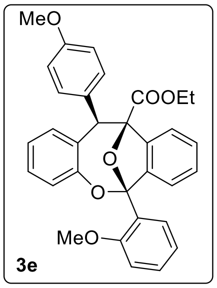

3e

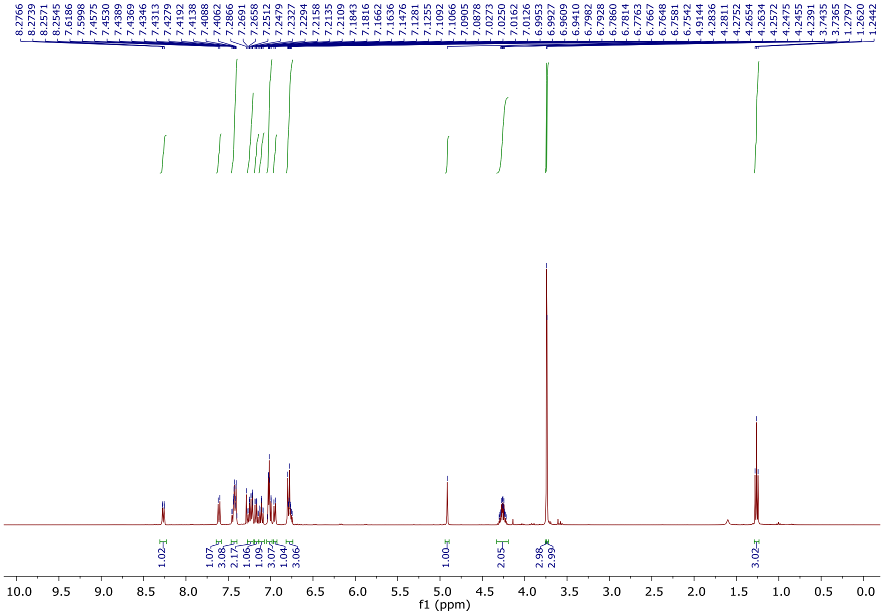

^{1}H NMR (400 MHz, $CDCl_3$) of compound **3e**

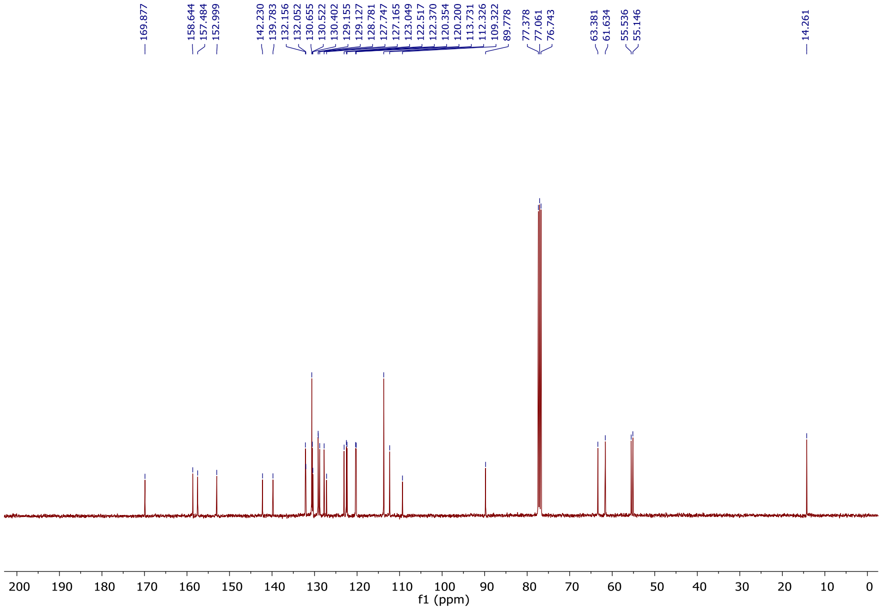

^{13}C NMR (100 MHz, $CDCl_3$) of compound **3e**

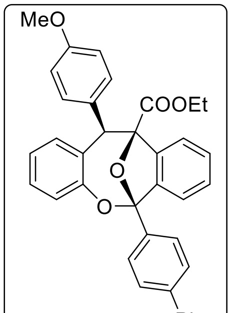

3f

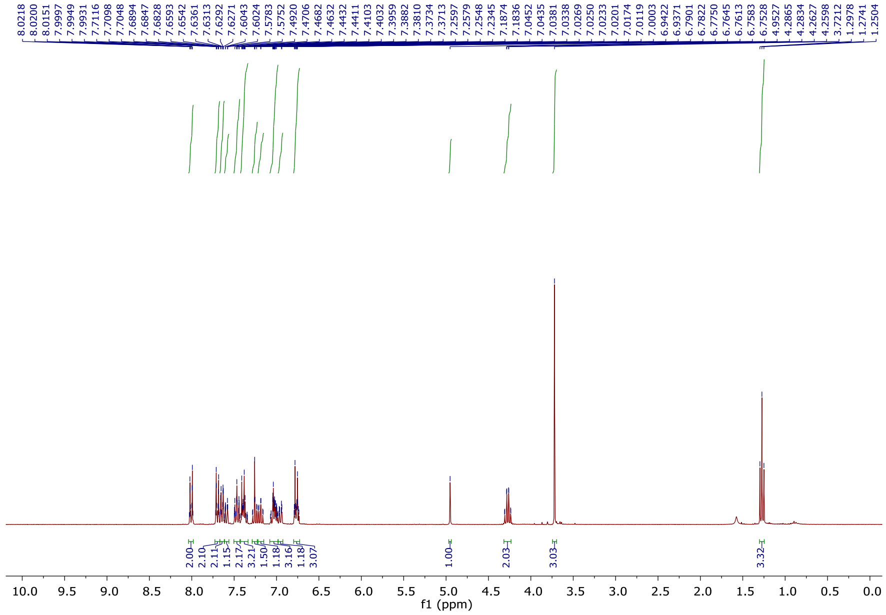

^{1}H NMR (300 MHz, $CDCl_3$) of compound **3f**

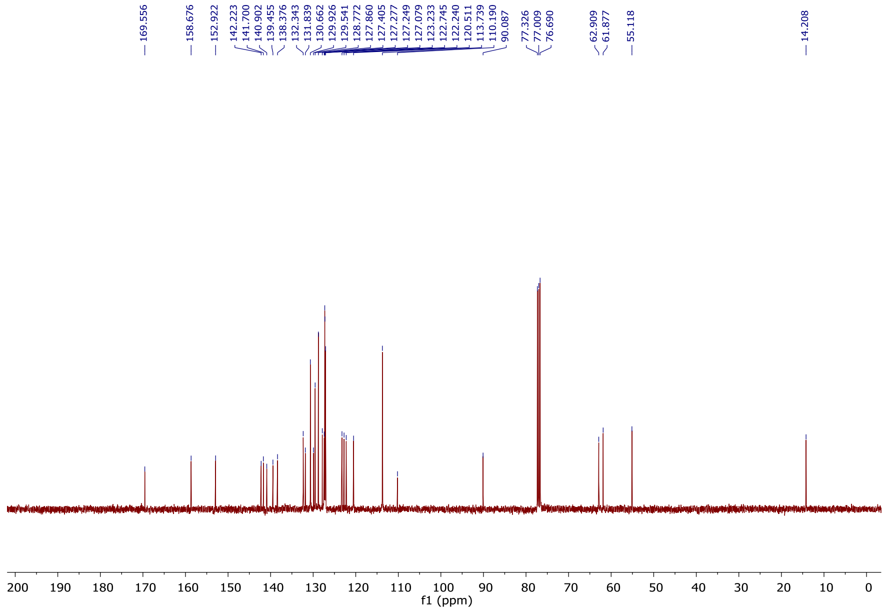

^{13}C NMR (100 MHz, $CDCl_3$) of compound **3f**

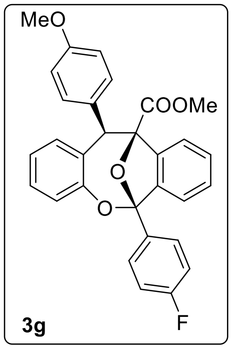

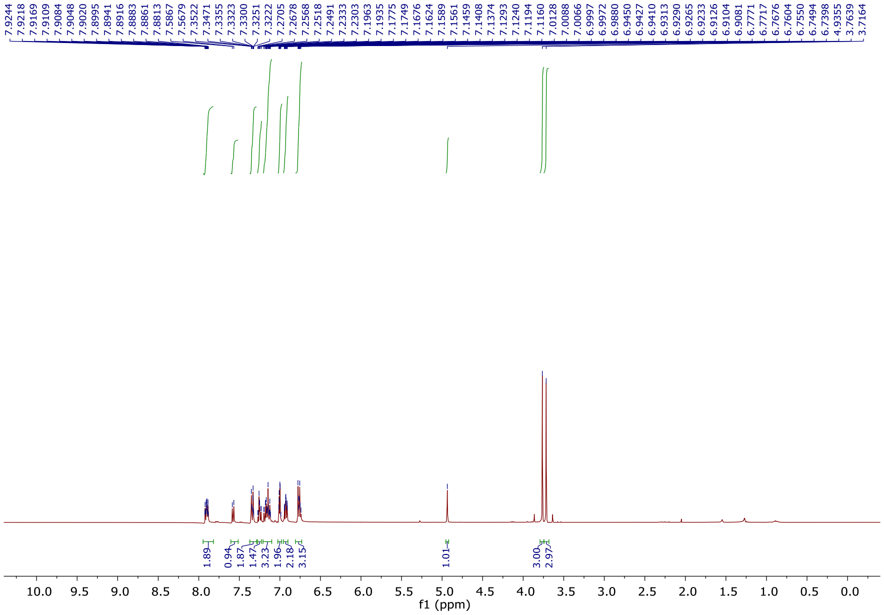

^{1}H NMR (400 MHz, $CDCl_3$) of compound **3g**

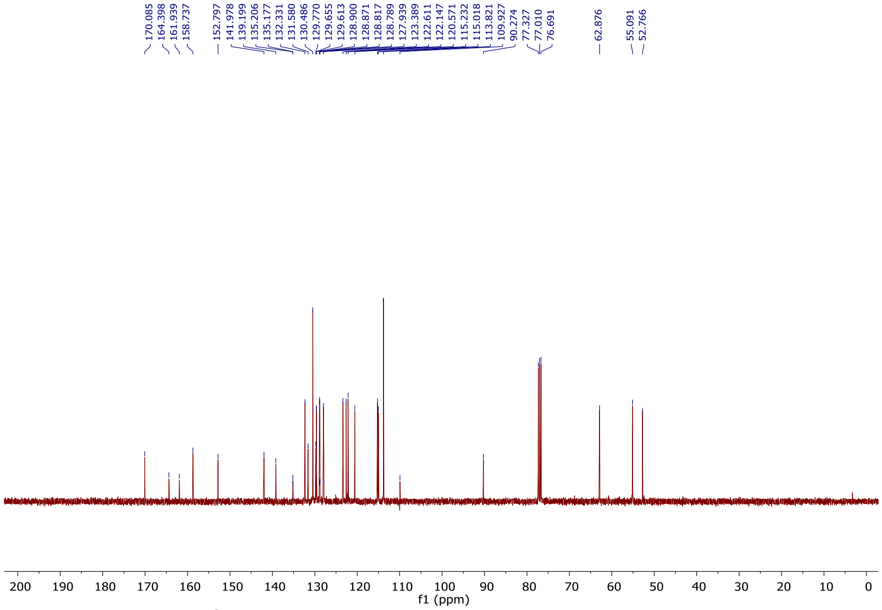

^{13}C NMR (100 MHz, $CDCl_3$) of compound **3g**

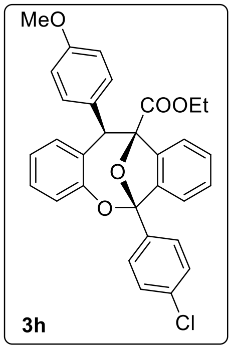

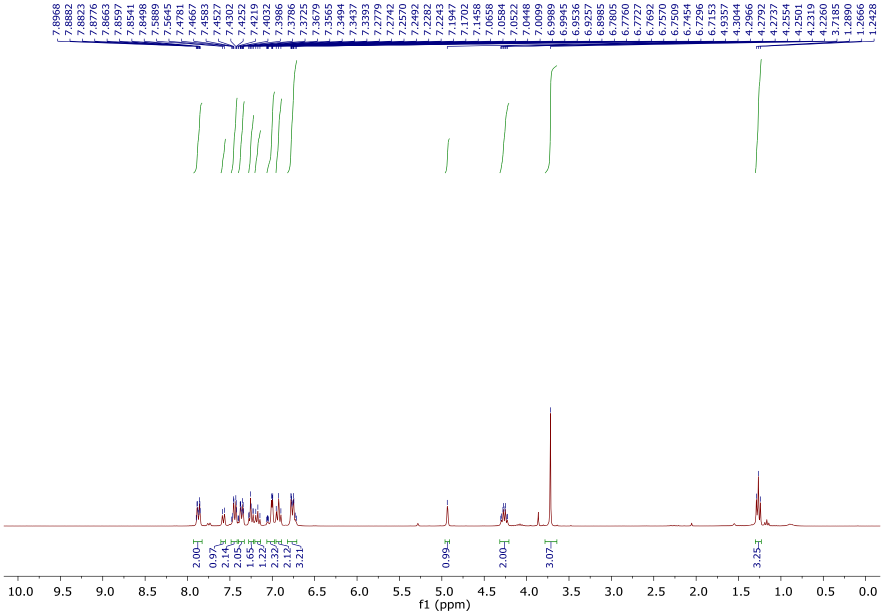

^{1}H NMR (300 MHz, $CDCl_3$) of compound **3h**

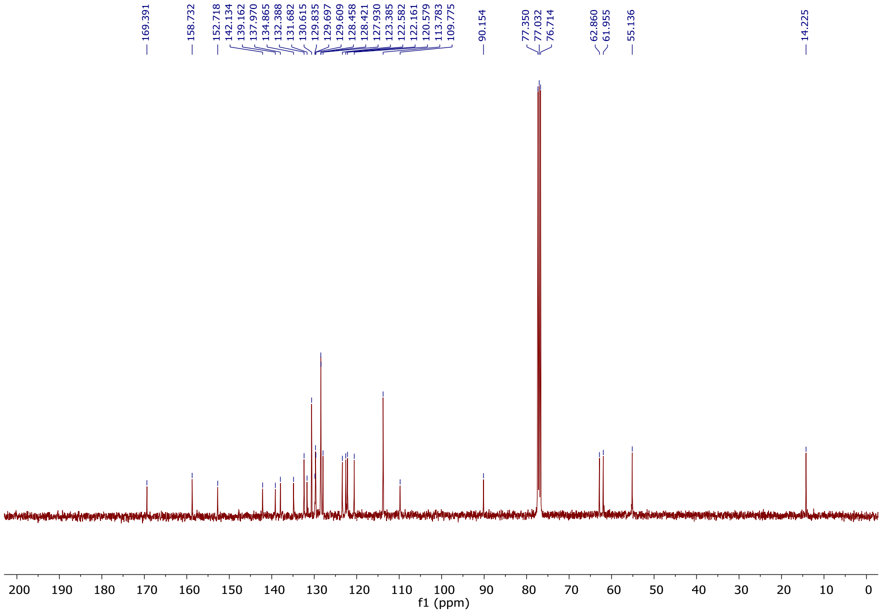

^{13}C NMR (100 MHz, $CDCl_3$) of compound **3h**

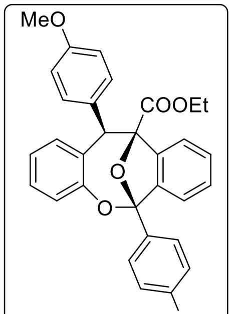

3i

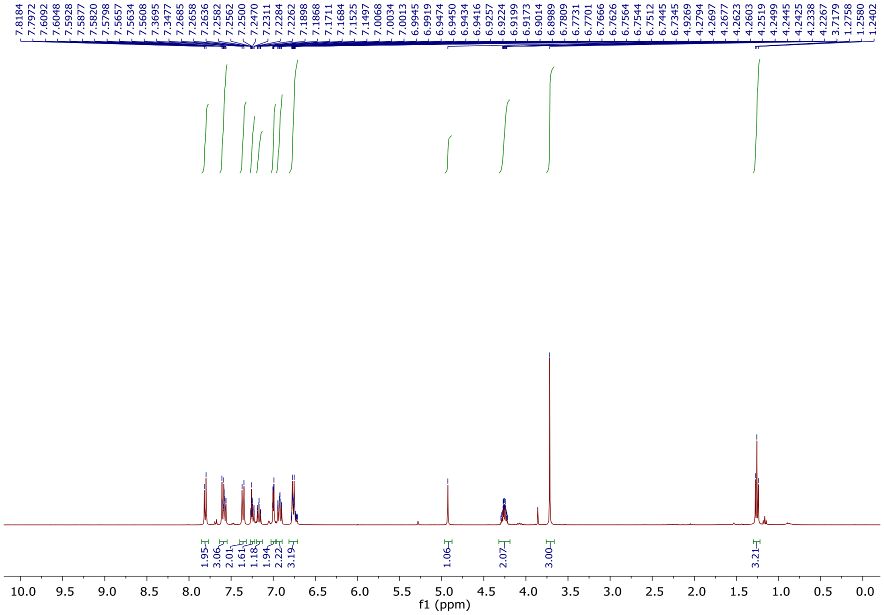

^{1}H NMR (400 MHz, $CDCl_3$) of compound **3i**

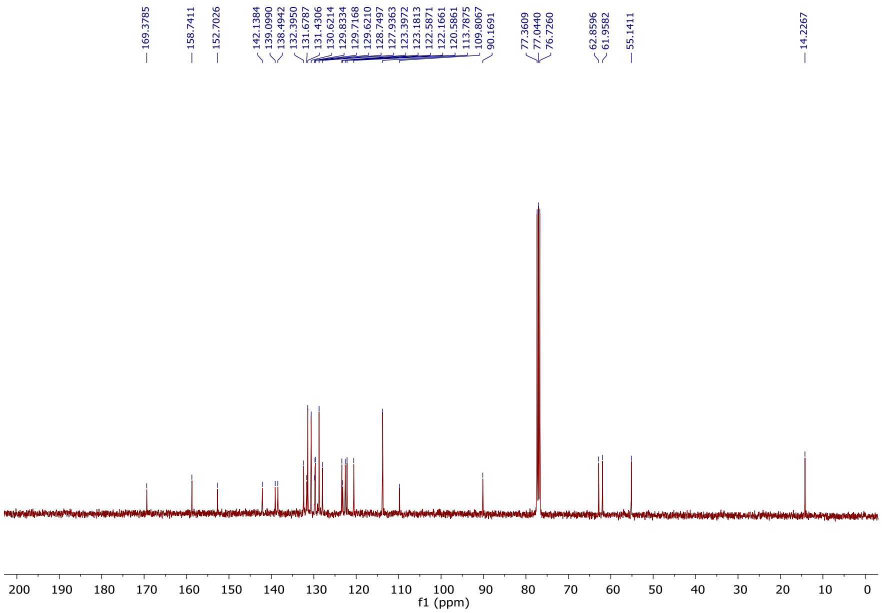

^{13}C NMR (100 MHz, $CDCl_3$) of compound **3i**

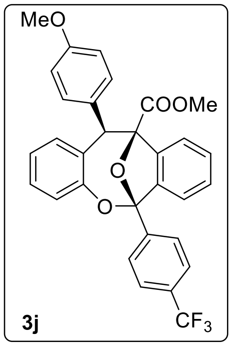

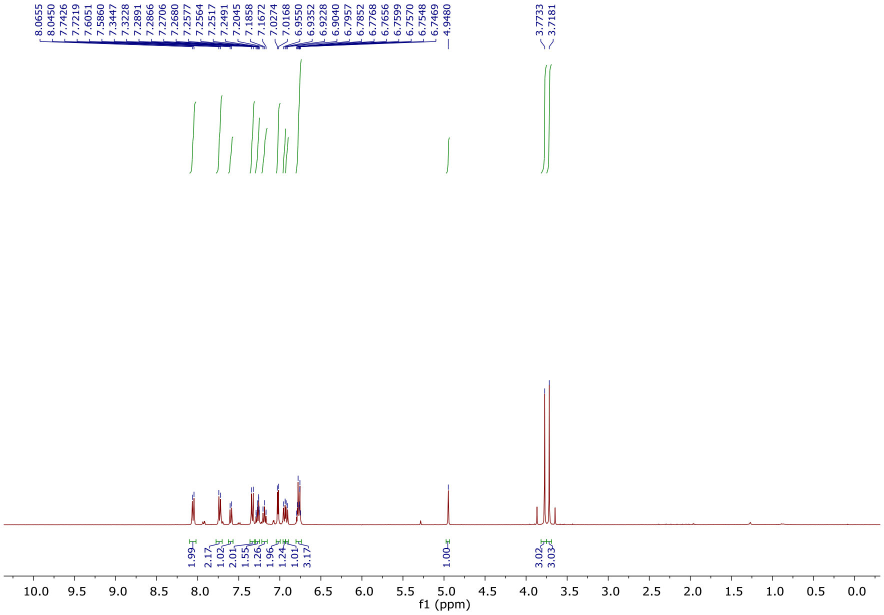

^{1}H NMR (400 MHz, $CDCl_3$) of compound **3j**

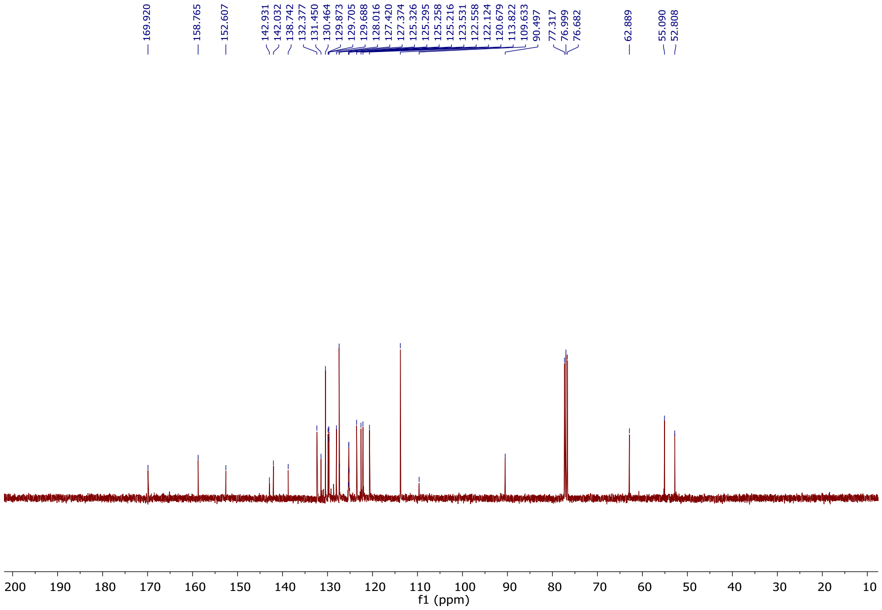

^{13}C NMR (100 MHz, $CDCl_3$) of compound **3j**

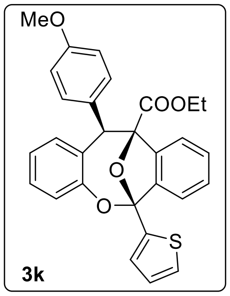

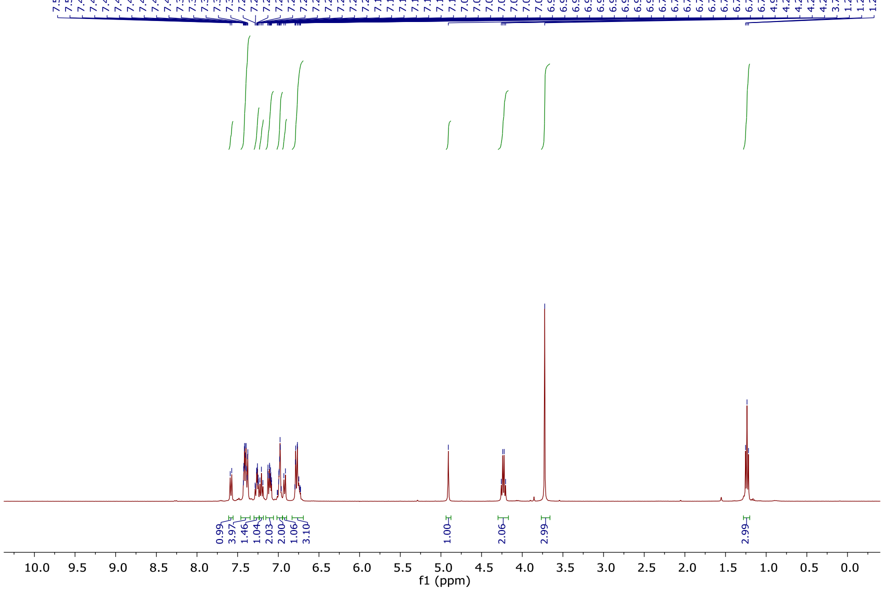

^{1}H NMR (400 MHz, $CDCl_3$) of compound **3k**

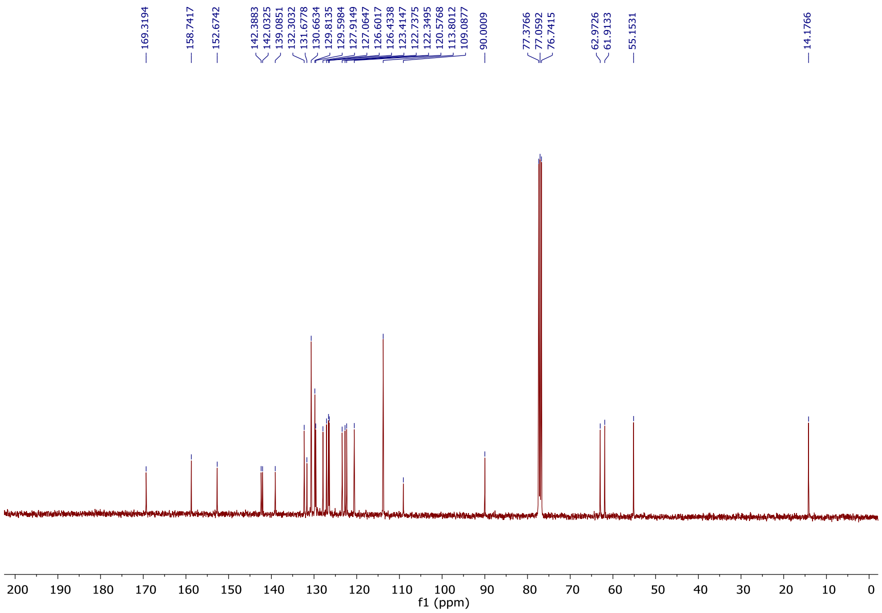

^{13}C NMR (100 MHz, $CDCl_3$) of compound **3k**

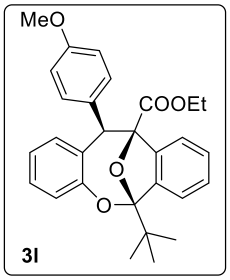

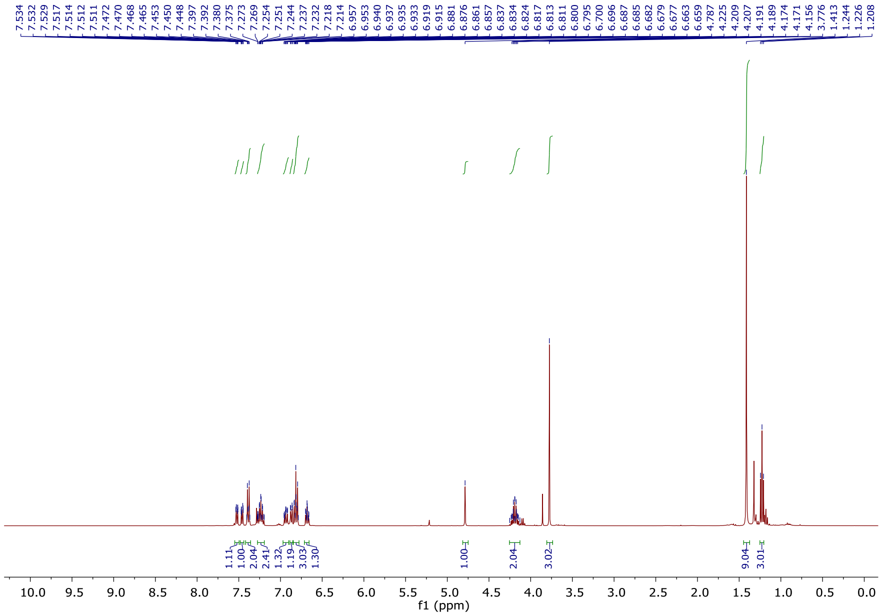

^{1}H NMR (400 MHz, $CDCl_3$) of compound **3l**

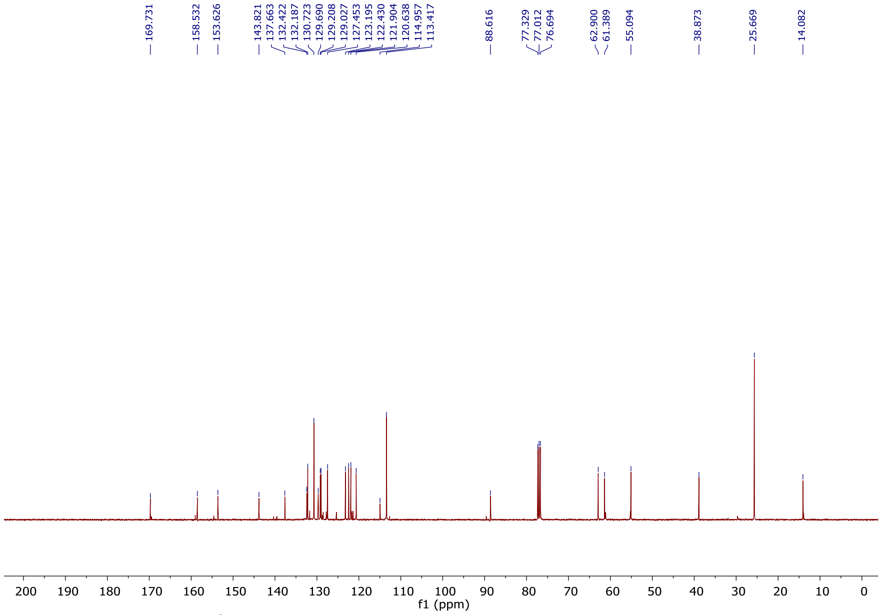

^{13}C NMR (100 MHz, $CDCl_3$) of compound **3l**

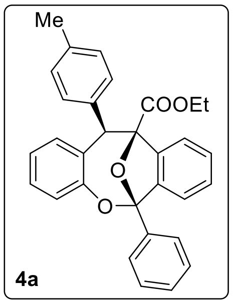

4a

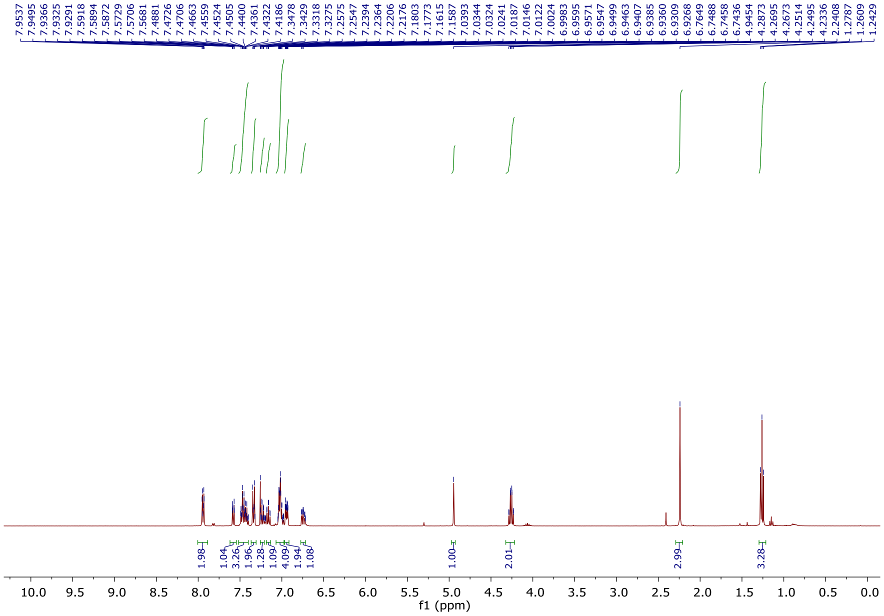

^{1}H NMR (400 MHz, $CDCl_3$) of compound **4a**

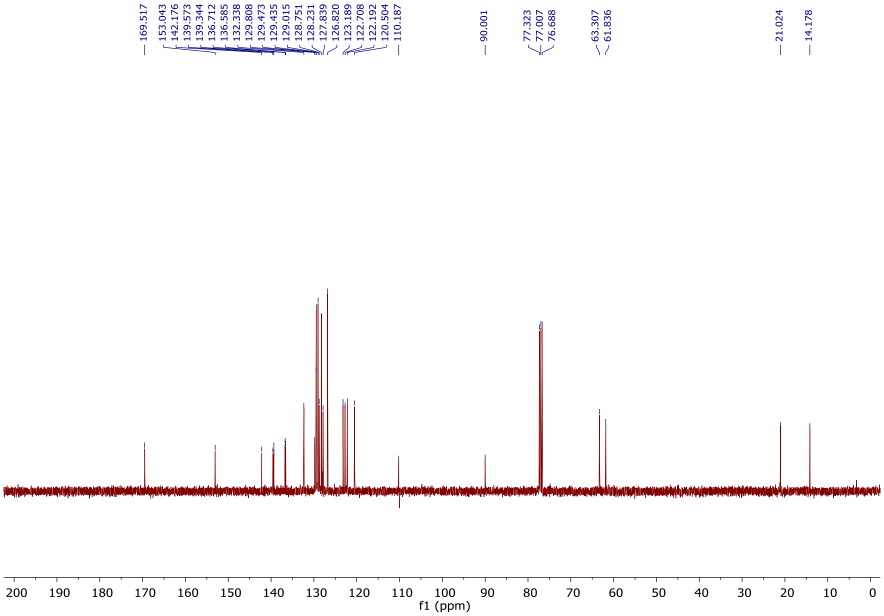

^{13}C NMR (100 MHz, $CDCl_3$) of compound **4a**

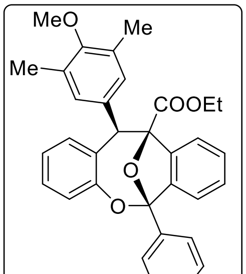

4b

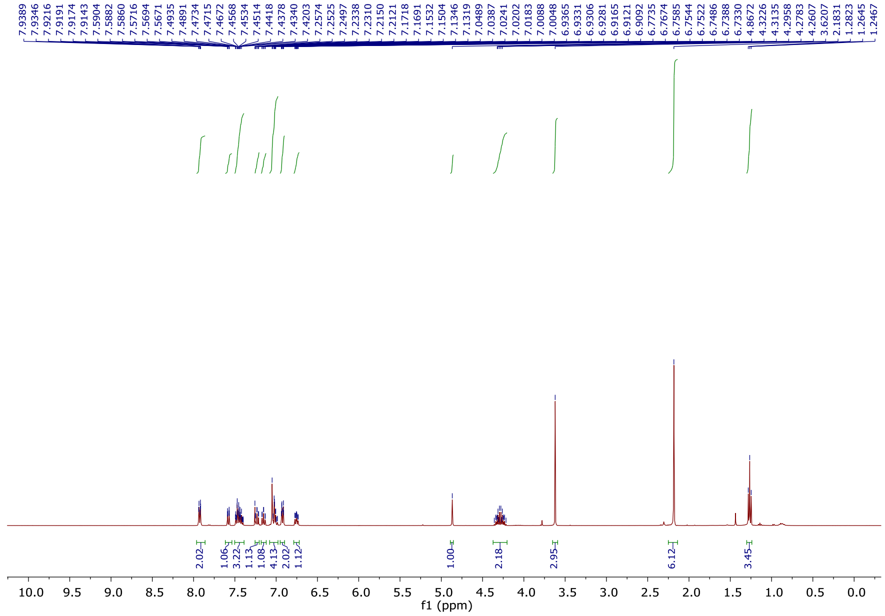

^{1}H NMR (400 MHz, CDCl$_3$) of compound **4b**

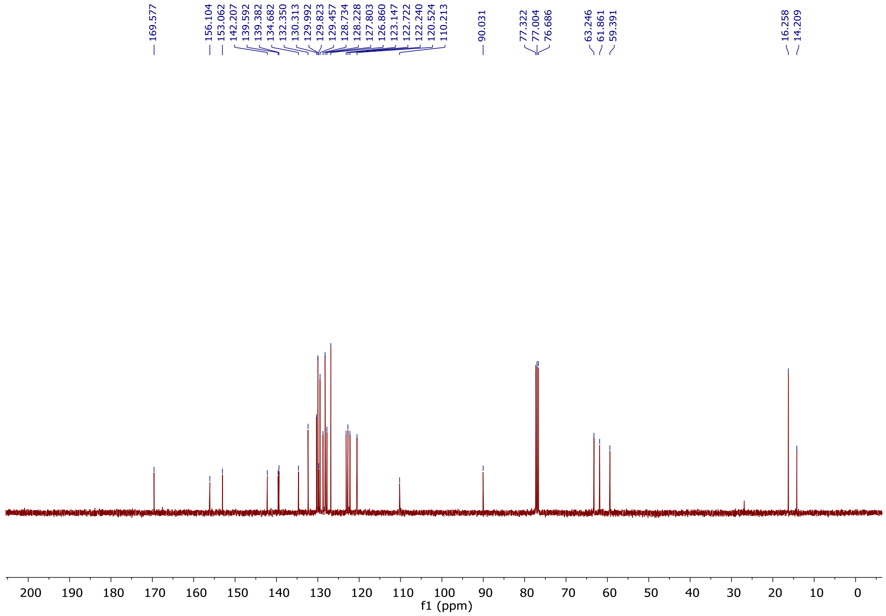

^{13}C NMR (100 MHz, CDCl$_3$) of compound **4b**

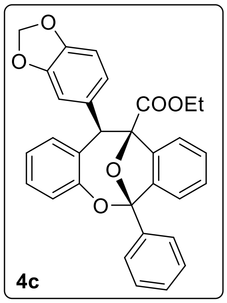

4c

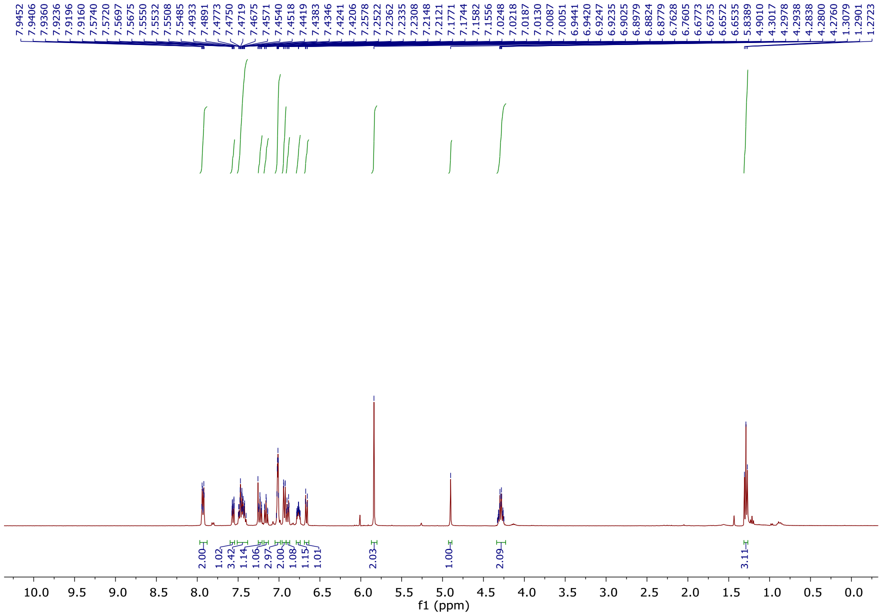

^{1}H NMR (400 MHz, $CDCl_3$) of compound **4c**

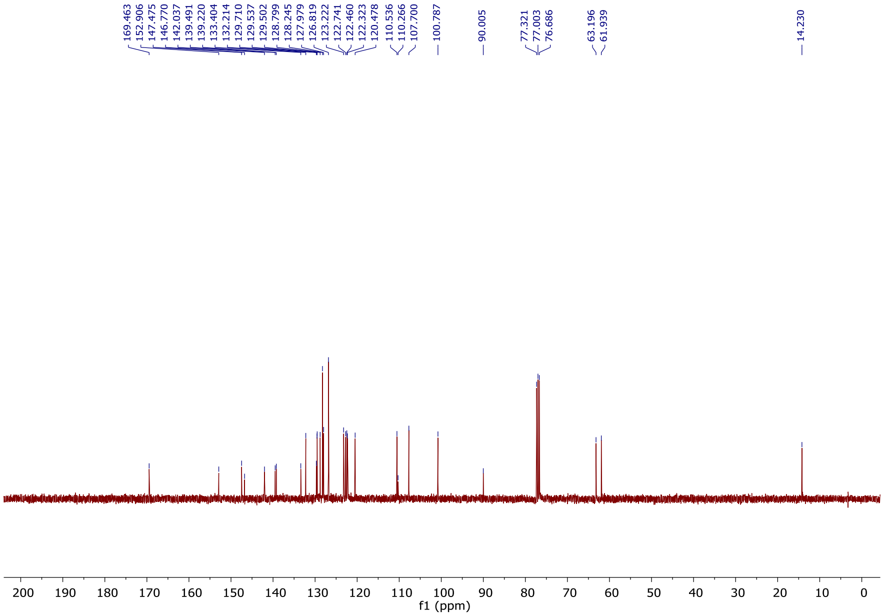

^{13}C NMR (100 MHz, $CDCl_3$) of compound **4c**

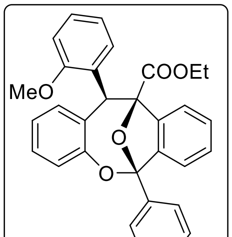

4d

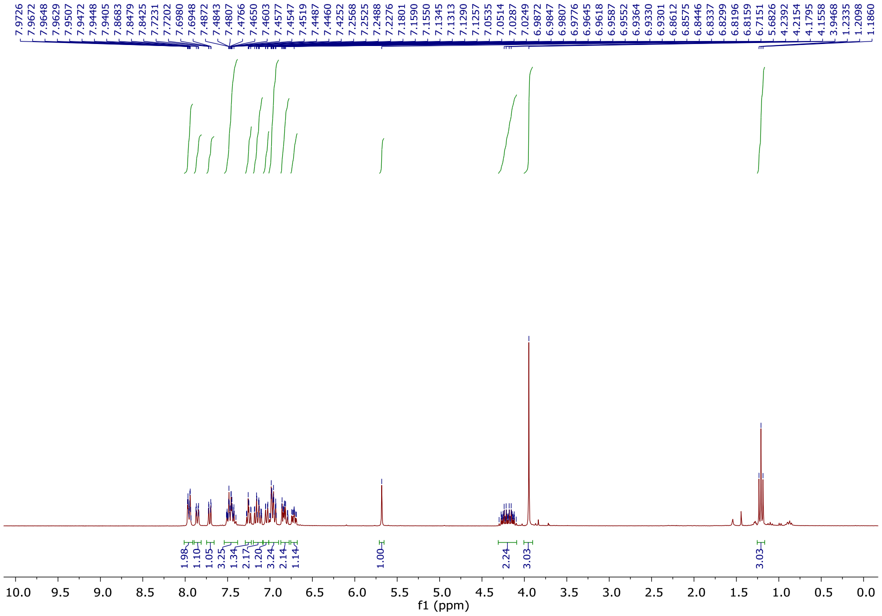

^{1}H NMR (300 MHz, $CDCl_3$) of compound **4d**

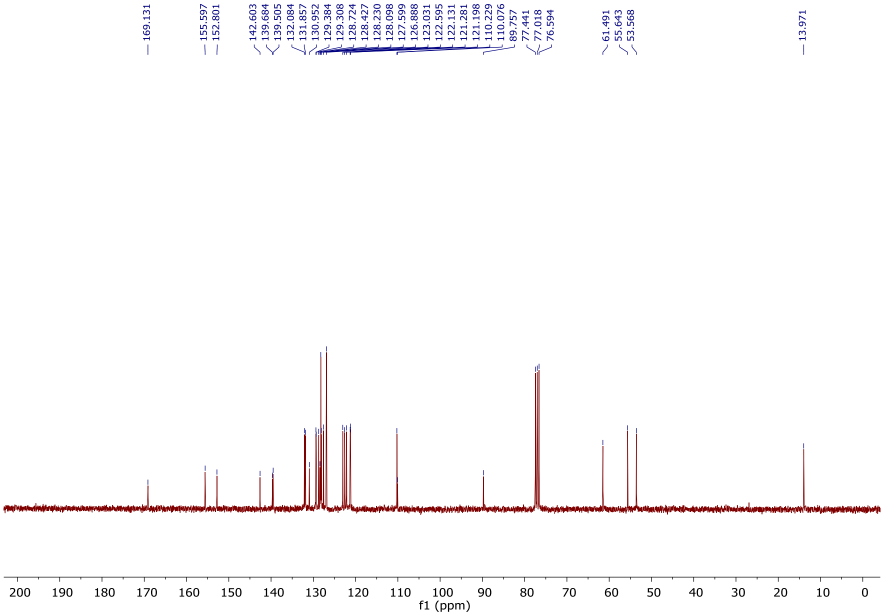

^{13}C NMR (75 MHz, $CDCl_3$) of compound **4d**

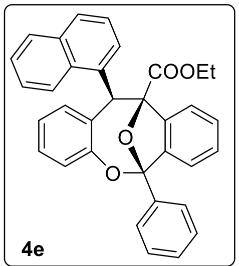

4e

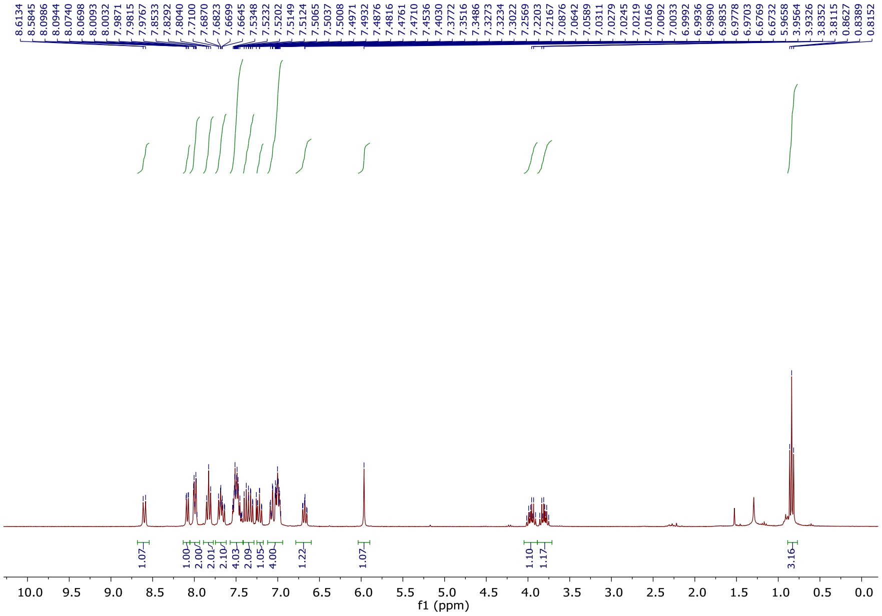

^{1}H NMR (300 MHz, $CDCl_3$) of compound **4e**

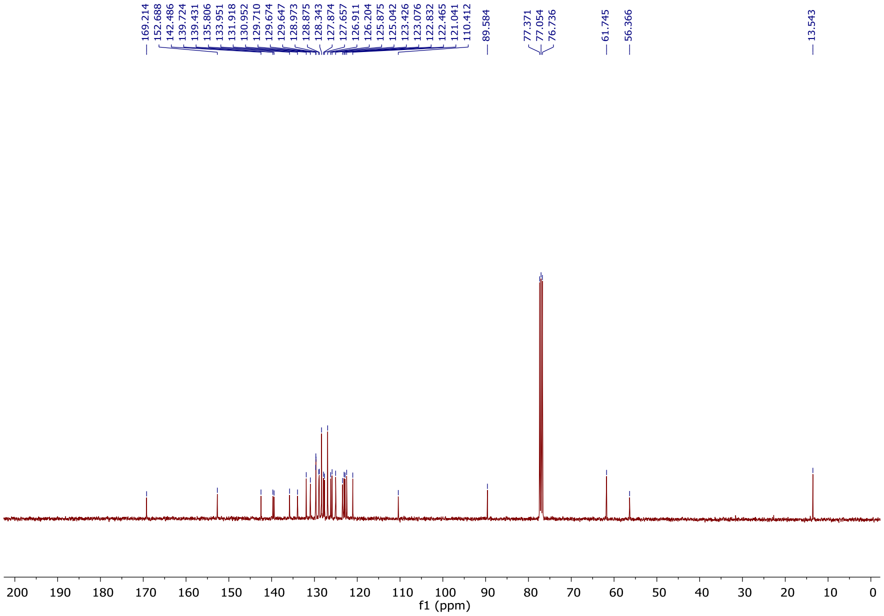

^{13}C NMR (100 MHz, $CDCl_3$) of compound **4e**

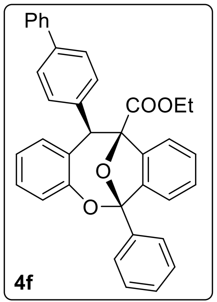

4f

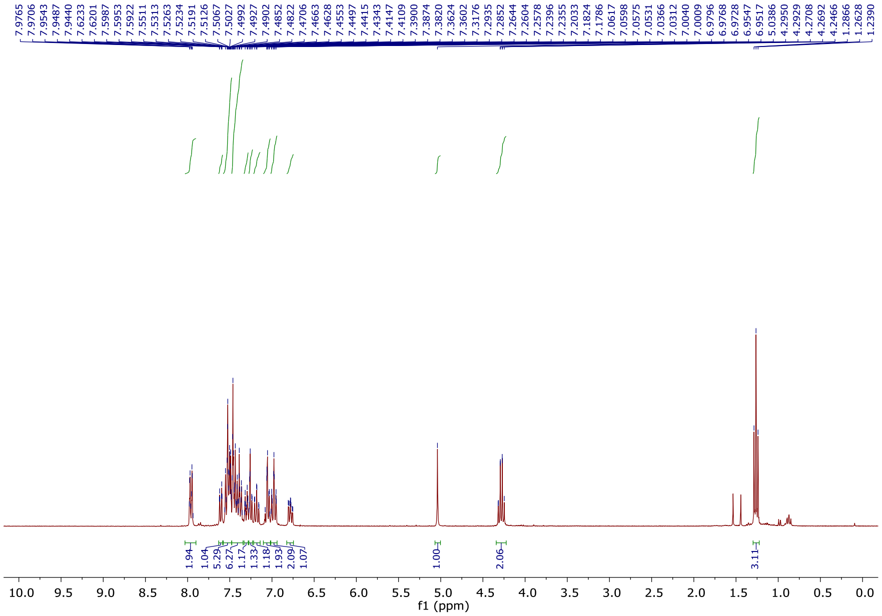

^{1}H NMR (300 MHz, $CDCl_3$) of compound **4f**

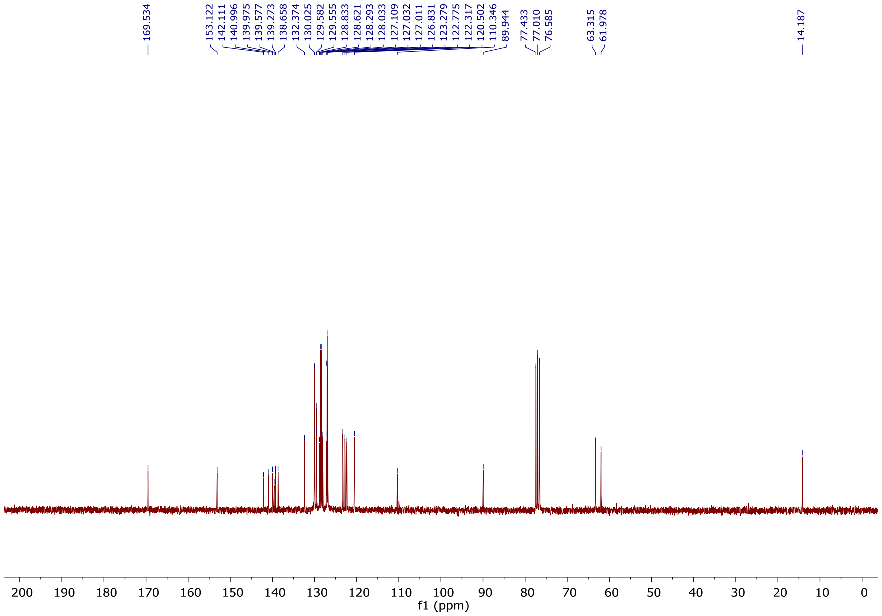

^{13}C NMR (75 MHz, $CDCl_3$) of compound **4f**

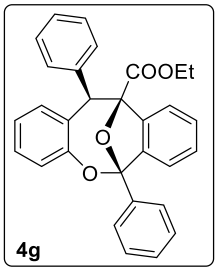

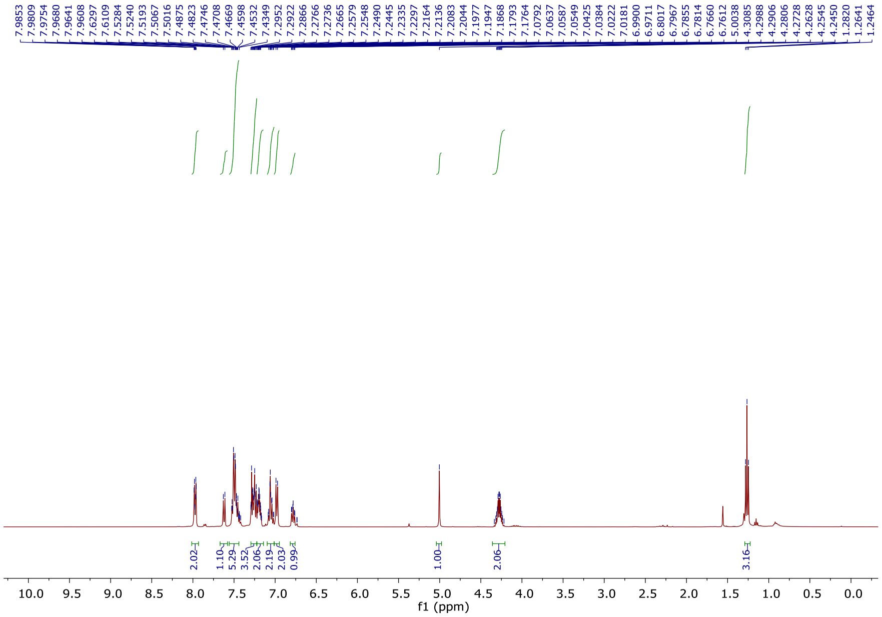

^{1}H NMR (400 MHz, $CDCl_3$) of compound **4g**

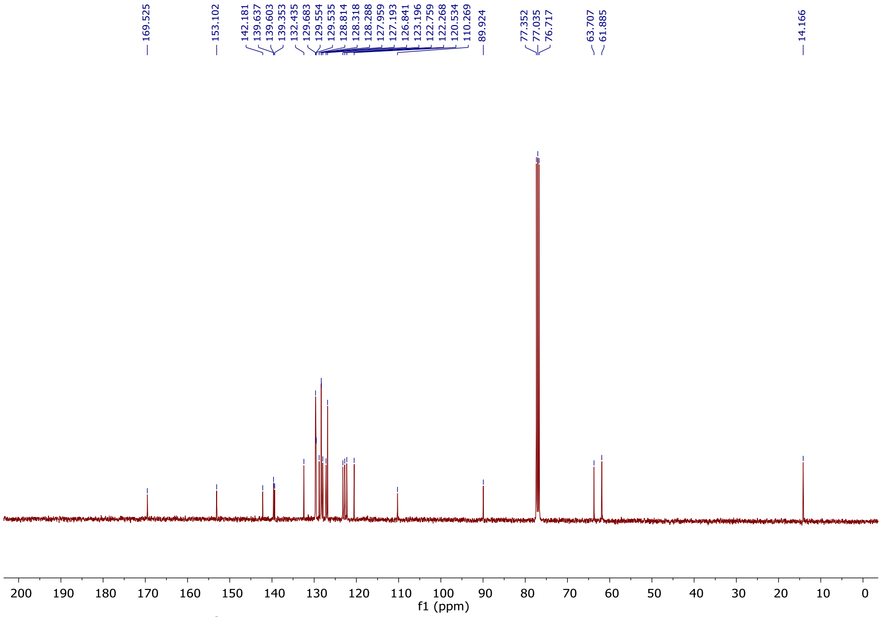

^{13}C NMR (100 MHz, $CDCl_3$) of compound **4g**

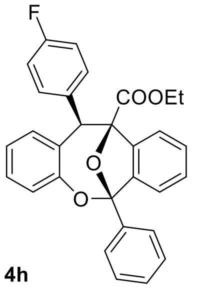

4h

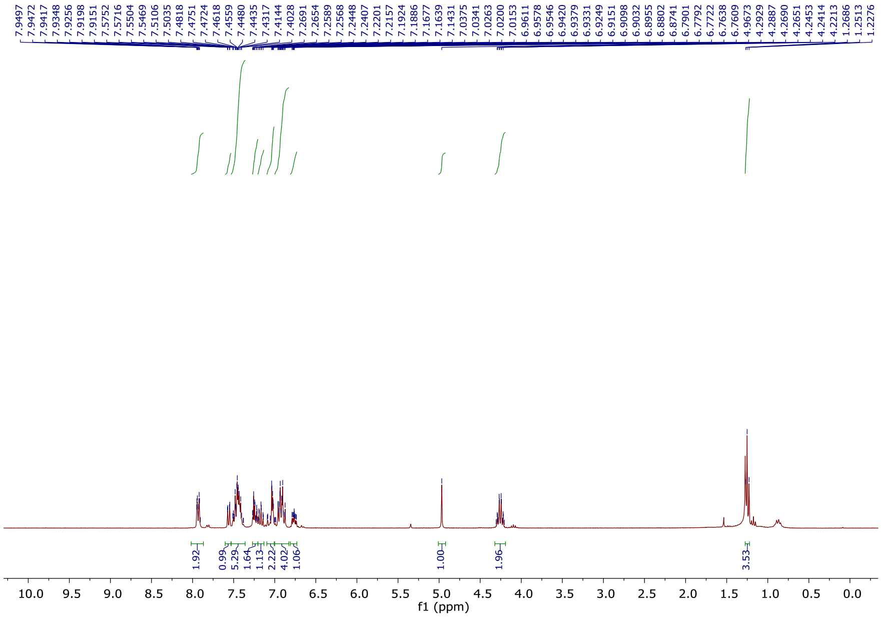

^{1}H NMR (300 MHz, $CDCl_3$) of compound **4h**

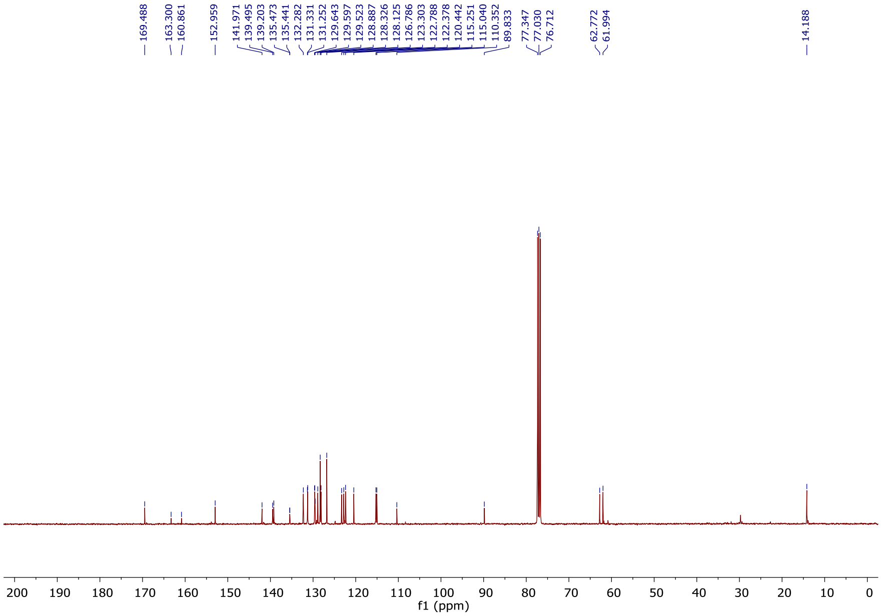

^{13}C NMR (100 MHz, $CDCl_3$) of compound **4h**

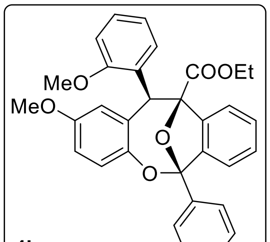

4i

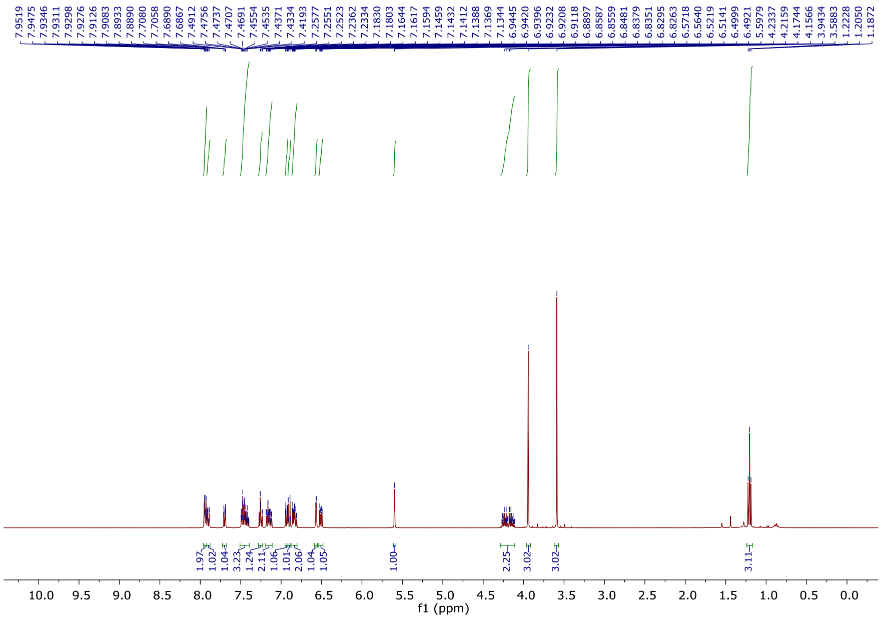

^{1}H NMR (400 MHz, $CDCl_3$) of compound **4i**

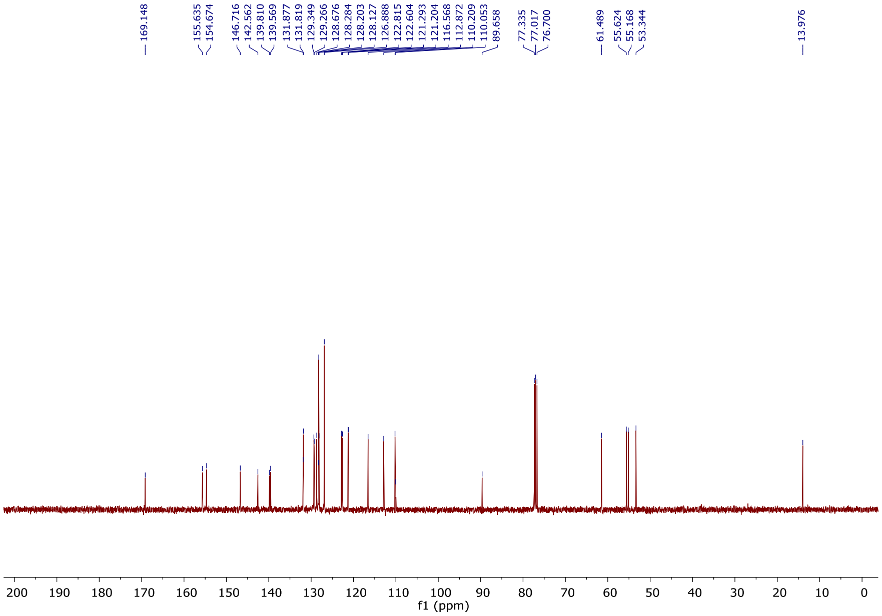

^{13}C NMR (100 MHz, $CDCl_3$) of compound **4i**

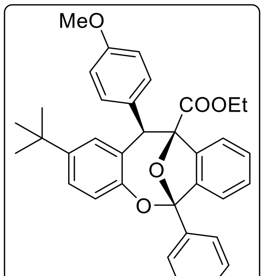

4j

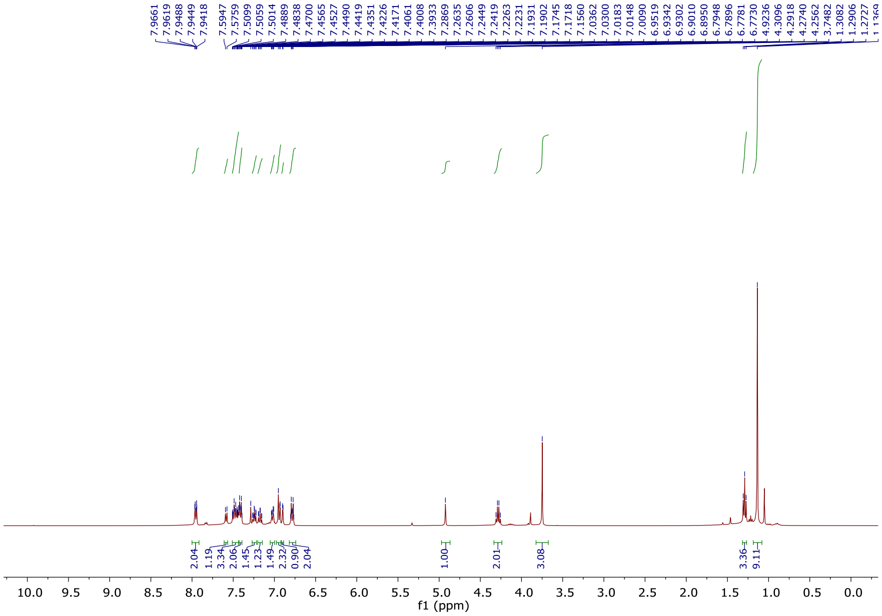

^{1}H NMR (400 MHz, $CDCl_3$) of compound **4j**

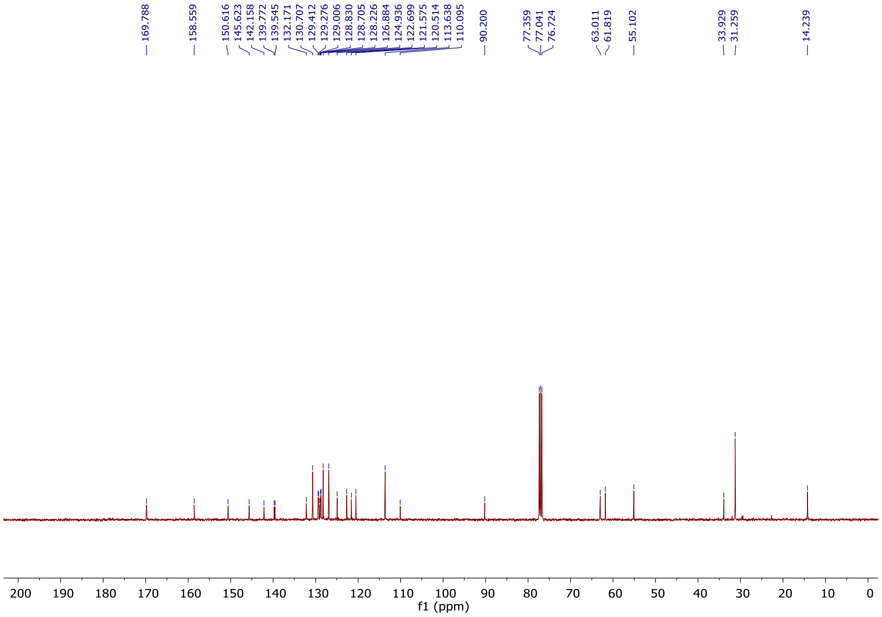

^{13}C NMR (100 MHz, $CDCl_3$) of compound **4j**

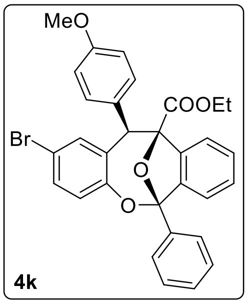

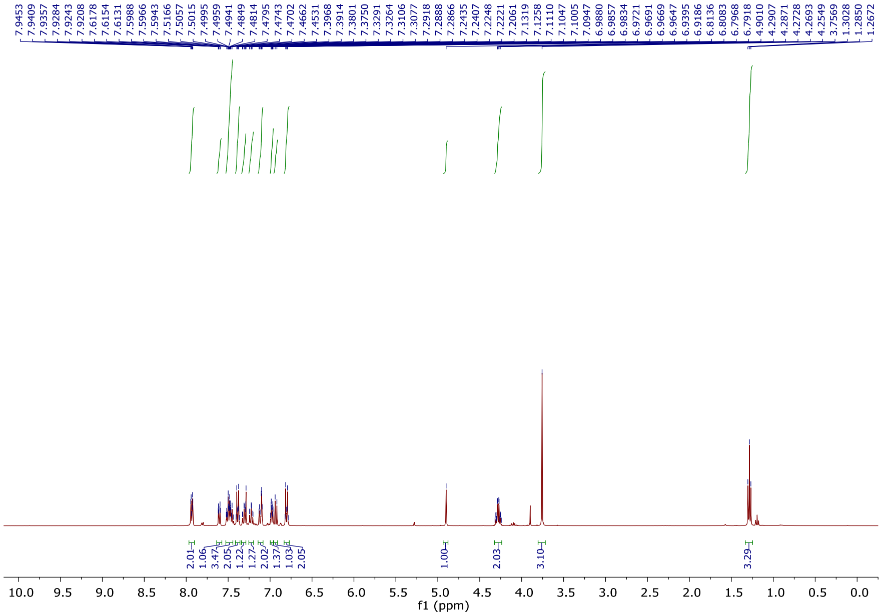

^{1}H NMR (400 MHz, $CDCl_3$) of compound **4k**

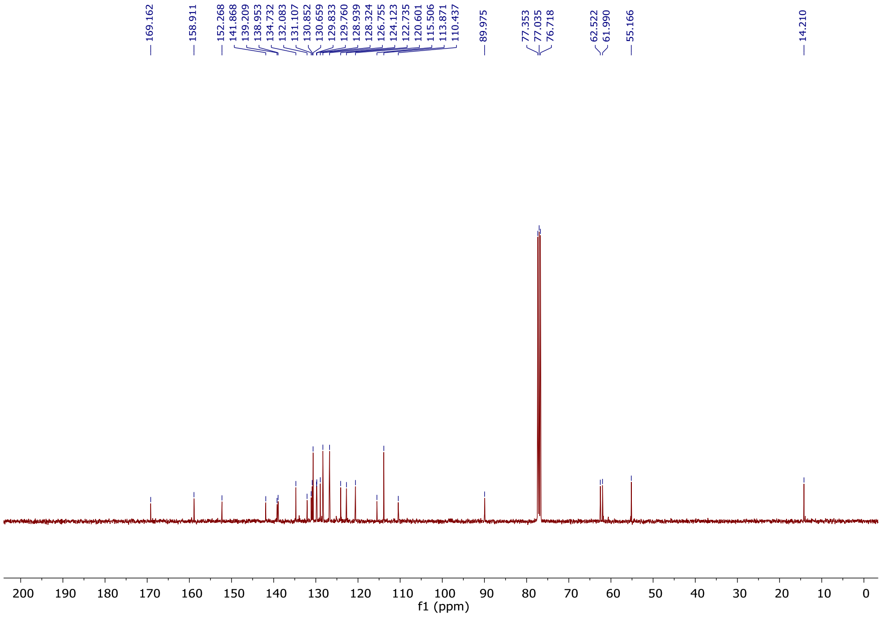

^{13}C NMR (100 MHz, $CDCl_3$) of compound **4k**

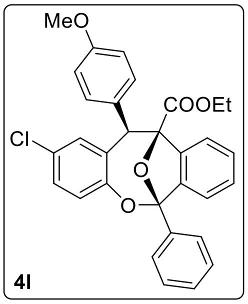

4l

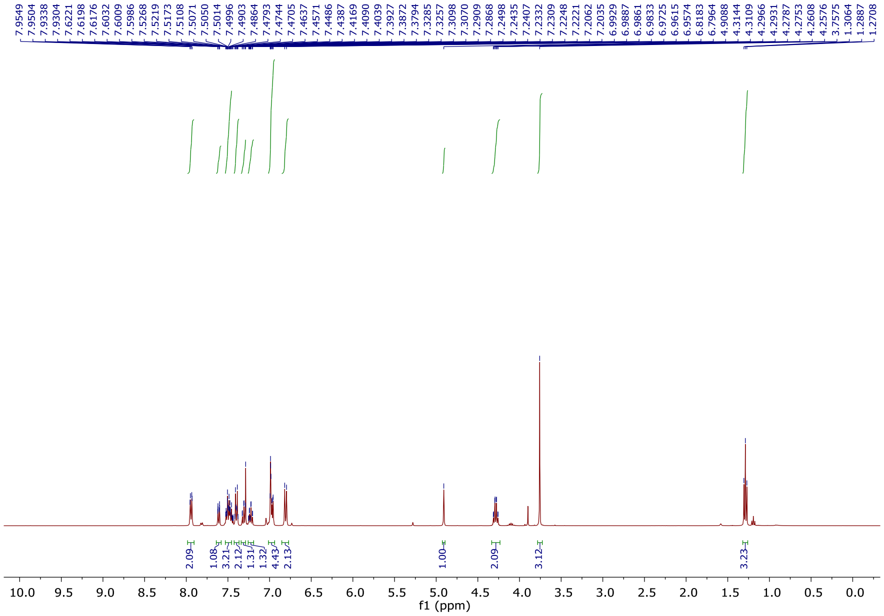

^{1}H NMR (400 MHz, $CDCl_3$) of compound **4l**

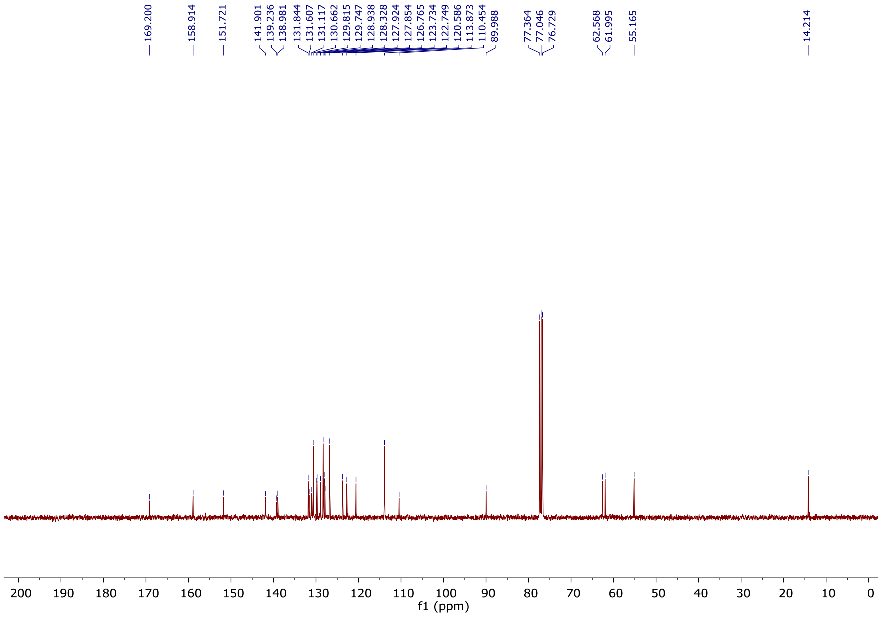

^{13}C NMR (100 MHz, $CDCl_3$) of compound **4l**

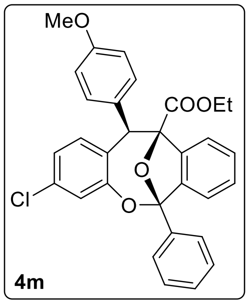

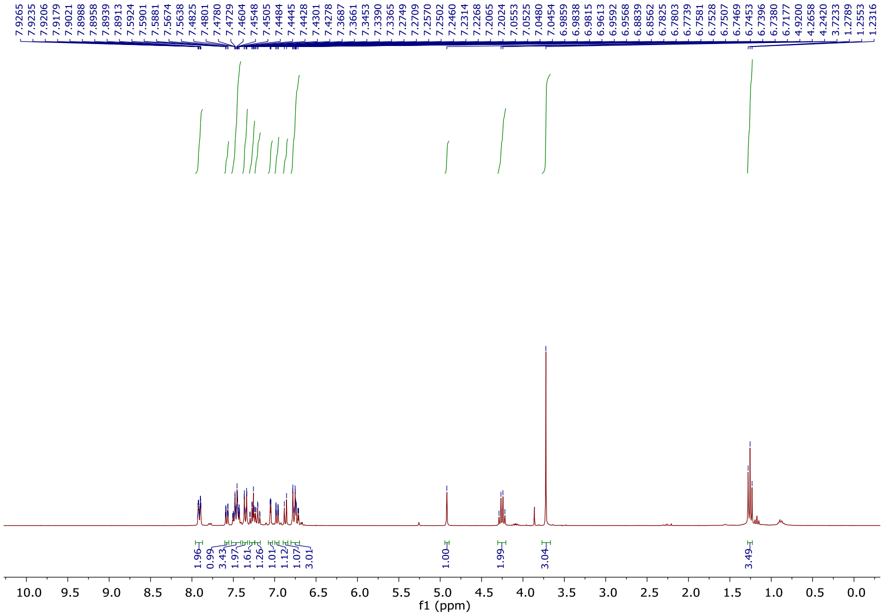

^{1}H NMR (300 MHz, $CDCl_3$) of compound **4m**

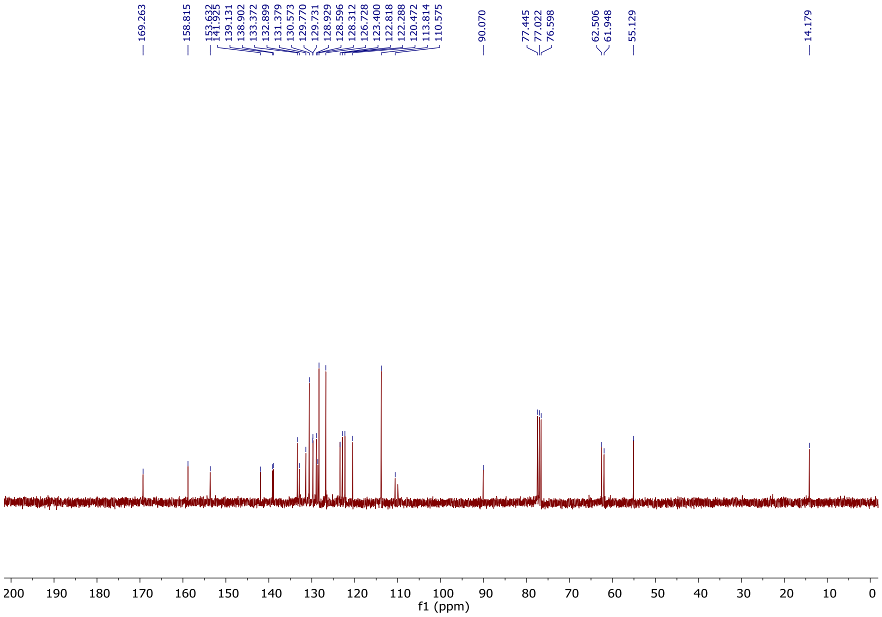

^{13}C NMR (75 MHz, $CDCl_3$) of compound **4m**

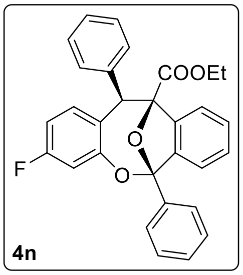

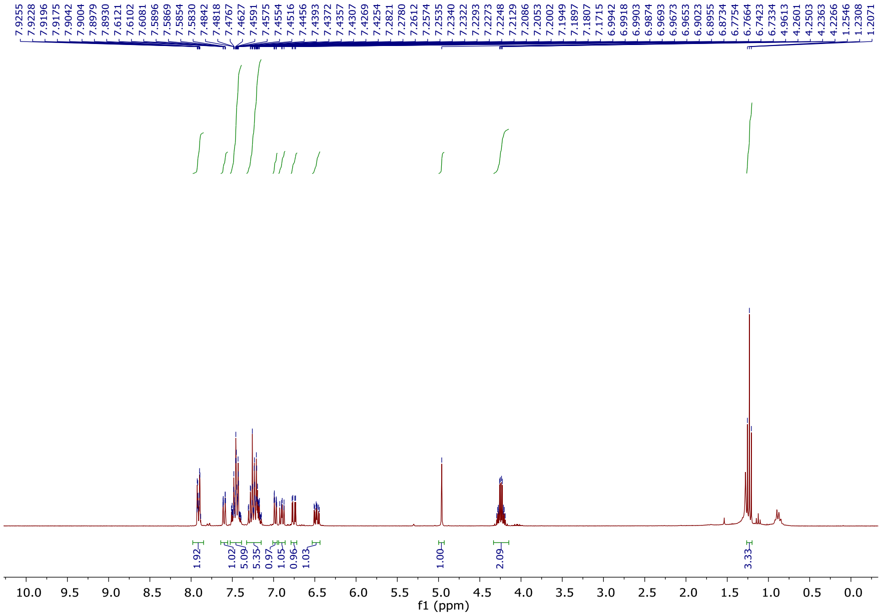

^{1}H NMR (300 MHz, $CDCl_3$) of compound **4n**

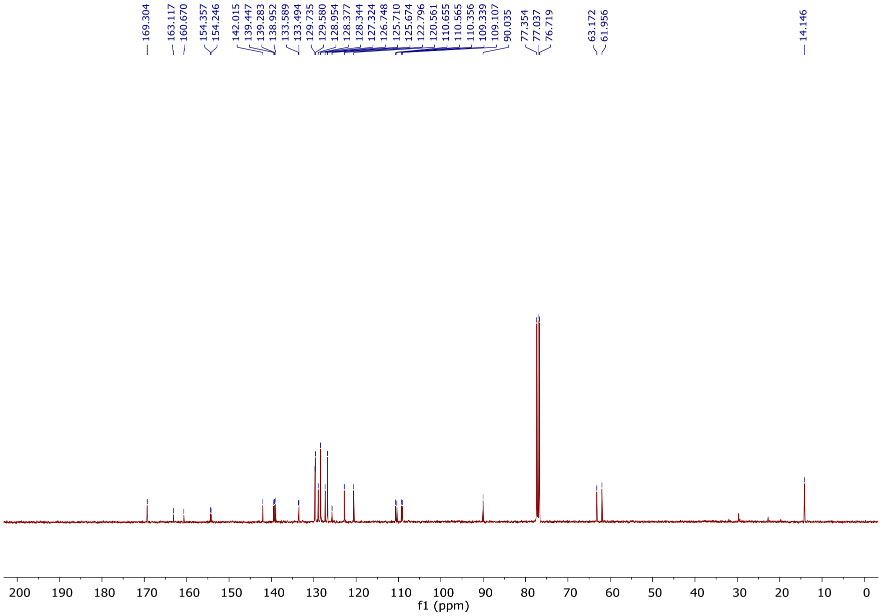

^{13}C NMR (100 MHz, $CDCl_3$) of compound **4n**

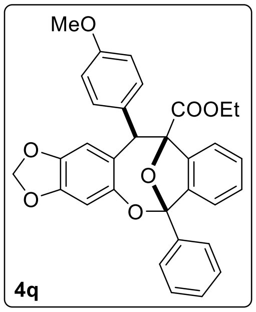

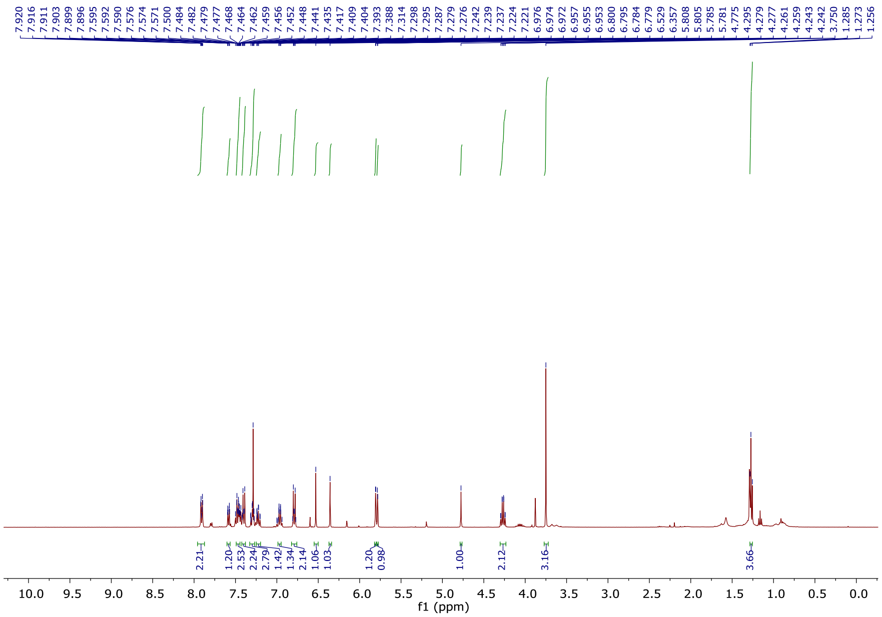

^{1}H NMR (400 MHz, $CDCl_3$) of compound **4q**

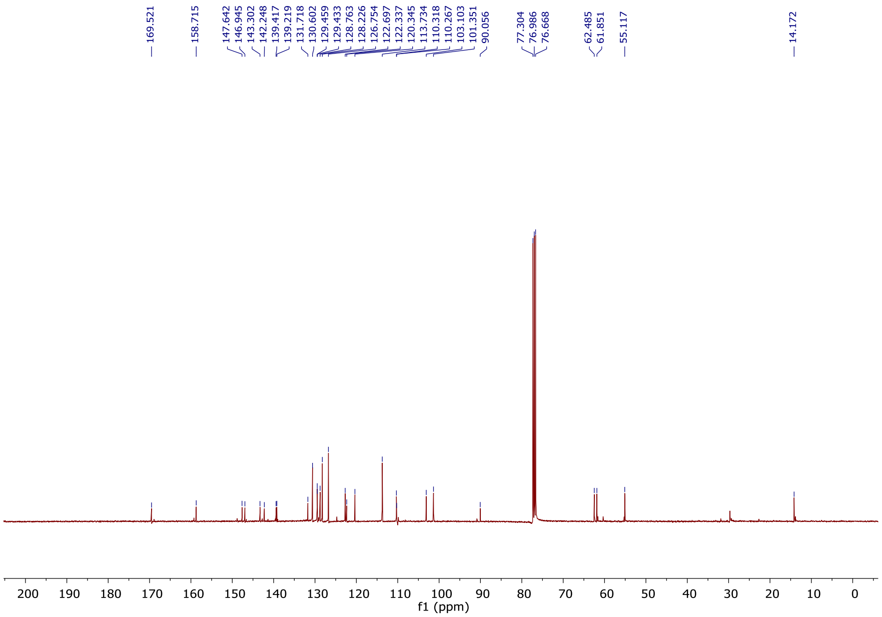

^{13}C NMR (100 MHz, $CDCl_3$) of compound **4q**

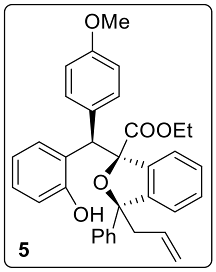

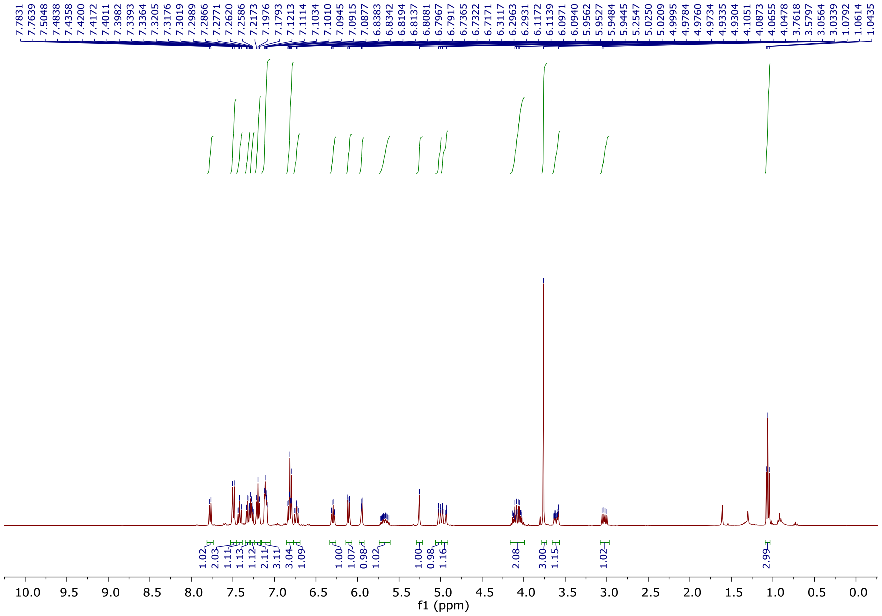

^{1}H NMR (400 MHz, CDCl$_3$) of compound **5**

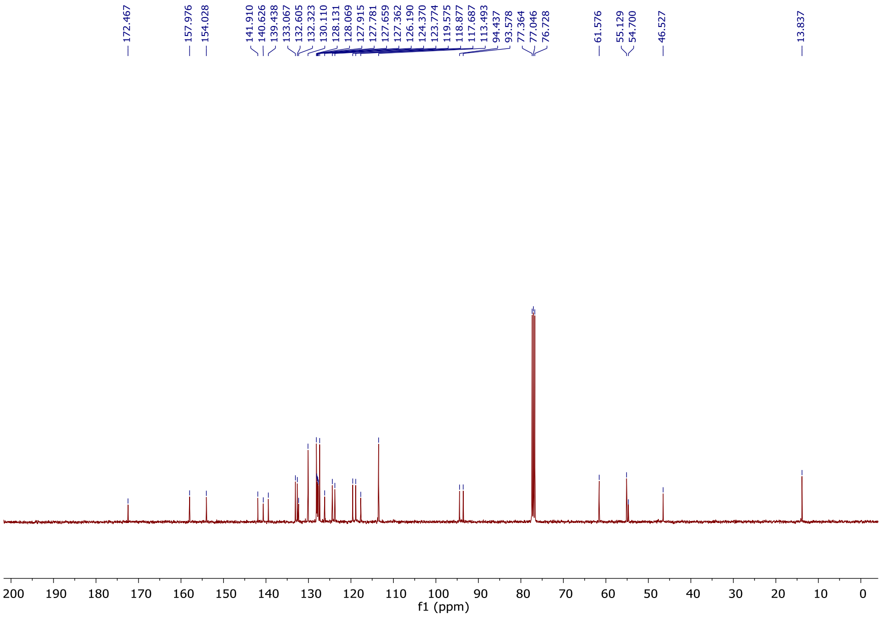

^{13}C NMR (100 MHz, CDCl$_3$) of compound **5**

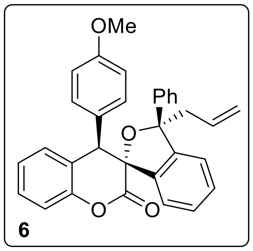

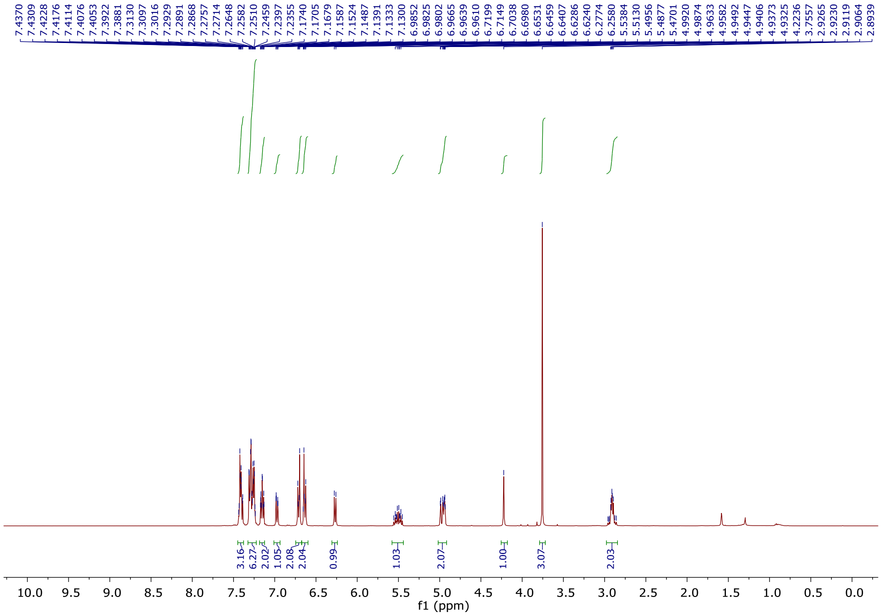

^{1}H NMR (400 MHz, $CDCl_3$) of compound **6**

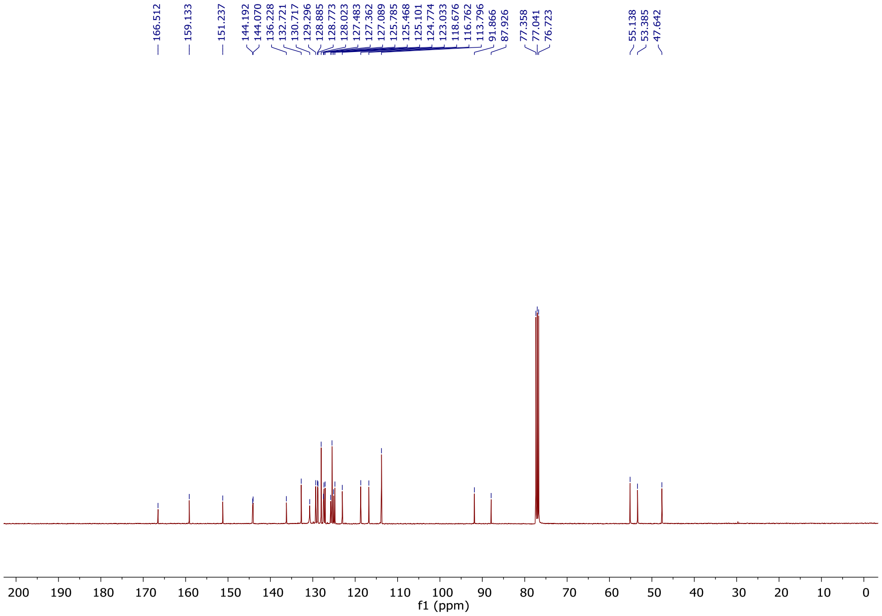

^{13}C NMR (100 MHz, $CDCl_3$) of compound **6**

HPLC Traces

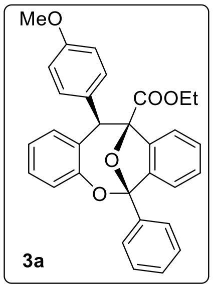

3a

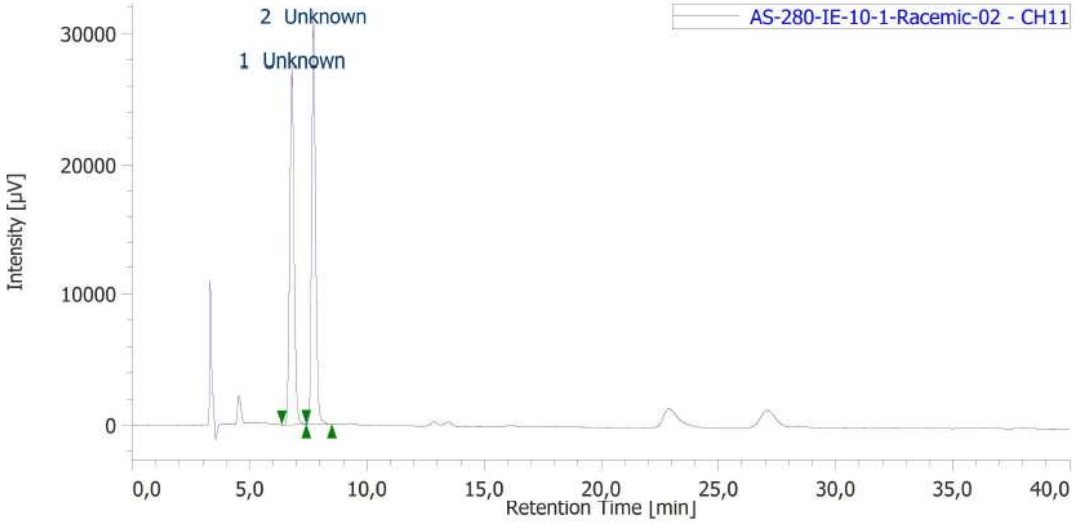

#	Peak Name	CH	tR [min]	Area [µV·sec]	Height [µV]	Area%	Height%	Quantity	NTP	Resolution	Symmetry Factor	Warning
1	Unknown	11	6,793	354791	27021	49,824	46,853	N/A	6369	2,918	1,124	
2	Unknown	11	7,720	357298	30651	50,176	53,147	N/A	10859	N/A	1,176	

HPLC data of compound (±)-**3a**

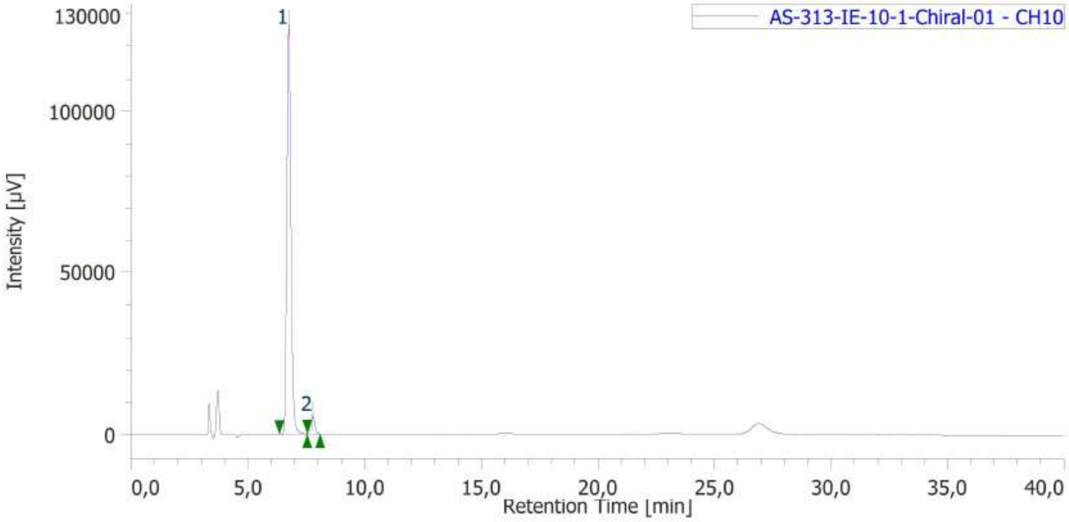

#	Peak Name	CH	tR [min]	Area [µV·sec]	Height [µV]	Area%	Height%	Quantity	NTP	Resolution	Symmetry Factor	Warning
1	Unknown	10	6,743	1555911	126418	95,787	95,666	N/A	7251	3,295	1,200	
2	Unknown	10	7,777	68427	5728	4,213	4,334	N/A	9932	N/A	1,056	

HPLC data of compound (-)-**3a**

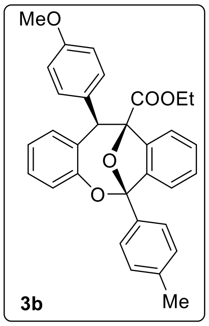

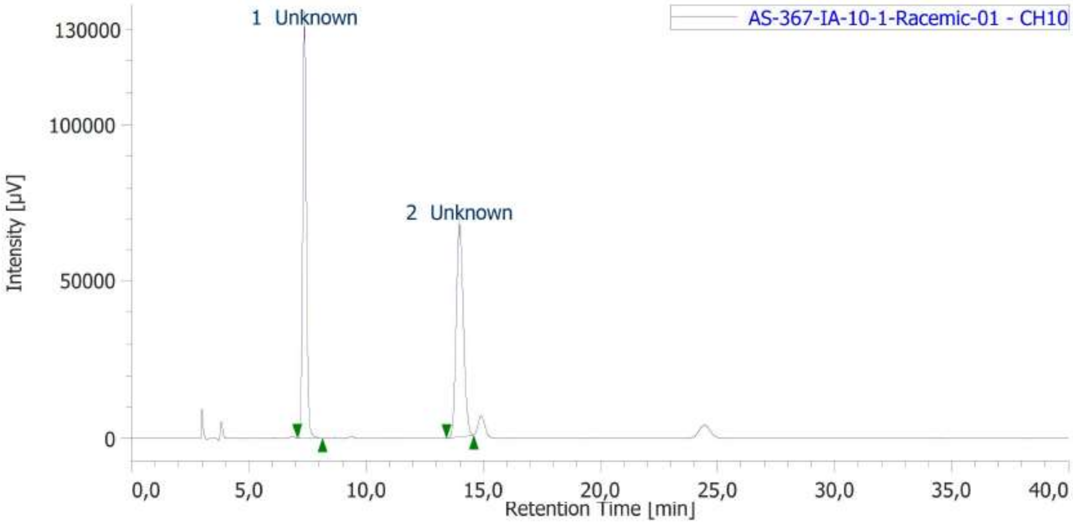

#	Peak Name	CH	tR [min]	Area [µV·sec]	Height [µV]	Area%	Height%	Quantity	NTP	Resolution	Symmetry Factor	Warning
1	Unknown	10	7,367	1472395	131140	50,590	65,930	N/A	10312	15,598	1,126	
2	Unknown	10	13,980	1438080	67769	49,410	34,070	N/A	9969	N/A	1,132	

HPLC data of compound (±)-**3b**

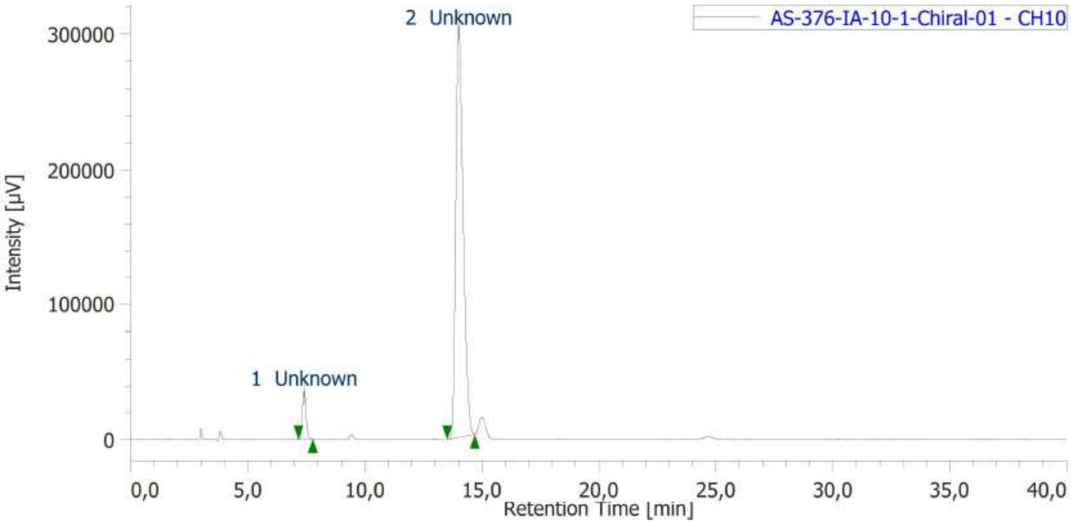

#	Peak Name	CH	tR [min]	Area [µV·sec]	Height [µV]	Area%	Height%	Quantity	NTP	Resolution	Symmetry Factor	Warning
1	Unknown	10	7,407	400168	36409	5,634	10,681	N/A	10575	15,237	1,104	
2	Unknown	10	14,000	6701998	304481	94,366	89,319	N/A	9334	N/A	1,391	

HPLC data of compound (-)-**3b**

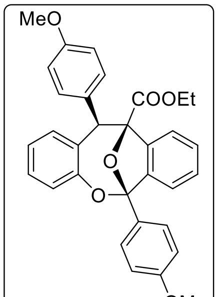

3c

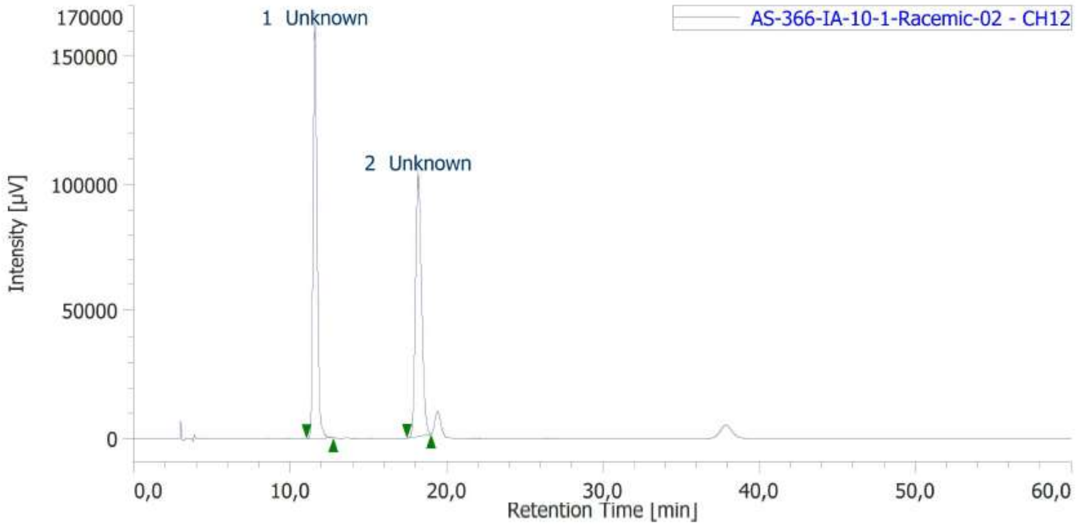

#	Peak Name	CH	tR [min]	Area [µV·sec]	Height [µV]	Area%	Height%	Quantity	NTP	Resolution	Symmetry Factor	Warning
1	Unknown	12	11,583	2905865	161863	50,930	61,152	N/A	10191	11,261	1,189	
2	Unknown	12	18,173	2799742	102826	49,070	38,848	N/A	10349	N/A	1,216	

HPLC data of compound (±)-**3c**

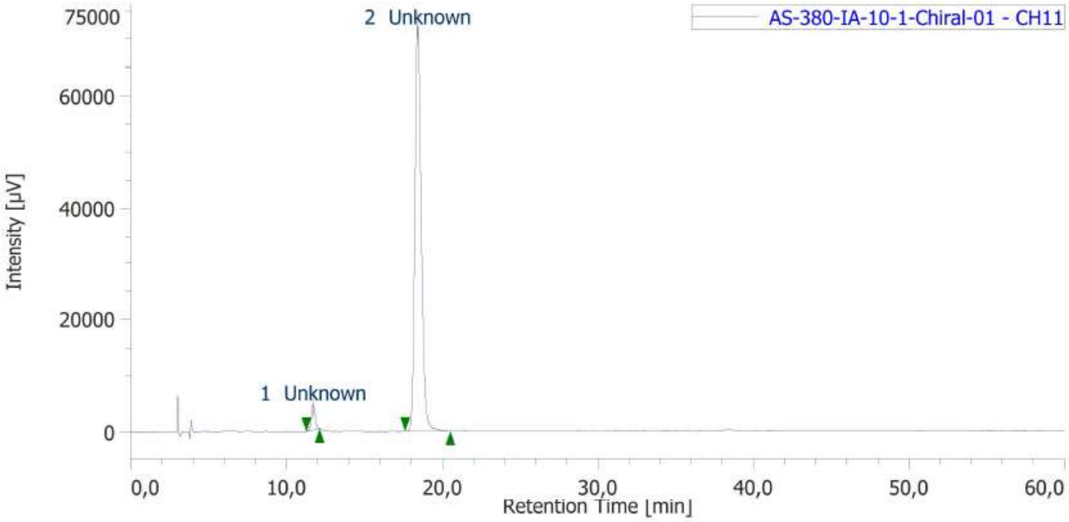

#	Peak Name	CH	tR [min]	Area [µV·sec]	Height [µV]	Area%	Height%	Quantity	NTP	Resolution	Symmetry Factor	Warning
1	Unknown	11	11,710	76001	4530	3,555	5,895	N/A	10710	11,346	1,015	
2	Unknown	11	18,410	2061726	72311	96,445	94,105	N/A	10131	N/A	1,222	

HPLC data of compound (-)-**3c**

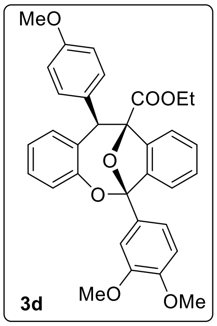

3d

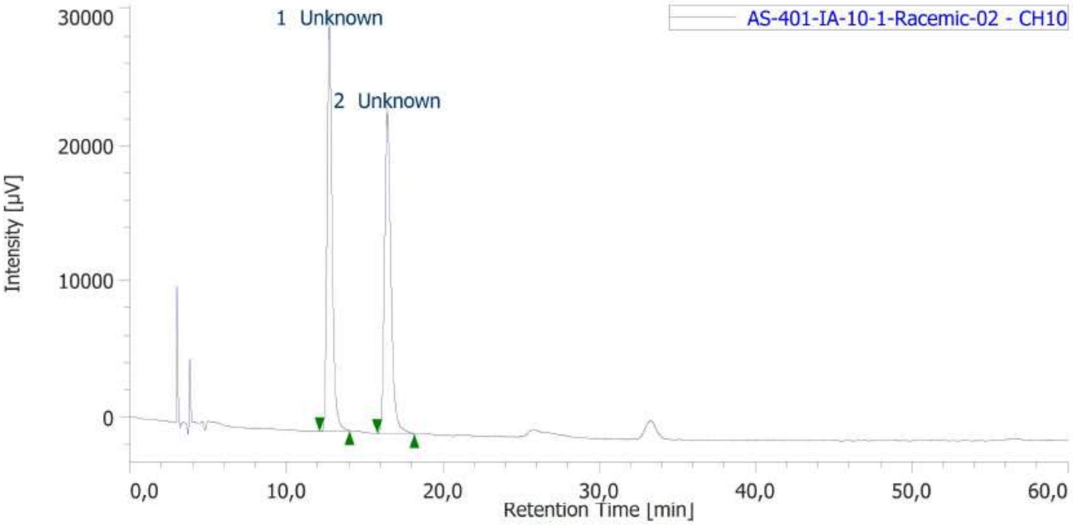

#	Peak Name	CH	tR [min]	Area [µV·sec]	Height [µV]	Area%	Height%	Quantity	NTP	Resolution	Symmetry Factor	Warning
1	Unknown	10	12,743	676501	29765	49,818	55,762	N/A	7984	5,729	1,319	
2	Unknown	10	16,440	681438	23614	50,182	44,238	N/A	8259	N/A	1,295	

HPLC data of compound (±)-**3d**

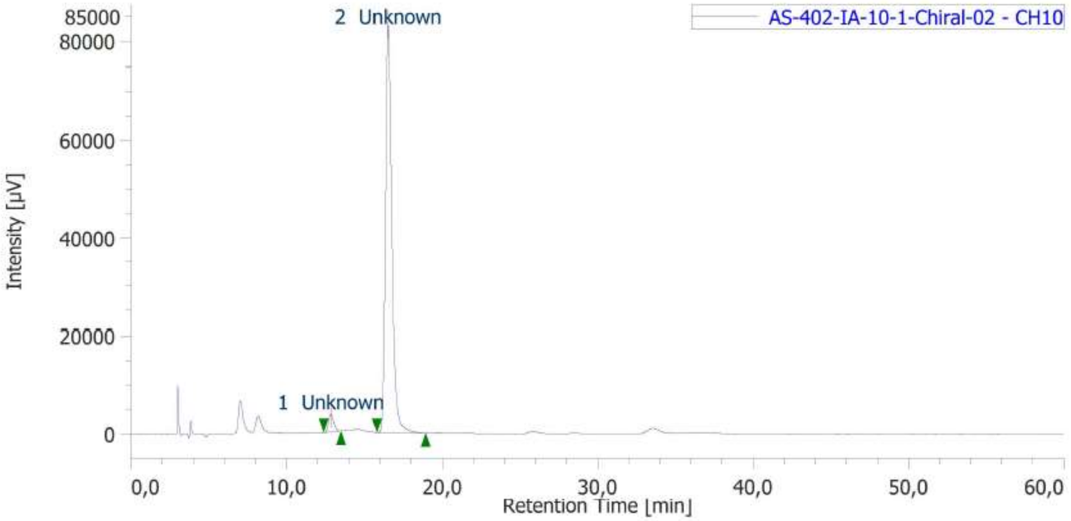

#	Peak Name	CH	tR [min]	Area [µV·sec]	Height [µV]	Area%	Height%	Quantity	NTP	Resolution	Symmetry Factor	Warning
1	Unknown	10	12,843	82752	3648	3,375	4,210	N/A	7616	5,643	1,244	
2	Unknown	10	16,517	2369110	83023	96,625	95,790	N/A	8496	N/A	1,323	

HPLC data of compound (-)-**3d**

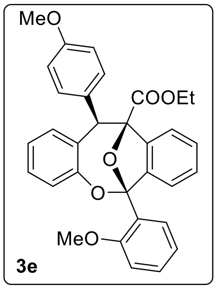

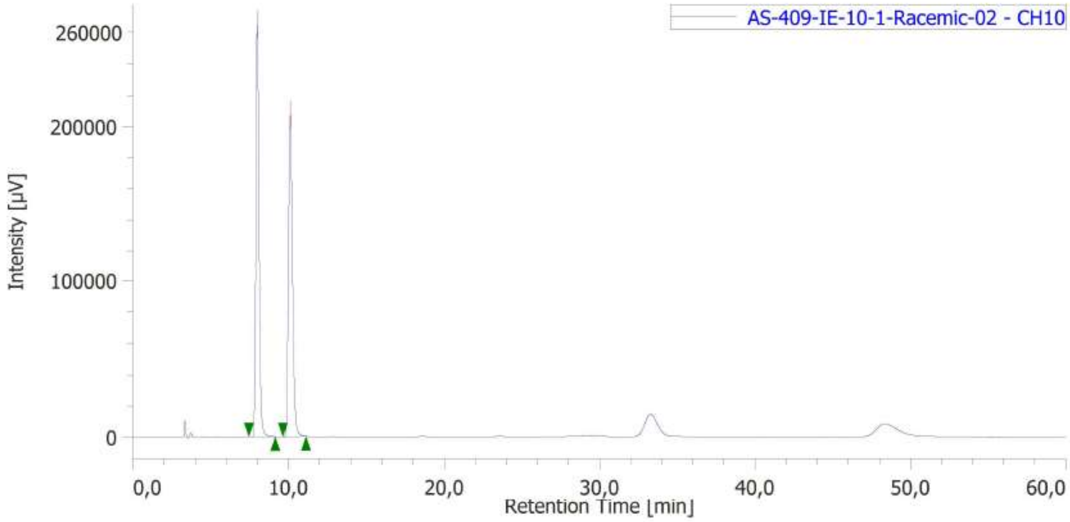

#	Peak Name	CH	tR [min]	Area [µV·sec]	Height [µV]	Area%	Height%	Quantity	NTP	Resolution	Symmetry Factor	Warning
1	Unknown	10	7,983	3749491	264392	49,974	56,022	N/A	7741	5,134	1,244	
2	Unknown	10	10,107	3753359	207552	50,026	43,978	N/A	7512	N/A	1,248	

HPLC data of compound (±)-**3e**

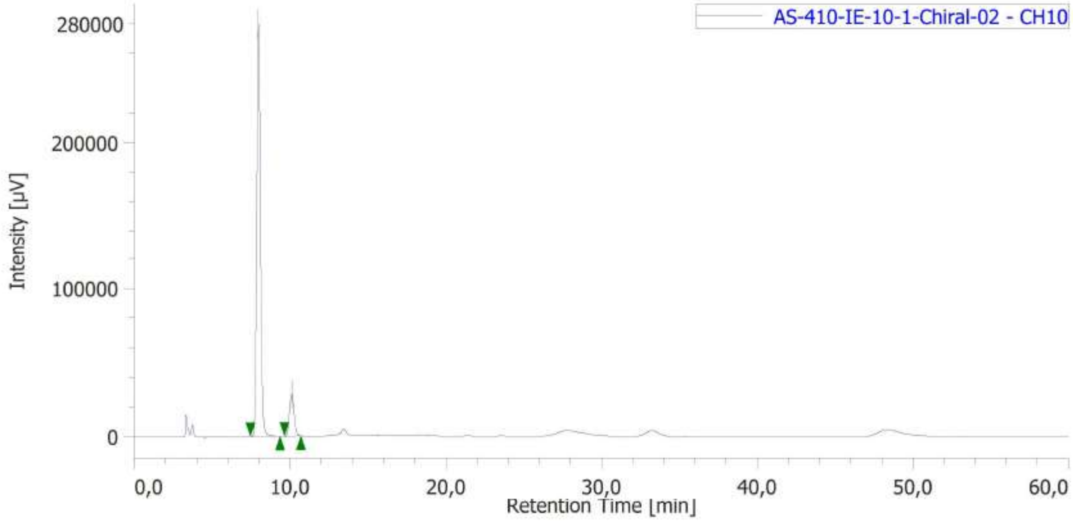

#	Peak Name	CH	tR [min]	Area [µV·sec]	Height [µV]	Area%	Height%	Quantity	NTP	Resolution	Symmetry Factor	Warning
1	Unknown	10	7,963	4644236	279639	88,610	90,700	N/A	5575	4,386	1,121	
2	Unknown	10	10,097	596997	28671	11,390	9,300	N/A	5416	N/A	1,110	

HPLC data of compound (-)-**3e**

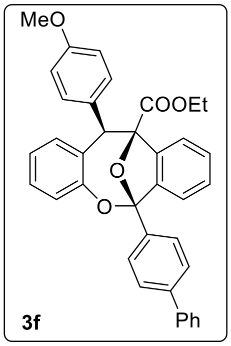

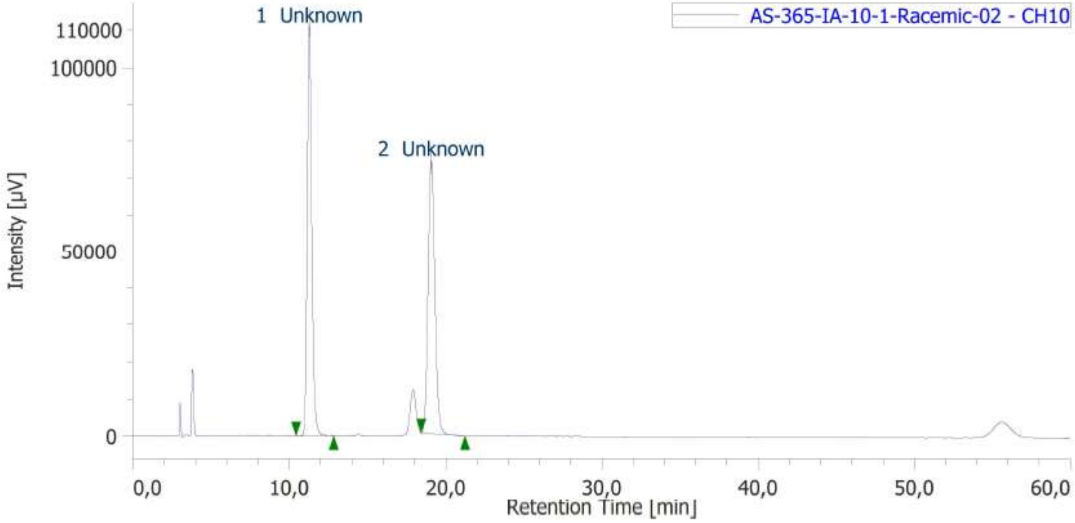

#	Peak Name	CH	tR [min]	Area [µV·sec]	Height [µV]	Area%	Height%	Quantity	NTP	Resolution	Symmetry Factor	Warning
1	Unknown	10	11,267	2290794	111735	50,721	60,121	N/A	7402	11,937	1,247	
2	Unknown	10	19,063	2225636	74115	49,279	39,879	N/A	9413	N/A	1,129	

HPLC data of compound (±)-**3f**

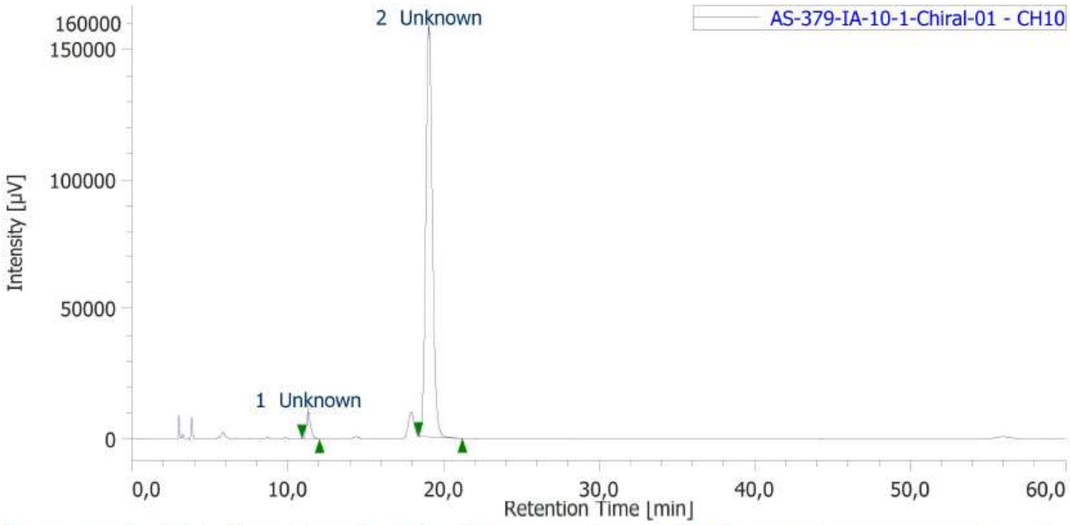

#	Peak Name	CH	tR [min]	Area [µV·sec]	Height [µV]	Area%	Height%	Quantity	NTP	Resolution	Symmetry Factor	Warning
1	Unknown	10	11,310	203742	10328	4,159	6,136	N/A	7897	12,100	1,231	
2	Unknown	10	19,053	4695618	157997	95,841	93,864	N/A	9689	N/A	1,141	

HPLC data of compound (-)-**3f**

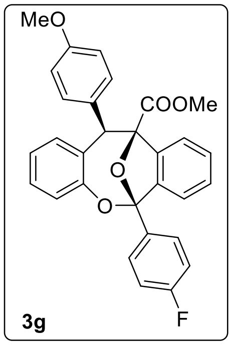

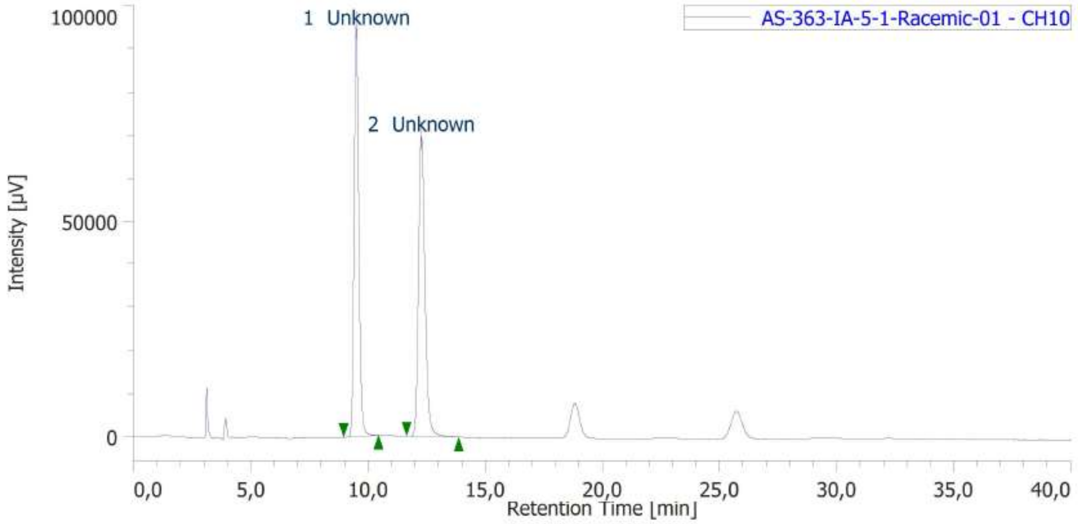

#	Peak Name	CH	tR [min]	Area [µV·sec]	Height [µV]	Area%	Height%	Quantity	NTP	Resolution	Symmetry Factor	Warning
1	Unknown	10	9,500	1333366	95119	49,898	57,716	N/A	11097	6,523	1,163	
2	Unknown	10	12,267	1338817	69685	50,102	42,284	N/A	10035	N/A	1,231	

HPLC data of compound (±)-**3g**

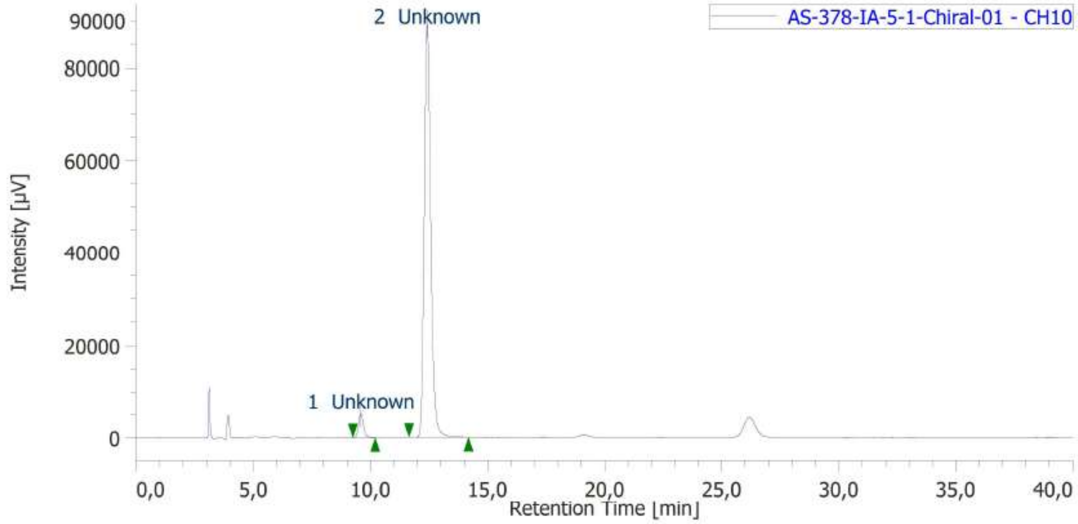

#	Peak Name	CH	tR [min]	Area [µV·sec]	Height [µV]	Area%	Height%	Quantity	NTP	Resolution	Symmetry Factor	Warning
1	Unknown	10	9,593	74859	5206	4,170	5,526	N/A	10809	6,555	1,144	
2	Unknown	10	12,410	1720492	89010	95,830	94,474	N/A	10154	N/A	1,238	

HPLC data of compound (-)-**3g**

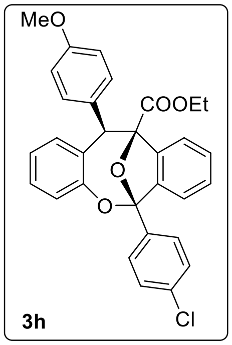

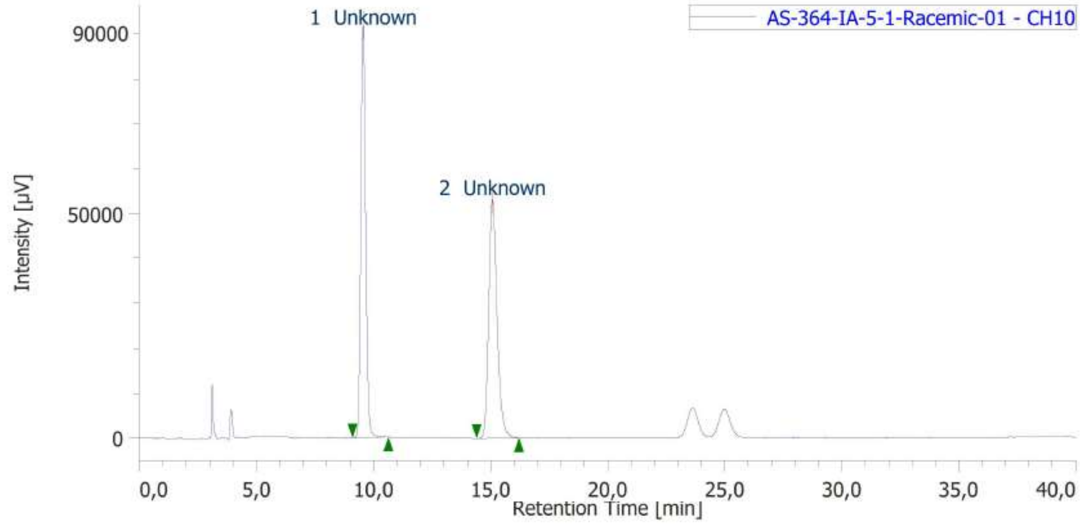

#	Peak Name	CH	tR [min]	Area [μV·sec]	Height [μV]	Area%	Height%	Quantity	NTP	Resolution	Symmetry Factor	Warning
1	Unknown	10	9,553	1316096	91551	50,086	63,274	N/A	10574	11,019	1,118	
2	Unknown	10	15,063	1311568	53138	49,914	36,726	N/A	9115	N/A	1,255	

HPLC data of compound (±)-**3h**

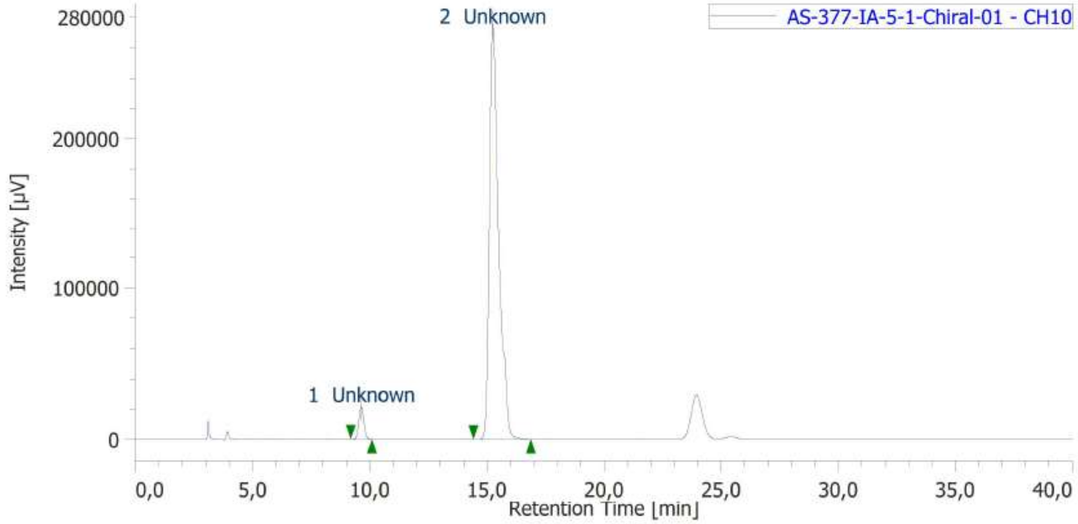

#	Peak Name	CH	tR [min]	Area [μV·sec]	Height [μV]	Area%	Height%	Quantity	NTP	Resolution	Symmetry Factor	Warning
1	Unknown	10	9,637	312366	21734	3,956	7,321	N/A	10627	10,749	1,094	
2	Unknown	10	15,243	7582869	275133	96,044	92,679	N/A	8231	N/A	1,541	

HPLC data of compound (-)-**3h**

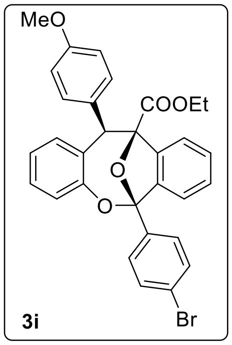

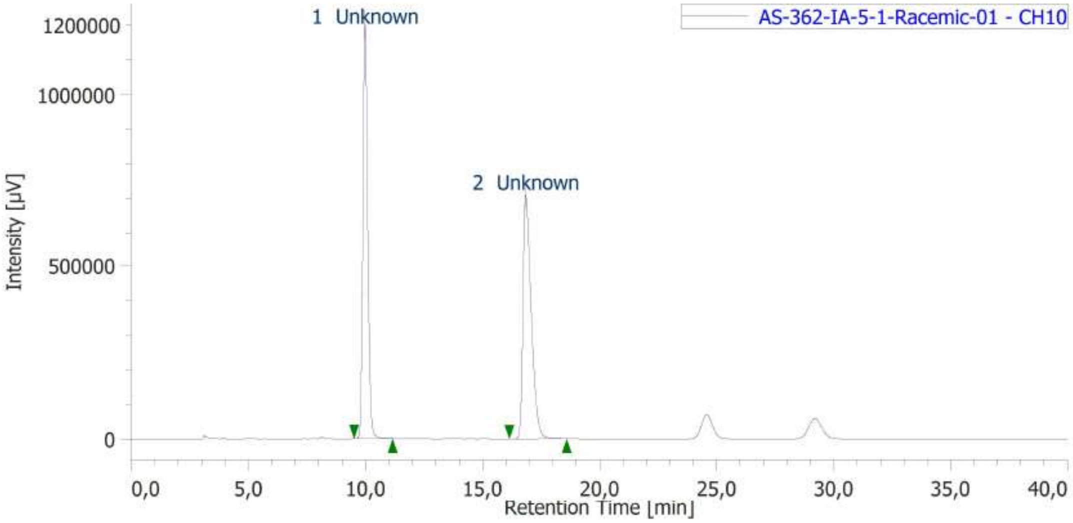

#	Peak Name	CH	tR [min]	Area [µV·sec]	Height [µV]	Area%	Height%	Quantity	NTP	Resolution	Symmetry Factor	Warning
1	Unknown	10	9,980	17191860	1200877	49,599	62,812	N/A	11706	13,704	1,191	
2	Unknown	10	16,833	17470003	710978	50,401	37,188	N/A	11283	N/A	1,666	

HPLC data of compound (±)-**3i**

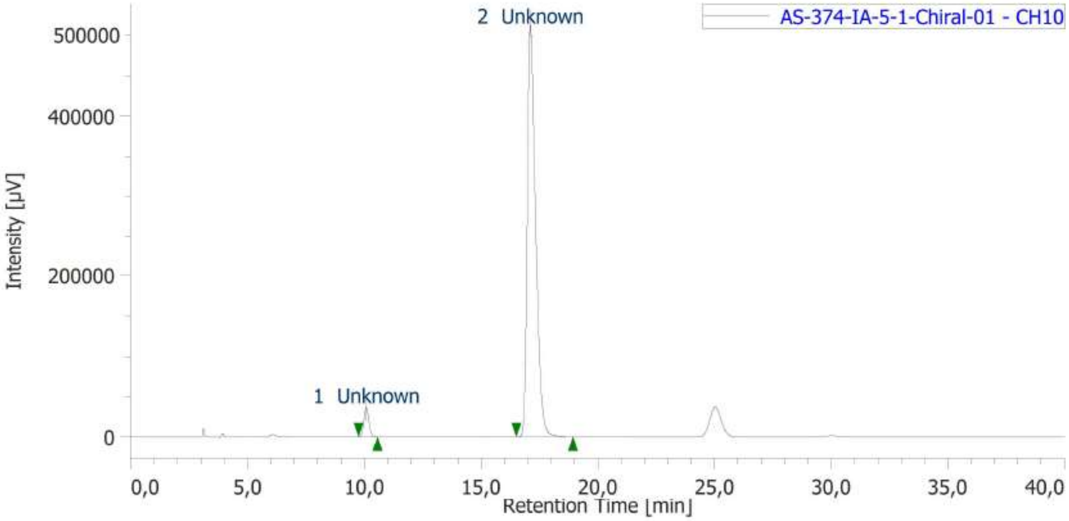

#	Peak Name	CH	tR [min]	Area [µV·sec]	Height [µV]	Area%	Height%	Quantity	NTP	Resolution	Symmetry Factor	Warning
1	Unknown	10	10,080	538056	36656	4,044	6,672	N/A	11021	13,701	1,110	
2	Unknown	10	17,100	12767032	512763	95,956	93,328	N/A	11302	N/A	1,506	

HPLC data of compound (-)-**3i**

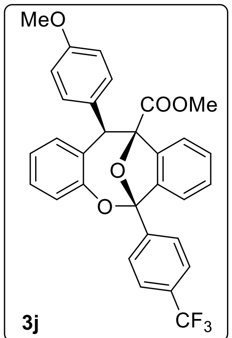

3j

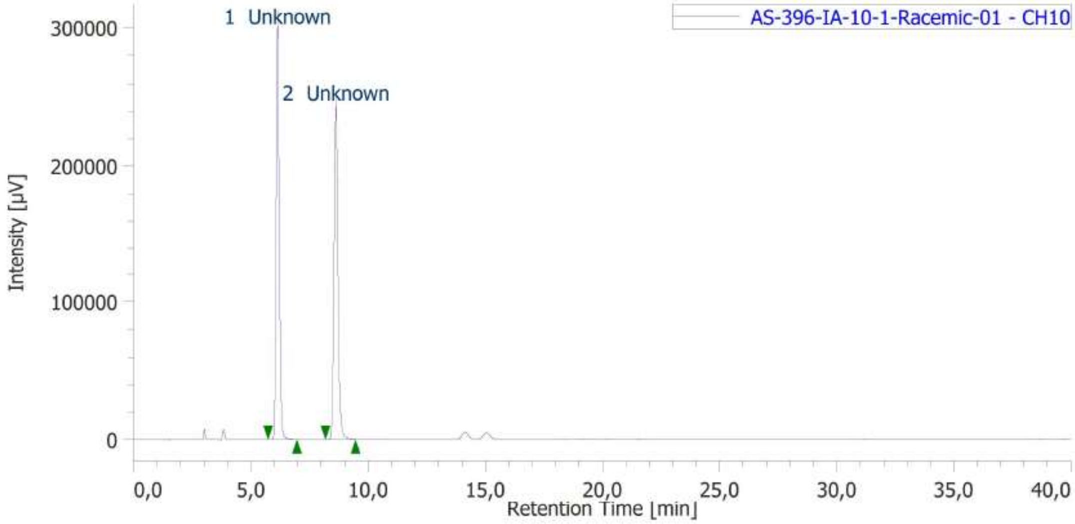

#	Peak Name	CH	tR [min]	Area [µV·sec]	Height [µV]	Area%	Height%	Quantity	NTP	Resolution	Symmetry Factor	Warning
1	Unknown	10	6,133	2833101	301894	49,943	55,300	N/A	10328	9,352	1,162	
2	Unknown	10	8,627	2839577	244022	50,057	44,700	N/A	13847	N/A	1,149	

HPLC data of compound (±)-**3j**

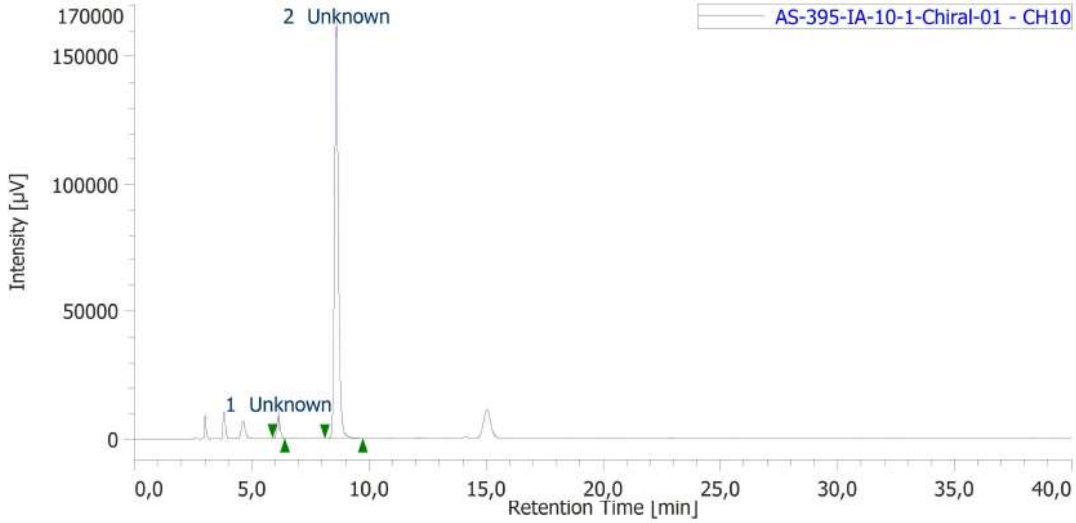

#	Peak Name	CH	tR [min]	Area [µV·sec]	Height [µV]	Area%	Height%	Quantity	NTP	Resolution	Symmetry Factor	Warning
1	Unknown	10	6,127	85280	8599	4,440	5,039	N/A	8845	9,272	1,107	
2	Unknown	10	8,610	1835556	162051	95,560	94,961	N/A	15514	N/A	1,178	

HPLC data of compound (-)-**3j**

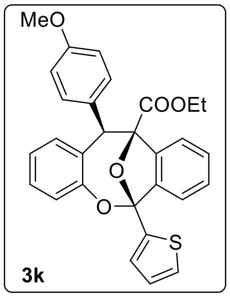

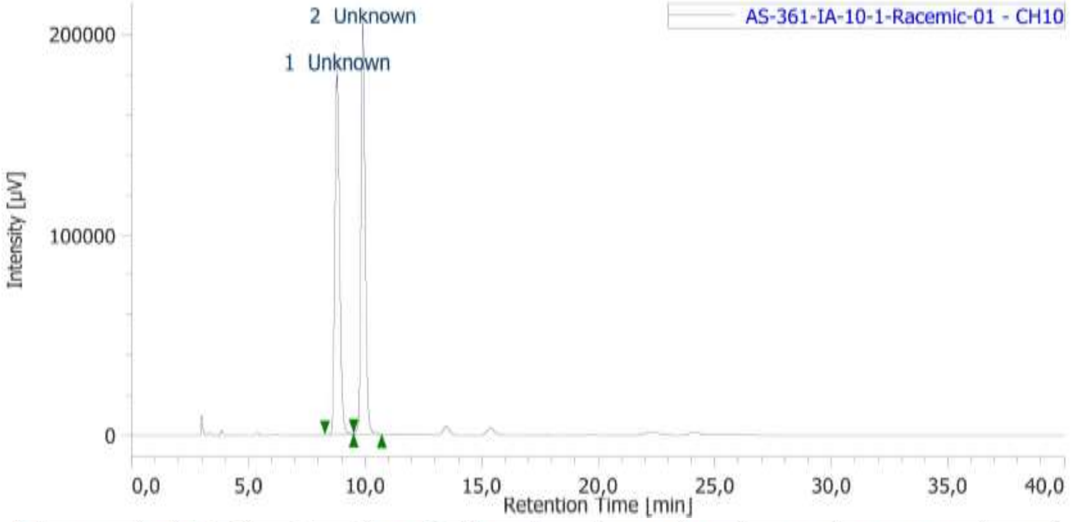

#	Peak Name	CH	tR [min]	Area [µV·sec]	Height [µV]	Area%	Height%	Quantity	NTP	Resolution	Symmetry Factor	Warning
1	Unknown	10	8,797	2574432	180086	49,871	46,795	N/A	8854	3,192	1,214	
2	Unknown	10	9,907	2587723	204758	50,129	53,205	N/A	15020	N/A	1,218	

HPLC data of compound (±)-**3k**

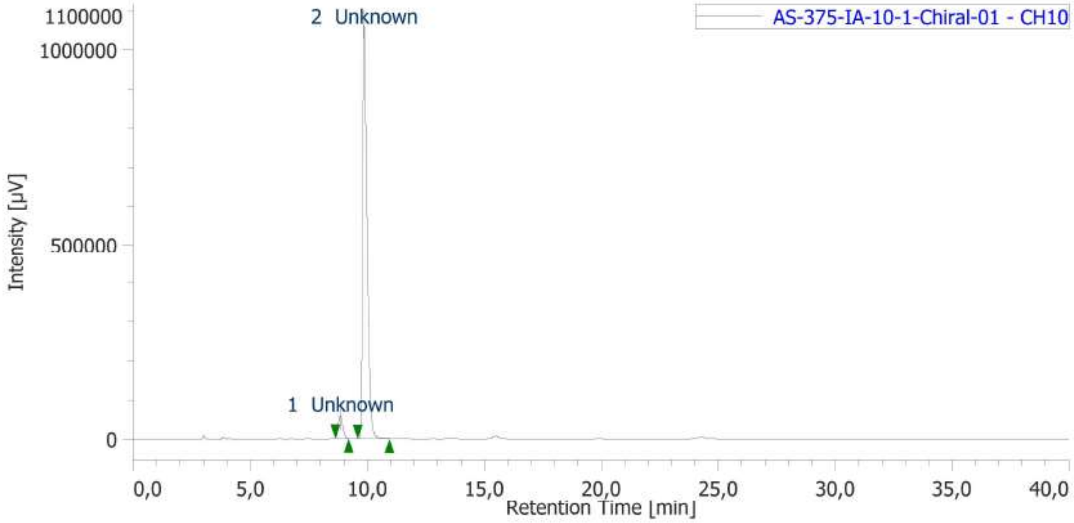

#	Peak Name	CH	tR [min]	Area [µV·sec]	Height [µV]	Area%	Height%	Quantity	NTP	Resolution	Symmetry Factor	Warning
1	Unknown	10	8,843	780665	57538	5,191	5,131	N/A	9394	2,890	1,236	
2	Unknown	10	9,863	14258367	1063930	94,809	94,869	N/A	13251	N/A	1,671	

HPLC data of compound (-)-**3k**

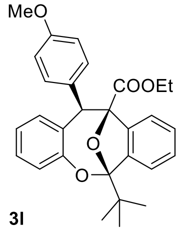

3l

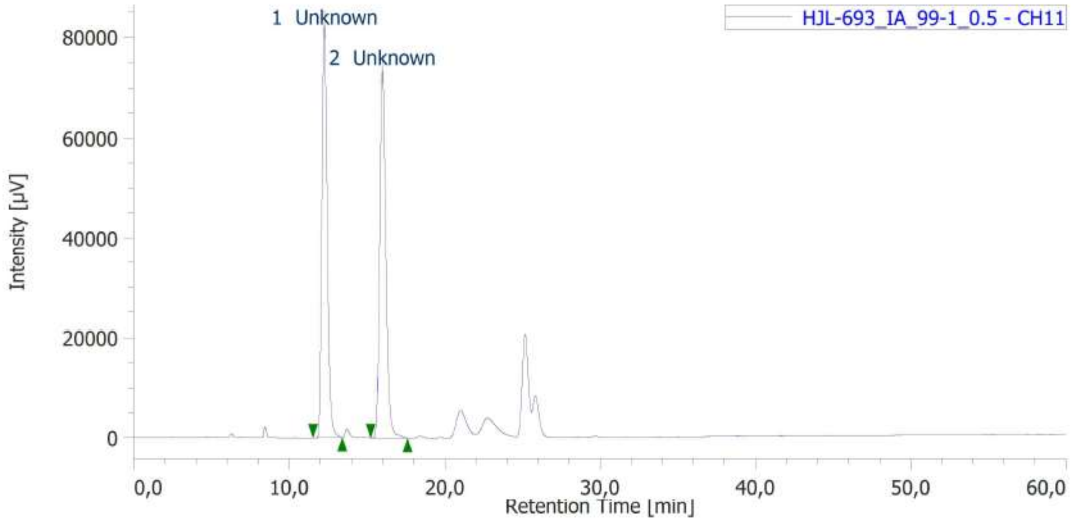

#	Peak Name	CH	tR [min]	Area [µV·sec]	Height [µV]	Area%	Height%	Quantity	NTP	Resolution	Symmetry Factor	Warning
1	Unknown	11	12.247	1999717	82316	49.809	52.826	N/A	5954	5.580	1.229	
2	Unknown	11	15.973	2015077	73510	50.191	47.174	N/A	8227	N/A	1.238	

HPLC data of compound (±)-**3l**

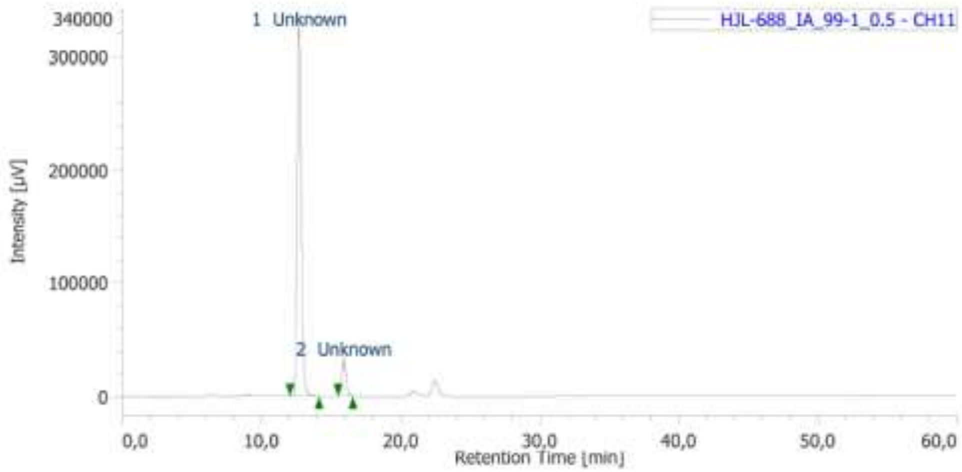

#	Peak Name	CH	tR [min]	Area [µV·sec]	Height [µV]	Area%	Height%	Quantity	NTP	Resolution	Symmetry Factor	Warning
1	Unknown	11	12.703	6839482	326068	90.849	90.944	N/A	8594	5.805	1.170	
2	Unknown	11	15.903	688929	32470	9.151	9.056	N/A	13025	N/A	1.146	

HPLC data of compound (-)-**3l**

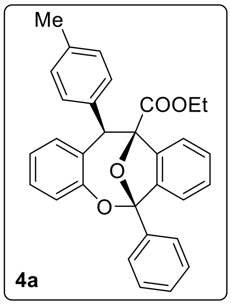

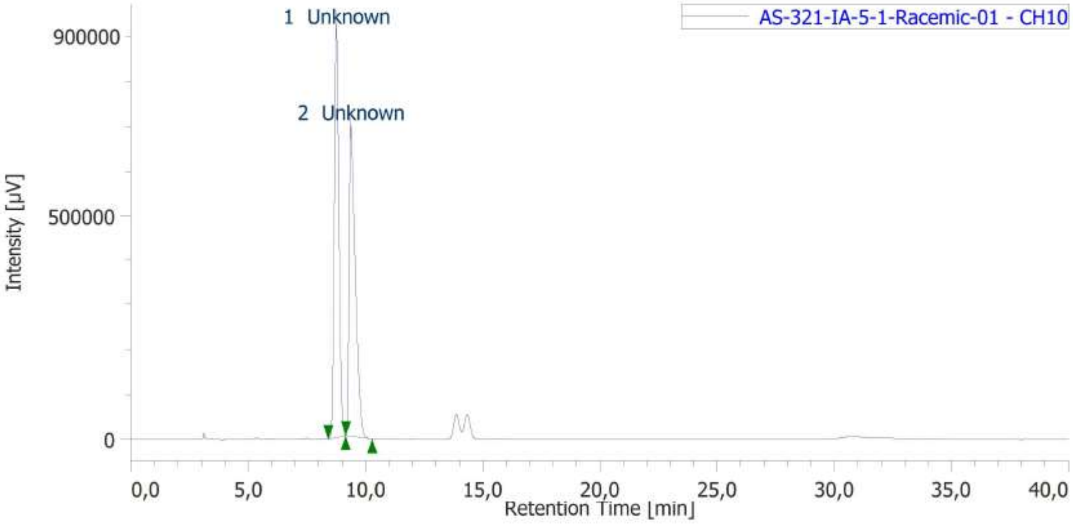

#	Peak Name	CH	tR [min]	Area [μV·sec]	Height [μV]	Area%	Height%	Quantity	NTP	Resolution	Symmetry Factor	Warning
1	Unknown	10	8,753	12723829	921348	49,789	56,950	N/A	9210	1,453	1,155	
2	Unknown	10	9,370	12831525	696471	50,211	43,050	N/A	5937	N/A	2,089	

HPLC data of compound (±)-**4a**

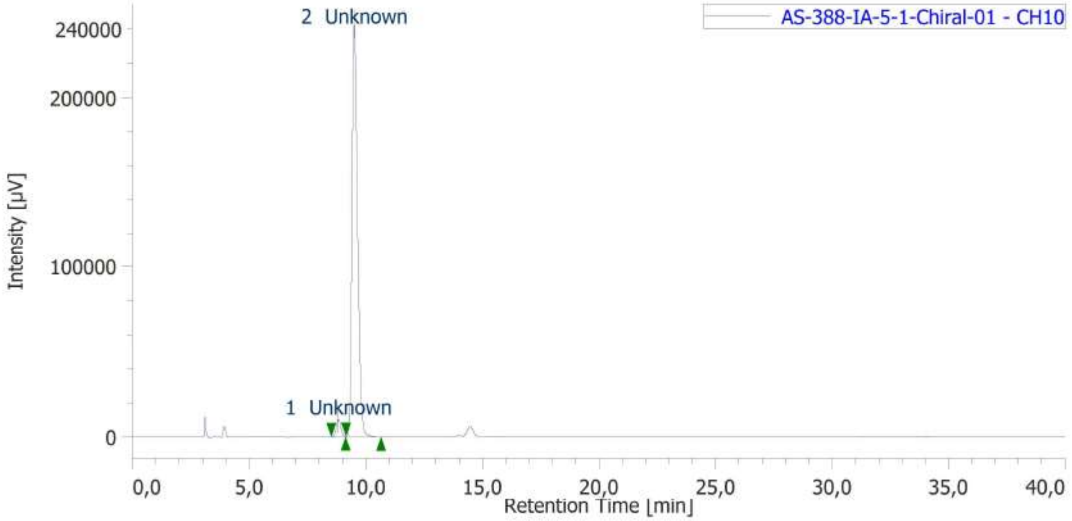

#	Peak Name	CH	tR [min]	Area [μV·sec]	Height [μV]	Area%	Height%	Quantity	NTP	Resolution	Symmetry Factor	Warning
1	Unknown	10	8,827	138399	10323	3,337	4,085	N/A	9846	1,713	1,058	
2	Unknown	10	9,500	4009388	242354	96,663	95,915	N/A	7730	N/A	1,390	

HPLC data of compound (-)-**4a**

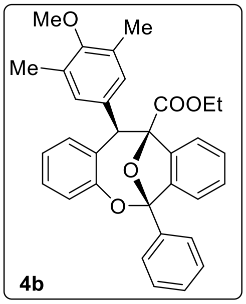

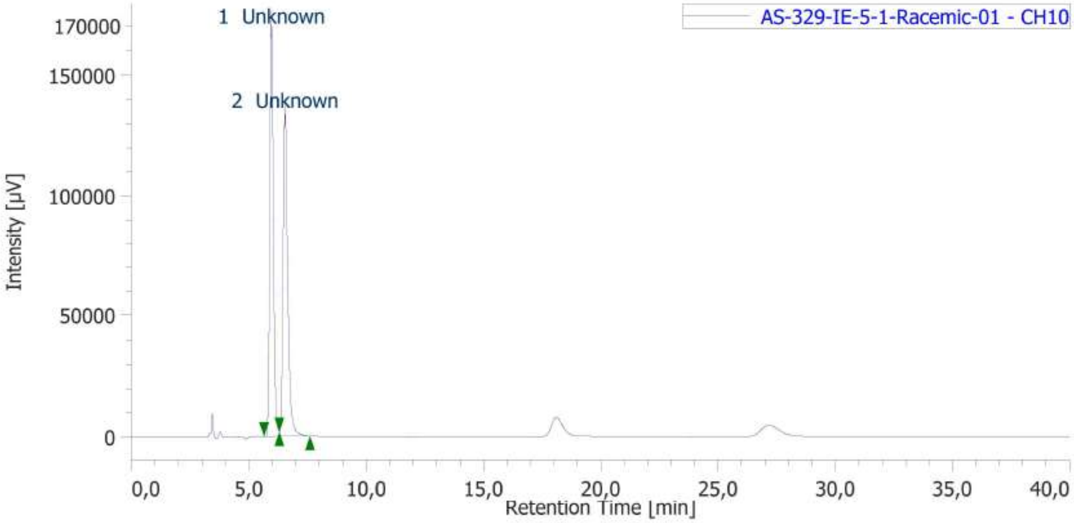

#	Peak Name	CH	tR [min]	Area [µV·sec]	Height [µV]	Area%	Height%	Quantity	NTP	Resolution	Symmetry Factor	Warning
1	Unknown	10	5,953	1840964	170276	49,740	55,941	N/A	7259	1,876	1,215	
2	Unknown	10	6,537	1860173	134107	50,260	44,059	N/A	5775	N/A	1,406	

HPLC data of compound (±)-**4b**

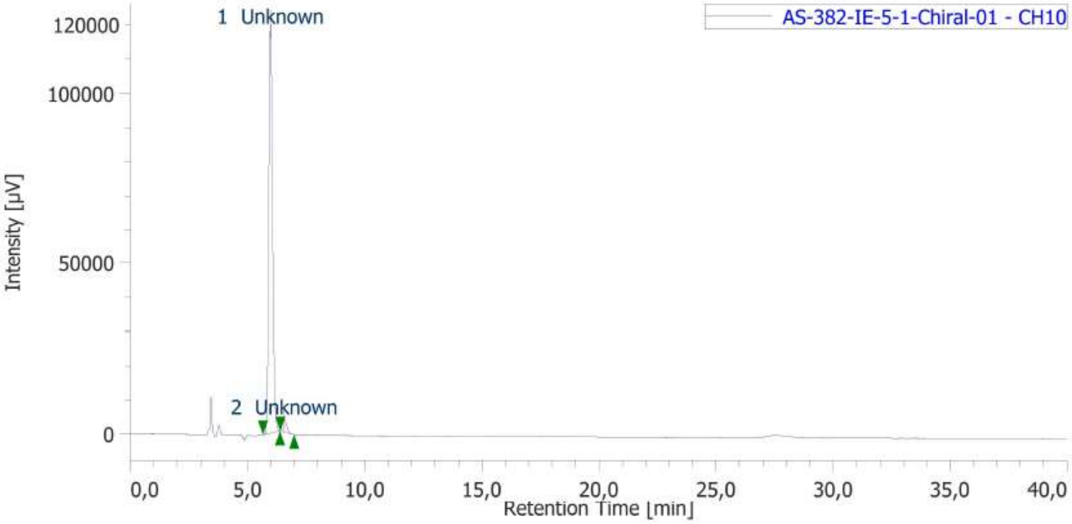

#	Peak Name	CH	tR [min]	Area [µV·sec]	Height [µV]	Area%	Height%	Quantity	NTP	Resolution	Symmetry Factor	Warning
1	Unknown	10	5,973	1341623	119698	96,660	96,913	N/A	6739	1,894	1,198	
2	Unknown	10	6,560	46366	3813	3,340	3,087	N/A	6324	N/A	1,276	

HPLC data of compound (-)-**4b**

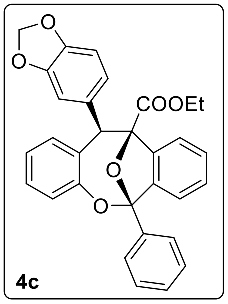

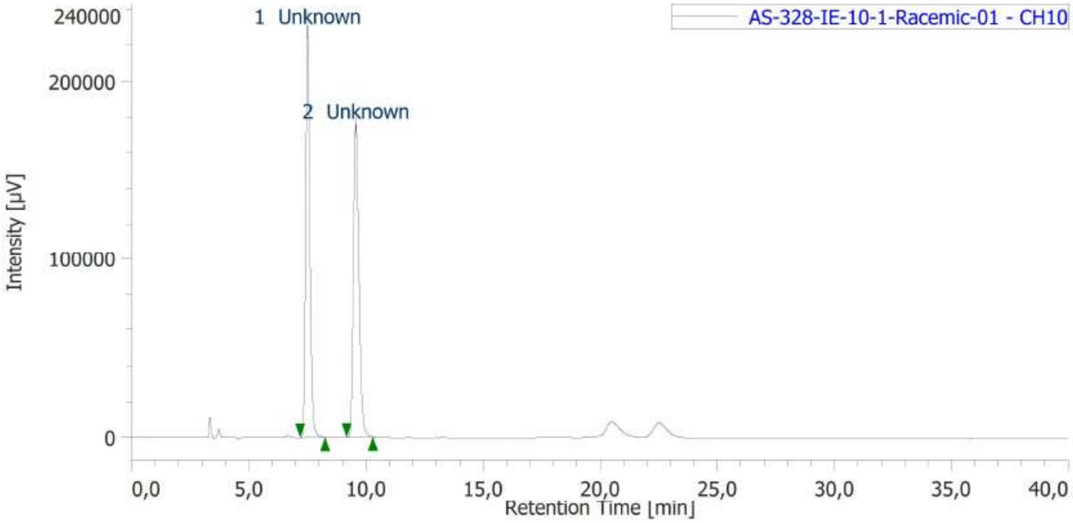

#	Peak Name	CH	tR [min]	Area [µV·sec]	Height [µV]	Area%	Height%	Quantity	NTP	Resolution	Symmetry Factor	Warning
1	Unknown	10	7,503	2885186	231435	50,052	56,739	N/A	8755	5,562	1,247	
2	Unknown	10	9,567	2879188	176457	49,948	43,261	N/A	8178	N/A	1,269	

HPLC data of compound (±)-**4c**

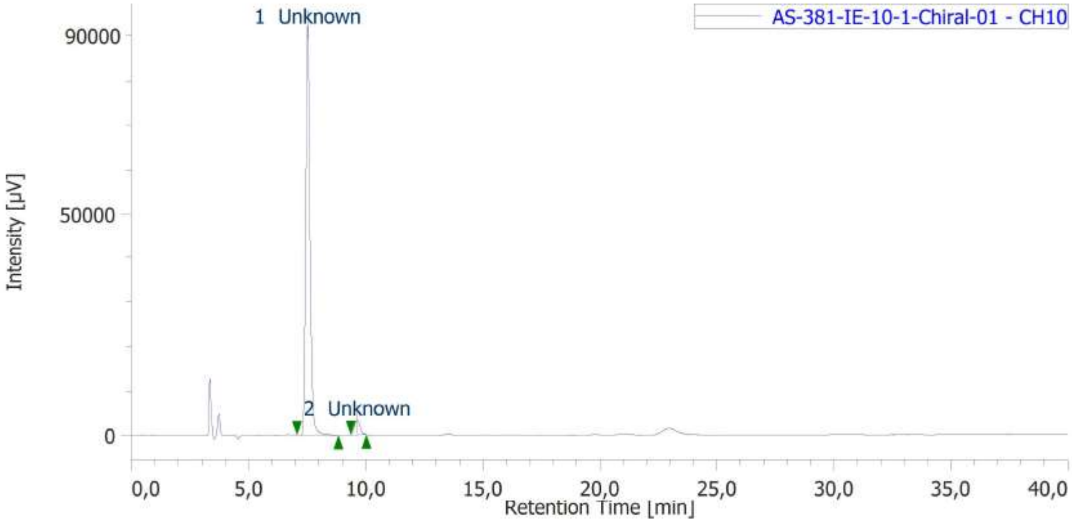

#	Peak Name	CH	tR [min]	Area [µV·sec]	Height [µV]	Area%	Height%	Quantity	NTP	Resolution	Symmetry Factor	Warning
1	Unknown	10	7,513	1179023	92460	95,545	96,539	N/A	8561	5,517	1,253	
2	Unknown	10	9,627	54977	3314	4,455	3,461	N/A	7545	N/A	1,176	

HPLC data of compound (-)-**4c**

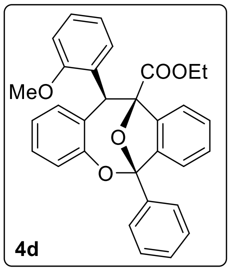

4d

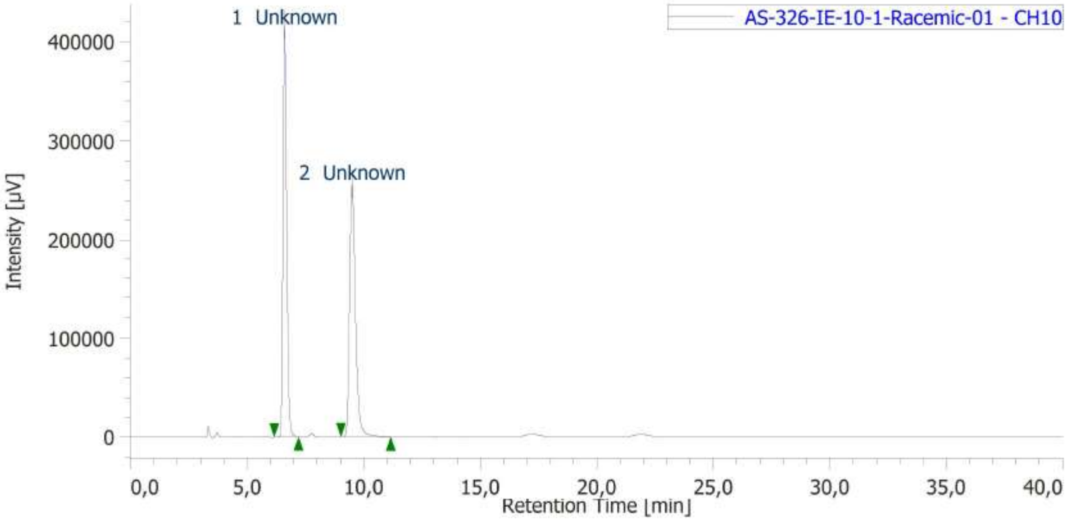

#	Peak Name	CH	tR [min]	Area [µV·sec]	Height [µV]	Area%	Height%	Quantity	NTP	Resolution	Symmetry Factor	Warning
1	Unknown	10	6,600	4524395	416692	50,080	61,939	N/A	8843	8,033	1,286	
2	Unknown	10	9,500	4510017	256059	49,920	38,061	N/A	7352	N/A	1,349	

HPLC data of compound (±)-**4d**

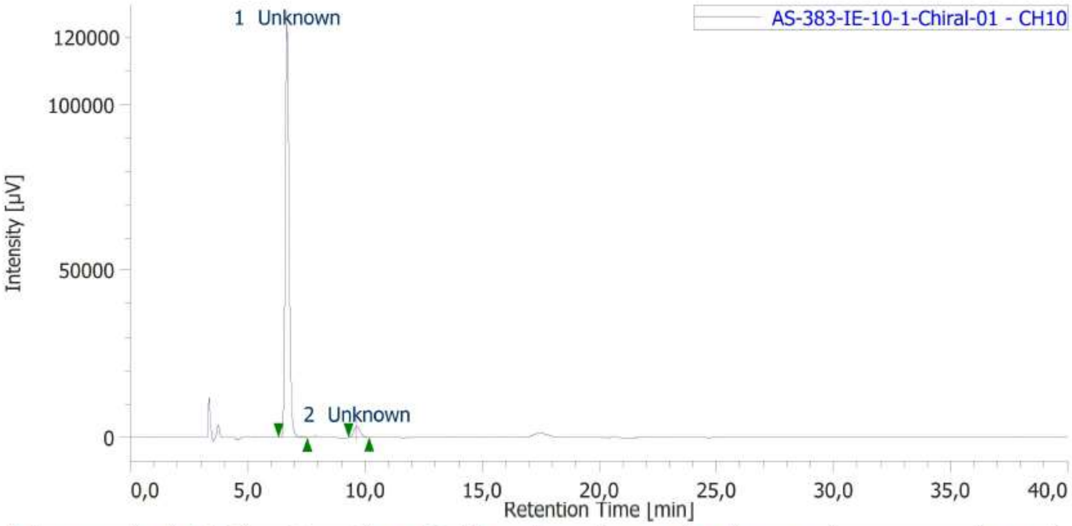

#	Peak Name	CH	tR [min]	Area [µV·sec]	Height [µV]	Area%	Height%	Quantity	NTP	Resolution	Symmetry Factor	Warning
1	Unknown	10	6,667	1414848	123452	95,701	97,378	N/A	8204	7,553	1,227	
2	Unknown	10	9,647	63561	3324	4,299	2,622	N/A	6032	N/A	1,241	

HPLC data of compound (-)-**4d**

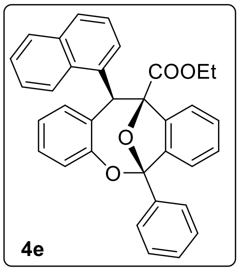

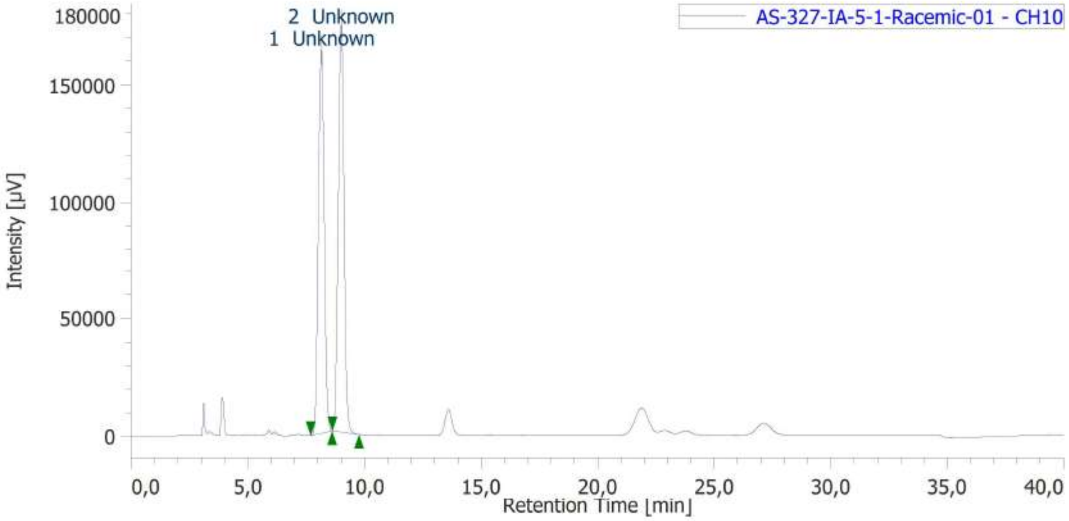

#	Peak Name	CH	tR [min]	Area [µV·sec]	Height [µV]	Area%	Height%	Quantity	NTP	Resolution	Symmetry Factor	Warning
1	Unknown	10	8,150	2917089	163148	49,231	48,436	N/A	4544	1,807	1,014	
2	Unknown	10	9,000	3008199	173685	50,769	51,564	N/A	6133	N/A	1,021	

HPLC data of compound (±)-**4e**

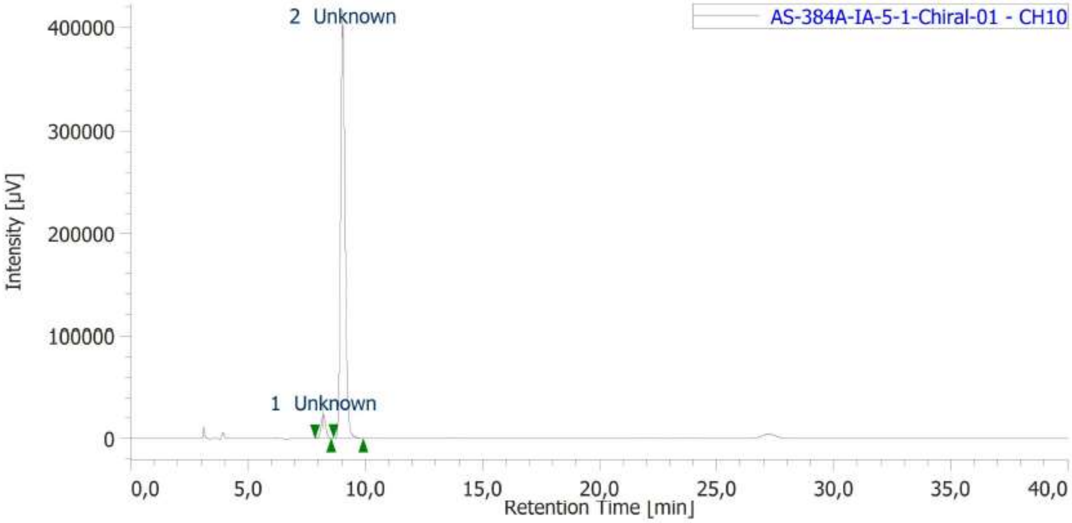

#	Peak Name	CH	tR [min]	Area [µV·sec]	Height [µV]	Area%	Height%	Quantity	NTP	Resolution	Symmetry Factor	Warning
1	Unknown	10	8,210	314924	23008	5,395	5,410	N/A	8655	2,341	1,004	
2	Unknown	10	9,037	5522671	402268	94,605	94,590	N/A	10360	N/A	1,159	

HPLC data of compound (-)-**4e**

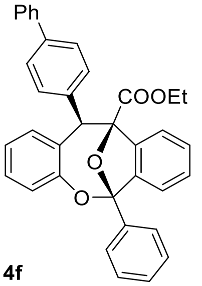

4f

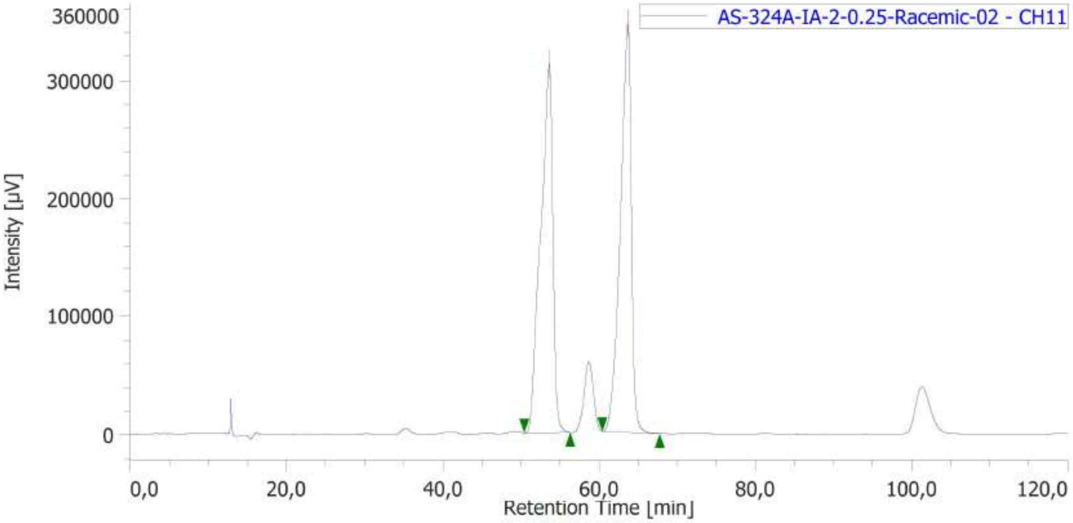

#	Peak Name	CH	tR [min]	Area [µV·sec]	Height [µV]	Area%	Height%	Quantity	NTP	Resolution	Symmetry Factor	Warning
1	Unknown	11	53,593	33753804	311885	50,084	47,501	N/A	5138	3,680	0,744	
2	Unknown	11	63,667	33640165	344700	49,916	52,499	N/A	10383	N/A	0,738	

HPLC data of compound (±)-**4f**

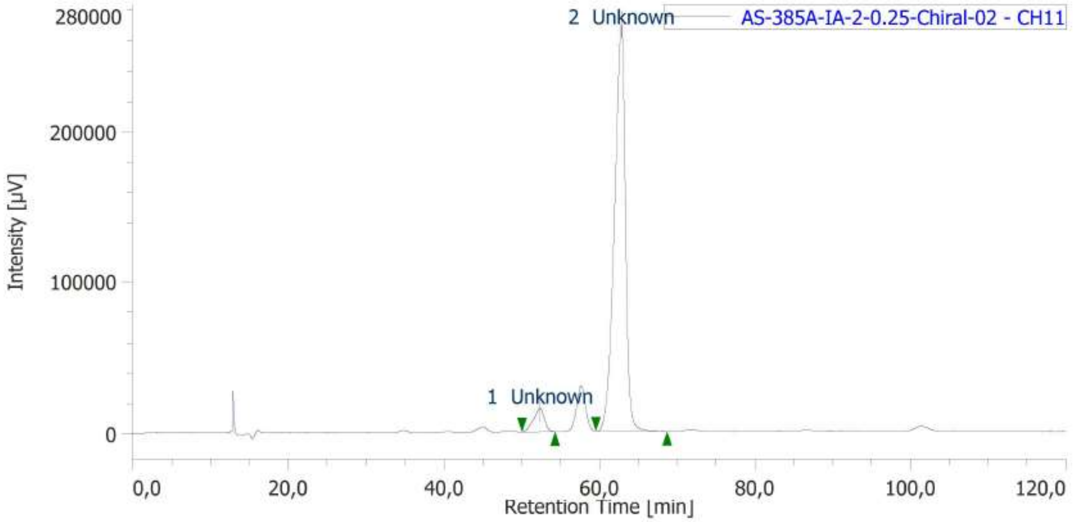

#	Peak Name	CH	tR [min]	Area [µV·sec]	Height [µV]	Area%	Height%	Quantity	NTP	Resolution	Symmetry Factor	Warning
1	Unknown	11	52,333	1508664	15676	5,406	5,521	N/A	6837	4,142	0,862	
2	Unknown	11	62,803	26396325	268280	94,594	94,479	N/A	9801	N/A	0,809	

HPLC data of compound (-)-**4f**

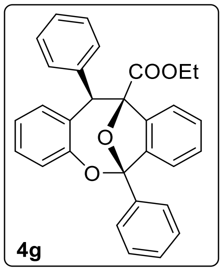

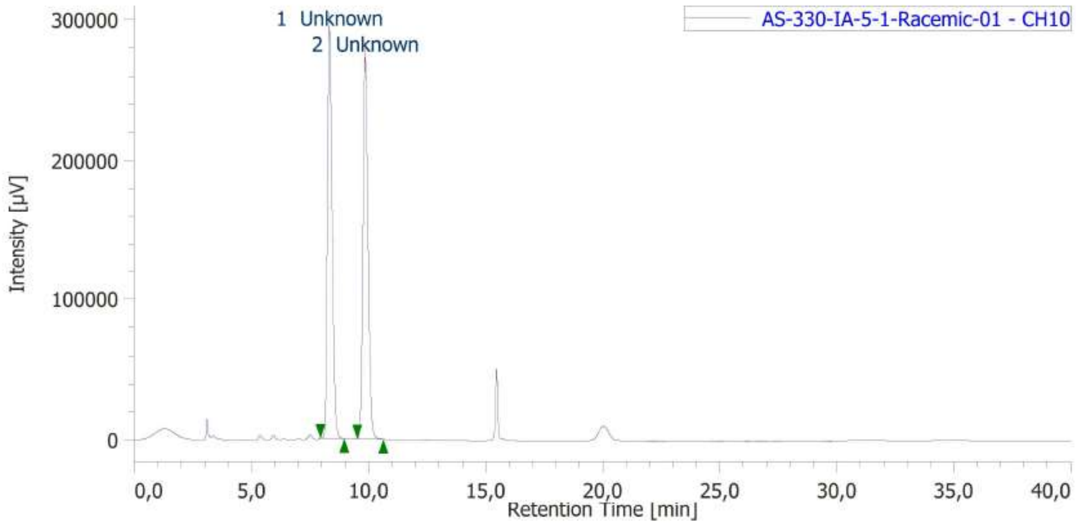

#	Peak Name	CH	tR [min]	Area [µV·sec]	Height [µV]	Area%	Height%	Quantity	NTP	Resolution	Symmetry Factor	Warning
1	Unknown	10	8,323	4133661	293991	49,956	51,851	N/A	8136	4,002	1,285	
2	Unknown	10	9,853	4140956	273001	50,044	48,149	N/A	9830	N/A	1,199	

HPLC data of compound (±)-**4g**

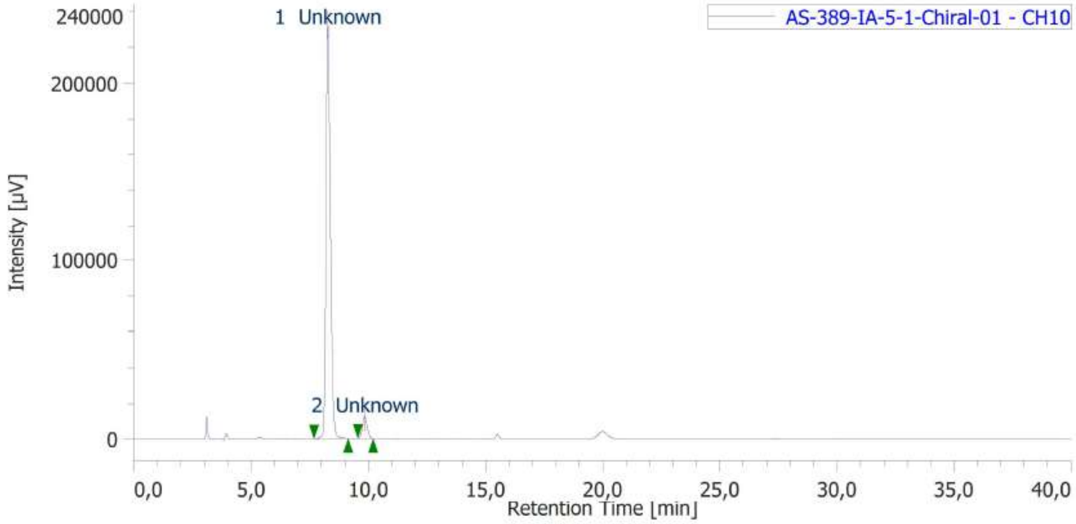

#	Peak Name	CH	tR [min]	Area [µV·sec]	Height [µV]	Area%	Height%	Quantity	NTP	Resolution	Symmetry Factor	Warning
1	Unknown	10	8,270	3045804	232075	94,591	95,012	N/A	9461	4,399	1,277	
2	Unknown	10	9,847	174157	12183	5,409	4,988	N/A	10815	N/A	1,075	

HPLC data of compound (-)-**4g**

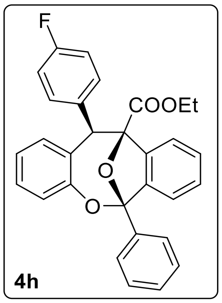

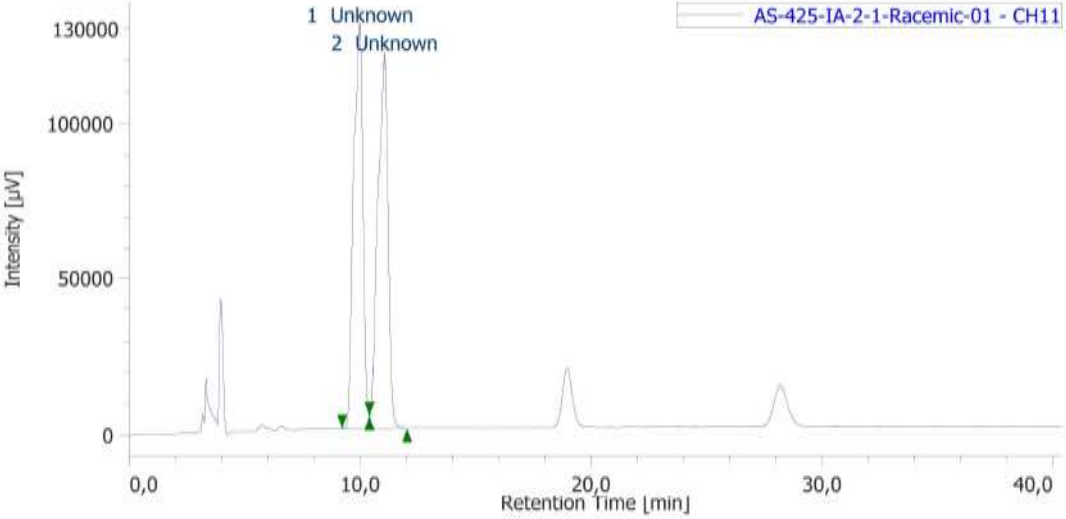

#	Peak Name	CH	tR [min]	Area [µV·sec]	Height [µV]	Area%	Height%	Quantity	NTP	Resolution	Symmetry Factor	Warning
1	Unknown	11	9,970	3673804	129772	49,756	52,065	N/A	2346	1,236	0,858	
2	Unknown	11	11,033	3709764	119478	50,244	47,935	N/A	2392	N/A	0,826	

HPLC data of compound (±)-**4h**

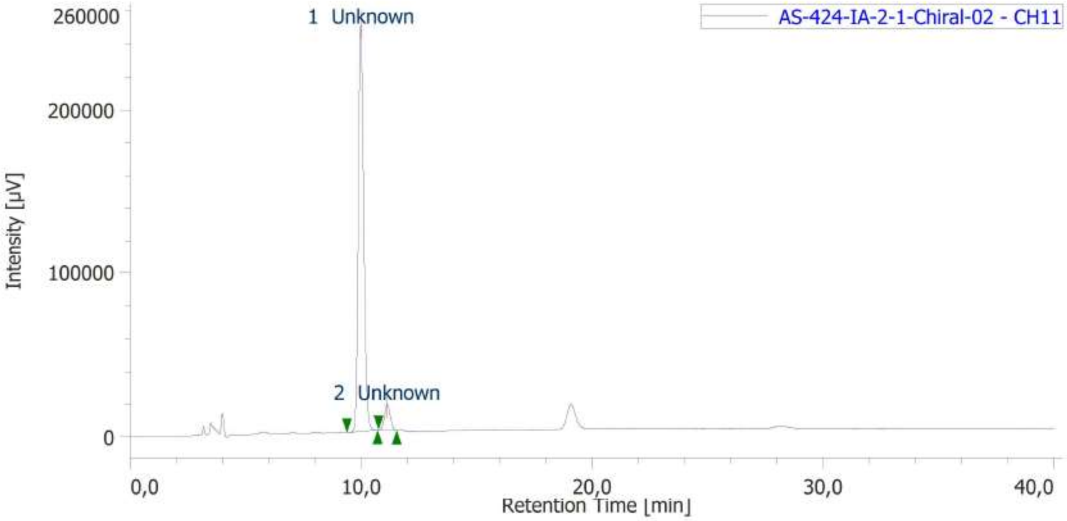

#	Peak Name	CH	tR [min]	Area [µV·sec]	Height [µV]	Area%	Height%	Quantity	NTP	Resolution	Symmetry Factor	Warning
1	Unknown	11	9,970	4024460	248164	93,695	94,043	N/A	8790	2,559	1,197	
2	Unknown	11	11,103	270795	15720	6,305	5,957	N/A	9217	N/A	1,066	

HPLC data of compound (-)-**4h**

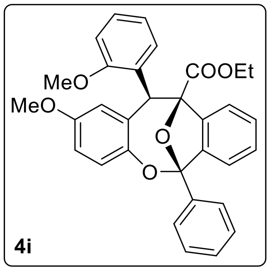

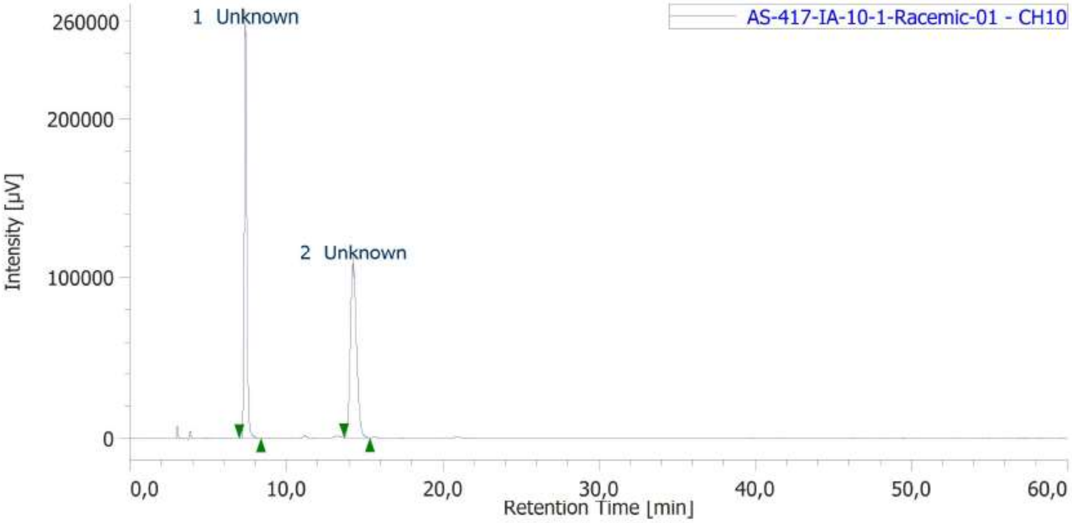

#	Peak Name	CH	tR [min]	Area [µV·sec]	Height [µV]	Area%	Height%	Quantity	NTP	Resolution	Symmetry Factor	Warning
1	Unknown	10	7,367	3022668	258227	50,130	70,522	N/A	9772	13,444	1,233	
2	Unknown	10	14,263	3007026	107939	49,870	29,478	N/A	6098	N/A	1,209	

HPLC data of compound (±)-**4i**

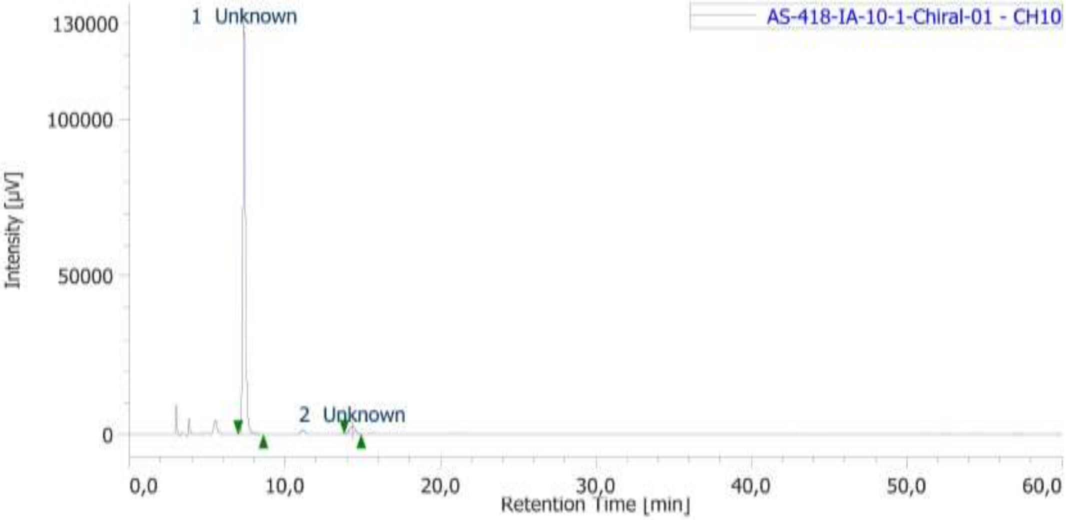

#	Peak Name	CH	tR [min]	Area [µV·sec]	Height [µV]	Area%	Height%	Quantity	NTP	Resolution	Symmetry Factor	Warning
1	Unknown	10	7,363	1576812	129757	95,810	98,082	N/A	9062	13,471	1,217	
2	Unknown	10	14,323	68961	2538	4,190	1,918	N/A	6216	N/A	1,098	

HPLC data of compound (-)-**4i**

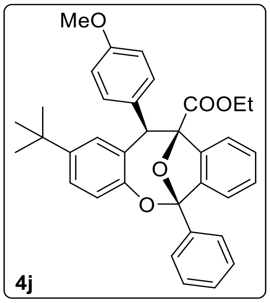

4j

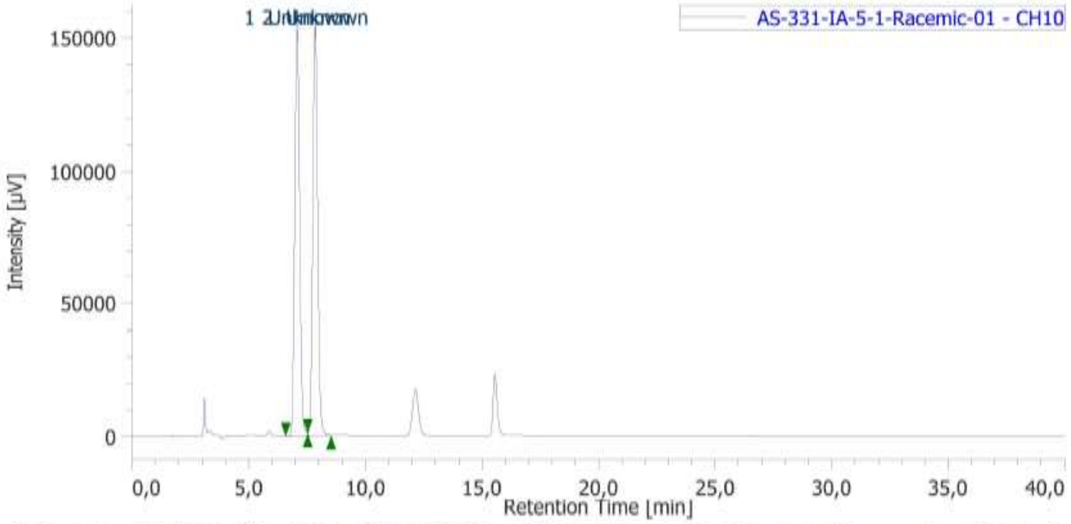

#	Peak Name	CH	tR [min]	Area [μV·sec]	Height [μV]	Area%	Height%	Quantity	NTP	Resolution	Symmetry Factor	Warning
1	Unknown	10	7,070	2246034	153659	49,937	49,900	N/A	5361	2,023	1,110	
2	Unknown	10	7,847	2251680	154274	50,063	50,100	N/A	6691	N/A	1,084	

HPLC data of compound (±)-**4j**

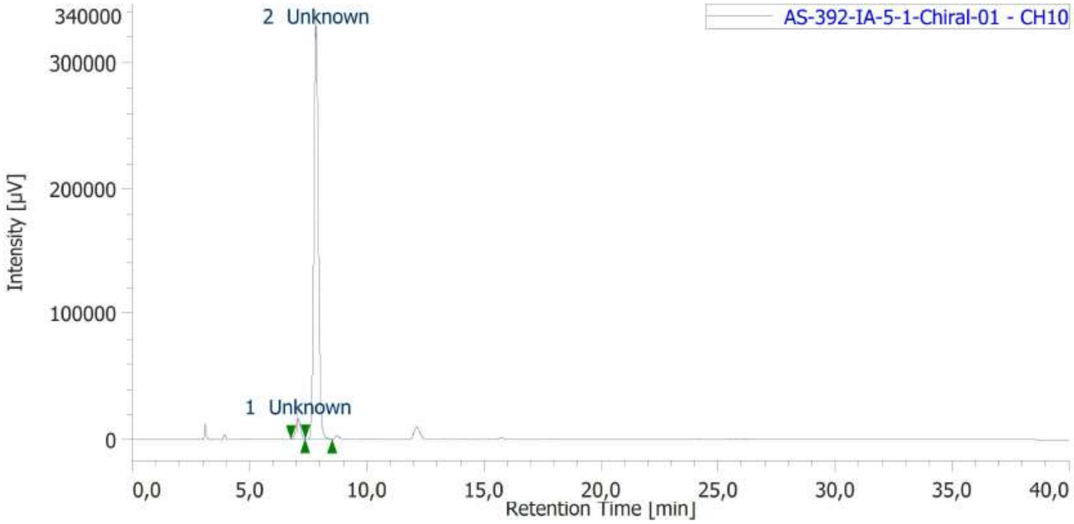

#	Peak Name	CH	tR [min]	Area [μV·sec]	Height [μV]	Area%	Height%	Quantity	NTP	Resolution	Symmetry Factor	Warning
1	Unknown	10	7,067	220354	16146	4,787	4,676	N/A	6028	2,153	1,031	
2	Unknown	10	7,830	4382559	329125	95,213	95,324	N/A	8148	N/A	1,117	

HPLC data of compound (-)-**4j**

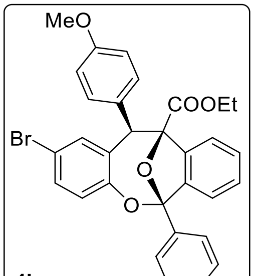

4k

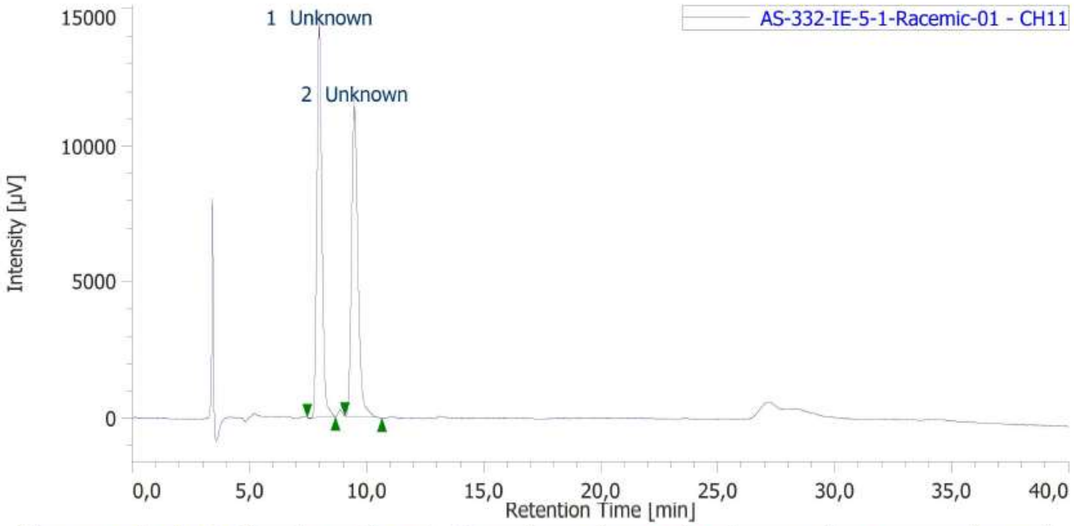

#	Peak Name	CH	tR [min]	Area [μV·sec]	Height [μV]	Area%	Height%	Quantity	NTP	Resolution	Symmetry Factor	Warning
1	Unknown	11	7,973	217438	14346	49,858	55,702	N/A	6712	3,458	1,200	
2	Unknown	11	9,480	218678	11408	50,142	44,298	N/A	6124	N/A	1,277	

HPLC data of compound (±)-**4k**

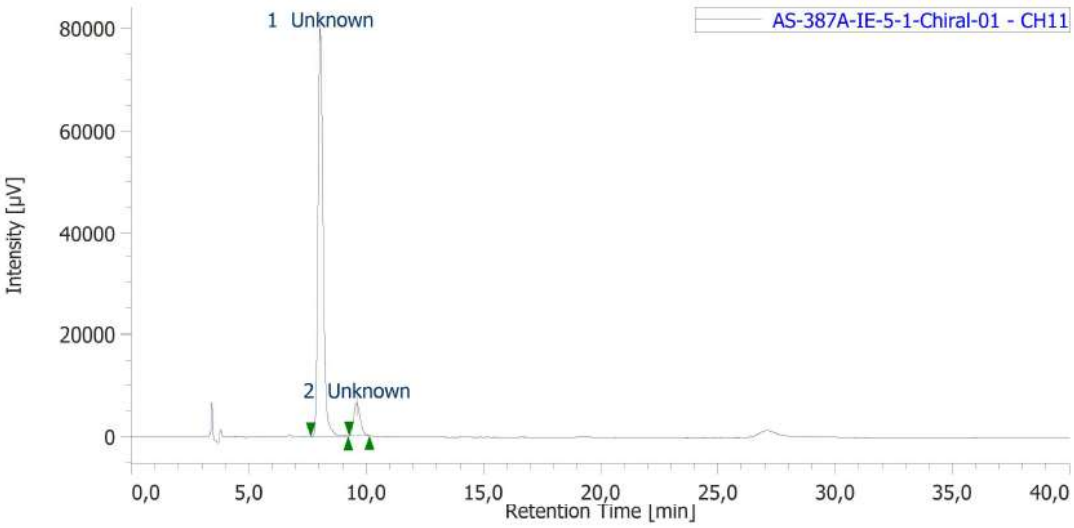

#	Peak Name	CH	tR [min]	Area [μV·sec]	Height [μV]	Area%	Height%	Quantity	NTP	Resolution	Symmetry Factor	Warning
1	Unknown	11	8,043	1218548	80046	90,746	92,406	N/A	6956	3,548	1,253	
2	Unknown	11	9,597	124263	6578	9,254	7,594	N/A	6080	N/A	1,250	

HPLC data of compound (-)-**4k**

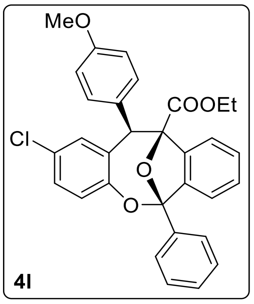

4l

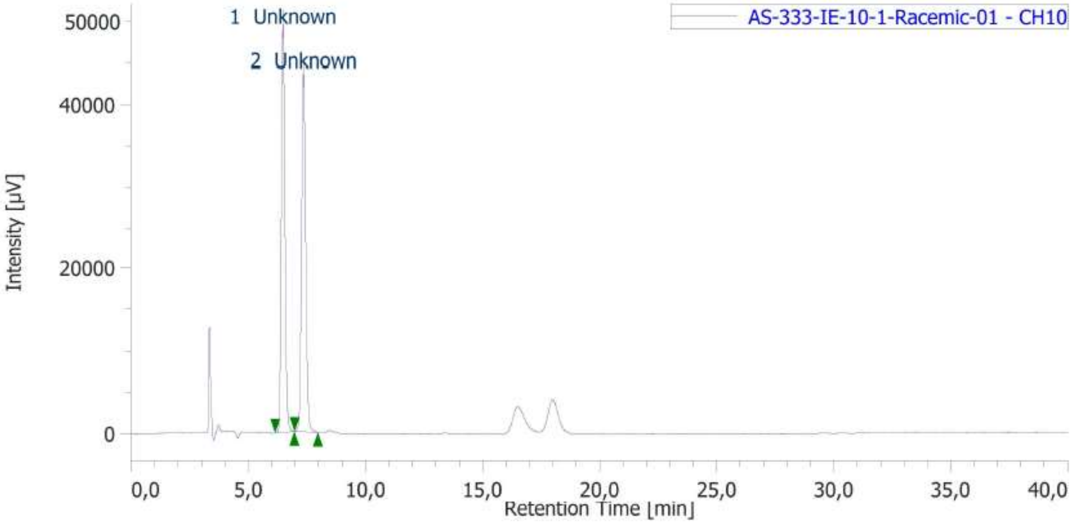

#	Peak Name	CH	tR [min]	Area [µV·sec]	Height [µV]	Area%	Height%	Quantity	NTP	Resolution	Symmetry Factor	Warning
1	Unknown	10	6,477	527253	49450	49,611	53,144	N/A	8704	2,949	1,149	
2	Unknown	10	7,350	535520	43599	50,389	46,856	N/A	8645	N/A	1,130	

HPLC data of compound (±)-**4l**

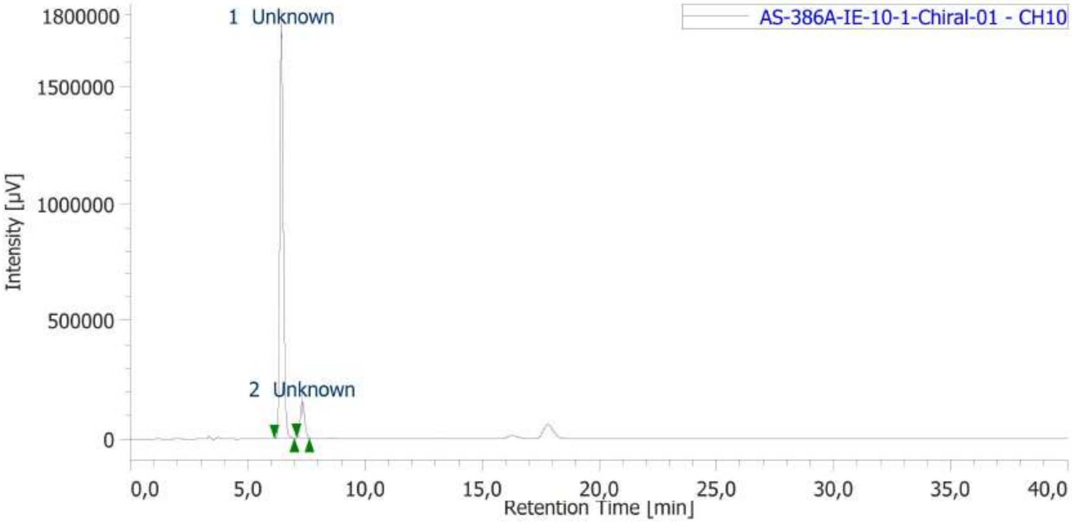

#	Peak Name	CH	tR [min]	Area [µV·sec]	Height [µV]	Area%	Height%	Quantity	NTP	Resolution	Symmetry Factor	Warning
1	Unknown	10	6,427	18482566	1756163	90,531	91,838	N/A	9059	2,997	1,233	
2	Unknown	10	7,320	1933274	156080	9,469	8,162	N/A	7989	N/A	1,140	

HPLC data of compound (-)-**4l**

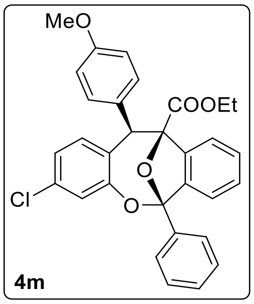

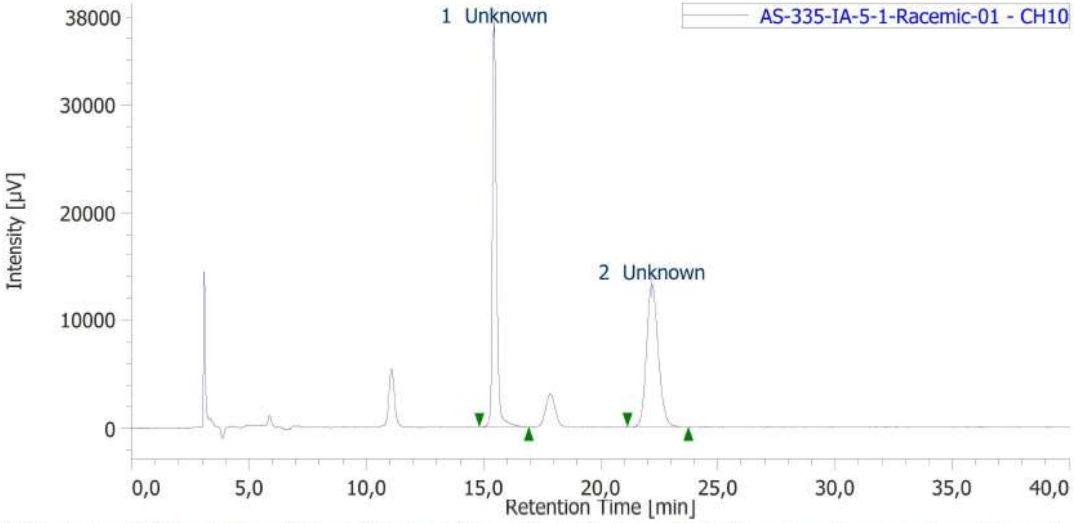

#	Peak Name	CH	tR [min]	Area [µV·sec]	Height [µV]	Area%	Height%	Quantity	NTP	Resolution	Symmetry Factor	Warning
1	Unknown	10	15,440	459440	37254	50,091	73,973	N/A	41959	11,130	1,386	
2	Unknown	10	22,180	457772	13108	49,909	26,027	N/A	9445	N/A	1,158	

HPLC data of compound (±)-**4m**

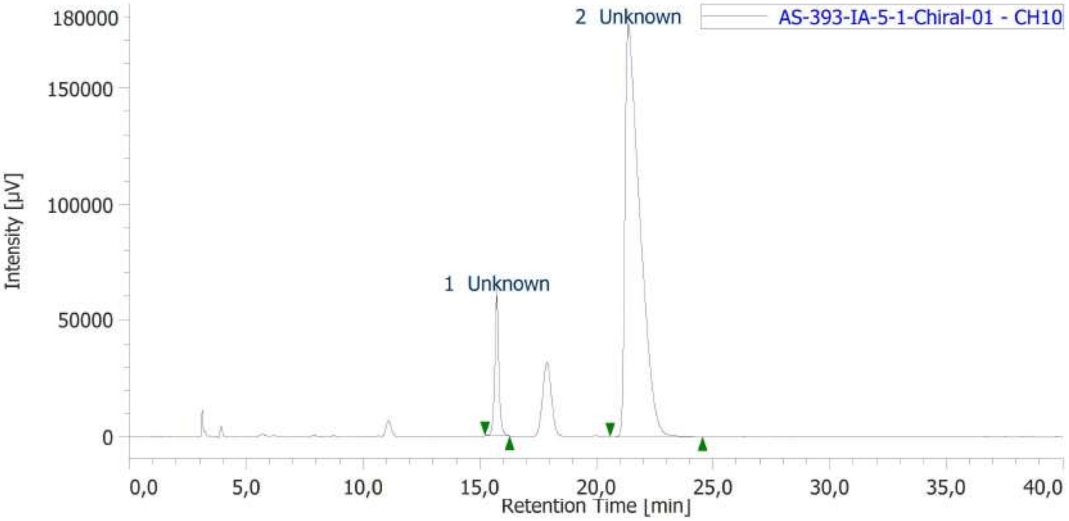

#	Peak Name	CH	tR [min]	Area [µV·sec]	Height [µV]	Area%	Height%	Quantity	NTP	Resolution	Symmetry Factor	Warning
1	Unknown	10	15,720	731852	59937	8,487	25,305	N/A	47829	7,749	1,077	
2	Unknown	10	21,363	7891149	176921	91,513	74,695	N/A	5308	N/A	2,618	

HPLC data of compound (-)-**4m**

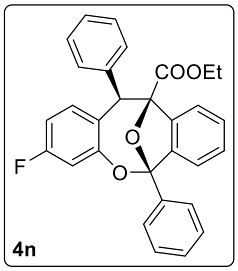

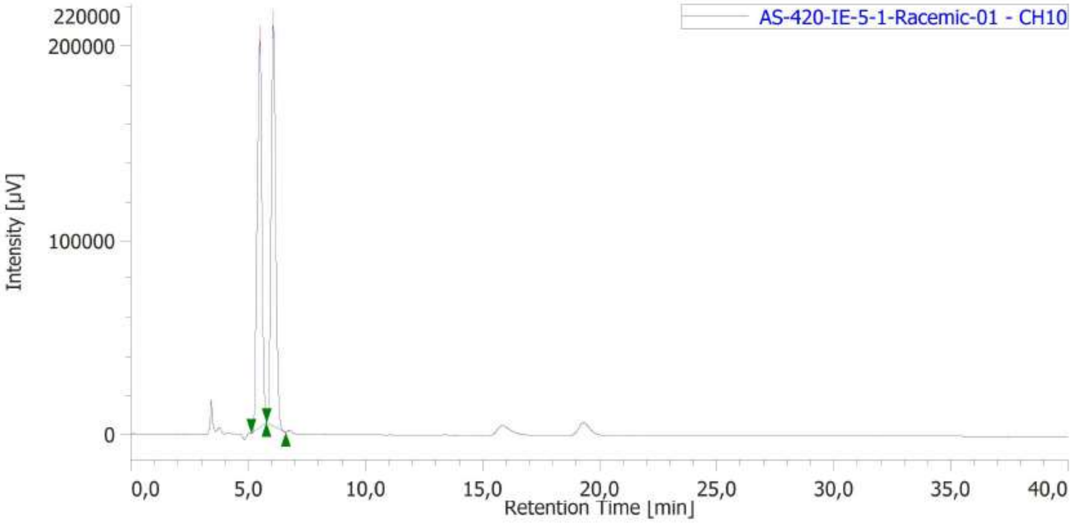

#	Peak Name	CH	tR [min]	Area [µV·sec]	Height [µV]	Area%	Height%	Quantity	NTP	Resolution	Symmetry Factor	Warning
1	Unknown	10	5,487	2847822	199432	49,994	49,101	N/A	3140	1,525	0,952	
2	Unknown	10	6,060	2848517	206733	50,006	50,899	N/A	4472	N/A	1,126	

HPLC data of compound (±)-**4n**

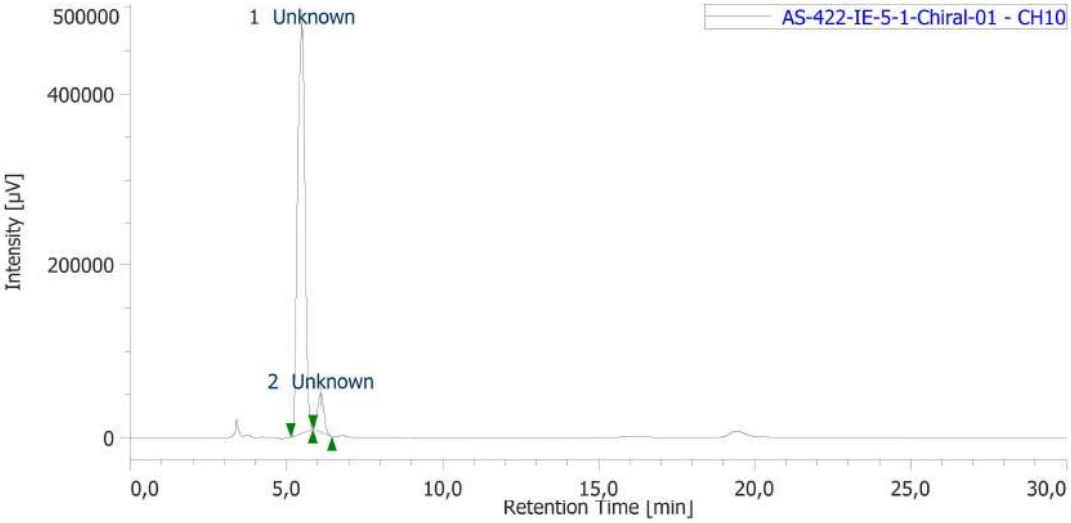

#	Peak Name	CH	tR [min]	Area [µV·sec]	Height [µV]	Area%	Height%	Quantity	NTP	Resolution	Symmetry Factor	Warning
1	Unknown	10	5,493	7871624	474406	92,593	91,273	N/A	2211	1,431	0,957	
2	Unknown	10	6,093	629684	45359	7,407	8,727	N/A	4264	N/A	1,058	

HPLC data of compound (-)-**4n**

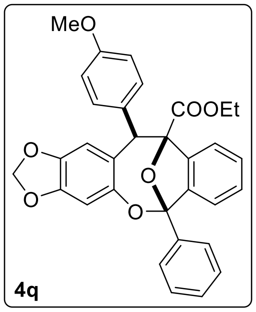

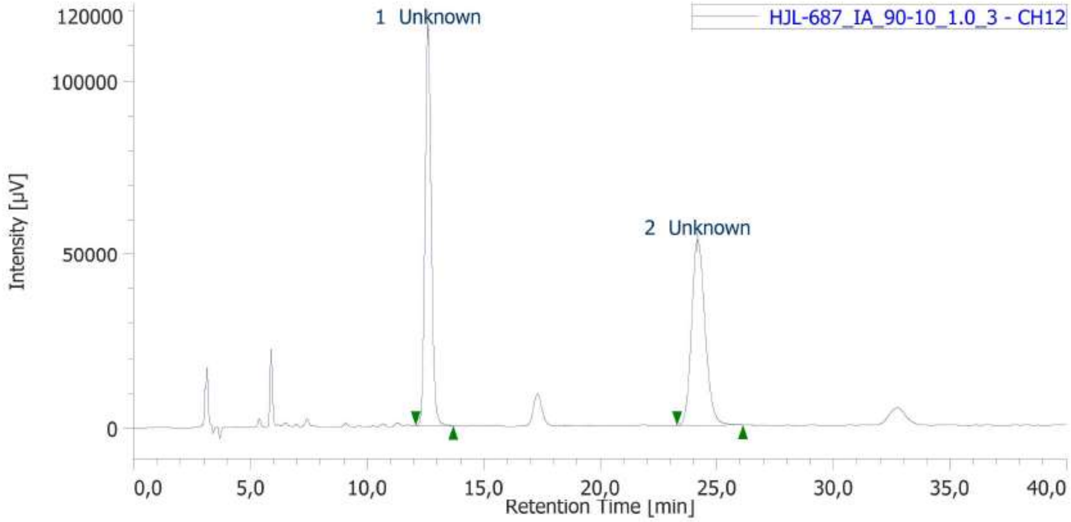

#	Peak Name	CH	tR [min]	Area [µV·sec]	Height [µV]	Area%	Height%	Quantity	NTP	Resolution	Symmetry Factor	Warning
1	Unknown	12	12,600	2160723	115147	50,085	68,212	N/A	10823	15,233	1,132	
2	Unknown	12	24,170	2153363	53660	49,915	31,788	N/A	8663	N/A	1,189	

HPLC data of compound (±)-**4q** using $Rh_2(OAc)_4$

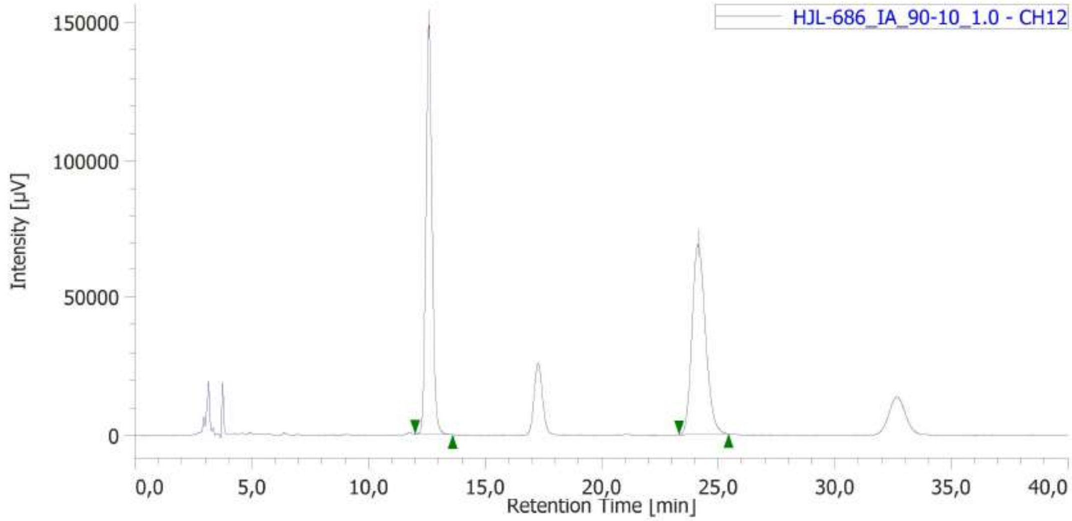

#	Peak Name	CH	tR [min]	Area [µV·sec]	Height [µV]	Area%	Height%	Quantity	NTP	Resolution	Symmetry Factor	Warning
1	Unknown	12	12,580	2758583	148653	49,805	68,156	N/A	11012	15,163	1,111	
2	Unknown	12	24,123	2780228	69454	50,195	31,844	N/A	8492	N/A	1,212	

HPLC data of compound (±)-**4q** using $Rh_2(S\text{-DOSP})_4$

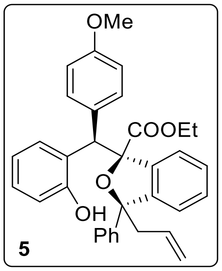

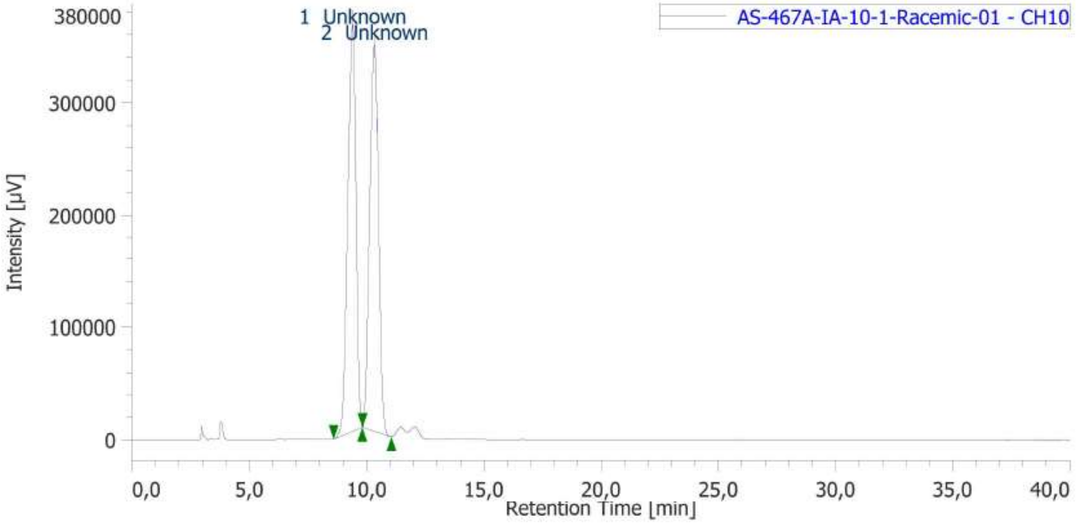

#	Peak Name	CH	tR [min]	Area [µV·sec]	Height [µV]	Area%	Height%	Quantity	NTP	Resolution	Symmetry Factor	Warning
1	Unknown	10	9,403	8877122	361101	50,061	51,268	N/A	3146	1,350	0,859	
2	Unknown	10	10,330	8855655	343242	49,939	48,732	N/A	3429	N/A	0,983	

HPLC data of compound (±)-**5**

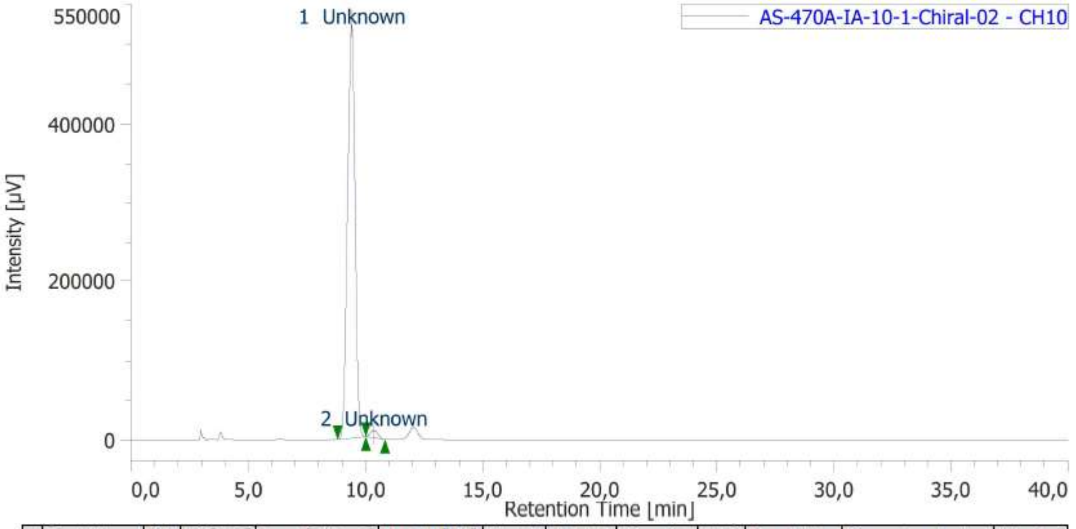

#	Peak Name	CH	tR [min]	Area [µV·sec]	Height [µV]	Area%	Height%	Quantity	NTP	Resolution	Symmetry Factor	Warning
1	Unknown	10	9,410	11836009	522520	98,330	98,252	N/A	3768	1,573	0,939	
2	Unknown	10	10,360	201008	9295	1,670	1,748	N/A	4802	N/A	1,044	

HPLC data of compound (+)-**5**

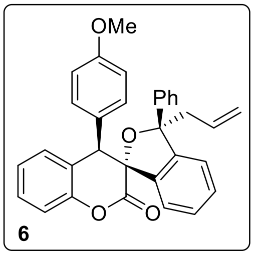

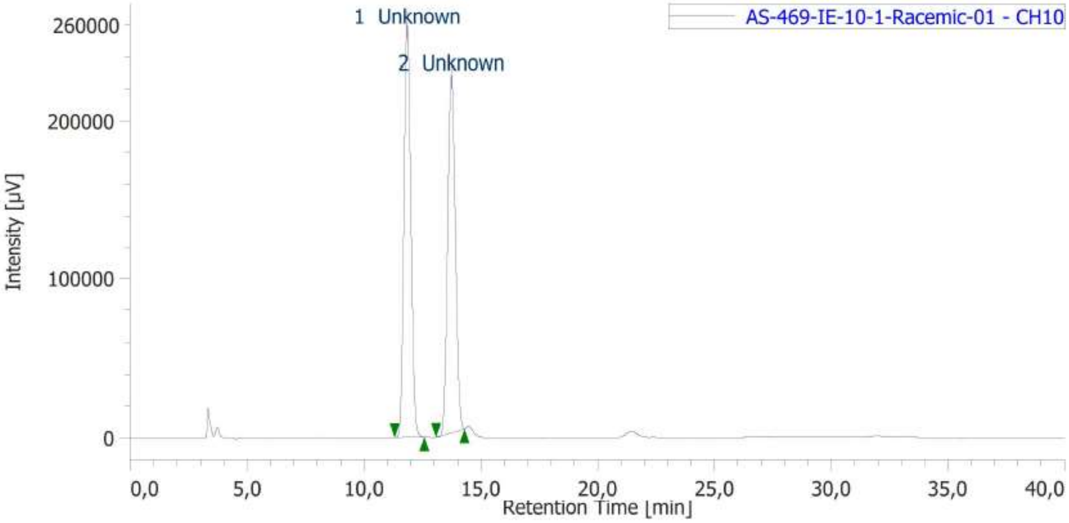

#	Peak Name	CH	tR [min]	Area [µV·sec]	Height [µV]	Area%	Height%	Quantity	NTP	Resolution	Symmetry Factor	Warning
1	Unknown	10	11,837	5344704	259957	50,826	53,521	N/A	7501	3,257	1,110	
2	Unknown	10	13,723	5171015	225751	49,174	46,479	N/A	7972	N/A	1,084	

HPLC data of compound (±)-**6**

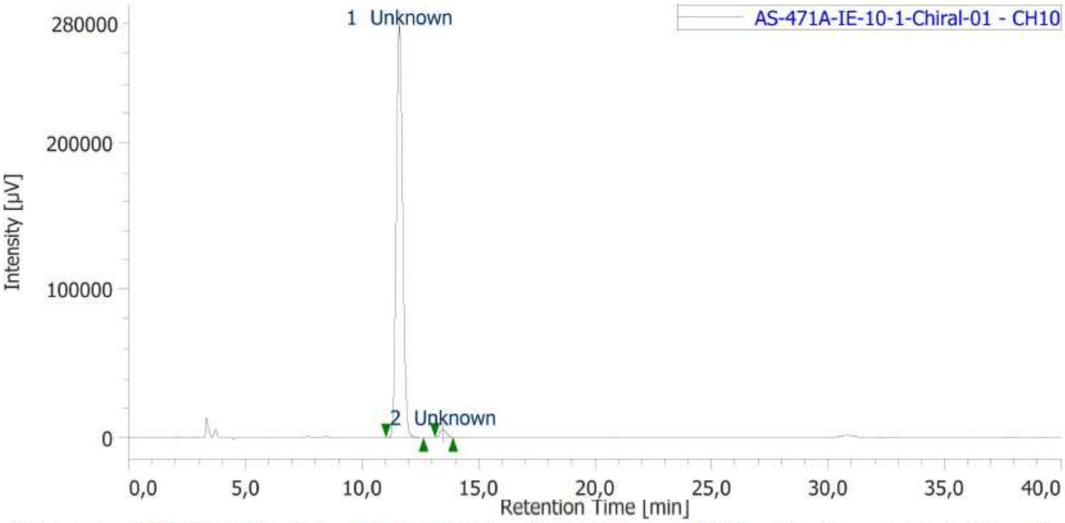

#	Peak Name	CH	tR [min]	Area [µV·sec]	Height [µV]	Area%	Height%	Quantity	NTP	Resolution	Symmetry Factor	Warning
1	Unknown	10	11,593	5341455	278573	98,058	98,176	N/A	8371	3,543	1,103	
2	Unknown	10	13,467	105812	5174	1,942	1,824	N/A	9470	N/A	1,067	

HPLC data of compound (-)-**6**

Table 1. Crystal data and structure refinement for compound **3k**.

Identification code	**3k**	
Empirical formula	C29 H24 O5 S	
Formula weight	484.54	
Temperature	130(2) K	
Wavelength	71.073 pm	
Crystal system	Orthorhombic	
Space group	P 21 21 21	
Unit cell dimensions	a = 834.60(2) pm	α= 90°.
	b = 1087.83(3) pm	β= 90°.
	c = 2640.99(7) pm	γ = 90°.
Volume	2.39776(11) nm^3	
Z	4	
Density (calculated)	1.342 Mg/m^3	
Absorption coefficient	0.174 mm^{-1}	
F(000)	1016	
Crystal size	0.30 x 0.10 x 0.05 mm^3	
Theta range for data collection	2.025 to 27.849°.	
Index ranges	-10<=h<=10, -14<=k<=14, -33<=l<=32	
Reflections collected	21916	
Independent reflections	5251 [R(int) = 0.0412]	
Completeness to theta = 26.375°	99.8 %	
Absorption correction	Semi-empirical from equivalents	
Max. and min. transmission	1.00000 and 0.99326	
Refinement method	Full-matrix least-squares on F^2	
Data / restraints / parameters	5251 / 0 / 412	
Goodness-of-fit on F^2	1.050	
Final R indices [I>2sigma(I)]	R1 = 0.0438, wR2 = 0.0753	
R indices (all data)	R1 = 0.0581, wR2 = 0.0802	
Absolute structure parameter	-0.03(4)	
Largest diff. peak and hole	0.219 and -0.235 e.Å^{-3}	

Comments: Structure solution with SHELXT-2018 (dual-space method). Anisotropic refinement of all non-hydrogen atoms with SHELXL-2018. All H atoms were located on difference Fourier maps calculated at the final stage of the structure refinement.

Table 2. Atomic coordinates (x 10^4) and equivalent isotropic displacement parameters (pm^2x 10^{-1}) for **3k**. U(eq) is defined as one third of the trace of the orthogonalized U^{ij} tensor.

	x	y	z	U(eq)
S(1)	1438(1)	2841(1)	2693(1)	35(1)
O(1)	1576(2)	5776(2)	3582(1)	19(1)
O(2)	3589(2)	4428(2)	3334(1)	23(1)
O(3)	633(2)	7301(2)	4730(1)	29(1)
O(4)	-355(2)	7350(2)	3939(1)	25(1)
O(5)	5320(3)	10916(2)	3243(1)	40(1)
C(1)	1821(3)	6014(3)	4109(1)	18(1)
C(2)	1576(3)	4774(2)	4355(1)	18(1)
C(3)	1649(3)	3893(3)	3982(1)	20(1)
C(4)	1950(3)	4533(3)	3484(1)	19(1)
C(5)	4775(3)	4560(3)	3707(1)	21(1)
C(6)	4789(3)	5439(3)	4091(1)	20(1)
C(7)	3564(3)	6445(2)	4198(1)	18(1)
C(8)	634(3)	6970(3)	4295(1)	20(1)
C(9)	-1529(4)	8289(3)	4084(1)	31(1)
C(10)	-843(5)	9540(3)	4009(2)	45(1)
C(11)	1410(3)	4454(3)	4859(1)	23(1)
C(12)	1288(4)	3216(3)	4975(1)	31(1)
C(13)	1363(4)	2332(3)	4599(1)	31(1)
C(14)	1561(3)	2654(3)	4096(1)	26(1)
C(15)	964(3)	4123(3)	3041(1)	23(1)
C(16)	-430(4)	4632(3)	2876(1)	26(1)
C(17)	-1101(4)	3998(3)	2463(1)	37(1)
C(18)	-235(4)	3012(3)	2323(1)	37(1)
C(19)	6038(3)	3746(3)	3644(1)	27(1)
C(20)	7339(3)	3781(3)	3960(1)	32(1)
C(21)	7390(3)	4625(3)	4350(1)	30(1)
C(22)	6129(3)	5434(3)	4411(1)	24(1)
C(23)	3962(3)	7628(3)	3922(1)	19(1)
C(24)	4521(3)	7629(3)	3428(1)	22(1)
C(25)	4971(3)	8705(3)	3187(1)	24(1)

C(26)	4864(3)	9810(3)	3440(1)	25(1)
C(27)	4285(3)	9831(3)	3932(1)	24(1)
C(28)	3847(3)	8755(3)	4168(1)	22(1)
C(29)	6074(8)	10888(4)	2760(2)	68(2)

Table 3. Bond lengths [pm] and angles [°] for **3k**.

S(1)-C(18)	171.5(3)
S(1)-C(15)	171.7(3)
O(1)-C(4)	141.1(3)
O(1)-C(1)	143.0(3)
O(2)-C(5)	140.4(3)
O(2)-C(4)	142.8(3)
O(3)-C(8)	120.6(3)
O(4)-C(8)	131.8(3)
O(4)-C(9)	146.6(3)
O(5)-C(26)	136.5(3)
O(5)-C(29)	142.2(4)
C(1)-C(2)	151.2(4)
C(1)-C(8)	151.8(4)
C(1)-C(7)	154.6(4)
C(2)-C(3)	137.6(4)
C(2)-C(11)	138.3(4)
C(3)-C(14)	138.3(4)
C(3)-C(4)	150.8(4)
C(4)-C(15)	149.9(4)
C(5)-C(19)	138.7(4)
C(5)-C(6)	139.3(4)
C(6)-C(22)	140.3(4)
C(6)-C(7)	152.4(4)
C(7)-C(23)	151.6(4)
C(7)-H(7)	99(3)
C(9)-C(10)	148.9(5)
C(9)-H(9A)	96(3)
C(9)-H(9B)	102(3)
C(10)-H(10A)	97(5)
C(10)-H(10B)	95(4)
C(10)-H(10C)	101(4)
C(11)-C(12)	138.4(4)
C(11)-H(11)	100(3)
C(12)-C(13)	138.4(4)

C(12)-H(12)	90(3)
C(13)-C(14)	138.3(4)
C(13)-H(13)	94(3)
C(14)-H(14)	97(3)
C(15)-C(16)	136.0(4)
C(16)-C(17)	140.5(4)
C(16)-H(16)	92(4)
C(17)-C(18)	134.5(5)
C(17)-H(17)	95(4)
C(18)-H(18)	92(4)
C(19)-C(20)	137.0(4)
C(19)-H(19)	97(3)
C(20)-C(21)	137.9(5)
C(20)-H(20)	96(3)
C(21)-C(22)	138.1(4)
C(21)-H(21)	96(3)
C(22)-H(22)	97(3)
C(23)-C(24)	138.6(4)
C(23)-C(28)	139.0(4)
C(24)-C(25)	138.4(4)
C(24)-H(24)	96(3)
C(25)-C(26)	138.0(4)
C(25)-H(25)	96(3)
C(26)-C(27)	138.7(4)
C(27)-C(28)	137.5(4)
C(27)-H(27)	93(3)
C(28)-H(28)	91(3)
C(29)-H(29A)	99(4)
C(29)-H(29B)	106(5)
C(29)-H(29C)	94(5)
C(18)-S(1)-C(15)	91.62(16)
C(4)-O(1)-C(1)	108.6(2)
C(5)-O(2)-C(4)	118.2(2)
C(8)-O(4)-C(9)	116.8(2)
C(26)-O(5)-C(29)	116.5(3)

O(1)-C(1)-C(2)	103.8(2)
O(1)-C(1)-C(8)	110.2(2)
C(2)-C(1)-C(8)	112.6(2)
O(1)-C(1)-C(7)	109.8(2)
C(2)-C(1)-C(7)	109.4(2)
C(8)-C(1)-C(7)	110.9(2)
C(3)-C(2)-C(11)	121.3(3)
C(3)-C(2)-C(1)	107.9(2)
C(11)-C(2)-C(1)	130.7(2)
C(2)-C(3)-C(14)	121.3(3)
C(2)-C(3)-C(4)	108.1(2)
C(14)-C(3)-C(4)	130.4(2)
O(1)-C(4)-O(2)	109.8(2)
O(1)-C(4)-C(15)	107.8(2)
O(2)-C(4)-C(15)	106.6(2)
O(1)-C(4)-C(3)	104.3(2)
O(2)-C(4)-C(3)	111.4(2)
C(15)-C(4)-C(3)	116.9(2)
C(19)-C(5)-C(6)	121.2(3)
C(19)-C(5)-O(2)	112.8(2)
C(6)-C(5)-O(2)	125.9(2)
C(5)-C(6)-C(22)	116.3(3)
C(5)-C(6)-C(7)	128.5(2)
C(22)-C(6)-C(7)	115.2(2)
C(23)-C(7)-C(6)	111.9(2)
C(23)-C(7)-C(1)	113.0(2)
C(6)-C(7)-C(1)	112.6(2)
C(23)-C(7)-H(7)	108.0(15)
C(6)-C(7)-H(7)	106.5(15)
C(1)-C(7)-H(7)	104.2(16)
O(3)-C(8)-O(4)	125.9(3)
O(3)-C(8)-C(1)	120.9(2)
O(4)-C(8)-C(1)	113.1(2)
O(4)-C(9)-C(10)	110.2(3)
O(4)-C(9)-H(9A)	102.2(18)
C(10)-C(9)-H(9A)	114.1(18)

O(4)-C(9)-H(9B)	107.4(19)
C(10)-C(9)-H(9B)	111.4(19)
H(9A)-C(9)-H(9B)	111(3)
C(9)-C(10)-H(10A)	110(3)
C(9)-C(10)-H(10B)	110(2)
H(10A)-C(10)-H(10B)	109(3)
C(9)-C(10)-H(10C)	107(2)
H(10A)-C(10)-H(10C)	111(3)
H(10B)-C(10)-H(10C)	109(3)
C(2)-C(11)-C(12)	117.7(3)
C(2)-C(11)-H(11)	120.3(16)
C(12)-C(11)-H(11)	122.0(16)
C(11)-C(12)-C(13)	121.0(3)
C(11)-C(12)-H(12)	118(2)
C(13)-C(12)-H(12)	121(2)
C(14)-C(13)-C(12)	121.2(3)
C(14)-C(13)-H(13)	117.6(18)
C(12)-C(13)-H(13)	121.3(18)
C(13)-C(14)-C(3)	117.5(3)
C(13)-C(14)-H(14)	122.5(19)
C(3)-C(14)-H(14)	120.0(19)
C(16)-C(15)-C(4)	126.8(3)
C(16)-C(15)-S(1)	110.9(2)
C(4)-C(15)-S(1)	122.2(2)
C(15)-C(16)-C(17)	112.9(3)
C(15)-C(16)-H(16)	120(2)
C(17)-C(16)-H(16)	127(2)
C(18)-C(17)-C(16)	113.1(3)
C(18)-C(17)-H(17)	123(2)
C(16)-C(17)-H(17)	124(2)
C(17)-C(18)-S(1)	111.5(3)
C(17)-C(18)-H(18)	129(2)
S(1)-C(18)-H(18)	119(2)
C(20)-C(19)-C(5)	120.8(3)
C(20)-C(19)-H(19)	121.7(18)
C(5)-C(19)-H(19)	117.5(18)

C(19)-C(20)-C(21)	119.8(3)
C(19)-C(20)-H(20)	119.5(18)
C(21)-C(20)-H(20)	120.7(18)
C(20)-C(21)-C(22)	119.2(3)
C(20)-C(21)-H(21)	118.1(17)
C(22)-C(21)-H(21)	122.7(17)
C(21)-C(22)-C(6)	122.6(3)
C(21)-C(22)-H(22)	122.6(17)
C(6)-C(22)-H(22)	114.8(17)
C(24)-C(23)-C(28)	117.5(3)
C(24)-C(23)-C(7)	121.8(2)
C(28)-C(23)-C(7)	120.6(2)
C(25)-C(24)-C(23)	121.7(3)
C(25)-C(24)-H(24)	118.5(17)
C(23)-C(24)-H(24)	119.7(17)
C(26)-C(25)-C(24)	119.7(3)
C(26)-C(25)-H(25)	120.5(19)
C(24)-C(25)-H(25)	119.7(19)
O(5)-C(26)-C(25)	124.4(3)
O(5)-C(26)-C(27)	116.1(3)
C(25)-C(26)-C(27)	119.5(3)
C(28)-C(27)-C(26)	120.2(3)
C(28)-C(27)-H(27)	123.1(19)
C(26)-C(27)-H(27)	116.8(19)
C(27)-C(28)-C(23)	121.4(3)
C(27)-C(28)-H(28)	119.9(18)
C(23)-C(28)-H(28)	118.6(18)
O(5)-C(29)-H(29A)	113(2)
O(5)-C(29)-H(29B)	109(3)
H(29A)-C(29)-H(29B)	108(4)
O(5)-C(29)-H(29C)	104(3)
H(29A)-C(29)-H(29C)	114(3)
H(29B)-C(29)-H(29C)	108(4)

Symmetry transformations used to generate equivalent atoms:

Table 4. Anisotropic displacement parameters ($pm^2 x 10^{-1}$)for **3k**. The anisotropic displacement factor exponent takes the form: $-2\pi^2[h^2a^{*2}U^{11} + ... + 2 h k a^* b^* U^{12}]$

	U^{11}	U^{22}	U^{33}	U^{23}	U^{13}	U^{12}
S(1)	42(1)	34(1)	30(1)	-15(1)	-3(1)	0(1)
O(1)	23(1)	19(1)	15(1)	-1(1)	-1(1)	0(1)
O(2)	21(1)	27(1)	21(1)	-5(1)	1(1)	0(1)
O(3)	33(1)	33(1)	19(1)	-5(1)	2(1)	7(1)
O(4)	24(1)	26(1)	24(1)	-2(1)	-4(1)	7(1)
O(5)	72(2)	20(1)	26(1)	2(1)	7(1)	-6(1)
C(1)	20(1)	23(2)	11(1)	-1(1)	-2(1)	0(1)
C(2)	14(1)	20(1)	21(1)	1(1)	-3(1)	0(1)
C(3)	16(1)	23(2)	22(1)	1(1)	-3(1)	-1(1)
C(4)	20(1)	18(2)	20(2)	-2(1)	1(1)	2(1)
C(5)	21(1)	22(2)	22(2)	2(1)	1(1)	-3(1)
C(6)	18(1)	20(2)	22(2)	5(1)	1(1)	-2(1)
C(7)	18(1)	21(2)	15(1)	-2(1)	-2(1)	0(1)
C(8)	20(1)	19(2)	21(2)	3(1)	0(1)	-3(1)
C(9)	24(2)	32(2)	36(2)	-2(1)	-2(2)	10(1)
C(10)	47(2)	26(2)	62(3)	1(2)	10(2)	9(2)
C(11)	20(1)	30(2)	18(1)	0(1)	-1(1)	1(1)
C(12)	36(2)	34(2)	23(2)	10(1)	-3(1)	-1(2)
C(13)	37(2)	22(2)	34(2)	8(1)	-6(1)	-2(2)
C(14)	26(2)	25(2)	28(2)	0(1)	-4(1)	1(1)
C(15)	27(2)	23(2)	17(1)	-2(1)	2(1)	-5(1)
C(16)	28(2)	26(2)	25(2)	-3(1)	-5(1)	-2(1)
C(17)	40(2)	41(2)	31(2)	2(2)	-13(1)	-9(2)
C(18)	50(2)	40(2)	22(2)	-7(2)	-7(2)	-13(2)
C(19)	25(2)	24(2)	33(2)	1(1)	7(1)	2(1)
C(20)	21(2)	30(2)	44(2)	9(2)	6(1)	6(1)
C(21)	18(2)	35(2)	37(2)	11(2)	-2(1)	-2(1)
C(22)	23(2)	25(2)	22(2)	4(1)	0(1)	-2(1)
C(23)	17(1)	20(2)	19(1)	-1(1)	-4(1)	-2(1)
C(24)	27(1)	19(2)	20(1)	-3(1)	-3(1)	-2(1)
C(25)	32(2)	23(2)	17(2)	0(1)	-1(1)	-1(1)

C(26)	34(2)	19(2)	22(2)	4(1)	-2(1)	-1(1)
C(27)	33(2)	17(2)	23(2)	-3(1)	-2(1)	3(1)
C(28)	22(2)	24(2)	18(2)	-4(1)	-2(1)	1(1)
C(29)	143(5)	31(2)	29(2)	3(2)	21(3)	-28(3)

Table 5. Hydrogen coordinates (x 10^4) and isotropic displacement parameters (pm^2x 10^{-1}) for **3k**.

	x	y	z	U(eq)
H(7)	3620(30)	6610(20)	4565(10)	14(6)
H(9A)	-2420(30)	8100(30)	3865(11)	25(8)
H(9B)	-1820(40)	8140(30)	4453(13)	46(10)
H(10A)	80(60)	9650(40)	4227(15)	78(15)
H(10B)	-1620(50)	10150(40)	4084(13)	55(11)
H(10C)	-540(40)	9610(30)	3640(14)	53(11)
H(11)	1360(30)	5100(30)	5125(10)	25(7)
H(12)	1200(40)	3000(30)	5301(12)	38(9)
H(13)	1280(40)	1490(30)	4676(11)	31(8)
H(14)	1650(40)	2050(30)	3829(12)	37(9)
H(16)	-840(40)	5310(30)	3035(12)	48(11)
H(17)	-2110(50)	4200(40)	2315(14)	62(12)
H(18)	-410(40)	2470(30)	2062(13)	52(11)
H(19)	5950(30)	3150(30)	3373(12)	32(9)
H(20)	8200(40)	3210(30)	3909(11)	29(8)
H(21)	8310(40)	4620(30)	4569(10)	25(8)
H(22)	6100(30)	6040(30)	4683(11)	25(8)
H(24)	4590(30)	6870(30)	3245(11)	27(8)
H(25)	5390(40)	8670(30)	2847(12)	32(8)
H(27)	4210(30)	10600(30)	4086(11)	30(9)
H(28)	3520(30)	8770(30)	4494(11)	20(7)
H(29A)	5370(50)	10560(40)	2490(15)	63(13)
H(29B)	7110(60)	10330(50)	2779(19)	110(20)
H(29C)	6400(50)	11700(40)	2704(16)	76(13)
